# Explore the Mechanism of Astragalus mongholicus Bunge against Nonalcoholic Fatty Liver Disease Based on Network Pharmacology and Experimental Verification

**DOI:** 10.1155/2022/4745042

**Published:** 2022-04-05

**Authors:** Lili Fu, Zhongming Wu, Yanjun Chu, Wenbin Chen, Ling Gao, Shumin Mu, Jiajun Zhao

**Affiliations:** ^1^Shandong University of Traditional Chinese Medicine, Jinan, Shandong 250355, China; ^2^Department of Endocrinology, Shandong Provincial Hospital Affiliated to Shandong First Medical University, Jinan, Shandong 250021, China; ^3^Shandong Key Laboratory of Endocrinology and Lipid Metabolism, Jinan, Shandong 250021, China; ^4^Scientific Center, Shandong Provincial Hospital Affiliated to Shandong First Medical University, Jinan, Shandong 250021, China; ^5^Shandong Clinical Research Center of Diabetes and Metabolic Diseases, Jinan, Shandong 250021, China; ^6^Shandong Prevention and Control Engineering Laboratory of Endocrine and Metabolic Diseases, Jinan, Shandong 250021, China; ^7^The Affiliated Hospital of Shandong University of Traditional Chinese Medicine, Jinan, Shandong, China

## Abstract

**Objective:**

Astragalus mongholicus Bunge [Fabaceae] (AMB), a traditional Chinese medicine (TCM), has been widely used to treat liver diseases in the clinic. However, the efficacy and mechanism of AMB in the treatment of nonalcoholic fatty liver disease (NAFLD) remain unclear. The purpose of this study was to systematically investigate the active components and mechanisms of AMB against NAFLD based on network pharmacology, molecular docking, and experimental verification.

**Methods:**

First, the bioactive components and relevant targets of AMB were screened from the Traditional Chinese Medicine Systematic Pharmacology (TCMSP) database, and NAFLD-related targets were obtained from the GeneCards database. Then, the AMB-NAFLD protein target interaction network was built by the STRING database. GO and KEGG pathway enrichment analyses were performed using the DAVID database. The component targets were visualized using Cytoscape software. Finally, molecular docking and experiments were used to verify the results of network pharmacological prediction.

**Results:**

Network pharmacology predicted that quercetin may be the main active component in AMB, and the TNF and MAPK signaling pathways may be the key targets of AMB against NAFLD. Molecular docking validation results demonstrated that quercetin, as the main active component of AMB, had the highest binding affinity with TNF. Furthermore, quercetin played a distinct role in alleviating NAFLD through in vitro experiments. Quercetin upregulated the phosphorylation levels of AMPK and inhibited the expression of p-MAPK and TNF-*α*. In addition, we further discovered that quercetin could increase ACC phosphorylation and CPT1*α* expression in PA-induced HepG2 cells.

**Conclusions:**

Our results indicated that quercetin, as the main active component in AMB, exerts an anti-NAFLD effect by regulating the AMPK/MAPK/TNF-*α* and AMPK/ACC/CPT1*α* signaling pathways to inhibit inflammation and alleviate lipid accumulation.

## 1. Introduction

Nonalcoholic fatty liver disease (NAFLD) refers to excessive lipid deposition in hepatocytes that has become a leading cause of chronic liver disease worldwide, with a prevalence of 25% in adults [1, 2]. NAFLD has a wide range of hepatic pathological features that range from simple hepatic lipid accumulation to nonalcoholic steatohepatitis (NASH) and even progress to cirrhosis and hepatocellular carcinoma. Due to this progressive feature, even NAFLD patients with simple steatosis may eventually lead to an increase in all-cause mortality [3]. Multiple parallel hits have been widely used to explain the pathogenesis of NAFLD, including inflammation, insulin resistance, and oxidative stress [4]. NAFLD has become a global public health issue that cannot be ignored. However, there are currently no approved drugs to treat NAFLD. Paying attention to a healthy diet and regular exercise are the primary therapeutic modalities for NAFLD, but few adherents have been successful [5]. Therefore, developing natural products with clinical therapeutic potential is of great significance and has received widespread attention from society in recent years.

Astragalus mongholicus Bunge [Fabaceae] (ABM) is one of the most widely used traditional Chinese medicines (TCMs) in clinics and is widely distributed in Northeast, North, and Northwest China as well as in Mongolia and Korea [6]. The root of AMB [Fabaceae] is medicinal and has been used for many years in traditional Chinese medicine to treat chronic fatigue, weakness, anemia, loss of appetite, uterine bleeding, and uterine prolapses [7]. Modern pharmacology has confirmed that this herb possesses a variety of activities, including regulating immunity [8], anti-inflammatory [9], antioxidant [10], and antihyperglycemic [9] activities. To date, it has been reported that more than 100 compounds have been isolated and identified in AMB, such as saponins, flavonoids, polysaccharide amino acids, and trace elements with various biological activities [11]. Previous studies suggested that AMB possessed hepatoprotective effects. For example, Huang-Qi San had a significant effect on improving glucose, lipid metabolism, and liver steatosis in high-fat rats [12]. Notwithstanding extensive research efforts, the main active components in AMB and their anti-NAFLD mechanisms have thus far unclear.

Due to the characteristics of multicomponents and multitargets, traditional experimental methods cannot systematically explain the pharmacological mechanism of TCM. Thus, we adopted network pharmacology analysis based on bioinformatics and systems biology to carry out the research [13]. Network pharmacology systematically reveals the complex relationship between drugs and diseases by constructing biological networks and visualizing the network to analyze potential active ingredients, pivotal targets, signaling pathways, and diseases [14]. It is a powerful tool to improve drug efficacy and accelerate drug research and development. The holistic and systematic features of network pharmacology are consistent with the holistic view of TCM and the principles of syndrome differentiation and treatment. Molecular docking is a computational method used to study the interactions between molecules [15]. The purpose is to predict the binding model of small molecule drugs and large molecule proteins. It is also commonly used to verify the accuracy of network pharmacological predictions.

**Figure 1:**
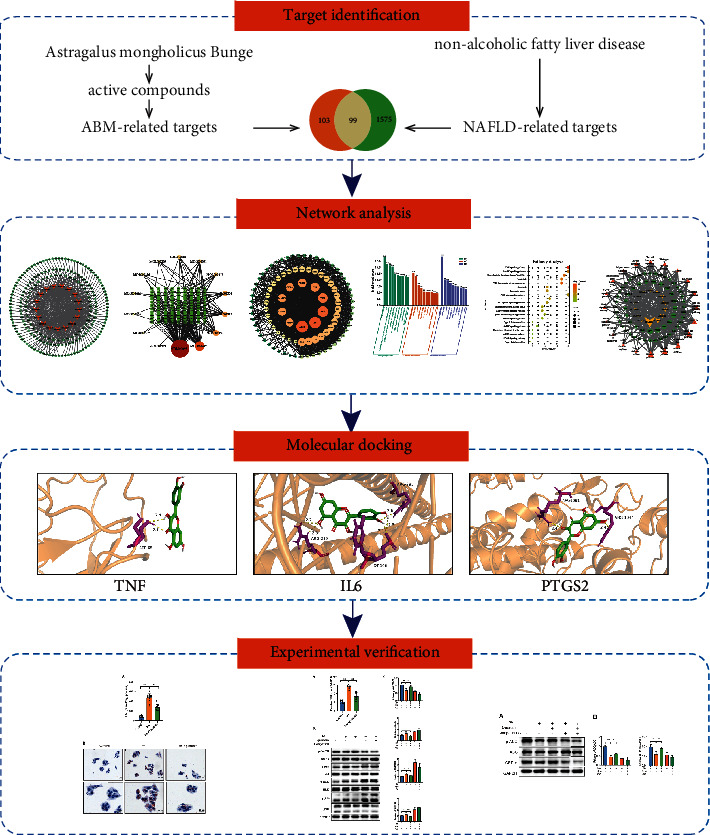
Flow chart of the pharmacological mechanisms of AMB against NAFLD.

In this study, we first predicted the bioactive compounds and mechanisms of ABM in ameliorating NAFLD based on network pharmacology. Then, molecular docking technology and experiments were utilized to verify the reliability and accuracy of the above results. To our knowledge, this study is the first to reveal the effect and mechanisms of AMB against NAFLD based on network pharmacology, molecular docking, and experimental validation. This research was carried out to provide a theoretical basis for AMB in the treatment of NAFLD. The workflow is shown in Figure 1.

## 2. Materials and Methods

### 2.1. Screening Active Components and Targets of AMB

The related chemical components of AMB were obtained from the Chinese Medicine System Pharmacology Database (TCMSP, http://lsp.nwu.edu.cn/tcmsp.php). Then, oral bioavailability (OB) ≥ 30% and drug − like quality (DL) ≥ 0.18 were defined as important ADME-related pharmacokinetic parameters for identifying active ingredients in AMB [16]. OB refers to the amount of medicine that reaches the blood circulation after oral administration [17]. DL represents the similarity between components and known drugs that can optimize pharmacokinetics [18]. Subsequently, the compound-related protein targets screened above were searched in TCMSP.

### 2.2. Predicting NAFLD-Related Targets

Information on NAFLD-related targets were obtained from the GeneCards database (https://www.genecards.org/), a comprehensive functional database that contains genomics, proteomics, and transcriptomics [19]. “nonalcoholic fatty liver disease” was selected as a keyword to search disease targets for the subsequent study. A Venn diagram was drawn based on the intersection targets of AMB and NAFLD.

### 2.3. Protein-Protein Interaction (PPI) Network Construction

The PPI network was constructed based on the STRING database (https://string-db.org/). Its function is to visually present the direct or indirect interactions between proteins. First, the common component-disease targets were entered into the STRING database and selected within the scope of “Homo sapiens”. PPI information was exported in tab-separated value (TSV) format with confidence score set to 0.4. Then, the results in TSV format were imported into Cytoscape (version 3.7.2; https://www.cytoscape.org/) software to visualize the protein interactions.

### 2.4. Network Construction

Cytoscape 3.7.2 software can be used to generate a visual network that reflects the interaction between the active compounds, potential targets, and pathways [20]. The network was composed of dots and lines. The nodes represent active ingredients, targets, or pathways, and the lines represent the interaction between them [14].

### 2.5. GO and KEGG Enrichment Pathway Analysis

Gene Ontology (GO) Knowledgebase and Kyoto Encyclopedia of Genes and Genomes (KEGG) pathway enrichment analyses of the common AMB-NAFLD targets were performed using the DAVID database (https://david.ncifcrf.gov/, ver. 6.8). GO analysis was used for gene functional classification analysis, including biological process (BP), molecular function (MF), and cell component (CC). A *P* value < 0.05 was employed for further analysis.

### 2.6. Molecular Docking Validation

To validate the binding affinities of ingredient targets, molecular docking was performed using AutoDock Vina 1.5.6. [21]. First, quercetin, the main component in AMB, was used as a ligand. The key targets in the PPI network include AKT1, IL6, TNF, TP53, JUN, PTGS2, CXCL8, MAPK8, MMP9, and CASP3, which are used as protein receptors. On the one hand, for small-molecule compound components (compounds of AMB), their 2D structures (SDF format) were downloaded from the PubChem Database (https://pubchem.ncbi.nlm.nih.gov). Then, the SDF format was transformed to PDB format by minimizing energy using ChemBio3D software. Finally, they were preprocessed and saved in PDBQT format as docking ligands in AutoDock Tools software. On the other hand, for protein receptors, their X-ray crystal structures were obtained from the Protein Data Bank (PDB) (https://www1.rcsb.org/), including AKT1 (PDB ID: 1unq), IL6 (PDB ID: 6 mg1), TNF (PDB ID: 6q00), TP53 (PDB ID: 4cz5), JUN (PDB ID: 6osn), PTGS2 (PDB ID: 1pxx), CXCL8 (PDB ID: 4xdx), MAPK8 (PDB ID: 2xrw), MMP9 (PDB ID: 6esm), and CASP3 (PDB ID: 2dko). Afterwards, solvent and organic protein receptors were removed using PyMOL software and converted to pdbqt format through AutoDock Tools [22]. Finally, the location of the grid box was determined, and molecular docking results were visualized using AutoDock Vina. The binding activity between ligand and protein was evaluated by Vina score; the lower the Vina score was, the higher the binding affinity. The docking results were visualized by PyMOL software.

### 2.7. Chemicals and Reagents

Quercetin (C15H10O7, CAS No. 117-39-5, Cat No. A0083) were purchased from Chengdu Must Biotechnology Co. Ltd. (Chengdu, China). The purity of quercetin was >98% and kept protected from light and refrigerated at 4°C. Palmitic acid (PA) was dissolved in ethanol and mixed with fatty acid-free bovine serum albumin (BSA) to prepare stock solutions. The AMPK inhibitor compound C (P5499) was purchased from Sigma-Aldrich.

### 2.8. Cell Culture and Treatment

HepG2 cells were cultured in DMEM containing 10% fetal bovine serum (FBS) and 1% 100 U/mL penicillin–streptomycin at 37°C in a 5% CO_2_ atmosphere. To mimic the NAFLD model in vitro, HepG2 cells were grown in medium containing palmitic acid (PA) at a concentration of 0.4 mM. The experiment was divided into three groups: the control group (BSA+DMSO), PA group (0.4 mM PA+DMSO), and PA+quercetin group (0.4 mM PA+25 *μ*M quercetin). The overall experimental time was 24 hours. Moreover, HepG2 cells were cultured in the absence or presence of compound C (20 *μ*M) to verify the specificity of quercetin in AMPK activation.

### 2.9. Cell Lipid Content Assay

Hepatocyte TG content was quantified using a commercial kit (Applygen Technologies Inc., Beijing, China). All experimental manipulations were performed in accordance with the manufacturer's instructions. In addition, oil red O staining was also used to assess lipid accumulation in cells. The cells were immobilized in 10% paraformaldehyde and then stained shielded from light. All pathological images were observed using a light microscope.

### 2.10. Quantitative Real-Time PCR

Total RNA was isolated from cell cultures using RNAiso plus reagent (TaKaRa) according to the manufacturer's instructions. Reverse transcription was performed using the PrimeScript RT reagent kit (TaKaRa). Real-time PCR was performed on a Roche LightCycler 480 (Roche, Mannheim, Germany) using SYBR Green (Bestar qPCR Mastermix, DBI, Germany). Relative gene expression levels were calculated by the 2^-△△CT^ method, and the results are expressed as the fold change relative to the control. The PCR primers used are shown in Table 1.

### 2.11. Western Blot Analysis

Total proteins were extracted from HepG2 cells using protease inhibitors and phosphatase inhibitors (Bimake, Houston, USA). Protein concentrations were measured using a BCA Protein Quantitative Assay Kit. The target proteins were blotted with the following antibodies: anti-phospho-p38 (CST, 4511), anti-p38 (CST, 9212), anti-phospho-ERK (CST, 4376), anti-ERK (CST, 4695), anti-phospho-JNK (CST, 4668), anti-JNK (CST, 9252), anti-phospho-AMPK (CST, 2535), anti-AMPK (CST, Thr172, 2532S), anti-phospho-ACC (CST, Ser79, 11818), anti-ACC (CST, 3662), anti-CPT1*α* (Abcam, ab128568), and anti-GAPDH (Proteintech, 60004-1-Ig). The appropriate secondary antibodies conjugated to horseradish peroxidase (HRP) (Amersham, Little Chalfont Bucks, UK) were used at a 1 : 5000 dilution. An Alpha Q detection system was used to visualize the bound primary antibodies.

### 2.12. Statistical Analysis

The data are presented as the mean ± standard deviation (SD). Statistical analysis was performed in GraphPad Prism 8.0 software. One-way analysis of variance (ANOVA) was performed to examine differences between multiple groups. A *P* value < 0.05 was considered statistically significant.

## 3. Results

### 3.1. The Active Compounds and Targets in AMB

**Figure 2:**
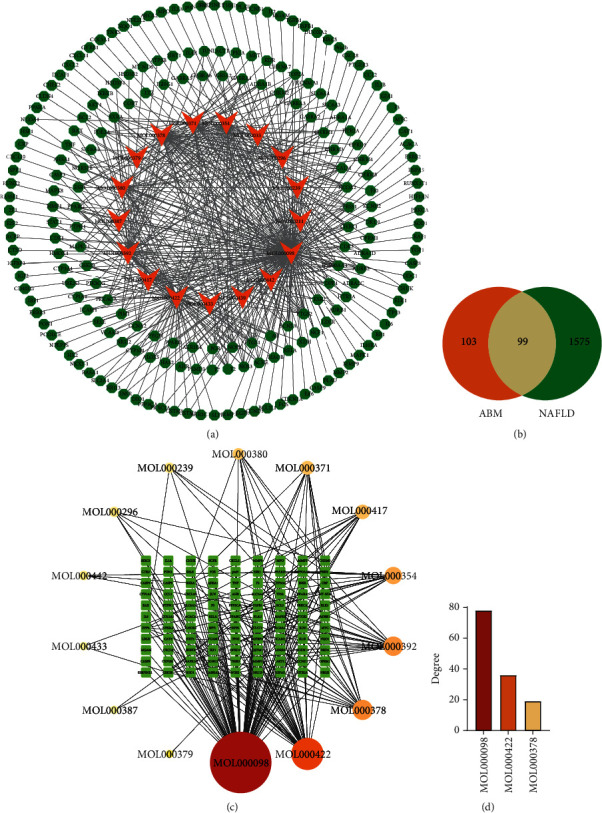
Compound-target network construction. (a) The component-target network of AMB. The orange triangles represent active compounds, and the green nodes represent component-related targets. (b) Venn diagram. The orange part represents the number of AMB targets, and the green part represents the number of NAFLD targets. (c) Compound-NAFLD targets network. The circular nodes represent potential active components in AMB, and the square represents the 99 common targets of AMB and NAFLD. (d) Degree of potential active compounds.

Eighty-seven components of AMB were acquired from TCMSP. According to the screening standards of OB ≥ 30% and DL ≥ 0.18, 20 active components of AMB were finally obtained for subsequent research and analysis (Table 2). Then, 450 active component-targets of AMB were screened using the TCMSP database, and 202 component-targets were finally identified after the deletion of duplicates (Supplementary Table S1). To elucidate intuitive interactions between the components of AMB and their targets, we constructed a compound-target network using Cytoscape 3.7.2 (Figure 2(a)). The network contains 219 nodes and 465 edges, which means that one compound can correspond to multiple targets. The top three active ingredients were quercetin, kaempferol, and 7-O-methylisomucronulatol, which correspond to 142, 61, and 44 targets, respectively. The network suggested that these components may serve as the main therapeutic ingredients of AMB anti-NAFLD.

### 3.2. Potential Targets and Active Components of AMB in Anti-NAFLD

A total of 1674 NAFLD-related protein targets were downloaded from the GeneCards database (Supplementary Table S2). Furthermore, the 202 active component targets of AMB intersected with the NAFLD-related targets. Finally, 99 common targets were obtained and are shown in the form of a Venn diagram (Figure 2(b) and Supplementary Table S3). The 99 common targets mentioned above may be key potential targets for the treatment of NAFLD.

To identify the main anti-NAFLD effective component in AMB, we constructed a component-NAFLD target network (Figure 2(c)). The circular nodes represent potential active components in AMB, and the square represents the 99 common targets of AMB and NAFLD. The larger the node area is, the darker the color, indicating the more important components. As shown in Figure 2(d), quercetin (MOL000098) had the highest degree, suggesting that quercetin may be the most important active component of AMB against NAFLD.

### 3.3. PPI Network Analysis

**Figure 3:**
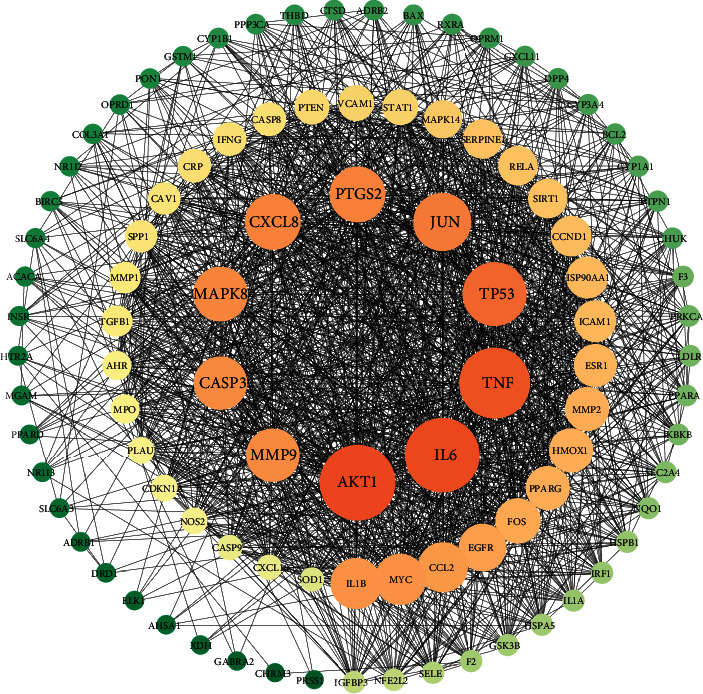
The protein-protein interaction (PPI) network analyze of overlapping targets. The color and depth of the nodes (orange → yellow → green) are in descending order of degree values, and node sizes are proportional to their degree.

To assess the protein-protein interactions, 99 common targets were uploaded to the STRING database. Then, Cytoscape 3.7.2 was used to build a visual PPI network (Figure 3). There were 97 nodes and 1476 edges in the PPI network. The larger and darker the color of nodes, the denser the lines indicating that the protein is more important. Finally, 10 hub targets were screened out according to betweenness (BC), and closeness (CC) values were ≥2 × median. [23] The top ten targets were AKT1, IL6, TNF, TP53, JUN, PTGS2, CXCL8, MAPK8, MMP9, and CASP3 (Table 3). These targets are likely to be the key targets of AMB in the treatment of NAFLD. Notably, these key targets were predicted mainly from quercetin.

### 3.4. GO Enrichment Analysis

**Figure 4:**
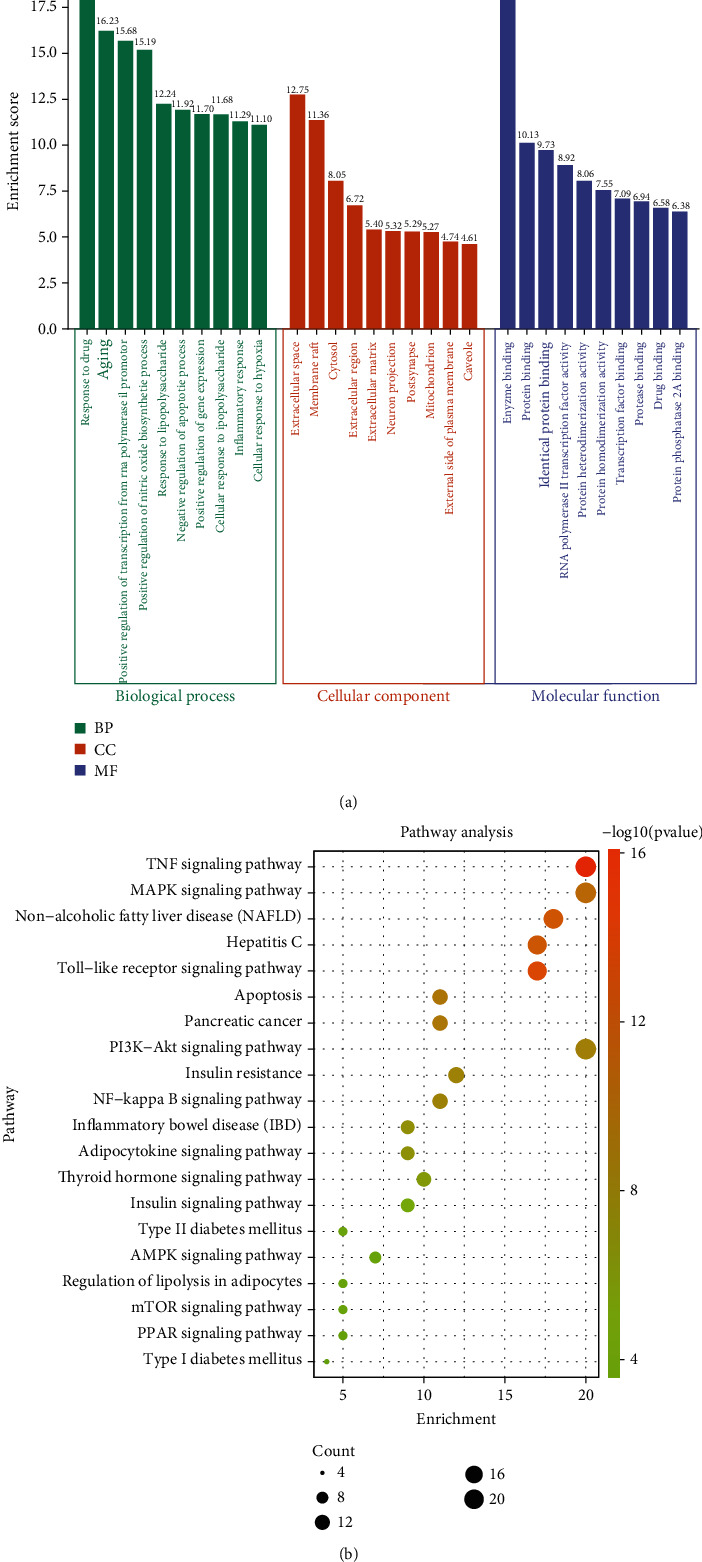
Overlapped term-based analysis. (a) GO enrichment analysis for the major targets of AMB against NAFLD. The green, orange, and purple color rectangles represent biological process (BP), cellular component (CC), and molecular function (MF), respectively. (b) The top 20 KEGG pathway analysis for the major targets of AMB against NAFLD.

To elucidate the biological function of therapeutic targets, 99 common component targets were uploaded to the DAVID database. A total of 486 GO terms were enriched according to the value of the parameter (*P* ≤ 0.05). Among them, 383 GO terms were biological processes (BP) (Supplementary Table S4), 40 GO terms were cellular components (CC) (Supplementary Table S5), and 63 GO terms were molecular functions (MF) (Supplementary Table S6). The top 10 GO terms of BP, MF, and CC are shown in (Figure 4(a)).

### 3.5. KEGG Pathway Enrichment Analysis and Compound-Target-Pathway Network Construction

To further explore the signaling pathway of AMB against NAFLD, we performed KEGG pathway enrichment analysis based on 99 common component targets. After deleting the unrelated broad-spectrum pathway [24], we identified the top 20 pathways based on a *P* value < 0.05 (Figure 4(b)). Data analysis showed that targets were significantly enriched in multiple pathways, such as the TNF signaling pathway, MAPK signaling pathway, nonalcoholic fatty liver disease (NAFLD), and Toll-like receptor signaling pathway. The most significantly enriched pathway was the TNF signaling pathway.

**Figure 5:**
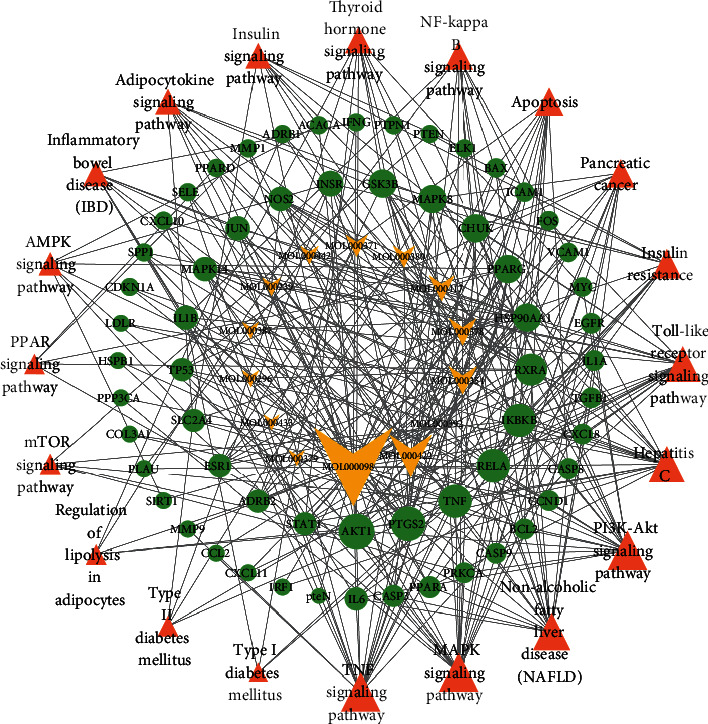
The component-target-pathway network constructed by Cytoscape. The yellow arrows represent active components of AMB, the green nodes represent the targets, and the orange triangles represent signaling pathways. Node sizes are proportional to their degree.

Moreover, to further systematically clarify the molecular mechanism of AMB against NAFLD, the top 20 significantly enriched pathways, corresponding targets, and components were used to complete a component-target-pathway network (Figure 5). The size of the graph area is proportional to the degree value. Quercetin exhibits the highest number of genes and is considered the most represented active component among AMB. The TNF and MAPK signaling pathways were significantly enriched by the corresponding targets. Collectively, these results indicate that the therapeutic effects of AMB on NAFLD are achieved in a multitarget and multipathway manner.

### 3.6. Molecular Docking Verification

**Figure 6:**
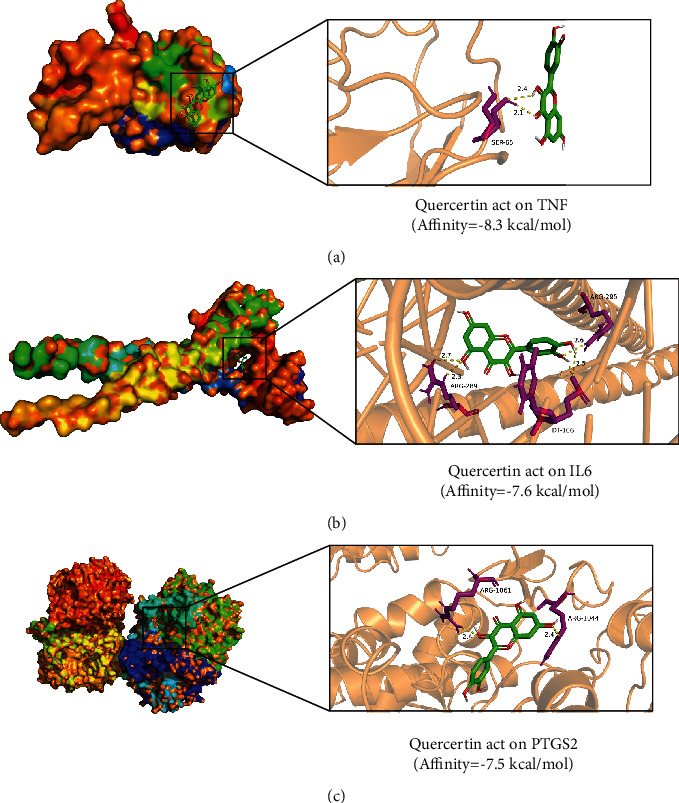
Molecular docking model of the top 3 key target proteins with the highest docking scores docked with quercetin. (a) TNF, (b) IL6, and (c) PTGS2.

To further verify the potential targets of AMB against ANFLD, ten hub genes in the PPI were screened for molecular docking analysis with quercetin. Ten key genes were AKT1, IL6, TNF, TP53, JUN, PTGS2, CXCL8, MAPK8, MMP9, and CASP3. The results are shown in Table 4. The affinity value is a vital parameter for evaluating the binding strength between receptors and ligands. The lower the affinity value is, the stronger the binding force. Generally, an affinity value < −5 kcal/mol indicates good binding affinity, and an affinity score value <−7 kcal/mol indicates stronger adhesion of the receptor to ligands. The results revealed that quercetin docked well with all the key targets, which also demonstrates the accuracy of network pharmacology. In addition, quercetin had the highest binding affinity with TNF (-8.3 kcal/mol), while it had the second and third highest binding affinity with IL6 and PTGS2 (-7.6 and -7.5 kcal/mol). Therefore, the above results indicated that TNF is expected to be the most critical target of AMB against NAFLD. Moreover, 3 pairs with the highest docking scores were selected for 3D visualization (Figure 6). The binding of quercetin to TNF mainly occurs through hydrogen bond interactions with SER-65 (Figure 6(a)). Quercetin binds to IL-6 mainly through hydrogen bonding with ARG-295, DT-106, and ARG-289 (Figure 6(b)). The combination of quercetin and PTGS2 occurs mainly through hydrogen bonding interactions of ARG-1061 and ARG-1044 (Figure 6(c)). In addition, the accuracy of network pharmacology was confirmed by molecular docking.

### 3.7. Quercetin Exhibits the Anti-NAFLD Effect in PA-Induced HepG2 cells

**Figure 7:**
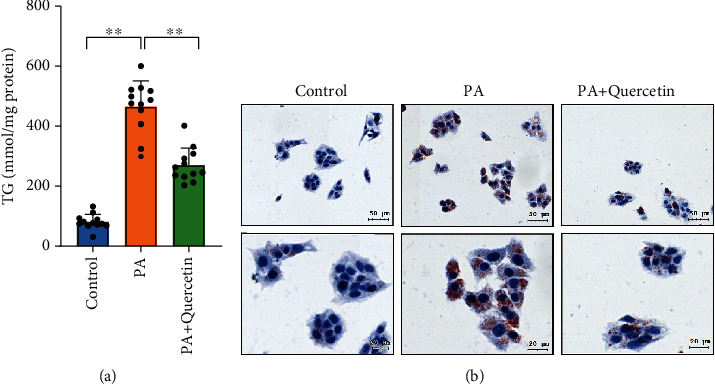
Anti-NAFLD effect of quercetin in PA-induced HepG2 cells. (a) TG contents in HepG2 cells treated with DMSO or 25 *μ*M quercetin in response to BSA or 0.4 mM PA stimulation for 24 h (*n* = 12). Data are presented as the mean ± S.E.M. ^∗^*P* < 0.05 and ^∗∗^*P* < 0.01. (b) Oil red O staining showing that PA induced lipid accumulation, which was suppressed after quercetin treatment for 24 h in HepG2 cells (*n* = 4).

To investigate the effect of quercetin on NAFLD, HepG2 cells were treated with quercetin (25 *μ*M) in the presence or absence of PA (0.4 mM) for 24 h. PA-induced HepG2 cells were used as the NAFLD model. The cell TG content results showed that TG levels were markedly higher in the PA group than in the control group. However, quercetin treatment significantly reduced cell TG levels (Figure 7(a)). The effect of quercetin on alleviating lipid accumulation in HepG2 cells was further confirmed by oil red O staining (Figure 7(b)). In summary, these studies suggested that quercetin exerts an anti-NAFLD effect in PA-induced HepG2 cells. Moreover, these results further highlighted that quercetin is the main active ingredient in AMB against NAFLD.

### 3.8. Quercetin Suppresses Inflammation by Regulating the AMPK/MAPK/TNF-*α* Signaling Pathway

**Figure 8:**
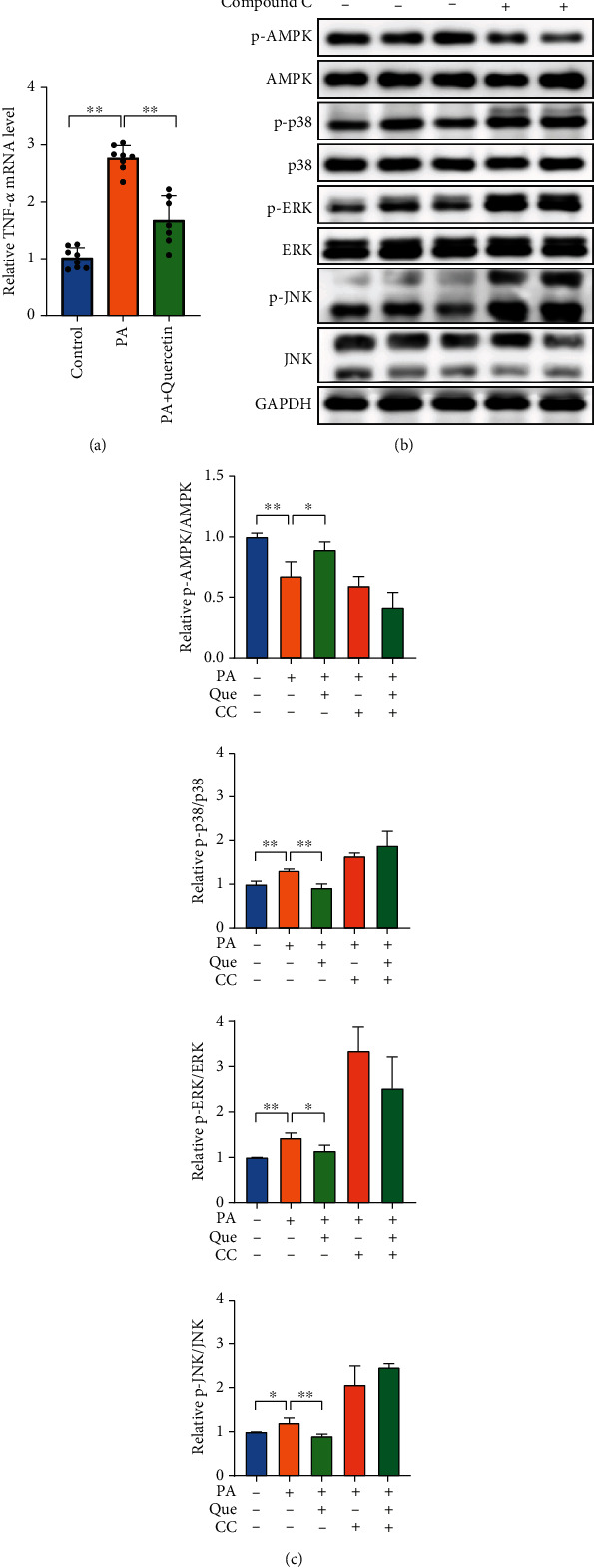
Quercetin suppresses inflammation by regulating the AMPK/MAPK/TNF-*α* signaling pathway in PA-induced HepG2 cells. (a) qPCR analyses of TNF-*α* mRNA levels in HepG2 cells stimulated by 0.4 mM PA with DMSO or 25 *μ*M quercetin for 24 h (*n* = 7 − 8). (b) Western blot analysis of p-AMPK, AMPK, p-p38, p38, p-ERK, ERK, p-JNK, and JNK levels in HepG2 cells stimulated by PA with quercetin (*n* = 3). (c) Gray value analysis of p-AMPK/AMPK, p-p38/p38, p-ERK/ERK, and p-JNK/JNK. Data are presented as the mean ± S.E.M. ^∗^*P* < 0.05 and ^∗∗^*P* < 0.01.

To explore the mechanism of AMB against NAFLD, we first tested the idea that TNF might be a key therapeutic target for NAFLD predicted by network pharmacology and molecular docking. The real-time qPCR results showed that PA significantly increased TNF-*α* mRNA expression, while quercetin remarkably decreased PA-induced TNF-*α* mRNA levels in HepG2 cells (Figure 8(a)).

TNF-*α* is known to be regulated by MAPK signaling pathways [25, 26]. In addition, the MAPK signaling pathway was regarded as a significant pathway with the highest number of enriched genes in KEGG enrichment analysis. Thus, we investigated whether quercetin influences the expression of the MAPK signaling pathway. Consistent with previous reports, PA treatment enhanced the expression of p-p38, p-ERK, and p-JNK. However, this expression trend was reversed after quercetin treatment. No significant changes were observed in total p38, ERK, or JNK levels. Furthermore, we found that quercetin upregulated the phosphorylation levels of AMPK. To determine whether AMPK activation inhibited the expression of MAPK, we applied the AMPK inhibitor compound C to PA-induced HepG2 cells. The inhibitory effect of quercetin on the MAPK pathway was abolished by compound C (Figures 8(b) and 8(c)). Together, these data suggested that the AMPK/MAPK/TNF-*α* signaling pathways are involved in the anti-inflammatory effect of quercetin in PA-induced HepG2 cells.

### 3.9. Quercetin Alleviates Lipid Accumulation by Regulating the AMPK/ACC/CPT1*α* Signaling Pathway

**Figure 9:**
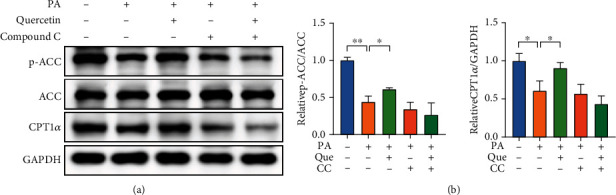
Quercetin increases fatty acid *β*-oxidation by regulating AMPK/ACC/CPT1*α* signaling in PA-induced HepG2 cells. (a) The protein expression levels of p-ACC, ACC, and CPT1*α* were examined by Western blot analysis in HepG2 cells (*n* = 3). (b) The gray value analysis of p-ACC, ACC, and CPT1*α*. Data are presented as the mean ± S.E.M. ^∗^*P* < 0.05 and ^∗∗^*P* < 0.01.

To further elucidate the mechanism by which quercetin alleviates lipid accumulation, we detected the expression of proteins related to the fatty acid *β*-oxidation pathway. Acetyl-CoA carboxylase (ACC) is a direct substrate of AMPK, and its phosphorylation level was increased after quercetin treatment. Quercetin also significantly enhanced the level of CPT1*α*. However, the upregulation of p-ACC and CPT1*α* was abrogated by compound C (Figure 9). In conclusion, these results indicated that quercetin alleviates lipid accumulation by activating AMPK/ACC/CPT1*α* signaling to increase fatty acid *β*-oxidation.

## 4. Discussion

NAFLD is a progressive disease. NAFLD refers to simple liver steatosis, which often occurs in the initial stage of the disease. NASH is defined as a more serious process accompanied by inflammation and liver cell damage, which can progress to liver cirrhosis and eventually liver cancer [27]. The complexity of the pathogenesis of NAFLD leads to significant challenges in its treatment. There are currently no relevant therapeutic drugs for NAFLD approved by the FDA [28]. To date, treatment targets focus on improving insulin resistance, reducing lipid deposition, and reducing inflammatory responses. According to relevant reports, AMB exhibits excellent hepatoprotective efficacy in clinical treatment by relieving inflammation and antioxidants [6]. However, the bioactive components and pharmacological mechanism of ABM against NAFLD have not been completely elucidated due to its multicomponent and multitarget features. Network pharmacology screens out numerous active components of TCM and comprehensively predicts multiple targets and pathways by building a systematic network [29]. Therefore, in the present study, we aimed to explore the active components and mechanisms of AMB in ameliorating NAFLD by integrating network pharmacology, molecular docking, and experimental verification.

First, network pharmacology predicted that ABM has the ability to improve NAFLD, and this effect is closely related to the regulation of the TNF-*α* and MAPK signaling pathways. Specifically, 20 active components of AMB and 202 component targets were screened from the TCMSP database. According to the topological value in the component-target network, the top three components in AMB were quercetin, kaempferol, and 7-O-methylisomucronulatol, suggesting that they are the key components of AMB anti-NAFLD. Quercetin was recognized as the component with the highest degree related to several NAFLD targets. Therefore, quercetin was selected as the most representative component of AMB for the follow-up study. Studies have indicated that quercetin protects the liver by promoting hepatic VLDL assembly [30]. Besides, quercetin improved NAFLD induced by T2DM, which was characterized by restoring abnormal liver enzymes and reducing hepatic lipid deposition in db/db mice [31]. Furthermore, quercetin not only improved liver steatosis but also alleviated liver fibrosis. The expression of proinflammatory factors and fibrogenic genes is decreased [32]. Despite numerous studies, the anti-NAFLD mechanisms remain ambiguous.

For other compounds studied, evidence showed that CYP2b9, Cyp4a12b, Mup17, Mup7, and Mup16 were differentially expressed genes for NASH treated with kaempferol through integrating transcriptomics and metabolomics [33]. Similarly, studies have confirmed that kaempferol can effectively alleviate the formation and development of liver fibrosis by selectively binding receptor–like kinase 5 and downregulating the TGF-*β*1/Smad pathway [34]. In general, several main active ingredients of AMB have different degrees of therapeutic effects on NAFLD.

PPI network analysis showed that the key genes of ABM against NAFLD were mainly related to inflammatory factors. The correlations of 99 common component-disease targets were presented in the PPI network, of which 10 hub genes were AKT1, IL6, TNF, TP53, JUN, PTGS2, CXCL8, MAPK8, MMP9, and CASP3. These genes play significant roles in glucose and lipid metabolism, the inflammatory response, and cell apoptosis. Meanwhile, the PPI network showed that 99 common targets were not independent but interacted, suggesting that AMB treated NAFLD by regulating multiple proteins.

KEGG enrichment analysis indicated that ABM may exert its anti-NAFLD effect by regulating inflammation and metabolism-related pathways, such as the TNF signaling pathway, MAPK signaling pathway, Toll-like receptor signaling pathway, PI3K-Akt signaling pathway, insulin resistance, NF-kappa B signaling pathway, AMPK signaling pathway, and other signaling pathways. Hepatic lipid accumulation is the initial pathological hallmark of NAFLD, which can lead to inflammation, oxidative stress, and eventually liver cancer in the absence of effective interventions [35]. Here, we selected pathways with good correlation to discuss the mechanism of AMB in the treatment of NAFLD. The TNF signaling pathway was the most significantly enriched pathway, and the results were further verified by molecular docking. In recent years, as a convenient and effective emerging technology, molecular docking has been commonly used to predict the binding force between components and targets. All these results suggested that the TNF signaling pathway may be a potential effective target for AMB against NAFLD through network pharmacology analysis and molecular docking.

To further validate the results predicted by network pharmacology, in vitro experiments were performed. First, HepG2 cells were induced with PA to form a NAFLD model. NAFLD is characterized by excessive lipid deposition in hepatocytes, mainly triglycerides [36]. Our in vitro experimental results showed that quercetin significantly reduced the triglyceride content in HepG2 cells, which was further supported by oil red O staining. Based on the above evidence, we believe that quercetin has a role in ameliorating NAFLD.

Next, we examined the mechanism of AMB against NAFLD. Excessive lipid accumulation in hepatocytes is the pathological feature of NAFLD in the initial stage. Excessive fatty acids produce lipotoxic species that lead to inflammasome activation, oxidative stress, and ER stress. Inflammation, which plays a critical role in the pathogenesis of NASH, can promote the progression of liver fibrosis to cirrhosis [4]. In this study, we found that the TNF-*α* mRNA level was also significantly decreased by quercetin treatment compared to the PA group. TNF is an inflammatory cytokine with multiple biological effects, including promoting cell growth, differentiation, and apoptosis and inducing inflammation [37]. TNF is composed of TNF-*α* secreted by macrophages and TNF-*β* produced by T lymphocytes, of which TNF-*α* accounts for 70% to 95% of the total activity [38]. TNF-*α* is the accelerator that promotes the progression of hepatic steatosis to steatohepatitis and ultimately to liver fibrosis. A study including 52 obese patients demonstrated that the liver TNF-*α* mRNA level was higher in NASH patients than in the control group. Next, NASH patients were further classified according to the presence or absence of fibrosis. It was found that liver TNF-*α* mRNA expression in NASH patients with liver fibrosis was stronger than that in NASH patients without fibrosis. In addition, TNF-*α* mRNA expression in the adipose tissue of NASH patients with inflammation was strikingly elevated [39]. Another study revealed that TNF-*α* can activate stellate cells and accelerate the progression of NASH. Moreover, TNF also promoted insulin resistance and ultimately led to increased hepatic steatosis in patients with NAFLD [40]. Therefore, the development of a TNF-*α* inhibitor may be an effective therapeutic strategy for NAFLD. In an experiment, NASH model mice were given intraperitoneal injection of infliximab. As a result, anti-TNF-*α* reduced AST and ALT levels and ameliorated hepatic inflammation, necrosis, and fibrosis compared to the control group intraperitoneally injected with sterile saline solution [41]. Similarly, another study indicated that HFD-induced mice that received injection of infliximab had lower liver levels of IL-6, IL-1*β*, and IL-10 than HFD model mice. Furthermore, infliximab also improved insulin resistance and inhibited hepatic lipid deposition and fibrosis [42]. Altogether, the above results verified that TNF is a key target of AMB in the treatment of NAFLD, which also confirmed the accuracy of network pharmacology and molecular docking technology.

Interestingly, we also discovered that quercetin could downregulate the expression of MAPK signaling pathways, including ERK, p38, and JNK. The MAPK signaling pathway is closely related to cell growth, differentiation, apoptosis, and inflammation and consists of extracellular signal-regulated kinase (ERK), Jun N-terminal kinase (JNK), and p38 [43, 44]. The MAPK signaling pathway has attracted wide attention due to its involvement in regulating the expression of multiple genes related to NAFLD. Liver lipid deposition, inflammation, and fibrosis can be improved by suppressing the MAPK pathway in HFD-induced mice [45]. AMB extract can effectively inhibit the secretion and expression of IL-1*β* and TNF-*α* in macrophages. The mechanism is closely related to the regulation of the p38 MAPK and NF-*κ*B signaling pathways [46]. In addition, the MAPK signaling pathway was another significant pathway with the highest number of enriched genes in KEGG enrichment analysis.

Furthermore, in the present study, we found that quercetin could increase AMPK phosphorylation levels and that the inhibitory effect of quercetin on the MAPK signaling pathway was abolished by the AMPK inhibitor compound C in PA-induced HepG2 cells. These results indicate that the AMPK signaling pathway regulates the expression of MAPK and that MAPK is a downstream protein of AMPK. In addition, previous studies have illustrated that TNF-*α* can be activated by MAPK signaling pathways [47]. In summary, these data demonstrated that quercetin could ameliorate NAFLD by regulating the AMPK/MAPK/TNF-*α* signaling pathway to inhibit the inflammatory response.

Beyond this, we further explored the mechanism by which quercetin improves lipid metabolism because quercetin alleviates lipid accumulation in HepG2 cells, which was also consistent with literature reports [31, 48]. Hepatic lipid deposition is caused by an imbalance between lipogenesis and lipolysis. Mitochondrial fatty acid *β*-oxidation accelerates lipolysis, which contributes to reducing lipid deposition in hepatocytes. AMPK is regarded as a key factor that regulates energy metabolism homeostasis, and AMPK signaling pathway activation has been confirmed to have a protective effect on NAFLD [49]. ACC catalyzes the production of malonyl-CoA, which is an allosteric inhibitor of CPT1 [50]. Phosphorylation of ACC by AMPK inactivates ACC, which enhances the activity of CPT1 and fatty acid *β*-oxidation. In our current study, quercetin treatment significantly increased AMPK and ACC phosphorylation levels, which inhibited ACC activity, and ultimately, the expression of CPT1*α* was upregulated. However, this effect was abolished by the AMPK inhibitor compound C. Altogether, quercetin alleviated hepatic lipid accumulation by enhancing fatty acid *β*-oxidation through activating the AMPK/ACC/CPT1*α* signaling pathway in PA-induced HepG2 cells.

However, there were several limitations in the present study. First, although quercetin was considered to be the main component of AMB proven to have an anti-NAFLD effect, it could not fully represent AMB. Therefore, to elucidate the mechanism of AMB in the treatment of NAFLD, further experiments are essential. Second, this study only confirmed the effect and mechanism of quercetin against NAFLD in vitro. Thus, further in vivo experiments and clinical trials are needed to verify our conclusions.

## 5. Conclusion

**Figure 10:**
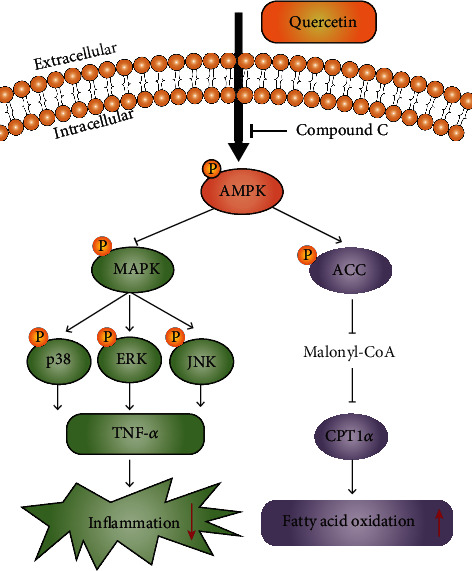
Quercetin exerts an anti-NAFLD effect by regulating the AMPK/MAPK/TNF-*α* and AMPK/ACC/CPT1*α* signaling pathways to inhibit inflammation and enhance fatty acid *β*-oxidation. Activation of AMPK by quercetin leads to downregulation of MAPK signaling pathways and decreases the expression of TNF-*α*, which may contribute to relieve inflammation. In addition, phospho-AMPK phosphorylates ACC, the rate-limiting enzyme of de novo lipogenesis. This decrease in ACC activity limits malonyl CoA production, relieving the inhibition of CPT-1*α* activity and enhancing fatty acid *β*-oxidation.

In conclusion, this study adopted an integrated strategy that combined network pharmacology, molecular docking, and experimental verification to illustrate novel mechanisms of AMB in the treatment of NAFLD. Our findings revealed that quercetin, as the main active component of AMB, could inhibit the inflammatory response, enhance fatty acid *β*-oxidation, and alleviate hepatic lipid accumulation via the AMPK/MAPK/TNF-*α* and AMPK/ACC/CPT1*α* signaling pathways to exert its anti-NAFLD effect (Figure 10). This discovery not only provides a scientific basis for revealing the molecular mechanism of AMB in the treatment of NAFLD but also suggests a novel promising therapeutic strategy for anti-NAFLD.

## Figures and Tables

**Table 1:** Primer sequences of TNF-*α* and GAPDH.

Genes	Forward primer	Reverse primer
TNF-*α*	CACGCTCTTCTGCCTGCTG	GGCTTGTCACTCGGGGTTC
GAPDH	GGAGCGAGATCCCTCCAAAAT	GGCTGTTGTCATACTTCTCATGG

**Table 2:** Characteristics of active ingredients in ABM.

No.	Molecule ID	Molecule name	OB (%)	DL
1	MOL000098	Quercetin	46.43334812	0.27525
2	MOL000422	Kaempferol	41.88224954	0.24066
3	MOL000378	7-O-methylisomucronulatol	74.68613752	0.29792
4	MOL000392	Formononetin	69.67388061	0.21202
5	MOL000354	Isorhamnetin	49.60437705	0.306
6	MOL000371	3,9-Di-O-methylnissolin	53.74152673	0.47573
7	MOL000296	Hederagenin	36.91390583	0.75072
8	MOL000380	(6aR,11aR)-9,10-dimethoxy-6a,11a-dihydro-6H-benzofurano[3,2-c]chromen-3-ol	64.25545452	0.42486
9	MOL000417	Calycosin	47.75182783	0.24278
10	MOL000239	Jaranol	50.82881677	0.29148
11	MOL000387	Bifendate	31.09782391	0.66553
12	MOL000442	1,7-Dihydroxy-3,9-dimethoxy pterocarpene	39.04541112	0.47943
13	MOL000379	9,10-Dimethoxypterocarpan-3-O-*β*-D-glucoside	36.73668801	0.9243
14	MOL000433	FA	68.96043622	0.7057
15	MOL000033	(3S,8S,9S,10R,13R,14S,17R)-10,13-dimethyl-17-[(2R,5S)-5-propan-2-yloctan-2-yl]-2,3,4,7,8,9,11,12,14,15,16,17-dodecahydro-1H-cyclopenta[a]phenanthren-3-ol	36.22847056	0.78288
16	MOL000439	Isomucronulatol-7,2′-di-O-glucosiole	49.28105539	0.62065
17	MOL000211	Mairin	55.37707338	0.7761
18	MOL000374	5′-Hydroxyiso-muronulatol-2′,5′-di-O-glucoside	41.71766574	0.69251
19	MOL000398	Isoflavanone	109.9866565	0.29572
20	MOL000438	(3R)-3-(2-hydroxy-3,4-dimethoxyphenyl)chroman-7-ol	67.6674794	0.26479

OB, oral bioavailability; DL, drug-likeness.

**Table 3:** Ten key targets of ABM in treating NAFLD.

No.	Gene symbol	Description	Degree (DC)	Betweenness centrality (BC)
1	AKT1	RAC-alpha serine/threonine-protein kinase	76	0.09007483
2	IL6	Interleukin-6	75	0.06038545
3	TNF	Tumor necrosis factor	72	0.04427747
4	TP53	Cellular tumor antigen p53	66	0.02811782
5	JUN	Transcription factor AP-1	61	0.01848417
6	PTGS2	Prostaglandin G/H synthase 2	59	0.01631192
7	CXCL8	Interleukin-8	59	0.01898645
8	MAPK8	Mitogen-activated protein kinase 8	58	0.01679413
9	MMP9	Matrix metalloproteinase-9	57	0.02436033
10	CASP3	Caspase-3	57	0.01529514

**Table 4:** Molecular docking parameters and results of ten key targets with quercetin.

Compounds	Target	PDB ID	Affinity (kcal/mol)
Quercetin	TNF	6q00	-8.3
Quercetin	IL6	6mg1	-7.6
Quercetin	PTGS2	1pxx	-7.5
Quercetin	TP53	4cz5	-7.3
Quercetin	CXCL8	4xdx	-7.3
Quercetin	MAPK8	2xrw	-7.2
Quercetin	CASP3	2dko	-6.8
Quercetin	JUN	6osn	-6.6
Quercetin	AKT1	1unq	-6.2
Quercetin	MMP9	6esm	-5.7

## Data Availability

The original contributions presented in the study are included in the article/Supplementary Material, and further inquiries can be directed to the corresponding authors.

## Supplementary Materials

Supplementary Materials Table S1: the bioactive components and corresponding targets of ABM. Table S2: information of NAFLD-related targets collected from the GeneCards database. Table S3: the common targets of ABM and NAFLD. Table S4: the 383 biological process terms. Table S5: the 40 cellular component terms. Table S6: the 63 molecular function terms.
