# Supplementary material for: Explore the Mechanism of Astragalus mongholicus Bunge against Nonalcoholic Fatty Liver Disease Based on Network Pharmacology and Experimental Verification
Source: Gastroenterol Res Pract. 2022 Apr 5;2022:4745042. doi: 10.1155/2022/4745042 (PMC9005278; doi:10.1155/2022/4745042)
Supplement: Supplementary Materials — Table S1: the bioactive components and corresponding targets of ABM. Table S2: information of NAFLD-related targets collected from the GeneCards database. Table S3: the common targets of ABM and NAFLD. Table S4: the 383 biological process terms. Table S5: the 40 cellular component terms. Table S6: the 63 molecular function terms. [file 4745042.f1.zip › 4745042.f1/Table S1.pdf]

Mol ID	MolName	Symbol	Symbol（removing duplicates）
MOL000211	Mairin	PGR	PGR
MOL000239	Jaranol	NOS2	NOS2
MOL000239	Jaranol	PTGS1	PTGS1
MOL000239	Jaranol	AR	AR
MOL000239	Jaranol	SCN5A	SCN5A
MOL000239	Jaranol	PTGS2	PTGS2
MOL000239	Jaranol	ESR2	ESR2
MOL000239	Jaranol	DPP4	DPP4
MOL000239	Jaranol	HSP90AA1	HSP90AA1
MOL000239	Jaranol	CDK2	CDK2
MOL000239	Jaranol	CHEK1	CHEK1
MOL000239	Jaranol	PRSS1	PRSS1
MOL000239	Jaranol	NCOA2	NCOA2
MOL000239	Jaranol	CALM	CALM
MOL000296	hederagenin	PGR	CHRM3
MOL000296	hederagenin	NCOA2	CHRM1
MOL000296	hederagenin	CHRM3	GABRA2
MOL000296	hederagenin	CHRM1	GABRA3
MOL000296	hederagenin	GABRA2	CHRM2
MOL000296	hederagenin	GABRA3	ADRA1B
MOL000296	hederagenin	CHRM2	GABRA1
MOL000296	hederagenin	ADRA1B	GRIA2
MOL000296	hederagenin	GABRA1	GABRA6
MOL000296	hederagenin	GRIA2	GABRA5
MOL000296	hederagenin	GABRA6	IGHG1
MOL000296	hederagenin	GABRA5	LYZ
MOL000296	hederagenin	IGHG1	COBT
MOL000296	hederagenin	LYZ	RXRA
MOL000296	hederagenin	COBT	PDE3A
MOL000296	hederagenin	PTGS1	SLC6A2
MOL000296	hederagenin	SCN5A	NOR51B
MOL000296	hederagenin	PTGS2	ESR1
MOL000296	hederagenin	RXRA	PPARG
MOL000296	hederagenin	PDE3A	PTPN1
MOL000296	hederagenin	SLC6A2	MAPK14
MOL000296	hederagenin	NOR51B	GSK3B
MOL000033	(3S,8S,9S,10R,1	PGR	PIK3CG
MOL000354	isorhamnetin	NOS2	PRKACA
MOL000354	isorhamnetin	PTGS1	PIM1
MOL000354	isorhamnetin	ESR1	CCNA2
MOL000354	isorhamnetin	AR	PYGM
MOL000354	isorhamnetin	PPARG	PPARD
MOL000354	isorhamnetin	PTGS2	AKR1B1
MOL000354	isorhamnetin	PTPN1	NCOA1
MOL000354	isorhamnetin	ESR2	F7
MOL000354	isorhamnetin	DPP4	F2
MOL000354	isorhamnetin	MAPK14	NOS3
MOL000354	isorhamnetin	GSK3B	ACHE
MOL000354	isorhamnetin	HSP90AA1	MAOB
MOL000354	isorhamnetin	CDK2	RELA

MOL000354	isorhamnetin	PIK3CG	XDH
MOL000354	isorhamnetin	PRKACA	NCF1
MOL000354	isorhamnetin	PRSS1	LDLR
MOL000354	isorhamnetin	PIM1	ADRB1
MOL000354	isorhamnetin	CCNA2	HTR3A
MOL000354	isorhamnetin	NCOA2	ADRA2C
MOL000354	isorhamnetin	CALM	ADRB2
MOL000354	isorhamnetin	PYGM	ADRA1D
MOL000354	isorhamnetin	PPARD	OPRM1
MOL000354	isorhamnetin	CHEK1	DRD1
MOL000354	isorhamnetin	AKR1B1	KCNH2
MOL000354	isorhamnetin	NCOA1	F10
MOL000354	isorhamnetin	F7	CHRM5
MOL000354	isorhamnetin	F2	CHRM4
MOL000354	isorhamnetin	NOS3	OPRD1
MOL000354	isorhamnetin	ACHE	HTR2A
MOL000354	isorhamnetin	GABRA1	ADRA1A
MOL000354	isorhamnetin	MAOB	SLC6A3
MOL000354	isorhamnetin	GRIA2	SLC6A4
MOL000354	isorhamnetin	NOR51B	KCNMA1
MOL000354	isorhamnetin	RELA	TOP2A
MOL000354	isorhamnetin	XDH	CHRNA7
MOL000354	isorhamnetin	NCF1	KDR
MOL000354	isorhamnetin	LDLR	MET
MOL000371	3,9-di-O-methyln	NOS2	PKIA
MOL000371	3,9-di-O-methyln	PTGS1	LACTB
MOL000371	3,9-di-O-methyln	CHRM3	JUN
MOL000371	3,9-di-O-methyln	F2	IL4R
MOL000371	3,9-di-O-methyln	CHRM1	SIRT1
MOL000371	3,9-di-O-methyln	ESR1	ATP5B
MOL000371	3,9-di-O-methyln	ADRB1	MT-ND6
MOL000371	3,9-di-O-methyln	SCN5A	HSD3B2
MOL000371	3,9-di-O-methyln	PTGS2	HSD3B1
MOL000371	3,9-di-O-methyln	NOS3	IKBKB
MOL000371	3,9-di-O-methyln	HTR3A	AKT1
MOL000371	3,9-di-O-methyln	ADRA2C	BCL2
MOL000371	3,9-di-O-methyln	RXRA	BAX
MOL000371	3,9-di-O-methyln	ACHE	TNF
MOL000371	3,9-di-O-methyln	PDE3A	AHSA1
MOL000371	3,9-di-O-methyln	ADRA1B	CASP3
MOL000371	3,9-di-O-methyln	ADRB2	MAPK8
MOL000371	3,9-di-O-methyln	ADRA1D	MMP1
MOL000371	3,9-di-O-methyln	OPRM1	STAT1
MOL000371	3,9-di-O-methyln	GABRA1	CDK1
MOL000371	3,9-di-O-methyln	PRSS1	HMOX1
MOL000371	3,9-di-O-methyln	NCOA2	CYP3A4
MOL000371	3,9-di-O-methyln	CALM	UBE2O
MOL000378	7-O-methylisomu	NOS2	CYP1A1
MOL000378	7-O-methylisomu	PTGS1	ICAM1
MOL000378	7-O-methylisomu	DRD1	SELE
MOL000378	7-O-methylisomu	CHRM3	VCAM1
MOL000378	7-O-methylisomu	F2	NR1I2
MOL000378	7-O-methylisomu	KCNH2	CYP1B1

MOL000378	7-O-methylisomu	CHRM1	ALOX5
MOL000378	7-O-methylisomu	ESR1	HAS2
MOL000378	7-O-methylisomu	AR	AHR
MOL000378	7-O-methylisomu	ADRB1	PSMD3
MOL000378	7-O-methylisomu	SCN5A	SLC2A4
MOL000378	7-O-methylisomu	PPARG	NR1I3
MOL000378	7-O-methylisomu	F10	INSR
MOL000378	7-O-methylisomu	CHRM5	DIO1
MOL000378	7-O-methylisomu	PTGS2	PPP3CA
MOL000378	7-O-methylisomu	NOS3	PRXC1A
MOL000378	7-O-methylisomu	ADRA2C	GSTM1
MOL000378	7-O-methylisomu	CHRM4	GSTM2
MOL000378	7-O-methylisomu	RXRA	AKR1C3
MOL000378	7-O-methylisomu	OPRD1	SLPI
MOL000378	7-O-methylisomu	PDE3A	MMP3
MOL000378	7-O-methylisomu	HTR2A	EGFR
MOL000378	7-O-methylisomu	ADRA1A	CCND1
MOL000378	7-O-methylisomu	CHRM2	BCL2L1
MOL000378	7-O-methylisomu	ADRA1B	FOS
MOL000378	7-O-methylisomu	SLC6A3	CDKN1A
MOL000378	7-O-methylisomu	ADRB2	Eif6
MOL000378	7-O-methylisomu	ADRA1D	CASP9
MOL000378	7-O-methylisomu	SLC6A4	PLAU
MOL000378	7-O-methylisomu	ESR2	MMP2
MOL000378	7-O-methylisomu	GABRA1	MMP9
MOL000378	7-O-methylisomu	DPP4	MAPK1
MOL000378	7-O-methylisomu	MAPK14	IL10RA
MOL000378	7-O-methylisomu	GSK3B	RB1
MOL000378	7-O-methylisomu	HSP90AA1	IL6
MOL000378	7-O-methylisomu	CDK2	TP53
MOL000378	7-O-methylisomu	CHEK1	ELK1
MOL000378	7-O-methylisomu	PRKACA	CHUK
MOL000378	7-O-methylisomu	PRSS1	ODC1
MOL000378	7-O-methylisomu	PIM1	CASP8
MOL000378	7-O-methylisomu	CCNA2	TOP1
MOL000378	7-O-methylisomu	NCOA2	RAF1
MOL000378	7-O-methylisomu	KCNMA1	SOD1
MOL000378	7-O-methylisomu	CALM	PRKCA
MOL000379	9,10-dimethoxyp	PTGS2	HIF1AN
MOL000379	9,10-dimethoxyp	TOP2A	RUNX1T1
MOL000379	9,10-dimethoxyp	NCOA2	HSPA5
MOL000380	(6aR,11aR)-9,10-	NOS2	ERBB2
MOL000380	(6aR,11aR)-9,10-	PTGS1	ACACA
MOL000380	(6aR,11aR)-9,10-	CHRM3	CAV1
MOL000380	(6aR,11aR)-9,10-	F2	MYC
MOL000380	(6aR,11aR)-9,10-	CHRM1	F3
MOL000380	(6aR,11aR)-9,10-	ESR1	GJA1
MOL000380	(6aR,11aR)-9,10-	SCN5A	IL1B
MOL000380	(6aR,11aR)-9,10-	PTGS2	CCL2
MOL000380	(6aR,11aR)-9,10-	HTR3A	PTGER3
MOL000380	(6aR,11aR)-9,10-	RXRA	CXCL8
MOL000380	(6aR,11aR)-9,10-	ACHE	Prkcb
MOL000380	(6aR,11aR)-9,10-	ADRA1B	BIRC5

MOL000380	(6aR,11aR)-9,10-	ADRB2	DUOXA2
MOL000380	(6aR,11aR)-9,10-	ADRA1D	HSPB1
MOL000380	(6aR,11aR)-9,10-	GABRA1	TGFB1
MOL000380	(6aR,11aR)-9,10-	HSP90AA1	MGAM
MOL000380	(6aR,11aR)-9,10-	CHRNA7	IL2
MOL000380	(6aR,11aR)-9,10-	PRSS1	CCNB1
MOL000380	(6aR,11aR)-9,10-	NCOA2	PLAT
MOL000380	(6aR,11aR)-9,10-	NCOA1	THBD
MOL000380	(6aR,11aR)-9,10-	CALM	SERPINE1
MOL000380	(6aR,11aR)-9,10-	CHRM4	IFNG
MOL000387	Bifendate	PTGS2	pteN
MOL000387	Bifendate	KDR	IL1A
MOL000387	Bifendate	MET	MPO
MOL000387	Bifendate	HSP90AA1	ABCA2
MOL000387	Bifendate	KCNMA1	NFE2L2
MOL000387	Bifendate	PTGS1	NQO1
MOL000387	Bifendate	TOP2A	TNKS
MOL000392	formononetin	NOS2	COL3A1
MOL000392	formononetin	PTGS1	GYRB1
MOL000392	formononetin	CHRM1	CXCL11
MOL000392	formononetin	ESR1	CXCL2
MOL000392	formononetin	AR	DCAF1
MOL000392	formononetin	PPARG	CHEK2
MOL000392	formononetin	PTGS2	CLDN4
MOL000392	formononetin	RXRA	PPARA
MOL000392	formononetin	PDE3A	HSF1
MOL000392	formononetin	ADRA1A	CRP
MOL000392	formononetin	SLC6A3	CXCL10
MOL000392	formononetin	ADRB2	SPP1
MOL000392	formononetin	SLC6A4	RUNX2
MOL000392	formononetin	ESR2	RASSF1
MOL000392	formononetin	DPP4	2F1
MOL000392	formononetin	MAPK14	E2F2
MOL000392	formononetin	GSK3B	ACPP
MOL000392	formononetin	HSP90AA1	CTSD
MOL000392	formononetin	CDK2	IGFBP3
MOL000392	formononetin	MAOB	IGF2
MOL000392	formononetin	CHEK1	CD40LG
MOL000392	formononetin	PRKACA	IRF1
MOL000392	formononetin	PRSS1	ERBB3
MOL000392	formononetin	PIM1	PON1
MOL000392	formononetin	CCNA2	PCOLCE
MOL000392	formononetin	CALM	NPEPPS
MOL000392	formononetin	PKIA	HK2
MOL000392	formononetin	F2	NKX3-1
MOL000392	formononetin	NOS3	RASA1
MOL000392	formononetin	ACHE	
MOL000392	formononetin	LACTB	
MOL000392	formononetin	JUN	
MOL000392	formononetin	PPARG	
MOL000392	formononetin	IL4R	
MOL000392	formononetin	SIRT1	
MOL000392	formononetin	ATP5B	

MOL000392	formononetin	MT-ND6
MOL000392	formononetin	HSD3B2
MOL000392	formononetin	HSD3B1
MOL000417	Calycosin	NOS2
MOL000417	Calycosin	PTGS1
MOL000417	Calycosin	ESR1
MOL000417	Calycosin	AR
MOL000417	Calycosin	PPARG
MOL000417	Calycosin	PTGS2
MOL000417	Calycosin	RXRA
MOL000417	Calycosin	PDE3A
MOL000417	Calycosin	ESR2
MOL000417	Calycosin	DPP4
MOL000417	Calycosin	MAPK14
MOL000417	Calycosin	GSK3B
MOL000417	Calycosin	HSP90AA1
MOL000417	Calycosin	CDK2
MOL000417	Calycosin	CHEK1
MOL000417	Calycosin	PRKACA
MOL000417	Calycosin	PRSS1
MOL000417	Calycosin	PIM1
MOL000417	Calycosin	CCNA2
MOL000417	Calycosin	NCOA2
MOL000417	Calycosin	CALM
MOL000417	Calycosin	ADRB2
MOL000422	kaempferol	NOS2
MOL000422	kaempferol	PTGS1
MOL000422	kaempferol	AR
MOL000422	kaempferol	PPARG
MOL000422	kaempferol	PTGS2
MOL000422	kaempferol	HSP90AA1
MOL000422	kaempferol	PIK3CG
MOL000422	kaempferol	PRKACA
MOL000422	kaempferol	NCOA2
MOL000422	kaempferol	DPP4
MOL000422	kaempferol	PRSS1
MOL000422	kaempferol	PGR
MOL000422	kaempferol	F2
MOL000422	kaempferol	CHRM1
MOL000422	kaempferol	NOS3
MOL000422	kaempferol	GABRA2
MOL000422	kaempferol	ACHE
MOL000422	kaempferol	SLC6A2
MOL000422	kaempferol	CHRM2
MOL000422	kaempferol	ADRA1B
MOL000422	kaempferol	GABRA1
MOL000422	kaempferol	TOP2A
MOL000422	kaempferol	F7
MOL000422	kaempferol	CALM
MOL000422	kaempferol	RELA
MOL000422	kaempferol	IKBKB
MOL000422	kaempferol	AKT1
MOL000422	kaempferol	BCL2

MOL000422	kaempferol	BAX
MOL000422	kaempferol	TNF
MOL000422	kaempferol	JUN
MOL000422	kaempferol	AHSA1
MOL000422	kaempferol	CASP3
MOL000422	kaempferol	MAPK8
MOL000422	kaempferol	XDH
MOL000422	kaempferol	MMP1
MOL000422	kaempferol	STAT1
MOL000422	kaempferol	CDK1
MOL000422	kaempferol	PPARG
MOL000422	kaempferol	HMOX1
MOL000422	kaempferol	CYP3A4
MOL000422	kaempferol	UBE2O
MOL000422	kaempferol	CYP1A1
MOL000422	kaempferol	ICAM1
MOL000422	kaempferol	SELE
MOL000422	kaempferol	VCAM1
MOL000422	kaempferol	NR1I2
MOL000422	kaempferol	CYP1B1
MOL000422	kaempferol	ALOX5
MOL000422	kaempferol	HAS2
MOL000422	kaempferol	AHR
MOL000422	kaempferol	PSMD3
MOL000422	kaempferol	SLC2A4
MOL000422	kaempferol	NR1I3
MOL000422	kaempferol	INSR
MOL000422	kaempferol	DIO1
MOL000422	kaempferol	PPP3CA
MOL000422	kaempferol	PRXC1A
MOL000422	kaempferol	GSTM1
MOL000422	kaempferol	GSTM2
MOL000422	kaempferol	AKR1C3
MOL000422	kaempferol	SLPI
MOL000433	FA	CDK2
MOL000433	FA	F2
MOL000433	FA	GSK3B
MOL000439	isomucronulatol-	TOP2A
MOL000442	1,7-Dihydroxy-3,	PTGS2
MOL000442	1,7-Dihydroxy-3,	RXRA
MOL000442	1,7-Dihydroxy-3,	HSP90AA1
MOL000442	1,7-Dihydroxy-3,	PRSS1
MOL000098	quercetin	PTGS1
MOL000098	quercetin	AR
MOL000098	quercetin	PPARG
MOL000098	quercetin	PTGS2
MOL000098	quercetin	HSP90AA1
MOL000098	quercetin	PIK3CG
MOL000098	quercetin	NCOA2
MOL000098	quercetin	DPP4
MOL000098	quercetin	AKR1B1
MOL000098	quercetin	PRSS1
MOL000098	quercetin	TOP2A

MOL000098	quercetin	F2
MOL000098	quercetin	KCNH2
MOL000098	quercetin	SCN5A
MOL000098	quercetin	F10
MOL000098	quercetin	ADRB2
MOL000098	quercetin	MMP3
MOL000098	quercetin	PRKACA
MOL000098	quercetin	F7
MOL000098	quercetin	NOS3
MOL000098	quercetin	RXRA
MOL000098	quercetin	ACHE
MOL000098	quercetin	GABRA1
MOL000098	quercetin	MAOB
MOL000098	quercetin	RELA
MOL000098	quercetin	EGFR
MOL000098	quercetin	AKT1
MOL000098	quercetin	CCND1
MOL000098	quercetin	BCL2
MOL000098	quercetin	BCL2L1
MOL000098	quercetin	FOS
MOL000098	quercetin	CDKN1A
MOL000098	quercetin	Eif6
MOL000098	quercetin	BAX
MOL000098	quercetin	CASP9
MOL000098	quercetin	PLAU
MOL000098	quercetin	MMP2
MOL000098	quercetin	MMP9
MOL000098	quercetin	MAPK1
MOL000098	quercetin	IL10RA
MOL000098	quercetin	RB1
MOL000098	quercetin	TNF
MOL000098	quercetin	JUN
MOL000098	quercetin	IL6
MOL000098	quercetin	CHEK1
MOL000098	quercetin	AHSA1
MOL000098	quercetin	CASP3
MOL000098	quercetin	TP53
MOL000098	quercetin	ELK1
MOL000098	quercetin	CHUK
MOL000098	quercetin	ODC1
MOL000098	quercetin	XDH
MOL000098	quercetin	CASP8
MOL000098	quercetin	TOP1
MOL000098	quercetin	RAF1
MOL000098	quercetin	SOD1
MOL000098	quercetin	PRKCA
MOL000098	quercetin	MMP1
MOL000098	quercetin	HIF1AN
MOL000098	quercetin	STAT1
MOL000098	quercetin	RUNX1T1
MOL000098	quercetin	CDK1
MOL000098	quercetin	HSPA5
MOL000098	quercetin	ERBB2

MOL000098	quercetin	PPARG
MOL000098	quercetin	ACACA
MOL000098	quercetin	HMOX1
MOL000098	quercetin	CYP3A4
MOL000098	quercetin	CAV1
MOL000098	quercetin	MYC
MOL000098	quercetin	F3
MOL000098	quercetin	GJA1
MOL000098	quercetin	CYP1A1
MOL000098	quercetin	ICAM1
MOL000098	quercetin	IL1B
MOL000098	quercetin	CCL2
MOL000098	quercetin	SELE
MOL000098	quercetin	VCAM1
MOL000098	quercetin	PTGER3
MOL000098	quercetin	CXCL8
MOL000098	quercetin	Prkcb
MOL000098	quercetin	BIRC5
MOL000098	quercetin	DUOXA2
MOL000098	quercetin	NOS3
MOL000098	quercetin	HSPB1
MOL000098	quercetin	TGFB1
MOL000098	quercetin	MGAM
MOL000098	quercetin	IL2
MOL000098	quercetin	NR1I2
MOL000098	quercetin	CYP1B1
MOL000098	quercetin	CCNB1
MOL000098	quercetin	PLAT
MOL000098	quercetin	THBD
MOL000098	quercetin	SERPINE1
MOL000098	quercetin	IFNG
MOL000098	quercetin	ALOX5
MOL000098	quercetin	pteN
MOL000098	quercetin	IL1A
MOL000098	quercetin	MPO
MOL000098	quercetin	TOP2A
MOL000098	quercetin	NCF1
MOL000098	quercetin	ABCA2
MOL000098	quercetin	HAS2
MOL000098	quercetin	NFE2L2
MOL000098	quercetin	NQO1
MOL000098	quercetin	TNKS
MOL000098	quercetin	AHR
MOL000098	quercetin	PSMD3
MOL000098	quercetin	SLC2A4
MOL000098	quercetin	COL3A1
MOL000098	quercetin	GYRB1
MOL000098	quercetin	CXCL11
MOL000098	quercetin	CXCL2
MOL000098	quercetin	DCAF1
MOL000098	quercetin	NR1I3
MOL000098	quercetin	CHEK2
MOL000098	quercetin	INSR

MOL000098	quercetin	CLDN4
MOL000098	quercetin	PPARA
MOL000098	quercetin	PPARD
MOL000098	quercetin	HSF1
MOL000098	quercetin	CRP
MOL000098	quercetin	CXCL10
MOL000098	quercetin	CHUK
MOL000098	quercetin	SPP1
MOL000098	quercetin	RUNX2
MOL000098	quercetin	RASSF1
MOL000098	quercetin	2F1
MOL000098	quercetin	E2F2
MOL000098	quercetin	ACPP
MOL000098	quercetin	CTSD
MOL000098	quercetin	IGFBP3
MOL000098	quercetin	IGF2
MOL000098	quercetin	CD40LG
MOL000098	quercetin	IRF1
MOL000098	quercetin	ERBB3
MOL000098	quercetin	PON1
MOL000098	quercetin	DIO1
MOL000098	quercetin	PCOLCE
MOL000098	quercetin	NPEPPS
MOL000098	quercetin	HK2
MOL000098	quercetin	NKX3-1
MOL000098	quercetin	RASA1
MOL000098	quercetin	PRXC1A
MOL000098	quercetin	GSTM1
MOL000098	quercetin	GSTM2
