# Supplementary material for: Explore the Mechanism of Astragalus mongholicus Bunge against Nonalcoholic Fatty Liver Disease Based on Network Pharmacology and Experimental Verification
Source: Gastroenterol Res Pract. 2022 Apr 5;2022:4745042. doi: 10.1155/2022/4745042 (PMC9005278; doi:10.1155/2022/4745042)
Supplement: Supplementary Materials — Table S1: the bioactive components and corresponding targets of ABM. Table S2: information of NAFLD-related targets collected from the GeneCards database. Table S3: the common targets of ABM and NAFLD. Table S4: the 383 biological process terms. Table S5: the 40 cellular component terms. Table S6: the 63 molecular function terms. [file 4745042.f1.zip › 4745042.f1/Table S2.pdf]

Gene	Description	Category	Gifts	GC Id	Relevance score	GeneCards Link
PNPLA3	Patatin Like Phospholipase Domain Containing 3	Protein Coding	41	GC22P043923	102.4237137	https://www.genecards.org/cgi-bin/carddisp.pl?gene=PNPLA3
IL6	Interleukin 6	Protein Coding	50	GC07P022725	95.30979919	https://www.genecards.org/cgi-bin/carddisp.pl?gene=IL6
APOE	Apolipoprotein E	Protein Coding	51	GC19P044906	94.36561584	https://www.genecards.org/cgi-bin/carddisp.pl?gene=APOE
PRMT7	Protein Arginine Methyltransferase 7	Protein Coding	45	GC16P068363	93.61840057	https://www.genecards.org/cgi-bin/carddisp.pl?gene=PRMT7
MIR122	MicroRNA 122	RNA Gene	21	GC18P058451	93.14680481	https://www.genecards.org/cgi-bin/carddisp.pl?gene=MIR122
TNF	Tumor Necrosis Factor	Protein Coding	51	GC06P055202	92.23382568	https://www.genecards.org/cgi-bin/carddisp.pl?gene=TNF
TP53	Tumor Protein P53	Protein Coding	54	GC17M007661	90.97099304	https://www.genecards.org/cgi-bin/carddisp.pl?gene=TP53
PKD2	Polycystin 2, Transient Receptor Potential Cation Channel	Protein Coding	47	GC04P088007	89.09770966	https://www.genecards.org/cgi-bin/carddisp.pl?gene=PKD2
INS	Insulin	Protein Coding	48	GC11M002159	85.24944305	https://www.genecards.org/cgi-bin/carddisp.pl?gene=INS
HFE	Homeostatic Iron Regulator	Protein Coding	44	GC06P026087	80.59532928	https://www.genecards.org/cgi-bin/carddisp.pl?gene=HFE
ALB	Albumin	Protein Coding	50	GC04P073397	79.82528687	https://www.genecards.org/cgi-bin/carddisp.pl?gene=ALB
IL10	Interleukin 10	Protein Coding	48	GC01M206767	77.66014862	https://www.genecards.org/cgi-bin/carddisp.pl?gene=IL10
PPARG	Peroxisome Proliferator Activated Receptor Gamma	Protein Coding	53	GC03P012287	77.08204651	https://www.genecards.org/cgi-bin/carddisp.pl?gene=PPARG
HADHA	Hydroxyacyl-CoA Dehydrogenase Trifunctional Multienzyme Complex Subunit Alpha	Protein Coding	47	GC02M026190	76.25171661	https://www.genecards.org/cgi-bin/carddisp.pl?gene=HADHA
TGFB1	Transforming Growth Factor Beta 1	Protein Coding	52	GC19M041301	72.79130554	https://www.genecards.org/cgi-bin/carddisp.pl?gene=TGFB1
LMNA	Lamin A/C	Protein Coding	48	GC01P156082	71.62808228	https://www.genecards.org/cgi-bin/carddisp.pl?gene=LMNA
MARS1	Methionyl-TRNA Synthetase 1	Protein Coding	36	GC12P057476	71.13464355	https://www.genecards.org/cgi-bin/carddisp.pl?gene=MARS1
FABP1	Fatty Acid Binding Protein 1	Protein Coding	44	GC02M088122	67.62335968	https://www.genecards.org/cgi-bin/carddisp.pl?gene=FABP1
SMPD1	Sphingomyelin Phosphodiesterase 1	Protein Coding	48	GC11P006390	66.9227829	https://www.genecards.org/cgi-bin/carddisp.pl?gene=SMPD1
SLC17A5	Solute Carrier Family 17 Member 5	Protein Coding	45	GC06M073593	66.39536285	https://www.genecards.org/cgi-bin/carddisp.pl?gene=SLC17A5
SERPINA1	Serpin Family A Member 1	Protein Coding	50	GC14M094376	66.35801697	https://www.genecards.org/cgi-bin/carddisp.pl?gene=SERPINA1
FASN	Fatty Acid Synthase	Protein Coding	50	GC17M082078	66.21472168	https://www.genecards.org/cgi-bin/carddisp.pl?gene=FASN
MIR21	MicroRNA 21	RNA Gene	24	GC17P059841	66.13240051	https://www.genecards.org/cgi-bin/carddisp.pl?gene=MIR21
ATP7B	ATPase Copper Transporting Beta	Protein Coding	48	GC13M051930	65.01322937	https://www.genecards.org/cgi-bin/carddisp.pl?gene=ATP7B
GPT	Glutamic--Pyruvic Transaminase	Protein Coding	43	GC08P144502	64.76797485	https://www.genecards.org/cgi-bin/carddisp.pl?gene=GPT
FAS	Fas Cell Surface Death Receptor	Protein Coding	51	GC10P088969	64.5565033	https://www.genecards.org/cgi-bin/carddisp.pl?gene=FAS
ABCB11	ATP Binding Cassette Subfamily B Member 11	Protein Coding	47	GC02M168922	63.66309357	https://www.genecards.org/cgi-bin/carddisp.pl?gene=ABCB11
GANAB	Glucosidase II Alpha Subunit	Protein Coding	45	GC11M069251	63.61607361	https://www.genecards.org/cgi-bin/carddisp.pl?gene=GANAB
APOA1	Apolipoprotein A1	Protein Coding	49	GC11M116835	62.34758759	https://www.genecards.org/cgi-bin/carddisp.pl?gene=APOA1
CTNNB1	Catenin Beta 1	Protein Coding	54	GC03P041236	62.09061432	https://www.genecards.org/cgi-bin/carddisp.pl?gene=CTNNB1
PPARA	Peroxisome Proliferator Activated Receptor Alpha	Protein Coding	47	GC22P046150	61.89193344	https://www.genecards.org/cgi-bin/carddisp.pl?gene=PPARA
IL1B	Interleukin 1 Beta	Protein Coding	49	GC02M112829	61.4563179	https://www.genecards.org/cgi-bin/carddisp.pl?gene=IL1B
MIR34A	MicroRNA 34a	RNA Gene	22	GC01M009151	61.06716156	https://www.genecards.org/cgi-bin/carddisp.pl?gene=MIR34A
APOB	Apolipoprotein B	Protein Coding	46	GC02M020956	61.02858734	https://www.genecards.org/cgi-bin/carddisp.pl?gene=APOB
NAFLD1	Fatty Liver Disease 1, Susceptiblity To	Genetic Locus	1	GC22U901005	61.00867462	https://www.genecards.org/cgi-bin/carddisp.pl?gene=NAFLD1
ADIPOQ	Adiponectin, C1Q And Collagen Domain Containing	Protein Coding	45	GC03P186842	60.28987122	https://www.genecards.org/cgi-bin/carddisp.pl?gene=ADIPOQ
NOD2	Nucleotide Binding Oligomerization Domain Containing 2	Protein Coding	48	GC16P050693	60.10681534	https://www.genecards.org/cgi-bin/carddisp.pl?gene=NOD2
MIR10B	MicroRNA 10b	RNA Gene	21	GC02P176150	59.71246338	https://www.genecards.org/cgi-bin/carddisp.pl?gene=MIR10B
LIPA	Lipase A, Lysosomal Acid Type	Protein Coding	48	GC10M089213	59.69533539	https://www.genecards.org/cgi-bin/carddisp.pl?gene=LIPA
PIK3CA	Phosphatidylinositol-4,5-Bisphosphate 3-Kinase Catalytic Subunit Alpha	Protein Coding	53	GC03P179148	59.36377335	https://www.genecards.org/cgi-bin/carddisp.pl?gene=PIK3CA
CPT2	Carnitine Palmitoyltransferase 2	Protein Coding	50	GC01P053196	59.22840118	https://www.genecards.org/cgi-bin/carddisp.pl?gene=CPT2
TLR4	Toll Like Receptor 4	Protein Coding	51	GC09P117704	59.15758514	https://www.genecards.org/cgi-bin/carddisp.pl?gene=TLR4
SQSTM1	Sequestosome 1	Protein Coding	49	GC05P179806	58.94549561	https://www.genecards.org/cgi-bin/carddisp.pl?gene=SQSTM1
CRP	C-Reactive Protein	Protein Coding	47	GC01M159719	58.70598602	https://www.genecards.org/cgi-bin/carddisp.pl?gene=CRP
NR1H4	Nuclear Receptor Subfamily 1 Group H Member 4	Protein Coding	49	GC12P100473	58.61371613	https://www.genecards.org/cgi-bin/carddisp.pl?gene=NR1H4
MFN2	Mitofusin 2	Protein Coding	48	GC01P011980	58.31835938	https://www.genecards.org/cgi-bin/carddisp.pl?gene=MFN2
CPT1A	Carnitine Palmitoyltransferase 1A	Protein Coding	50	GC11M068754	58.00518799	https://www.genecards.org/cgi-bin/carddisp.pl?gene=CPT1A
ABCA1	ATP Binding Cassette Subfamily A Member 1	Protein Coding	48	GC09M104781	57.74860001	https://www.genecards.org/cgi-bin/carddisp.pl?gene=ABCA1
APP	Amyloid Beta Precursor Protein	Protein Coding	52	GC21M025880	57.08755493	https://www.genecards.org/cgi-bin/carddisp.pl?gene=APP
MIR17	MicroRNA 17	RNA Gene	21	GC13P091350	56.95721436	https://www.genecards.org/cgi-bin/carddisp.pl?gene=MIR17
G6PC1	Glucose-6-Phosphatase Catalytic Subunit 1	Protein Coding	36	GC17P044746	56.77759171	https://www.genecards.org/cgi-bin/carddisp.pl?gene=G6PC1
KRT18	Keratin 18	Protein Coding	49	GC12P052948	56.36305237	https://www.genecards.org/cgi-bin/carddisp.pl?gene=KRT18
MIR155	MicroRNA 155	RNA Gene	19	GC21P025560	55.06369781	https://www.genecards.org/cgi-bin/carddisp.pl?gene=MIR155
CXCL8	C-X-C Motif Chemokine Ligand 8	Protein Coding	42	GC04P073740	54.85639954	https://www.genecards.org/cgi-bin/carddisp.pl?gene=CXCL8
AKT1	AKT Serine/Threonine Kinase 1	Protein Coding	54	GC14M104769	54.75885391	https://www.genecards.org/cgi-bin/carddisp.pl?gene=AKT1
LPL	Lipoprotein Lipase	Protein Coding	50	GC08P019901	54.21808624	https://www.genecards.org/cgi-bin/carddisp.pl?gene=LPL
PTEN	Phosphatase And Tensin Homolog	Protein Coding	53	GC10P087863	53.96323013	https://www.genecards.org/cgi-bin/carddisp.pl?gene=PTEN
CD36	CD36 Molecule	Protein Coding	49	GC07P080369	53.85723114	https://www.genecards.org/cgi-bin/carddisp.pl?gene=CD36
CTLA4	Cytotoxic T-Lymphocyte Associated Protein 4	Protein Coding	46	GC02P203867	53.4139328	https://www.genecards.org/cgi-bin/carddisp.pl?gene=CTLA4
GGT1	Gamma-Glutamyltransferase 1	Protein Coding	48	GC22P024583	52.50072098	https://www.genecards.org/cgi-bin/carddisp.pl?gene=GGT1
HNF4A	Hepatocyte Nuclear Factor 4 Alpha	Protein Coding	51	GC20P044355	52.43869781	https://www.genecards.org/cgi-bin/carddisp.pl?gene=HNF4A
CYP2E1	Cytochrome P450 Family 2 Subfamily E Member 1	Protein Coding	46	GC10P133520	52.40969467	https://www.genecards.org/cgi-bin/carddisp.pl?gene=CYP2E1
LEP	Leptin	Protein Coding	48	GC07P128241	52.03404236	https://www.genecards.org/cgi-bin/carddisp.pl?gene=LEP
VWF	Von Willebrand Factor	Protein Coding	49	GC12M005917	51.99846649	https://www.genecards.org/cgi-bin/carddisp.pl?gene=VWF
VCP	Valosin Containing Protein	Protein Coding	50	GC09M035056	51.8118248	https://www.genecards.org/cgi-bin/carddisp.pl?gene=VCP
FABP4	Fatty Acid Binding Protein 4	Protein Coding	45	GC08M081478	51.40408707	https://www.genecards.org/cgi-bin/carddisp.pl?gene=FABP4
SREBF1	Sterol Regulatory Element Binding Transcription Factor 1	Protein Coding	46	GC17M017810	51.28795624	https://www.genecards.org/cgi-bin/carddisp.pl?gene=SREBF1
F2	Coagulation Factor II, Thrombin	Protein Coding	49	GC11P046720	51.17159271	https://www.genecards.org/cgi-bin/carddisp.pl?gene=F2
MTTP	Microsomal Triglyceride Transfer Protein	Protein Coding	45	GC04P099563	50.73419189	https://www.genecards.org/cgi-bin/carddisp.pl?gene=MTTP
INSR	Insulin Receptor	Protein Coding	54	GC19M007112	49.7062912	https://www.genecards.org/cgi-bin/carddisp.pl?gene=INSR
IFNG	Interferon Gamma	Protein Coding	48	GC12M068154	49.25480652	https://www.genecards.org/cgi-bin/carddisp.pl?gene=IFNG
NR1H3	Nuclear Receptor Subfamily 1 Group H Member 3	Protein Coding	48	GC11P047248	49.17764664	https://www.genecards.org/cgi-bin/carddisp.pl?gene=NR1H3
CFTR	CF Transmembrane Conductance	Protein Coding	52	GC07P117287	49.02300262	https://www.genecards.org/cgi-bin/carddisp.pl?gene=CFTR
ACE	Angiotensin I Converting Enzyme	Protein Coding	50	GC17P063477	48.6252594	https://www.genecards.org/cgi-bin/carddisp.pl?gene=ACE
TERT	Telomerase Reverse Transcriptase	Protein Coding	52	GC05M001253	48.496418	https://www.genecards.org/cgi-bin/carddisp.pl?gene=TERT
FABP2	Fatty Acid Binding Protein 2	Protein Coding	43	GC04M119317	48.23287582	https://www.genecards.org/cgi-bin/carddisp.pl?gene=FABP2
MIR146A	MicroRNA 146a	RNA Gene	22	GC05P160485	47.88103485	https://www.genecards.org/cgi-bin/carddisp.pl?gene=MIR146A
ACOX1	Acyl-CoA Oxidase 1	Protein Coding	47	GC17M075941	47.87750244	https://www.genecards.org/cgi-bin/carddisp.pl?gene=ACOX1
ABCB4	ATP Binding Cassette Subfamily B Member 4	Protein Coding	47	GC07M087401	47.29715347	https://www.genecards.org/cgi-bin/carddisp.pl?gene=ABCB4
SERPINE1	Serpin Family E Member 1	Protein Coding	51	GC07P101127	46.52587891	https://www.genecards.org/cgi-bin/carddisp.pl?gene=SERPINE1
EGFR	Epidermal Growth Factor Receptor	Protein Coding	54	GC07P055019	46.42074203	https://www.genecards.org/cgi-bin/carddisp.pl?gene=EGFR
CASP8	Caspase 8	Protein Coding	52	GC02P201233	46.29785156	https://www.genecards.org/cgi-bin/carddisp.pl?gene=CASP8
IGF1	Insulin Like Growth Factor 1	Protein Coding	50	GC12M102395	46.25844574	https://www.genecards.org/cgi-bin/carddisp.pl?gene=IGF1
CYBB	Cytochrome B-245 Beta Chain	Protein Coding	47	GC0XP037780	46.05702591	https://www.genecards.org/cgi-bin/carddisp.pl?gene=CYBB
STAT3	Signal Transducer And Activator Of Transcription 3	Protein Coding	53	GC17M042313	45.96729279	https://www.genecards.org/cgi-bin/carddisp.pl?gene=STAT3
RBP4	Retinol Binding Protein 4	Protein Coding	45	GC10M093591	45.69139862	https://www.genecards.org/cgi-bin/carddisp.pl?gene=RBP4
HMOX1	Heme Oxygenase 1	Protein Coding	53	GC22P035380	45.51039124	https://www.genecards.org/cgi-bin/carddisp.pl?gene=HMOX1
HLA-DQB1	Major Histocompatibility Complex, Class II, DQ Beta 1	Protein Coding	43	GC06M046939	45.46877289	https://www.genecards.org/cgi-bin/carddisp.pl?gene=HLA-DQB1

HBB	Hemoglobin Subunit Beta	Protein Coding	46	GC11M005434	44.67763901	https://www.genecards.org/cgi-bin/carddisp.pl?gene=HBB
TTR	Transthyretin	Protein Coding	50	GC18P031557	44.22271729	https://www.genecards.org/cgi-bin/carddisp.pl?gene=TTR
IRS1	Insulin Receptor Substrate 1	Protein Coding	48	GC02M226731	43.72430801	https://www.genecards.org/cgi-bin/carddisp.pl?gene=IRS1
EGF	Epidermal Growth Factor	Protein Coding	51	GC04P109912	43.58280182	https://www.genecards.org/cgi-bin/carddisp.pl?gene=EGF
SLC25A13	Solute Carrier Family 25 Member 13	Protein Coding	47	GC07M096120	43.45845413	https://www.genecards.org/cgi-bin/carddisp.pl?gene=SLC25A13
IL1A	Interleukin 1 Alpha	Protein Coding	44	GC02M112773	43.39876175	https://www.genecards.org/cgi-bin/carddisp.pl?gene=IL1A
AFP	Alpha Fetoprotein	Protein Coding	46	GC04P073431	43.34745026	https://www.genecards.org/cgi-bin/carddisp.pl?gene=AFP
TLR2	Toll Like Receptor 2	Protein Coding	52	GC04P153684	43.21223068	https://www.genecards.org/cgi-bin/carddisp.pl?gene=TLR2
KRT8	Keratin 8	Protein Coding	48	GC12M052897	43.20010376	https://www.genecards.org/cgi-bin/carddisp.pl?gene=KRT8
ABHD5	Abhydrolase Domain Containing 5, Lysophosphatidic Acid Acyltransferase	Protein Coding	45	GC03P043707	42.95199966	https://www.genecards.org/cgi-bin/carddisp.pl?gene=ABHD5
MIR27A	MicroRNA 27a	RNA Gene	22	GC19M014109	42.90045929	https://www.genecards.org/cgi-bin/carddisp.pl?gene=MIR27A
LEPR	Leptin Receptor	Protein Coding	50	GC01P065421	42.75074005	https://www.genecards.org/cgi-bin/carddisp.pl?gene=LEPR
CASP3	Caspase 3	Protein Coding	51	GC04M184627	42.58610153	https://www.genecards.org/cgi-bin/carddisp.pl?gene=CASP3
CCL2	C-C Motif Chemokine Ligand 2	Protein Coding	49	GC17P034255	42.4992485	https://www.genecards.org/cgi-bin/carddisp.pl?gene=CCL2
SOD1	Superoxide Dismutase 1	Protein Coding	52	GC21P031659	42.46434402	https://www.genecards.org/cgi-bin/carddisp.pl?gene=SOD1
MTHFR	Methylenetetrahydrofolate Reductase	Protein Coding	49	GC01M011785	42.33821869	https://www.genecards.org/cgi-bin/carddisp.pl?gene=MTHFR
BSCL2	BSCL2 Lipid Droplet Biogenesis Associated, Seipin	Protein Coding	43	GC11M069260	42.32678604	https://www.genecards.org/cgi-bin/carddisp.pl?gene=BSCL2
PPARGC1A	PPARG Coactivator 1 Alpha	Protein Coding	47	GC04M023755	42.03114319	https://www.genecards.org/cgi-bin/carddisp.pl?gene=PPARGC1A
MIR29A	MicroRNA 29a	RNA Gene	22	GC07M130876	41.87608719	https://www.genecards.org/cgi-bin/carddisp.pl?gene=MIR29A
VEGFA	Vascular Endothelial Growth Factor A	Protein Coding	49	GC06P043770	41.61352921	https://www.genecards.org/cgi-bin/carddisp.pl?gene=VEGFA
MIR200B	MicroRNA 200b	RNA Gene	20	GC01P001167	41.41399384	https://www.genecards.org/cgi-bin/carddisp.pl?gene=MIR200B
MIR203A	MicroRNA 203a	RNA Gene	19	GC14P106054	41.18896484	https://www.genecards.org/cgi-bin/carddisp.pl?gene=MIR203A
RETN	Resistin	Protein Coding	44	GC19P007669	40.77745056	https://www.genecards.org/cgi-bin/carddisp.pl?gene=RETN
PON1	Paraoxonase 1	Protein Coding	46	GC07M095297	40.4878006	https://www.genecards.org/cgi-bin/carddisp.pl?gene=PON1
JAG1	Jagged Canonical Notch Ligand 1	Protein Coding	51	GC20M010637	40.26721573	https://www.genecards.org/cgi-bin/carddisp.pl?gene=JAG1
MMP9	Matrix Metallopeptidase 9	Protein Coding	54	GC20P046008	40.25731659	https://www.genecards.org/cgi-bin/carddisp.pl?gene=MMP9
GARS1	Glycyl-TRNA Synthetase 1	Protein Coding	37	GC07P030580	40.09081268	https://www.genecards.org/cgi-bin/carddisp.pl?gene=GARS1
YARS1	Tyrosyl-TRNA Synthetase 1	Protein Coding	37	GC01M032776	40.0786438	https://www.genecards.org/cgi-bin/carddisp.pl?gene=YARS1
SMAD4	SMAD Family Member 4	Protein Coding	51	GC18P051028	40.06649017	https://www.genecards.org/cgi-bin/carddisp.pl?gene=SMAD4
CYP3A4	Cytochrome P450 Family 3 Subfamily A Member 4	Protein Coding	51	GC07M099759	40.04926682	https://www.genecards.org/cgi-bin/carddisp.pl?gene=CYP3A4
FADS2	Fatty Acid Desaturase 2	Protein Coding	45	GC11P061792	39.96272278	https://www.genecards.org/cgi-bin/carddisp.pl?gene=FADS2
LDLR	Low Density Lipoprotein Receptor	Protein Coding	50	GC19P011091	39.88188553	https://www.genecards.org/cgi-bin/carddisp.pl?gene=LDLR
FABP3	Fatty Acid Binding Protein 3	Protein Coding	44	GC01M031365	39.87561798	https://www.genecards.org/cgi-bin/carddisp.pl?gene=FABP3
HSPB1	Heat Shock Protein Family B (Small) Member 1	Protein Coding	52	GC07P076302	39.70830917	https://www.genecards.org/cgi-bin/carddisp.pl?gene=HSPB1
UCP2	Uncoupling Protein 2	Protein Coding	46	GC11M073974	39.67284775	https://www.genecards.org/cgi-bin/carddisp.pl?gene=UCP2
MMP1	Matrix Metallopeptidase 1	Protein Coding	52	GC11M102810	39.0076828	https://www.genecards.org/cgi-bin/carddisp.pl?gene=MMP1
MIR22	MicroRNA 22	RNA Gene	20	GC17M001713	38.98328018	https://www.genecards.org/cgi-bin/carddisp.pl?gene=MIR22
NOTCH1	Notch Receptor 1	Protein Coding	51	GC09M136705	38.94213867	https://www.genecards.org/cgi-bin/carddisp.pl?gene=NOTCH1
IL18	Interleukin 18	Protein Coding	44	GC11M112143	38.8327179	https://www.genecards.org/cgi-bin/carddisp.pl?gene=IL18
SOD2	Superoxide Dismutase 2	Protein Coding	51	GC06M159669	38.7767334	https://www.genecards.org/cgi-bin/carddisp.pl?gene=SOD2
MPO	Myeloperoxidase	Protein Coding	51	GC17M058269	38.43812561	https://www.genecards.org/cgi-bin/carddisp.pl?gene=MPO
SCD	Stearoyl-CoA Desaturase	Protein Coding	49	GC10P100347	38.4039917	https://www.genecards.org/cgi-bin/carddisp.pl?gene=SCD
CYBA	Cytochrome B-245 Alpha Chain	Protein Coding	48	GC16M088643	38.31346512	https://www.genecards.org/cgi-bin/carddisp.pl?gene=CYBA
CFH	Complement Factor H	Protein Coding	46	GC01P196621	37.9663887	https://www.genecards.org/cgi-bin/carddisp.pl?gene=CFH
MLXIPL	MLX Interacting Protein Like	Protein Coding	43	GC07M073593	37.83913422	https://www.genecards.org/cgi-bin/carddisp.pl?gene=MLXIPL
CAV1	Caveolin 1	Protein Coding	48	GC07P116524	37.80889511	https://www.genecards.org/cgi-bin/carddisp.pl?gene=CAV1
MAPK8	Mitogen-Activated Protein Kinase 8	Protein Coding	51	GC10P048306	37.78774261	https://www.genecards.org/cgi-bin/carddisp.pl?gene=MAPK8
PNPLA2	Patatin Like Phospholipase Domain Containing 2	Protein Coding	45	GC11P000994	37.62009811	https://www.genecards.org/cgi-bin/carddisp.pl?gene=PNPLA2
TNFRSF1A	TNF Receptor Superfamily Member 1A	Protein Coding	50	GC12M006328	37.56293488	https://www.genecards.org/cgi-bin/carddisp.pl?gene=TNFRSF1A
CP	Ceruloplasmin	Protein Coding	48	GC03M149162	37.3901062	https://www.genecards.org/cgi-bin/carddisp.pl?gene=CP
SERPINA3	Serpin Family A Member 3	Protein Coding	44	GC14P094612	37.31189346	https://www.genecards.org/cgi-bin/carddisp.pl?gene=SERPINA3
SPP1	Secreted Phosphoprotein 1	Protein Coding	47	GC04P087975	37.20774841	https://www.genecards.org/cgi-bin/carddisp.pl?gene=SPP1
MIR20A	MicroRNA 20a	RNA Gene	19	GC13P091444	37.10332489	https://www.genecards.org/cgi-bin/carddisp.pl?gene=MIR20A
BRCA2	BRCA2 DNA Repair Associated	Protein Coding	50	GC13P032315	37.04915619	https://www.genecards.org/cgi-bin/carddisp.pl?gene=BRCA2
UGT1A1	UDP Glucuronosyltransferase Family 1 Member A1	Protein Coding	49	GC02P233760	36.9387207	https://www.genecards.org/cgi-bin/carddisp.pl?gene=UGT1A1
MTOR	Mechanistic Target Of Rapamycin Kinase	Protein Coding	54	GC01M011106	36.73412704	https://www.genecards.org/cgi-bin/carddisp.pl?gene=MTOR
RELA	RELA Proto-Oncogene, NF-KB Subunit	Protein Coding	51	GC11M065653	36.6779213	https://www.genecards.org/cgi-bin/carddisp.pl?gene=RELA
SIRT1	Sirtuin 1	Protein Coding	50	GC10P067884	36.48433685	https://www.genecards.org/cgi-bin/carddisp.pl?gene=SIRT1
APOC3	Apolipoprotein C3	Protein Coding	44	GC11P116829	36.37243652	https://www.genecards.org/cgi-bin/carddisp.pl?gene=APOC3
DNAJB11	DnaJ Heat Shock Protein Family (Hsp40) Member B11	Protein Coding	41	GC03P186567	36.25784302	https://www.genecards.org/cgi-bin/carddisp.pl?gene=DNAJB11
ACACA	Acetyl-CoA Carboxylase Alpha	Protein Coding	50	GC17M037084	36.21570587	https://www.genecards.org/cgi-bin/carddisp.pl?gene=ACACA
ELOVL6	ELOVL Fatty Acid Elongase 6	Protein Coding	41	GC04M110045	35.96213531	https://www.genecards.org/cgi-bin/carddisp.pl?gene=ELOVL6
PRKAG2	Protein Kinase AMP-Activated Non-Catalytic Subunit Gamma 2	Protein Coding	51	GC07M151556	35.65856171	https://www.genecards.org/cgi-bin/carddisp.pl?gene=PRKAG2
ABCC2	ATP Binding Cassette Subfamily C Member 2	Protein Coding	48	GC10P099782	35.48709869	https://www.genecards.org/cgi-bin/carddisp.pl?gene=ABCC2
LIPC	Lipase C, Hepatic Type	Protein Coding	46	GC15P058410	35.48361969	https://www.genecards.org/cgi-bin/carddisp.pl?gene=LIPC
ICAM1	Intercellular Adhesion Molecule 1	Protein Coding	51	GC19P010270	35.46846771	https://www.genecards.org/cgi-bin/carddisp.pl?gene=ICAM1
CCND1	Cyclin D1	Protein Coding	52	GC11P069641	35.30378723	https://www.genecards.org/cgi-bin/carddisp.pl?gene=CCND1
MIR126	MicroRNA 126	RNA Gene	22	GC09P136670	35.23448944	https://www.genecards.org/cgi-bin/carddisp.pl?gene=MIR126
HAMP	Hepcidin Antimicrobial Peptide	Protein Coding	44	GC19P040344	35.09099579	https://www.genecards.org/cgi-bin/carddisp.pl?gene=HAMP
HMGCR	3-Hydroxy-3-Methylglutaryl-CoA	Protein Coding	47	GC05P075336	35.07630539	https://www.genecards.org/cgi-bin/carddisp.pl?gene=HMGCR
TF	Transferrin	Protein Coding	50	GC03P133666	34.86120605	https://www.genecards.org/cgi-bin/carddisp.pl?gene=TF
HGF	Hepatocyte Growth Factor	Protein Coding	52	GC07M081699	34.77748108	https://www.genecards.org/cgi-bin/carddisp.pl?gene=HGF
NFKB1	Nuclear Factor Kappa B Subunit 1	Protein Coding	52	GC04P102501	34.63681793	https://www.genecards.org/cgi-bin/carddisp.pl?gene=NFKB1
ANO5	Anoctamin 5	Protein Coding	38	GC11P021799	34.59410858	https://www.genecards.org/cgi-bin/carddisp.pl?gene=ANO5
LCAT	Lecithin-Cholesterol Acyltransferase	Protein Coding	47	GC16M067939	34.5559082	https://www.genecards.org/cgi-bin/carddisp.pl?gene=LCAT
CAT	Catalase	Protein Coding	50	GC11P034460	34.48881531	https://www.genecards.org/cgi-bin/carddisp.pl?gene=CAT
MIR221	MicroRNA 221	RNA Gene	20	GC0XM045746	34.46895599	https://www.genecards.org/cgi-bin/carddisp.pl?gene=MIR221
PTGS2	Prostaglandin-Endoperoxide Synthase 2	Protein Coding	49	GC01M186640	34.3535881	https://www.genecards.org/cgi-bin/carddisp.pl?gene=PTGS2
TIMP1	TIMP Metallopeptidase Inhibitor 1	Protein Coding	45	GC0XP047583	34.31763458	https://www.genecards.org/cgi-bin/carddisp.pl?gene=TIMP1
IRS2	Insulin Receptor Substrate 2	Protein Coding	45	GC13M109752	34.14591217	https://www.genecards.org/cgi-bin/carddisp.pl?gene=IRS2
ESR1	Estrogen Receptor 1	Protein Coding	53	GC06P151656	34.093647	https://www.genecards.org/cgi-bin/carddisp.pl?gene=ESR1
FASLG	Fas Ligand	Protein Coding	48	GC01P172628	34.00541687	https://www.genecards.org/cgi-bin/carddisp.pl?gene=FASLG
IL1RN	Interleukin 1 Receptor Antagonist	Protein Coding	48	GC02P116694	33.9354744	https://www.genecards.org/cgi-bin/carddisp.pl?gene=IL1RN
XBP1	X-Box Binding Protein 1	Protein Coding	46	GC22M028794	33.89216995	https://www.genecards.org/cgi-bin/carddisp.pl?gene=XBP1
PTPN11	Protein Tyrosine Phosphatase Non-Receptor Type 11	Protein Coding	54	GC12P112418	33.66088867	https://www.genecards.org/cgi-bin/carddisp.pl?gene=PTPN11
MIR132	MicroRNA 132	RNA Gene	21	GC17M002049	33.63765335	https://www.genecards.org/cgi-bin/carddisp.pl?gene=MIR132
MIR222	MicroRNA 222	RNA Gene	21	GC0XM045747	33.41287994	https://www.genecards.org/cgi-bin/carddisp.pl?gene=MIR222
ADIPOR1	Adiponectin Receptor 1	Protein Coding	45	GC01M202940	33.19025803	https://www.genecards.org/cgi-bin/carddisp.pl?gene=ADIPOR1
CDKN2A	Cyclin Dependent Kinase Inhibitor 2A	Protein Coding	52	GC09M021967	33.02046204	https://www.genecards.org/cgi-bin/carddisp.pl?gene=CDKN2A
DDIT3	DNA Damage Inducible Transcript 3	Protein Coding	46	GC12M057516	32.84270477	https://www.genecards.org/cgi-bin/carddisp.pl?gene=DDIT3
ADIPOR2	Adiponectin Receptor 2	Protein Coding	43	GC12P001670	32.60053635	https://www.genecards.org/cgi-bin/carddisp.pl?gene=ADIPOR2
LITAF	Lipopolysaccharide Induced TNF Factor	Protein Coding	45	GC16M011547	32.4716568	https://www.genecards.org/cgi-bin/carddisp.pl?gene=LITAF
CYCS	Cytochrome C, Somatic	Protein Coding	49	GC07M025118	32.37697983	https://www.genecards.org/cgi-bin/carddisp.pl?gene=CYCS
SDHB	Succinate Dehydrogenase Complex Iron Sulfur Subunit B	Protein Coding	48	GC01M017238	32.02085114	https://www.genecards.org/cgi-bin/carddisp.pl?gene=SDHB
STAT1	Signal Transducer And Activator Of Transcription 1	Protein Coding	54	GC02M190908	31.95772362	https://www.genecards.org/cgi-bin/carddisp.pl?gene=STAT1
MT-CYB	Mitochondrially Encoded Cytochrome B	Protein Coding	32	GCMTP014749	31.85387611	https://www.genecards.org/cgi-bin/carddisp.pl?gene=MT-CYB

BDNF	Brain Derived Neurotrophic Factor	Protein Coding	48	GC11M027654	31.83507347	https://www.genecards.org/cgi-bin/carddisp.pl?gene=BDNF
HP	Haptoglobin	Protein Coding	45	GC16P072089	31.81941605	https://www.genecards.org/cgi-bin/carddisp.pl?gene=HP
MIR140	MicroRNA 140	RNA Gene	22	GC16P069934	31.75845909	https://www.genecards.org/cgi-bin/carddisp.pl?gene=MIR140
AGT	Angiotensinogen	Protein Coding	50	GC01M230702	31.73010635	https://www.genecards.org/cgi-bin/carddisp.pl?gene=AGT
TM6SF2	Transmembrane 6 Superfamily Member 2	Protein Coding	29	GC19M019264	31.40005493	https://www.genecards.org/cgi-bin/carddisp.pl?gene=TM6SF2
MMP2	Matrix Metallopeptidase 2	Protein Coding	54	GC16P055390	31.33286285	https://www.genecards.org/cgi-bin/carddisp.pl?gene=MMP2
ENPP1	Ectonucleotide Pyrophosphatase/Phosphodiesterase 1	Protein Coding	48	GC06P131808	31.26300621	https://www.genecards.org/cgi-bin/carddisp.pl?gene=ENPP1
TGFBR2	Transforming Growth Factor Beta Receptor 2	Protein Coding	51	GC03P030623	31.11475754	https://www.genecards.org/cgi-bin/carddisp.pl?gene=TGFBR2
FOS	Fos Proto-Oncogene, AP-1 Transcription Factor Subunit	Protein Coding	51	GC14P075278	31.08187485	https://www.genecards.org/cgi-bin/carddisp.pl?gene=FOS
NOS2	Nitric Oxide Synthase 2	Protein Coding	50	GC17M027756	31.06771851	https://www.genecards.org/cgi-bin/carddisp.pl?gene=NOS2
MIR181A1	MicroRNA 181a-1	RNA Gene	19	GC01M198860	31.06432343	https://www.genecards.org/cgi-bin/carddisp.pl?gene=MIR181A1
JUN	Jun Proto-Oncogene, AP-1 Transcription Factor Subunit	Protein Coding	50	GC01M058780	31.01990891	https://www.genecards.org/cgi-bin/carddisp.pl?gene=JUN
MYC	MYC Proto-Oncogene, BHLH Transcription Factor	Protein Coding	51	GC08P127735	30.94736099	https://www.genecards.org/cgi-bin/carddisp.pl?gene=MYC
FABP12	Fatty Acid Binding Protein 12	Protein Coding	32	GC08M081524	30.87104034	https://www.genecards.org/cgi-bin/carddisp.pl?gene=FABP12
CYP1A1	Cytochrome P450 Family 1 Subfamily A Member 1	Protein Coding	48	GC15M074719	30.83807373	https://www.genecards.org/cgi-bin/carddisp.pl?gene=CYP1A1
SLC27A2	Solute Carrier Family 27 Member 2	Protein Coding	46	GC15P050182	30.83671761	https://www.genecards.org/cgi-bin/carddisp.pl?gene=SLC27A2
TARDBP	TAR DNA Binding Protein	Protein Coding	47	GC01P011013	30.79296494	https://www.genecards.org/cgi-bin/carddisp.pl?gene=TARDBP
IL17A	Interleukin 17A	Protein Coding	43	GC06P052186	30.7634449	https://www.genecards.org/cgi-bin/carddisp.pl?gene=IL17A
SDHD	Succinate Dehydrogenase Complex Subunit D	Protein Coding	45	GC11P112087	30.75510979	https://www.genecards.org/cgi-bin/carddisp.pl?gene=SDHD
SLC2A1	Solute Carrier Family 2 Member 1	Protein Coding	53	GC01M042925	30.74088669	https://www.genecards.org/cgi-bin/carddisp.pl?gene=SLC2A1
NR1H2	Nuclear Receptor Subfamily 1 Group H Member 2	Protein Coding	49	GC19P050329	30.56098175	https://www.genecards.org/cgi-bin/carddisp.pl?gene=NR1H2
GCKR	Glucokinase Regulator	Protein Coding	42	GC02P027496	30.43237877	https://www.genecards.org/cgi-bin/carddisp.pl?gene=GCKR
GFER	Growth Factor, Augmenter Of Liver Regeneration	Protein Coding	45	GC16P001984	30.36795425	https://www.genecards.org/cgi-bin/carddisp.pl?gene=GFER
MT-CO1	Mitochondrially Encoded Cytochrome C Oxidase I	Protein Coding	34	GCMTP005906	30.12521744	https://www.genecards.org/cgi-bin/carddisp.pl?gene=MT-CO1
TNFRSF11B	TNF Receptor Superfamily Member 11b	Protein Coding	48	GC08M118923	30.02269554	https://www.genecards.org/cgi-bin/carddisp.pl?gene=TNFRSF11B
PEMT	Phosphatidylethanolamine N-Methyltransferase	Protein Coding	41	GC17M017506	29.98512268	https://www.genecards.org/cgi-bin/carddisp.pl?gene=PEMT
COL1A1	Collagen Type I Alpha 1 Chain	Protein Coding	51	GC17M050183	29.84389877	https://www.genecards.org/cgi-bin/carddisp.pl?gene=COL1A1
MIR107	MicroRNA 107	RNA Gene	19	GC10M089600	29.81664085	https://www.genecards.org/cgi-bin/carddisp.pl?gene=MIR107
SLC40A1	Solute Carrier Family 40 Member 1	Protein Coding	47	GC02M189560	29.70161438	https://www.genecards.org/cgi-bin/carddisp.pl?gene=SLC40A1
CEBPA	CCAAT Enhancer Binding Protein Alpha	Protein Coding	48	GC19M033299	29.64617348	https://www.genecards.org/cgi-bin/carddisp.pl?gene=CEBPA
CETP	Cholesteryl Ester Transfer Protein	Protein Coding	47	GC16P056961	29.60931015	https://www.genecards.org/cgi-bin/carddisp.pl?gene=CETP
NDUFS1	NADH:Ubiquinone Oxidoreductase Core Subunit S1	Protein Coding	47	GC02M206114	29.59335709	https://www.genecards.org/cgi-bin/carddisp.pl?gene=NDUFS1
PLAU	Plasminogen Activator, Urokinase	Protein Coding	52	GC10P073909	29.57400703	https://www.genecards.org/cgi-bin/carddisp.pl?gene=PLAU
CYP1A2	Cytochrome P450 Family 1 Subfamily A Member 2	Protein Coding	46	GC15P074748	29.37054825	https://www.genecards.org/cgi-bin/carddisp.pl?gene=CYP1A2
DGAT1	Diacylglycerol O-Acyltransferase 1	Protein Coding	47	GC08M144316	29.37005615	https://www.genecards.org/cgi-bin/carddisp.pl?gene=DGAT1
MIR29C	MicroRNA 29c	RNA Gene	18	GC01M207802	29.35494232	https://www.genecards.org/cgi-bin/carddisp.pl?gene=MIR29C
GSTP1	Glutathione S-Transferase Pi 1	Protein Coding	51	GC11P067583	29.33395195	https://www.genecards.org/cgi-bin/carddisp.pl?gene=GSTP1
SLC27A4	Solute Carrier Family 27 Member 4	Protein Coding	47	GC09P128340	29.23885918	https://www.genecards.org/cgi-bin/carddisp.pl?gene=SLC27A4
IFNA1	Interferon Alpha 1	Protein Coding	40	GC09P021522	29.23606491	https://www.genecards.org/cgi-bin/carddisp.pl?gene=IFNA1
AKT2	AKT Serine/Threonine Kinase 2	Protein Coding	54	GC19M040230	29.22427368	https://www.genecards.org/cgi-bin/carddisp.pl?gene=AKT2
GSTM1	Glutathione S-Transferase Mu 1	Protein Coding	43	GC01P109687	29.21365356	https://www.genecards.org/cgi-bin/carddisp.pl?gene=GSTM1
GCG	Glucagon	Protein Coding	41	GC02M162142	29.18795395	https://www.genecards.org/cgi-bin/carddisp.pl?gene=GCG
GRN	Granulin Precursor	Protein Coding	47	GC17P044345	29.08464813	https://www.genecards.org/cgi-bin/carddisp.pl?gene=GRN
AKR1D1	Aldo-Keto Reductase Family 1 Member	Protein Coding	47	GC07P138003	29.05905914	https://www.genecards.org/cgi-bin/carddisp.pl?gene=AKR1D1
PCSK9	Proprotein Convertase Subtilisin/Kexin Type 9	Protein Coding	51	GC01P055039	28.94561386	https://www.genecards.org/cgi-bin/carddisp.pl?gene=PCSK9
VDR	Vitamin D Receptor	Protein Coding	52	GC12M047841	28.85274124	https://www.genecards.org/cgi-bin/carddisp.pl?gene=VDR
HNRNPA1	Heterogeneous Nuclear Ribonucleoprotein A1	Protein Coding	47	GC12P054280	28.8478508	https://www.genecards.org/cgi-bin/carddisp.pl?gene=HNRNPA1
ALMS1	ALMS1 Centrosome And Basal Body Associated Protein	Protein Coding	42	GC02P073385	28.76959801	https://www.genecards.org/cgi-bin/carddisp.pl?gene=ALMS1
SMAD3	SMAD Family Member 3	Protein Coding	50	GC15P067063	28.75627518	https://www.genecards.org/cgi-bin/carddisp.pl?gene=SMAD3
MIR195	MicroRNA 195	RNA Gene	19	GC17M007018	28.73178864	https://www.genecards.org/cgi-bin/carddisp.pl?gene=MIR195
CDKN3	Cyclin Dependent Kinase Inhibitor 3	Protein Coding	43	GC14P054398	28.72810555	https://www.genecards.org/cgi-bin/carddisp.pl?gene=CDKN3
GCK	Glucokinase	Protein Coding	51	GC07M044154	28.71801376	https://www.genecards.org/cgi-bin/carddisp.pl?gene=GCK
AHSG	Alpha 2-HS Glycoprotein	Protein Coding	44	GC03P186612	28.60829544	https://www.genecards.org/cgi-bin/carddisp.pl?gene=AHSG
CTSD	Cathepsin D	Protein Coding	52	GC11M001752	28.59474182	https://www.genecards.org/cgi-bin/carddisp.pl?gene=CTSD
SLC27A1	Solute Carrier Family 27 Member 1	Protein Coding	41	GC19P026639	28.4191494	https://www.genecards.org/cgi-bin/carddisp.pl?gene=SLC27A1
MAP3K5	Mitogen-Activated Protein Kinase Kinase Kinase 5	Protein Coding	48	GC06M136557	28.39349556	https://www.genecards.org/cgi-bin/carddisp.pl?gene=MAP3K5
NLRP3	NLR Family Pyrin Domain Containing 3	Protein Coding	48	GC01P247415	28.35830688	https://www.genecards.org/cgi-bin/carddisp.pl?gene=NLRP3
TFR2	Transferrin Receptor 2	Protein Coding	45	GC07M100620	28.3279171	https://www.genecards.org/cgi-bin/carddisp.pl?gene=TFR2
AGTR1	Angiotensin II Receptor Type 1	Protein Coding	51	GC03P148697	28.28638649	https://www.genecards.org/cgi-bin/carddisp.pl?gene=AGTR1
DSP	Desmoplakin	Protein Coding	50	GC06P007541	28.1997757	https://www.genecards.org/cgi-bin/carddisp.pl?gene=DSP
SLC2A4	Solute Carrier Family 2 Member 4	Protein Coding	47	GC17P009071	28.08086395	https://www.genecards.org/cgi-bin/carddisp.pl?gene=SLC2A4
DPP4	Dipeptidyl Peptidase 4	Protein Coding	50	GC02M161992	27.92372894	https://www.genecards.org/cgi-bin/carddisp.pl?gene=DPP4
MT-CO3	Mitochondrially Encoded Cytochrome C Oxidase III	Protein Coding	31	GCMTP009209	27.90365219	https://www.genecards.org/cgi-bin/carddisp.pl?gene=MT-CO3
SERPINC1	Serpin Family C Member 1	Protein Coding	49	GC01M174251	27.83083153	https://www.genecards.org/cgi-bin/carddisp.pl?gene=SERPINC1
CASR	Calcium Sensing Receptor	Protein Coding	51	GC03P122183	27.77983093	https://www.genecards.org/cgi-bin/carddisp.pl?gene=CASR
CES1	Carboxylesterase 1	Protein Coding	47	GC16M055836	27.63164139	https://www.genecards.org/cgi-bin/carddisp.pl?gene=CES1
APOA5	Apolipoprotein A5	Protein Coding	45	GC11M116789	27.54660797	https://www.genecards.org/cgi-bin/carddisp.pl?gene=APOA5
MIR31	MicroRNA 31	RNA Gene	20	GC09M021513	27.51093292	https://www.genecards.org/cgi-bin/carddisp.pl?gene=MIR31
C3	Complement C3	Protein Coding	48	GC19M006677	27.40309906	https://www.genecards.org/cgi-bin/carddisp.pl?gene=C3
MIR30A	MicroRNA 30a	RNA Gene	20	GC06M071403	27.36931419	https://www.genecards.org/cgi-bin/carddisp.pl?gene=MIR30A
PIK3R1	Phosphoinositide-3-Kinase Regulatory Subunit 1	Protein Coding	51	GC05P068215	27.31510544	https://www.genecards.org/cgi-bin/carddisp.pl?gene=PIK3R1
MIR200A	MicroRNA 200a	RNA Gene	22	GC01P002022	27.26833916	https://www.genecards.org/cgi-bin/carddisp.pl?gene=MIR200A
MIR23B	MicroRNA 23b	RNA Gene	19	GC09P095085	27.25074387	https://www.genecards.org/cgi-bin/carddisp.pl?gene=MIR23B
SOCS3	Suppressor Of Cytokine Signaling 3	Protein Coding	45	GC17M078356	27.24120712	https://www.genecards.org/cgi-bin/carddisp.pl?gene=SOCS3
MIR181B1	MicroRNA 181b-1	RNA Gene	19	GC01M198858	27.18358612	https://www.genecards.org/cgi-bin/carddisp.pl?gene=MIR181B1
BAX	BCL2 Associated X, Apoptosis Regulator	Protein Coding	49	GC19P048954	27.06827736	https://www.genecards.org/cgi-bin/carddisp.pl?gene=BAX
CCN2	Cellular Communication Network Factor 2	Protein Coding	40	GC06M131948	27.01944733	https://www.genecards.org/cgi-bin/carddisp.pl?gene=CCN2
THBD	Thrombomodulin	Protein Coding	44	GC20M023026	26.96779633	https://www.genecards.org/cgi-bin/carddisp.pl?gene=THBD
ADH1C	Alcohol Dehydrogenase 1C (Class I), Gamma Polypeptide	Protein Coding	43	GC04M099336	26.79531097	https://www.genecards.org/cgi-bin/carddisp.pl?gene=ADH1C
LIPE	Lipase E, Hormone Sensitive Type	Protein Coding	48	GC19M042401	26.70900536	https://www.genecards.org/cgi-bin/carddisp.pl?gene=LIPE
F3	Coagulation Factor III, Tissue Factor	Protein Coding	45	GC01M094530	26.66098785	https://www.genecards.org/cgi-bin/carddisp.pl?gene=F3
MIR199A1	MicroRNA 199a-1	RNA Gene	19	GC19M010817	26.62546349	https://www.genecards.org/cgi-bin/carddisp.pl?gene=MIR199A1
NAMPT	Nicotinamide Phosphoribosyltransferase	Protein Coding	48	GC07M106248	26.59061813	https://www.genecards.org/cgi-bin/carddisp.pl?gene=NAMPT
ALDH2	Aldehyde Dehydrogenase 2 Family	Protein Coding	51	GC12P111766	26.49488258	https://www.genecards.org/cgi-bin/carddisp.pl?gene=ALDH2
PKLR	Pyruvate Kinase L/R	Protein Coding	48	GC01M155289	26.48228836	https://www.genecards.org/cgi-bin/carddisp.pl?gene=PKLR
CYP7A1	Cytochrome P450 Family 7 Subfamily A Member 1	Protein Coding	44	GC08M058476	26.47492027	https://www.genecards.org/cgi-bin/carddisp.pl?gene=CYP7A1

ATP2A2	ATPase Sarcoplasmic/Endoplasmic Reticulum Ca2+ Transporting 2	Protein Coding	52	GC12P110280	26.42674637	https://www.genecards.org/cgi-bin/carddisp.pl?gene=ATP2A2
ASS1	Argininosuccinate Synthase 1	Protein Coding	49	GC09P130444	26.4234066	https://www.genecards.org/cgi-bin/carddisp.pl?gene=ASS1
ACTB	Actin Beta	Protein Coding	50	GC07M005527	26.38704491	https://www.genecards.org/cgi-bin/carddisp.pl?gene=ACTB
NDUFS4	NADH:Ubiquinone Oxidoreductase Subunit S4	Protein Coding	45	GC05P053560	26.36944962	https://www.genecards.org/cgi-bin/carddisp.pl?gene=NDUFS4
SDHA	Succinate Dehydrogenase Complex Flavoprotein Subunit A	Protein Coding	48	GC05P000208	26.30423355	https://www.genecards.org/cgi-bin/carddisp.pl?gene=SDHA
FN1	Fibronectin 1	Protein Coding	50	GC02M215360	26.24593925	https://www.genecards.org/cgi-bin/carddisp.pl?gene=FN1
GHRL	Ghrelin And Obestatin Prepropeptide	Protein Coding	44	GC03M010285	26.2089653	https://www.genecards.org/cgi-bin/carddisp.pl?gene=GHRL
SMARCA4	SWI/SNF Related, Matrix Associated, Actin Dependent Regulator Of Chromatin, Subfamily A, Member 4	Protein Coding	50	GC19P010932	26.17413139	https://www.genecards.org/cgi-bin/carddisp.pl?gene=SMARCA4
IKBKB	Inhibitor Of Nuclear Factor Kappa B Kinase Subunit Beta	Protein Coding	53	GC08P042271	26.08623123	https://www.genecards.org/cgi-bin/carddisp.pl?gene=IKBKB
NDUFV1	NADH:Ubiquinone Oxidoreductase Core Subunit V1	Protein Coding	47	GC11P067670	26.07613182	https://www.genecards.org/cgi-bin/carddisp.pl?gene=NDUFV1
SREBF2	Sterol Regulatory Element Binding Transcription Factor 2	Protein Coding	43	GC22P041833	26.01618958	https://www.genecards.org/cgi-bin/carddisp.pl?gene=SREBF2
ACACB	Acetyl-CoA Carboxylase Beta	Protein Coding	47	GC12P109116	25.94504547	https://www.genecards.org/cgi-bin/carddisp.pl?gene=ACACB
HIF1A	Hypoxia Inducible Factor 1 Subunit Alpha	Protein Coding	48	GC14P061695	25.86544037	https://www.genecards.org/cgi-bin/carddisp.pl?gene=HIF1A
HJV	Hemojuvelin BMP Co-Receptor	Protein Coding	36	GC01M146018	25.79007912	https://www.genecards.org/cgi-bin/carddisp.pl?gene=HJV
FGF21	Fibroblast Growth Factor 21	Protein Coding	40	GC19P048781	25.7541008	https://www.genecards.org/cgi-bin/carddisp.pl?gene=FGF21
ALPL	Alkaline Phosphatase, Biomineralization Associated	Protein Coding	51	GC01P021508	25.72567749	https://www.genecards.org/cgi-bin/carddisp.pl?gene=ALPL
GSK3B	Glycogen Synthase Kinase 3 Beta	Protein Coding	51	GC03M119821	25.71948242	https://www.genecards.org/cgi-bin/carddisp.pl?gene=GSK3B
CYP2A6	Cytochrome P450 Family 2 Subfamily A Member 6	Protein Coding	48	GC19M040843	25.65201378	https://www.genecards.org/cgi-bin/carddisp.pl?gene=CYP2A6
MT-ND3	Mitochondrially Encoded NADH:Ubiquinone Oxidoreductase Core	Protein Coding	33	GCMTP010061	25.59578133	https://www.genecards.org/cgi-bin/carddisp.pl?gene=MT-ND3
AGPAT2	1-Acylglycerol-3-Phosphate O-Acyltransferase 2	Protein Coding	47	GC09M136673	25.53592682	https://www.genecards.org/cgi-bin/carddisp.pl?gene=AGPAT2
MIR224	MicroRNA 224	RNA Gene	17	GC0XM151958	25.41087723	https://www.genecards.org/cgi-bin/carddisp.pl?gene=MIR224
MSH6	MutS Homolog 6	Protein Coding	51	GC02P047695	25.33275223	https://www.genecards.org/cgi-bin/carddisp.pl?gene=MSH6
MT-ND6	Mitochondrially Encoded NADH:Ubiquinone Oxidoreductase Core	Protein Coding	33	GCMTM014151	25.23871994	https://www.genecards.org/cgi-bin/carddisp.pl?gene=MT-ND6
IL6R	Interleukin 6 Receptor	Protein Coding	50	GC01P154405	25.18363953	https://www.genecards.org/cgi-bin/carddisp.pl?gene=IL6R
BMP6	Bone Morphogenetic Protein 6	Protein Coding	44	GC06P007726	25.16589737	https://www.genecards.org/cgi-bin/carddisp.pl?gene=BMP6
HSPD1	Heat Shock Protein Family D (Hsp60) Member 1	Protein Coding	48	GC02M197486	25.16395569	https://www.genecards.org/cgi-bin/carddisp.pl?gene=HSPD1
FFAR4	Free Fatty Acid Receptor 4	Protein Coding	39	GC10P093566	25.0192318	https://www.genecards.org/cgi-bin/carddisp.pl?gene=FFAR4
TNFRSF1B	TNF Receptor Superfamily Member 1B	Protein Coding	48	GC01P012167	24.98200226	https://www.genecards.org/cgi-bin/carddisp.pl?gene=TNFRSF1B
PRKAB1	Protein Kinase AMP-Activated Non-Catalytic Subunit Beta 1	Protein Coding	48	GC12P119632	24.95871735	https://www.genecards.org/cgi-bin/carddisp.pl?gene=PRKAB1
PRKAA1	Protein Kinase AMP-Activated Catalytic Subunit Alpha 1	Protein Coding	48	GC05M040759	24.95615959	https://www.genecards.org/cgi-bin/carddisp.pl?gene=PRKAA1
BCL2	BCL2 Apoptosis Regulator	Protein Coding	51	GC18M063123	24.95194244	https://www.genecards.org/cgi-bin/carddisp.pl?gene=BCL2
COX5A	Cytochrome C Oxidase Subunit 5A	Protein Coding	45	GC15M074919	24.93875122	https://www.genecards.org/cgi-bin/carddisp.pl?gene=COX5A
H19	H19 Imprinted Maternally Expressed Transcript	RNA Gene	28	GC11M001995	24.76796341	https://www.genecards.org/cgi-bin/carddisp.pl?gene=H19
ELANE	Elastase, Neutrophil Expressed	Protein Coding	47	GC19P000855	24.69678497	https://www.genecards.org/cgi-bin/carddisp.pl?gene=ELANE
RARRES2	Retinoic Acid Receptor Responder 2	Protein Coding	40	GC07M150333	24.6766777	https://www.genecards.org/cgi-bin/carddisp.pl?gene=RARRES2
UGT1A7	UDP Glucuronosyltransferase Family 1 Member A7	Protein Coding	39	GC02P233681	24.61125755	https://www.genecards.org/cgi-bin/carddisp.pl?gene=UGT1A7
VIM	Vimentin	Protein Coding	51	GC10P017227	24.5502224	https://www.genecards.org/cgi-bin/carddisp.pl?gene=VIM
ITCH	Itchy E3 Ubiquitin Protein Ligase	Protein Coding	46	GC20P034363	24.52334976	https://www.genecards.org/cgi-bin/carddisp.pl?gene=ITCH
AIFM1	Apoptosis Inducing Factor Mitochondria Associated 1	Protein Coding	50	GC0XM130129	24.51913452	https://www.genecards.org/cgi-bin/carddisp.pl?gene=AIFM1
CDKN1A	Cyclin Dependent Kinase Inhibitor 1A	Protein Coding	50	GC06P055348	24.48690414	https://www.genecards.org/cgi-bin/carddisp.pl?gene=CDKN1A
FGF23	Fibroblast Growth Factor 23	Protein Coding	46	GC12M004368	24.33061218	https://www.genecards.org/cgi-bin/carddisp.pl?gene=FGF23
PLIN2	Perilipin 2	Protein Coding	44	GC09M019127	24.32180786	https://www.genecards.org/cgi-bin/carddisp.pl?gene=PLIN2
MIR200C	MicroRNA 200c	RNA Gene	21	GC12P011871	24.12338257	https://www.genecards.org/cgi-bin/carddisp.pl?gene=MIR200C
FGA	Fibrinogen Alpha Chain	Protein Coding	48	GC04M154583	24.08589172	https://www.genecards.org/cgi-bin/carddisp.pl?gene=FGA
A2M	Alpha-2-Macroglobulin	Protein Coding	47	GC12M009067	23.90711975	https://www.genecards.org/cgi-bin/carddisp.pl?gene=A2M
MIR125A	MicroRNA 125a	RNA Gene	21	GC19P051720	23.89912224	https://www.genecards.org/cgi-bin/carddisp.pl?gene=MIR125A
MT-CO2	Mitochondrially Encoded Cytochrome C Oxidase II	Protein Coding	34	GCMTP007587	23.81887436	https://www.genecards.org/cgi-bin/carddisp.pl?gene=MT-CO2
BTNL2	Butyrophilin Like 2	Protein Coding	39	GC06M032393	23.80173492	https://www.genecards.org/cgi-bin/carddisp.pl?gene=BTNL2
LPA	Lipoprotein(A)	Protein Coding	42	GC06M160531	23.78948212	https://www.genecards.org/cgi-bin/carddisp.pl?gene=LPA
SDHC	Succinate Dehydrogenase Complex Subunit C	Protein Coding	45	GC01P161314	23.74804688	https://www.genecards.org/cgi-bin/carddisp.pl?gene=SDHC
SELE	Selectin E	Protein Coding	44	GC01M169722	23.58281708	https://www.genecards.org/cgi-bin/carddisp.pl?gene=SELE
IGF1R	Insulin Like Growth Factor 1 Receptor	Protein Coding	55	GC15P098648	23.55892944	https://www.genecards.org/cgi-bin/carddisp.pl?gene=IGF1R
NR1I2	Nuclear Receptor Subfamily 1 Group I Member 2	Protein Coding	47	GC03P119780	23.4993782	https://www.genecards.org/cgi-bin/carddisp.pl?gene=NR1I2
FTL	Ferritin Light Chain	Protein Coding	48	GC19P048965	23.48954773	https://www.genecards.org/cgi-bin/carddisp.pl?gene=FTL
PDGFRB	Platelet Derived Growth Factor Receptor Beta	Protein Coding	55	GC05M150113	23.46020126	https://www.genecards.org/cgi-bin/carddisp.pl?gene=PDGFRB
UGT1A6	UDP Glucuronosyltransferase Family 1 Member A6	Protein Coding	42	GC02P233691	23.270998	https://www.genecards.org/cgi-bin/carddisp.pl?gene=UGT1A6
MSR1	Macrophage Scavenger Receptor 1	Protein Coding	47	GC08M016107	23.25499344	https://www.genecards.org/cgi-bin/carddisp.pl?gene=MSR1
NPPB	Natriuretic Peptide B	Protein Coding	44	GC01M011858	23.25296783	https://www.genecards.org/cgi-bin/carddisp.pl?gene=NPPB
CXCL10	C-X-C Motif Chemokine Ligand 10	Protein Coding	45	GC04M076021	23.22422409	https://www.genecards.org/cgi-bin/carddisp.pl?gene=CXCL10
IFIH1	Interferon Induced With Helicase C Domain 1	Protein Coding	48	GC02M162267	23.21790695	https://www.genecards.org/cgi-bin/carddisp.pl?gene=IFIH1
XDH	Xanthine Dehydrogenase	Protein Coding	47	GC02M031334	23.17886925	https://www.genecards.org/cgi-bin/carddisp.pl?gene=XDH
ATF4	Activating Transcription Factor 4	Protein Coding	47	GC22P039547	23.17003822	https://www.genecards.org/cgi-bin/carddisp.pl?gene=ATF4
MYH9	Myosin Heavy Chain 9	Protein Coding	50	GC22M036281	23.12662125	https://www.genecards.org/cgi-bin/carddisp.pl?gene=MYH9
TGFBR1	Transforming Growth Factor Beta Receptor 1	Protein Coding	54	GC09P099104	23.08447647	https://www.genecards.org/cgi-bin/carddisp.pl?gene=TGFBR1
IDH1	Isocitrate Dehydrogenase (NADP(+)) 1	Protein Coding	53	GC02M208236	23.08345795	https://www.genecards.org/cgi-bin/carddisp.pl?gene=IDH1
KRT7	Keratin 7	Protein Coding	43	GC12P052232	23.07139206	https://www.genecards.org/cgi-bin/carddisp.pl?gene=KRT7
KRT19	Keratin 19	Protein Coding	46	GC17M041523	23.06446457	https://www.genecards.org/cgi-bin/carddisp.pl?gene=KRT19
ADA2	Adenosine Deaminase 2	Protein Coding	34	GC22M017179	23.01762009	https://www.genecards.org/cgi-bin/carddisp.pl?gene=ADA2
POMC	Proopiomelanocortin	Protein Coding	48	GC02M025160	22.94438934	https://www.genecards.org/cgi-bin/carddisp.pl?gene=POMC
NFE2L2	Nuclear Factor, Erythroid 2 Like 2	Protein Coding	50	GC02M177227	22.85059738	https://www.genecards.org/cgi-bin/carddisp.pl?gene=NFE2L2
ALPP	Alkaline Phosphatase, Placental	Protein Coding	47	GC02P232378	22.80949783	https://www.genecards.org/cgi-bin/carddisp.pl?gene=ALPP
TLR9	Toll Like Receptor 9	Protein Coding	45	GC03M052222	22.79990768	https://www.genecards.org/cgi-bin/carddisp.pl?gene=TLR9
MIR130A	MicroRNA 130a	RNA Gene	20	GC11P057641	22.72928238	https://www.genecards.org/cgi-bin/carddisp.pl?gene=MIR130A
MAPK14	Mitogen-Activated Protein Kinase 14	Protein Coding	52	GC06P055339	22.71913147	https://www.genecards.org/cgi-bin/carddisp.pl?gene=MAPK14
PRKCQ	Protein Kinase C Theta	Protein Coding	50	GC10M006393	22.58216286	https://www.genecards.org/cgi-bin/carddisp.pl?gene=PRKCQ
VCAM1	Vascular Cell Adhesion Molecule 1	Protein Coding	46	GC01P100719	22.57697296	https://www.genecards.org/cgi-bin/carddisp.pl?gene=VCAM1
UGT1A8	UDP Glucuronosyltransferase Family 1 Member A8	Protein Coding	36	GC02P233618	22.53224373	https://www.genecards.org/cgi-bin/carddisp.pl?gene=UGT1A8
NDUFS2	NADH:Ubiquinone Oxidoreductase Core Subunit S2	Protein Coding	45	GC01P161197	22.51537323	https://www.genecards.org/cgi-bin/carddisp.pl?gene=NDUFS2
FGF19	Fibroblast Growth Factor 19	Protein Coding	44	GC11M070382	22.49826813	https://www.genecards.org/cgi-bin/carddisp.pl?gene=FGF19

ERN1	Endoplasmic Reticulum To Nucleus Signaling 1	Protein Coding	46	GC17M064039	22.49084854	https://www.genecards.org/cgi-bin/carddisp.pl?gene=ERN1
SPINK1	Serine Peptidase Inhibitor Kazal Type 1	Protein Coding	44	GC05M147825	22.4596138	https://www.genecards.org/cgi-bin/carddisp.pl?gene=SPINK1
EIF2AK4	Eukaryotic Translation Initiation Factor 2 Alpha Kinase 4	Protein Coding	45	GC15P039934	22.43599892	https://www.genecards.org/cgi-bin/carddisp.pl?gene=EIF2AK4
BGLAP	Bone Gamma-Carboxyglutamate Protein	Protein Coding	41	GC01P156242	22.3146019	https://www.genecards.org/cgi-bin/carddisp.pl?gene=BGLAP
CCL5	C-C Motif Chemokine Ligand 5	Protein Coding	44	GC17M035871	22.25790977	https://www.genecards.org/cgi-bin/carddisp.pl?gene=CCL5
TBP	TATA-Box Binding Protein	Protein Coding	49	GC06P170554	22.20865059	https://www.genecards.org/cgi-bin/carddisp.pl?gene=TBP
MIR16-1	MicroRNA 16-1	RNA Gene	22	GC13M050048	22.20575523	https://www.genecards.org/cgi-bin/carddisp.pl?gene=MIR16-1
MIR29B1	MicroRNA 29b-1	RNA Gene	20	GC07M130877	22.15891647	https://www.genecards.org/cgi-bin/carddisp.pl?gene=MIR29B1
IFNA2	Interferon Alpha 2	Protein Coding	41	GC09M021384	22.1397171	https://www.genecards.org/cgi-bin/carddisp.pl?gene=IFNA2
NDUFS7	NADH:Ubiquinone Oxidoreductase Core Subunit S7	Protein Coding	47	GC19P001635	22.11656189	https://www.genecards.org/cgi-bin/carddisp.pl?gene=NDUFS7
SHH	Sonic Hedgehog Signaling Molecule	Protein Coding	50	GC07M155799	22.06713486	https://www.genecards.org/cgi-bin/carddisp.pl?gene=SHH
MIRLET7D	MicroRNA Let-7d	RNA Gene	21	GC09P094178	21.96260452	https://www.genecards.org/cgi-bin/carddisp.pl?gene=MIRLET7D
EIF2S1	Eukaryotic Translation Initiation Factor 2 Subunit Alpha	Protein Coding	45	GC14P067359	21.94793892	https://www.genecards.org/cgi-bin/carddisp.pl?gene=EIF2S1
LCN2	Lipocalin 2	Protein Coding	44	GC09P128149	21.90474701	https://www.genecards.org/cgi-bin/carddisp.pl?gene=LCN2
UGT1A9	UDP Glucuronosyltransferase Family 1 Member A9	Protein Coding	44	GC02P233671	21.84596825	https://www.genecards.org/cgi-bin/carddisp.pl?gene=UGT1A9
HBA1	Hemoglobin Subunit Alpha 1	Protein Coding	43	GC16P005490	21.71658516	https://www.genecards.org/cgi-bin/carddisp.pl?gene=HBA1
RXRA	Retinoid X Receptor Alpha	Protein Coding	51	GC09P134317	21.71615219	https://www.genecards.org/cgi-bin/carddisp.pl?gene=RXRA
SELP	Selectin P	Protein Coding	46	GC01M169558	21.67138672	https://www.genecards.org/cgi-bin/carddisp.pl?gene=SELP
CNR1	Cannabinoid Receptor 1	Protein Coding	47	GC06M088139	21.61171913	https://www.genecards.org/cgi-bin/carddisp.pl?gene=CNR1
CXCR4	C-X-C Motif Chemokine Receptor 4	Protein Coding	52	GC02M136114	21.59842873	https://www.genecards.org/cgi-bin/carddisp.pl?gene=CXCR4
MIRLET7C	MicroRNA Let-7c	RNA Gene	22	GC21P016551	21.58302879	https://www.genecards.org/cgi-bin/carddisp.pl?gene=MIRLET7C
LGALS3	Galectin 3	Protein Coding	45	GC14P055124	21.55549622	https://www.genecards.org/cgi-bin/carddisp.pl?gene=LGALS3
TET2	Tet Methylcytosine Dioxygenase 2	Protein Coding	45	GC04P105145	21.51708221	https://www.genecards.org/cgi-bin/carddisp.pl?gene=TET2
EP300	E1A Binding Protein P300	Protein Coding	51	GC22P041091	21.46718597	https://www.genecards.org/cgi-bin/carddisp.pl?gene=EP300
TCF7L2	Transcription Factor 7 Like 2	Protein Coding	46	GC10P112950	21.46273422	https://www.genecards.org/cgi-bin/carddisp.pl?gene=TCF7L2
ADRB2	Adrenoceptor Beta 2	Protein Coding	50	GC05P148825	21.45920944	https://www.genecards.org/cgi-bin/carddisp.pl?gene=ADRB2
NDUFV2	NADH:Ubiquinone Oxidoreductase Core Subunit V2	Protein Coding	45	GC18P009092	21.40446472	https://www.genecards.org/cgi-bin/carddisp.pl?gene=NDUFV2
MIR33A	MicroRNA 33a	RNA Gene	19	GC22P041900	21.35975266	https://www.genecards.org/cgi-bin/carddisp.pl?gene=MIR33A
MST1	Macrophage Stimulating 1	Protein Coding	46	GC03M049683	21.30723381	https://www.genecards.org/cgi-bin/carddisp.pl?gene=MST1
COX6A1	Cytochrome C Oxidase Subunit 6A1	Protein Coding	45	GC12P120439	21.29756927	https://www.genecards.org/cgi-bin/carddisp.pl?gene=COX6A1
LIVAR	Liver Cell Viability Associated LncRNA	RNA Gene	10	GC18M070336	21.27138519	https://www.genecards.org/cgi-bin/carddisp.pl?gene=LIVAR
CD14	CD14 Molecule	Protein Coding	45	GC05M140631	21.18961334	https://www.genecards.org/cgi-bin/carddisp.pl?gene=CD14
FLT1	Fms Related Receptor Tyrosine Kinase 1	Protein Coding	51	GC13M028300	21.17339325	https://www.genecards.org/cgi-bin/carddisp.pl?gene=FLT1
LRP6	LDL Receptor Related Protein 6	Protein Coding	49	GC12M015714	21.11747742	https://www.genecards.org/cgi-bin/carddisp.pl?gene=LRP6
MIR26A1	MicroRNA 26a-1	RNA Gene	20	GC03P037969	21.05493164	https://www.genecards.org/cgi-bin/carddisp.pl?gene=MIR26A1
EIF2AK3	Eukaryotic Translation Initiation Factor 2 Alpha Kinase 3	Protein Coding	50	GC02M088556	21.0508213	https://www.genecards.org/cgi-bin/carddisp.pl?gene=EIF2AK3
TNFAIP3	TNF Alpha Induced Protein 3	Protein Coding	48	GC06P137866	21.04412079	https://www.genecards.org/cgi-bin/carddisp.pl?gene=TNFAIP3
IGFBP3	Insulin Like Growth Factor Binding	Protein Coding	45	GC07M045912	21.04045677	https://www.genecards.org/cgi-bin/carddisp.pl?gene=IGFBP3
MIR141	MicroRNA 141	RNA Gene	22	GC12P011872	21.01330948	https://www.genecards.org/cgi-bin/carddisp.pl?gene=MIR141
MIR183	MicroRNA 183	RNA Gene	18	GC07M129798	21.00595474	https://www.genecards.org/cgi-bin/carddisp.pl?gene=MIR183
NDUFB9	NADH:Ubiquinone Oxidoreductase Subunit B9	Protein Coding	45	GC08P124539	20.99195862	https://www.genecards.org/cgi-bin/carddisp.pl?gene=NDUFB9
ERCC2	ERCC Excision Repair 2, TFIIH Core Complex Helicase Subunit	Protein Coding	48	GC19M045349	20.98767281	https://www.genecards.org/cgi-bin/carddisp.pl?gene=ERCC2
TNFSF11	TNF Superfamily Member 11	Protein Coding	48	GC13P042562	20.97611237	https://www.genecards.org/cgi-bin/carddisp.pl?gene=TNFSF11
MIR144	MicroRNA 144	RNA Gene	17	GC17M031086	20.89792252	https://www.genecards.org/cgi-bin/carddisp.pl?gene=MIR144
MDM2	MDM2 Proto-Oncogene	Protein Coding	53	GC12P068808	20.87329674	https://www.genecards.org/cgi-bin/carddisp.pl?gene=MDM2
MIR451A	MicroRNA 451a	RNA Gene	18	GC17M028861	20.86265373	https://www.genecards.org/cgi-bin/carddisp.pl?gene=MIR451A
CPS1	Carbamoyl-Phosphate Synthase 1	Protein Coding	46	GC02P210477	20.84814072	https://www.genecards.org/cgi-bin/carddisp.pl?gene=CPS1
DGAT2	Diacylglycerol O-Acyltransferase 2	Protein Coding	44	GC11P075759	20.81304932	https://www.genecards.org/cgi-bin/carddisp.pl?gene=DGAT2
SHBG	Sex Hormone Binding Globulin	Protein Coding	41	GC17P007613	20.80622482	https://www.genecards.org/cgi-bin/carddisp.pl?gene=SHBG
CTSB	Cathepsin B	Protein Coding	51	GC08M011842	20.73703384	https://www.genecards.org/cgi-bin/carddisp.pl?gene=CTSB
NR5A2	Nuclear Receptor Subfamily 5 Group A Member 2	Protein Coding	47	GC01P199996	20.71928215	https://www.genecards.org/cgi-bin/carddisp.pl?gene=NR5A2
DNMT1	DNA Methyltransferase 1	Protein Coding	51	GC19M010133	20.66369629	https://www.genecards.org/cgi-bin/carddisp.pl?gene=DNMT1
CD44	CD44 Molecule (Indian Blood Group)	Protein Coding	48	GC11P035139	20.63989639	https://www.genecards.org/cgi-bin/carddisp.pl?gene=CD44
APOA2	Apolipoprotein A2	Protein Coding	44	GC01M161222	20.63027	https://www.genecards.org/cgi-bin/carddisp.pl?gene=APOA2
CHIT1	Chitinase 1	Protein Coding	44	GC01M203181	20.58336639	https://www.genecards.org/cgi-bin/carddisp.pl?gene=CHIT1
COL3A1	Collagen Type III Alpha 1 Chain	Protein Coding	47	GC02P188974	20.57392311	https://www.genecards.org/cgi-bin/carddisp.pl?gene=COL3A1
IL21	Interleukin 21	Protein Coding	43	GC04M122612	20.53334045	https://www.genecards.org/cgi-bin/carddisp.pl?gene=IL21
CLU	Clusterin	Protein Coding	47	GC08M027596	20.50471687	https://www.genecards.org/cgi-bin/carddisp.pl?gene=CLU
ABCB7	ATP Binding Cassette Subfamily B Member 7	Protein Coding	44	GC0XM075053	20.49568176	https://www.genecards.org/cgi-bin/carddisp.pl?gene=ABCB7
UGT1A4	UDP Glucuronosyltransferase Family 1 Member A4	Protein Coding	42	GC02P233718	20.49494934	https://www.genecards.org/cgi-bin/carddisp.pl?gene=UGT1A4
NDUFS3	NADH:Ubiquinone Oxidoreductase Core Subunit S3	Protein Coding	48	GC11P047567	20.49392509	https://www.genecards.org/cgi-bin/carddisp.pl?gene=NDUFS3
GHR	Growth Hormone Receptor	Protein Coding	47	GC05P042429	20.44636154	https://www.genecards.org/cgi-bin/carddisp.pl?gene=GHR
MYD88	MYD88 Innate Immune Signal Transduction Adaptor	Protein Coding	50	GC03P038139	20.36114693	https://www.genecards.org/cgi-bin/carddisp.pl?gene=MYD88
MIR182	MicroRNA 182	RNA Gene	20	GC07M129770	20.32253456	https://www.genecards.org/cgi-bin/carddisp.pl?gene=MIR182
SLC10A1	Solute Carrier Family 10 Member 1	Protein Coding	45	GC14M069775	20.31093025	https://www.genecards.org/cgi-bin/carddisp.pl?gene=SLC10A1
COPA	COPI Coat Complex Subunit Alpha	Protein Coding	43	GC01M160288	20.20817757	https://www.genecards.org/cgi-bin/carddisp.pl?gene=COPA
UQCRB	Ubiquinol-Cytochrome C Reductase Binding Protein	Protein Coding	44	GC08M096225	20.19953156	https://www.genecards.org/cgi-bin/carddisp.pl?gene=UQCRB
SCARB1	Scavenger Receptor Class B Member 1	Protein Coding	46	GC12M124776	20.18565941	https://www.genecards.org/cgi-bin/carddisp.pl?gene=SCARB1
PC	Pyruvate Carboxylase	Protein Coding	48	GC11M066848	20.17343903	https://www.genecards.org/cgi-bin/carddisp.pl?gene=PC
PPARD	Peroxisome Proliferator Activated Receptor Delta	Protein Coding	48	GC06P055324	20.14023972	https://www.genecards.org/cgi-bin/carddisp.pl?gene=PPARD
LOX	Lysyl Oxidase	Protein Coding	45	GC05M122063	20.06209946	https://www.genecards.org/cgi-bin/carddisp.pl?gene=LOX
NGF	Nerve Growth Factor	Protein Coding	51	GC01M115285	20.05589676	https://www.genecards.org/cgi-bin/carddisp.pl?gene=NGF
PLA2G4A	Phospholipase A2 Group IVA	Protein Coding	50	GC01P186798	20.0489502	https://www.genecards.org/cgi-bin/carddisp.pl?gene=PLA2G4A
CDH2	Cadherin 2	Protein Coding	53	GC18M027950	19.97754669	https://www.genecards.org/cgi-bin/carddisp.pl?gene=CDH2
MIRLET7E	MicroRNA Let-7e	RNA Gene	20	GC19P051718	19.92986679	https://www.genecards.org/cgi-bin/carddisp.pl?gene=MIRLET7E
IL1R1	Interleukin 1 Receptor Type 1	Protein Coding	46	GC02P102136	19.91479301	https://www.genecards.org/cgi-bin/carddisp.pl?gene=IL1R1
IGFBP1	Insulin Like Growth Factor Binding	Protein Coding	44	GC07P046838	19.88154602	https://www.genecards.org/cgi-bin/carddisp.pl?gene=IGFBP1
UGT1A10	UDP Glucuronosyltransferase Family 1 Member A10	Protein Coding	41	GC02P233636	19.87574959	https://www.genecards.org/cgi-bin/carddisp.pl?gene=UGT1A10
CYP4A11	Cytochrome P450 Family 4 Subfamily A Member 11	Protein Coding	44	GC01M046929	19.8310051	https://www.genecards.org/cgi-bin/carddisp.pl?gene=CYP4A11
MIR151A	MicroRNA 151a	RNA Gene	18	GC08M140733	19.82871246	https://www.genecards.org/cgi-bin/carddisp.pl?gene=MIR151A
TIMP2	TIMP Metallopeptidase Inhibitor 2	Protein Coding	44	GC17M078852	19.76366043	https://www.genecards.org/cgi-bin/carddisp.pl?gene=TIMP2
PPIG	Peptidylprolyl Isomerase G	Protein Coding	43	GC02P169584	19.74473572	https://www.genecards.org/cgi-bin/carddisp.pl?gene=PPIG
GH1	Growth Hormone 1	Protein Coding	44	GC17M063917	19.72757721	https://www.genecards.org/cgi-bin/carddisp.pl?gene=GH1
ACSL3	Acyl-CoA Synthetase Long Chain Family Member 3	Protein Coding	41	GC02P222860	19.67117882	https://www.genecards.org/cgi-bin/carddisp.pl?gene=ACSL3
SLC6A3	Solute Carrier Family 6 Member 3	Protein Coding	50	GC05M001392	19.65219879	https://www.genecards.org/cgi-bin/carddisp.pl?gene=SLC6A3
CDK4	Cyclin Dependent Kinase 4	Protein Coding	54	GC12M057743	19.62770844	https://www.genecards.org/cgi-bin/carddisp.pl?gene=CDK4
CRYAA	Crystallin Alpha A	Protein Coding	45	GC21P043169	19.57661819	https://www.genecards.org/cgi-bin/carddisp.pl?gene=CRYAA
ERBB4	Erb-B2 Receptor Tyrosine Kinase 4	Protein Coding	55	GC02M211375	19.56996918	https://www.genecards.org/cgi-bin/carddisp.pl?gene=ERBB4
TNFSF10	TNF Superfamily Member 10	Protein Coding	47	GC03M172505	19.5359993	https://www.genecards.org/cgi-bin/carddisp.pl?gene=TNFSF10

EPHX1	Epoxide Hydrolase 1	Protein Coding	48	GC01P225810	19.52431297	https://www.genecards.org/cgi-bin/carddisp.pl?gene=EPHX1
UGT1A3	UDP Glucuronosyltransferase Family 1 Member A3	Protein Coding	39	GC02P233729	19.51120567	https://www.genecards.org/cgi-bin/carddisp.pl?gene=UGT1A3
IL18BP	Interleukin 18 Binding Protein	Protein Coding	40	GC11P071998	19.50037193	https://www.genecards.org/cgi-bin/carddisp.pl?gene=IL18BP
HSPA5	Heat Shock Protein Family A (Hsp70) Member 5	Protein Coding	48	GC09M125234	19.48897552	https://www.genecards.org/cgi-bin/carddisp.pl?gene=HSPA5
SLC6A4	Solute Carrier Family 6 Member 4	Protein Coding	48	GC17M030194	19.44141388	https://www.genecards.org/cgi-bin/carddisp.pl?gene=SLC6A4
SIRT3	Sirtuin 3	Protein Coding	48	GC11M000215	19.38682938	https://www.genecards.org/cgi-bin/carddisp.pl?gene=SIRT3
NPY	Neuropeptide Y	Protein Coding	46	GC07P024290	19.33066177	https://www.genecards.org/cgi-bin/carddisp.pl?gene=NPY
SPARC	Secreted Protein Acidic And Cysteine Rich	Protein Coding	51	GC05M151661	19.32937622	https://www.genecards.org/cgi-bin/carddisp.pl?gene=SPARC
MIR378A	MicroRNA 378a	RNA Gene	19	GC05P149732	19.31931877	https://www.genecards.org/cgi-bin/carddisp.pl?gene=MIR378A
ADH1B	Alcohol Dehydrogenase 1B (Class I), Beta Polypeptide	Protein Coding	44	GC04M099304	19.2811203	https://www.genecards.org/cgi-bin/carddisp.pl?gene=ADH1B
KHK	Ketohexokinase	Protein Coding	45	GC02P027086	19.27867317	https://www.genecards.org/cgi-bin/carddisp.pl?gene=KHK
CEL	Carboxyl Ester Lipase	Protein Coding	47	GC09P133061	19.21060181	https://www.genecards.org/cgi-bin/carddisp.pl?gene=CEL
MIR192	MicroRNA 192	RNA Gene	22	GC11M064891	19.18599129	https://www.genecards.org/cgi-bin/carddisp.pl?gene=MIR192
GNMT	Glycine N-Methyltransferase	Protein Coding	46	GC06P042960	19.16465569	https://www.genecards.org/cgi-bin/carddisp.pl?gene=GNMT
MIR429	MicroRNA 429	RNA Gene	19	GC01P002023	19.11920357	https://www.genecards.org/cgi-bin/carddisp.pl?gene=MIR429
UCP3	Uncoupling Protein 3	Protein Coding	44	GC11M074000	19.11037827	https://www.genecards.org/cgi-bin/carddisp.pl?gene=UCP3
MEG3	Maternally Expressed 3	RNA Gene	30	GC14P106296	19.07384491	https://www.genecards.org/cgi-bin/carddisp.pl?gene=MEG3
CRAT	Carnitine O-Acetyltransferase	Protein Coding	45	GC09M129094	18.94405556	https://www.genecards.org/cgi-bin/carddisp.pl?gene=CRAT
NDUFA13	NADH:Ubiquinone Oxidoreductase Subunit A13	Protein Coding	45	GC19P019515	18.90237999	https://www.genecards.org/cgi-bin/carddisp.pl?gene=NDUFA13
CYP1B1	Cytochrome P450 Family 1 Subfamily B Member 1	Protein Coding	49	GC02M038066	18.88570023	https://www.genecards.org/cgi-bin/carddisp.pl?gene=CYP1B1
NEU1	Neuraminidase 1	Protein Coding	45	GC06M031857	18.82324219	https://www.genecards.org/cgi-bin/carddisp.pl?gene=NEU1
IL15	Interleukin 15	Protein Coding	41	GC04P141636	18.78727341	https://www.genecards.org/cgi-bin/carddisp.pl?gene=IL15
NAT2	N-Acetyltransferase 2	Protein Coding	44	GC08P018391	18.71180725	https://www.genecards.org/cgi-bin/carddisp.pl?gene=NAT2
FNDC5	Fibronectin Type III Domain Containing 5	Protein Coding	34	GC01M032866	18.67414093	https://www.genecards.org/cgi-bin/carddisp.pl?gene=FNDC5
NDUFA1	NADH:Ubiquinone Oxidoreductase Subunit A1	Protein Coding	45	GC0XP119871	18.66812134	https://www.genecards.org/cgi-bin/carddisp.pl?gene=NDUFA1
GLUD1	Glutamate Dehydrogenase 1	Protein Coding	50	GC10M087050	18.65625572	https://www.genecards.org/cgi-bin/carddisp.pl?gene=GLUD1
CASP9	Caspase 9	Protein Coding	48	GC01M015491	18.55427933	https://www.genecards.org/cgi-bin/carddisp.pl?gene=CASP9
GC	GC Vitamin D Binding Protein	Protein Coding	44	GC04M071741	18.5395565	https://www.genecards.org/cgi-bin/carddisp.pl?gene=GC
PRKAA2	Protein Kinase AMP-Activated Catalytic Subunit Alpha 2	Protein Coding	51	GC01P056645	18.48451614	https://www.genecards.org/cgi-bin/carddisp.pl?gene=PRKAA2
GSTT1	Glutathione S-Transferase Theta 1	Protein Coding	33	GC22Mi00270	18.47049713	https://www.genecards.org/cgi-bin/carddisp.pl?gene=GSTT1
EIF4G1	Eukaryotic Translation Initiation Factor 4 Gamma 1	Protein Coding	47	GC03P184314	18.41026306	https://www.genecards.org/cgi-bin/carddisp.pl?gene=EIF4G1
CYP2B6	Cytochrome P450 Family 2 Subfamily B Member 6	Protein Coding	48	GC19P040991	18.40908432	https://www.genecards.org/cgi-bin/carddisp.pl?gene=CYP2B6
EPRS1	Glutamyl-Prolyl-TRNA Synthetase 1	Protein Coding	37	GC01M219969	18.34876251	https://www.genecards.org/cgi-bin/carddisp.pl?gene=EPRS1
CHUK	Component Of Inhibitor Of Nuclear Factor Kappa B Kinase Complex	Protein Coding	52	GC10M100188	18.32289314	https://www.genecards.org/cgi-bin/carddisp.pl?gene=CHUK
SERPINF2	Serpin Family F Member 2	Protein Coding	44	GC17P001742	18.27783012	https://www.genecards.org/cgi-bin/carddisp.pl?gene=SERPINF2
SERPINA6	Serpin Family A Member 6	Protein Coding	44	GC14M095823	18.27275848	https://www.genecards.org/cgi-bin/carddisp.pl?gene=SERPINA6
PARP1	Poly(ADP-Ribose) Polymerase 1	Protein Coding	50	GC01M226360	18.22401428	https://www.genecards.org/cgi-bin/carddisp.pl?gene=PARP1
MIR30D	MicroRNA 30d	RNA Gene	17	GC08M134804	18.22224998	https://www.genecards.org/cgi-bin/carddisp.pl?gene=MIR30D
AGER	Advanced Glycosylation End-Product Specific Receptor	Protein Coding	45	GC06M032180	18.18928909	https://www.genecards.org/cgi-bin/carddisp.pl?gene=AGER
MIR483	MicroRNA 483	RNA Gene	18	GC11M002219	18.14042473	https://www.genecards.org/cgi-bin/carddisp.pl?gene=MIR483
DKK1	Dickkopf WNT Signaling Pathway Inhibitor 1	Protein Coding	45	GC10P052314	18.08655548	https://www.genecards.org/cgi-bin/carddisp.pl?gene=DKK1
S100A9	S100 Calcium Binding Protein A9	Protein Coding	43	GC01P153357	18.07329178	https://www.genecards.org/cgi-bin/carddisp.pl?gene=S100A9
SDCCAG8	SHH Signaling And Ciliogenesis Regulator SDCCAG8	Protein Coding	43	GC01P243255	18.03864479	https://www.genecards.org/cgi-bin/carddisp.pl?gene=SDCCAG8
EZH2	Enhancer Of Zeste 2 Polycomb Repressive Complex 2 Subunit	Protein Coding	54	GC07M148807	17.97975922	https://www.genecards.org/cgi-bin/carddisp.pl?gene=EZH2
LRPPRC	Leucine Rich Pentatricopeptide Repeat Containing	Protein Coding	43	GC02M043850	17.92406082	https://www.genecards.org/cgi-bin/carddisp.pl?gene=LRPPRC
MIR205	MicroRNA 205	RNA Gene	20	GC01P209493	17.92008209	https://www.genecards.org/cgi-bin/carddisp.pl?gene=MIR205
CCL3	C-C Motif Chemokine Ligand 3	Protein Coding	40	GC17M036088	17.90954018	https://www.genecards.org/cgi-bin/carddisp.pl?gene=CCL3
SP1	Sp1 Transcription Factor	Protein Coding	45	GC12P053380	17.85421753	https://www.genecards.org/cgi-bin/carddisp.pl?gene=SP1
CEBPB	CCAAT Enhancer Binding Protein Beta	Protein Coding	44	GC20P050190	17.85057068	https://www.genecards.org/cgi-bin/carddisp.pl?gene=CEBPB
SOST	Sclerostin	Protein Coding	44	GC17M043753	17.82912827	https://www.genecards.org/cgi-bin/carddisp.pl?gene=SOST
CSF1	Colony Stimulating Factor 1	Protein Coding	44	GC01P109911	17.75559616	https://www.genecards.org/cgi-bin/carddisp.pl?gene=CSF1
NDUFS8	NADH:Ubiquinone Oxidoreductase Core Subunit S8	Protein Coding	46	GC11P068030	17.73937988	https://www.genecards.org/cgi-bin/carddisp.pl?gene=NDUFS8
FGFR4	Fibroblast Growth Factor Receptor 4	Protein Coding	52	GC05P177086	17.73563576	https://www.genecards.org/cgi-bin/carddisp.pl?gene=FGFR4
DRD2	Dopamine Receptor D2	Protein Coding	51	GC11M113409	17.66122055	https://www.genecards.org/cgi-bin/carddisp.pl?gene=DRD2
TMPRSS6	Transmembrane Serine Protease 6	Protein Coding	44	GC22M037066	17.62794876	https://www.genecards.org/cgi-bin/carddisp.pl?gene=TMPRSS6
RAC1	Rac Family Small GTPase 1	Protein Coding	50	GC07P006377	17.59141159	https://www.genecards.org/cgi-bin/carddisp.pl?gene=RAC1
HSD11B1	Hydroxysteroid 11-Beta Dehydrogenase 1	Protein Coding	50	GC01P209686	17.57976151	https://www.genecards.org/cgi-bin/carddisp.pl?gene=HSD11B1
RPS27A	Ribosomal Protein S27a	Protein Coding	44	GC02P055231	17.52258873	https://www.genecards.org/cgi-bin/carddisp.pl?gene=RPS27A
UCP1	Uncoupling Protein 1	Protein Coding	45	GC04M140559	17.26399612	https://www.genecards.org/cgi-bin/carddisp.pl?gene=UCP1
PIK3CD	Phosphatidylinositol-4,5-Bisphosphate 3-Kinase Catalytic Subunit Delta	Protein Coding	54	GC01P009629	17.26249123	https://www.genecards.org/cgi-bin/carddisp.pl?gene=PIK3CD
CCR1	C-C Motif Chemokine Receptor 1	Protein Coding	45	GC03M046218	17.22773552	https://www.genecards.org/cgi-bin/carddisp.pl?gene=CCR1
NPC1L1	NPC1 Like Intracellular Cholesterol Transporter 1	Protein Coding	44	GC07M044512	17.19471741	https://www.genecards.org/cgi-bin/carddisp.pl?gene=NPC1L1
MIR27B	MicroRNA 27b	RNA Gene	20	GC09P095097	17.17765427	https://www.genecards.org/cgi-bin/carddisp.pl?gene=MIR27B
NR0B2	Nuclear Receptor Subfamily 0 Group B Member 2	Protein Coding	44	GC01M026922	17.08059311	https://www.genecards.org/cgi-bin/carddisp.pl?gene=NR0B2
IFNL3	Interferon Lambda 3	Protein Coding	35	GC19M039243	17.02130127	https://www.genecards.org/cgi-bin/carddisp.pl?gene=IFNL3
LOC110806263	TERT 5' Regulatory Region	Biological Region	2	GC05P001294	16.94519043	https://www.genecards.org/cgi-bin/carddisp.pl?gene=LOC110806263
MIR214	MicroRNA 214	RNA Gene	20	GC01M172234	16.94231033	https://www.genecards.org/cgi-bin/carddisp.pl?gene=MIR214
NR1I3	Nuclear Receptor Subfamily 1 Group I Member 3	Protein Coding	45	GC01M161229	16.8729744	https://www.genecards.org/cgi-bin/carddisp.pl?gene=NR1I3
AHR	Aryl Hydrocarbon Receptor	Protein Coding	49	GC07P016916	16.86351013	https://www.genecards.org/cgi-bin/carddisp.pl?gene=AHR
KL	Klotho	Protein Coding	46	GC13P033016	16.86110878	https://www.genecards.org/cgi-bin/carddisp.pl?gene=KL
PRL	Prolactin	Protein Coding	44	GC06M022287	16.85406494	https://www.genecards.org/cgi-bin/carddisp.pl?gene=PRL
GSR	Glutathione-Disulfide Reductase	Protein Coding	50	GC08M030678	16.76444817	https://www.genecards.org/cgi-bin/carddisp.pl?gene=GSR
SUMF1	Sulfatase Modifying Factor 1	Protein Coding	43	GC03M003700	16.68222046	https://www.genecards.org/cgi-bin/carddisp.pl?gene=SUMF1
SERPINH1	Serpin Family H Member 1	Protein Coding	46	GC11P075562	16.63241386	https://www.genecards.org/cgi-bin/carddisp.pl?gene=SERPINH1
NDUFA6	NADH:Ubiquinone Oxidoreductase Subunit A6	Protein Coding	45	GC22M042085	16.63097191	https://www.genecards.org/cgi-bin/carddisp.pl?gene=NDUFA6
ABCC3	ATP Binding Cassette Subfamily C Member 3	Protein Coding	47	GC17P050634	16.56124687	https://www.genecards.org/cgi-bin/carddisp.pl?gene=ABCC3
LTBP3	Latent Transforming Growth Factor Beta Binding Protein 3	Protein Coding	42	GC11M065538	16.55593491	https://www.genecards.org/cgi-bin/carddisp.pl?gene=LTBP3
HTR2A	5-Hydroxytryptamine Receptor 2A	Protein Coding	48	GC13M046831	16.53968048	https://www.genecards.org/cgi-bin/carddisp.pl?gene=HTR2A
GPX1	Glutathione Peroxidase 1	Protein Coding	48	GC03M049486	16.5343132	https://www.genecards.org/cgi-bin/carddisp.pl?gene=GPX1
AKT3	AKT Serine/Threonine Kinase 3	Protein Coding	54	GC01M243488	16.53372192	https://www.genecards.org/cgi-bin/carddisp.pl?gene=AKT3
BLVRA	Biliverdin Reductase A	Protein Coding	45	GC07P043758	16.52719688	https://www.genecards.org/cgi-bin/carddisp.pl?gene=BLVRA
DNMT3A	DNA Methyltransferase 3 Alpha	Protein Coding	52	GC02M025228	16.47757721	https://www.genecards.org/cgi-bin/carddisp.pl?gene=DNMT3A
NDUFA10	NADH:Ubiquinone Oxidoreductase Subunit A10	Protein Coding	45	GC02M239893	16.45397758	https://www.genecards.org/cgi-bin/carddisp.pl?gene=NDUFA10

MIR9-1	MicroRNA 9-1	RNA Gene	20	GC01M156420	16.45192528	https://www.genecards.org/cgi-bin/carddisp.pl?gene=MIR9-1
EPHX2	Epoxide Hydrolase 2	Protein Coding	48	GC08P027490	16.41978073	https://www.genecards.org/cgi-bin/carddisp.pl?gene=EPHX2
CD163	CD163 Molecule	Protein Coding	43	GC12M007471	16.40397072	https://www.genecards.org/cgi-bin/carddisp.pl?gene=CD163
COX4I1	Cytochrome C Oxidase Subunit 4I1	Protein Coding	47	GC16P085798	16.39952278	https://www.genecards.org/cgi-bin/carddisp.pl?gene=COX4I1
HSP90AA1	Heat Shock Protein 90 Alpha Family Class A Member 1	Protein Coding	48	GC14M102080	16.3786068	https://www.genecards.org/cgi-bin/carddisp.pl?gene=HSP90AA1
IRGM	Immunity Related GTPase M	Protein Coding	38	GC05P150846	16.36742401	https://www.genecards.org/cgi-bin/carddisp.pl?gene=IRGM
IRF1	Interferon Regulatory Factor 1	Protein Coding	47	GC05M132440	16.34394264	https://www.genecards.org/cgi-bin/carddisp.pl?gene=IRF1
HMGB1	High Mobility Group Box 1	Protein Coding	45	GC13M030456	16.3422699	https://www.genecards.org/cgi-bin/carddisp.pl?gene=HMGB1
DYSF	Dysferlin	Protein Coding	44	GC02P071453	16.32235718	https://www.genecards.org/cgi-bin/carddisp.pl?gene=DYSF
DNASE1	Deoxyribonuclease 1	Protein Coding	43	GC16P003611	16.29515839	https://www.genecards.org/cgi-bin/carddisp.pl?gene=DNASE1
NDUFB8	NADH:Ubiquinone Oxidoreductase Subunit B8	Protein Coding	43	GC10M100523	16.28831863	https://www.genecards.org/cgi-bin/carddisp.pl?gene=NDUFB8
FGB	Fibrinogen Beta Chain	Protein Coding	47	GC04P154572	16.28487778	https://www.genecards.org/cgi-bin/carddisp.pl?gene=FGB
MMP13	Matrix Metallopeptidase 13	Protein Coding	51	GC11M102942	16.26388931	https://www.genecards.org/cgi-bin/carddisp.pl?gene=MMP13
CXCR3	C-X-C Motif Chemokine Receptor 3	Protein Coding	45	GC0XM071615	16.24189758	https://www.genecards.org/cgi-bin/carddisp.pl?gene=CXCR3
FLI1	Fli-1 Proto-Oncogene, ETS Transcription Factor	Protein Coding	49	GC11P128686	16.21395111	https://www.genecards.org/cgi-bin/carddisp.pl?gene=FLI1
LMBRD1	LMBR1 Domain Containing 1	Protein Coding	39	GC06M069675	16.20565033	https://www.genecards.org/cgi-bin/carddisp.pl?gene=LMBRD1
SERPINF1	Serpin Family F Member 1	Protein Coding	45	GC17P001761	16.18712997	https://www.genecards.org/cgi-bin/carddisp.pl?gene=SERPINF1
NQO1	NAD(P)H Quinone Dehydrogenase 1	Protein Coding	50	GC16M069706	16.1689415	https://www.genecards.org/cgi-bin/carddisp.pl?gene=NQO1
PRKCA	Protein Kinase C Alpha	Protein Coding	52	GC17P066302	16.13891029	https://www.genecards.org/cgi-bin/carddisp.pl?gene=PRKCA
UGT1A5	UDP Glucuronosyltransferase Family 1 Member A5	Protein Coding	34	GC02P233712	16.1357193	https://www.genecards.org/cgi-bin/carddisp.pl?gene=UGT1A5
ANGPTL3	Angiopoietin Like 3	Protein Coding	46	GC01P062597	16.09730911	https://www.genecards.org/cgi-bin/carddisp.pl?gene=ANGPTL3
NDUFB11	NADH:Ubiquinone Oxidoreductase Subunit B11	Protein Coding	40	GC0XM047142	16.08590698	https://www.genecards.org/cgi-bin/carddisp.pl?gene=NDUFB11
NDUFS6	NADH:Ubiquinone Oxidoreductase Subunit S6	Protein Coding	45	GC05P001801	16.07621384	https://www.genecards.org/cgi-bin/carddisp.pl?gene=NDUFS6
BIRC5	Baculoviral IAP Repeat Containing 5	Protein Coding	47	GC17P078214	16.07241058	https://www.genecards.org/cgi-bin/carddisp.pl?gene=BIRC5
AKR1A1	Aldo-Keto Reductase Family 1 Member	Protein Coding	45	GC01P045550	16.06908417	https://www.genecards.org/cgi-bin/carddisp.pl?gene=AKR1A1
MERTK	MER Proto-Oncogene, Tyrosine Kinase	Protein Coding	51	GC02P111898	16.00081635	https://www.genecards.org/cgi-bin/carddisp.pl?gene=MERTK
MAPK10	Mitogen-Activated Protein Kinase 10	Protein Coding	52	GC04M085990	15.95767784	https://www.genecards.org/cgi-bin/carddisp.pl?gene=MAPK10
PRSS1	Serine Protease 1	Protein Coding	47	GC07P145775	15.9276638	https://www.genecards.org/cgi-bin/carddisp.pl?gene=PRSS1
CCK	Cholecystokinin	Protein Coding	43	GC03M042274	15.90043545	https://www.genecards.org/cgi-bin/carddisp.pl?gene=CCK
HSPG2	Heparan Sulfate Proteoglycan 2	Protein Coding	46	GC01M021822	15.88156986	https://www.genecards.org/cgi-bin/carddisp.pl?gene=HSPG2
GDF15	Growth Differentiation Factor 15	Protein Coding	43	GC19P026656	15.87571144	https://www.genecards.org/cgi-bin/carddisp.pl?gene=GDF15
CDC42	Cell Division Cycle 42	Protein Coding	51	GC01P022112	15.85445023	https://www.genecards.org/cgi-bin/carddisp.pl?gene=CDC42
MAOA	Monoamine Oxidase A	Protein Coding	51	GC0XP043654	15.81402779	https://www.genecards.org/cgi-bin/carddisp.pl?gene=MAOA
NDUFA12	NADH:Ubiquinone Oxidoreductase Subunit A12	Protein Coding	45	GC12M094898	15.79312706	https://www.genecards.org/cgi-bin/carddisp.pl?gene=NDUFA12
GSS	Glutathione Synthetase	Protein Coding	47	GC20M034928	15.64771557	https://www.genecards.org/cgi-bin/carddisp.pl?gene=GSS
COX6B1	Cytochrome C Oxidase Subunit 6B1	Protein Coding	43	GC19P040358	15.64626884	https://www.genecards.org/cgi-bin/carddisp.pl?gene=COX6B1
NDUFB3	NADH:Ubiquinone Oxidoreductase Subunit B3	Protein Coding	43	GC02P201071	15.64473438	https://www.genecards.org/cgi-bin/carddisp.pl?gene=NDUFB3
HDAC9	Histone Deacetylase 9	Protein Coding	47	GC07P018086	15.61901283	https://www.genecards.org/cgi-bin/carddisp.pl?gene=HDAC9
ATP1A1	ATPase Na+/K+ Transporting Subunit Alpha 1	Protein Coding	51	GC01P116372	15.59727097	https://www.genecards.org/cgi-bin/carddisp.pl?gene=ATP1A1
ABCG1	ATP Binding Cassette Subfamily G Member 1	Protein Coding	44	GC21P042199	15.56394768	https://www.genecards.org/cgi-bin/carddisp.pl?gene=ABCG1
GOT2	Glutamic-Oxaloacetic Transaminase 2	Protein Coding	47	GC16M058707	15.54222679	https://www.genecards.org/cgi-bin/carddisp.pl?gene=GOT2
CHI3L1	Chitinase 3 Like 1	Protein Coding	44	GC01M203148	15.52296543	https://www.genecards.org/cgi-bin/carddisp.pl?gene=CHI3L1
MATR3	Matrin 3	Protein Coding	43	GC05P139274	15.51981354	https://www.genecards.org/cgi-bin/carddisp.pl?gene=MATR3
CD55	CD55 Molecule (Cromer Blood Group)	Protein Coding	48	GC01P207321	15.50701332	https://www.genecards.org/cgi-bin/carddisp.pl?gene=CD55
CCL20	C-C Motif Chemokine Ligand 20	Protein Coding	44	GC02P227814	15.50111008	https://www.genecards.org/cgi-bin/carddisp.pl?gene=CCL20
COX8A	Cytochrome C Oxidase Subunit 8A	Protein Coding	41	GC11P063994	15.4643364	https://www.genecards.org/cgi-bin/carddisp.pl?gene=COX8A
UQCRC2	Ubiquinol-Cytochrome C Reductase Core Protein 2	Protein Coding	47	GC16P021963	15.43040752	https://www.genecards.org/cgi-bin/carddisp.pl?gene=UQCRC2
THBS1	Thrombospondin 1	Protein Coding	45	GC15P039581	15.41858387	https://www.genecards.org/cgi-bin/carddisp.pl?gene=THBS1
ETS1	ETS Proto-Oncogene 1, Transcription	Protein Coding	49	GC11M128458	15.40030289	https://www.genecards.org/cgi-bin/carddisp.pl?gene=ETS1
PEPD	Peptidase D	Protein Coding	46	GC19M033386	15.37115002	https://www.genecards.org/cgi-bin/carddisp.pl?gene=PEPD
ACSM2B	Acyl-CoA Synthetase Medium Chain Family Member 2B	Protein Coding	36	GC16M020547	15.28150845	https://www.genecards.org/cgi-bin/carddisp.pl?gene=ACSM2B
EIF2AK2	Eukaryotic Translation Initiation Factor 2 Alpha Kinase 2	Protein Coding	46	GC02M037099	15.26604939	https://www.genecards.org/cgi-bin/carddisp.pl?gene=EIF2AK2
MIR149	MicroRNA 149	RNA Gene	20	GC02P240456	15.20406532	https://www.genecards.org/cgi-bin/carddisp.pl?gene=MIR149
PLCB1	Phospholipase C Beta 1	Protein Coding	49	GC20P008061	15.19641972	https://www.genecards.org/cgi-bin/carddisp.pl?gene=PLCB1
FOXO1	Forkhead Box O1	Protein Coding	48	GC13M040555	15.14049435	https://www.genecards.org/cgi-bin/carddisp.pl?gene=FOXO1
CA2	Carbonic Anhydrase 2	Protein Coding	52	GC08P085463	15.12652779	https://www.genecards.org/cgi-bin/carddisp.pl?gene=CA2
DDX3X	DEAD-Box Helicase 3 X-Linked	Protein Coding	48	GC0XP041333	15.11061478	https://www.genecards.org/cgi-bin/carddisp.pl?gene=DDX3X
SNAI2	Snail Family Transcriptional Repressor 2	Protein Coding	45	GC08M048917	15.10995197	https://www.genecards.org/cgi-bin/carddisp.pl?gene=SNAI2
PLIN1	Perilipin 1	Protein Coding	45	GC15M089664	15.08190918	https://www.genecards.org/cgi-bin/carddisp.pl?gene=PLIN1
ALOX12	Arachidonate 12-Lipoxygenase, 12S Type	Protein Coding	44	GC17P006995	15.08088303	https://www.genecards.org/cgi-bin/carddisp.pl?gene=ALOX12
FARSA	Phenylalanyl-TRNA Synthetase Subunit Alpha	Protein Coding	41	GC19M012922	15.07990646	https://www.genecards.org/cgi-bin/carddisp.pl?gene=FARSA
RPS6KB1	Ribosomal Protein S6 Kinase B1	Protein Coding	50	GC17P059893	15.05170727	https://www.genecards.org/cgi-bin/carddisp.pl?gene=RPS6KB1
DICER1	Dicer 1, Ribonuclease III	Protein Coding	48	GC14M095086	15.03832054	https://www.genecards.org/cgi-bin/carddisp.pl?gene=DICER1
BMPR1A	Bone Morphogenetic Protein Receptor Type 1A	Protein Coding	51	GC10P086756	15.0340662	https://www.genecards.org/cgi-bin/carddisp.pl?gene=BMPR1A
CNTNAP2	Contactin Associated Protein 2	Protein Coding	44	GC07P146116	15.01487637	https://www.genecards.org/cgi-bin/carddisp.pl?gene=CNTNAP2
CPOX	Coproporphyrinogen Oxidase	Protein Coding	44	GC03M098576	14.96734905	https://www.genecards.org/cgi-bin/carddisp.pl?gene=CPOX
DNAH11	Dynein Axonemal Heavy Chain 11	Protein Coding	42	GC07P021543	14.86241722	https://www.genecards.org/cgi-bin/carddisp.pl?gene=DNAH11
PIK3R2	Phosphoinositide-3-Kinase Regulatory Subunit 2	Protein Coding	50	GC19P018153	14.8380537	https://www.genecards.org/cgi-bin/carddisp.pl?gene=PIK3R2
IRF8	Interferon Regulatory Factor 8	Protein Coding	47	GC16P085898	14.79246044	https://www.genecards.org/cgi-bin/carddisp.pl?gene=IRF8
CYP17A1	Cytochrome P450 Family 17 Subfamily A Member 1	Protein Coding	50	GC10M102830	14.77322292	https://www.genecards.org/cgi-bin/carddisp.pl?gene=CYP17A1
PLAUR	Plasminogen Activator, Urokinase	Protein Coding	45	GC19M043646	14.73099422	https://www.genecards.org/cgi-bin/carddisp.pl?gene=PLAUR
SDC1	Syndecan 1	Protein Coding	44	GC02M020200	14.6667614	https://www.genecards.org/cgi-bin/carddisp.pl?gene=SDC1
PLTP	Phospholipid Transfer Protein	Protein Coding	44	GC20M045898	14.64484978	https://www.genecards.org/cgi-bin/carddisp.pl?gene=PLTP
POR	Cytochrome P450 Oxidoreductase	Protein Coding	50	GC07P075899	14.60950947	https://www.genecards.org/cgi-bin/carddisp.pl?gene=POR
ADRB3	Adrenoceptor Beta 3	Protein Coding	46	GC08M037962	14.55301666	https://www.genecards.org/cgi-bin/carddisp.pl?gene=ADRB3
ALAD	Aminolevulinate Dehydratase	Protein Coding	47	GC09M113386	14.53653908	https://www.genecards.org/cgi-bin/carddisp.pl?gene=ALAD
TGM2	Transglutaminase 2	Protein Coding	48	GC20M038127	14.51640129	https://www.genecards.org/cgi-bin/carddisp.pl?gene=TGM2
STAR	Steroidogenic Acute Regulatory Protein	Protein Coding	47	GC08M038145	14.50080585	https://www.genecards.org/cgi-bin/carddisp.pl?gene=STAR
SMAD7	SMAD Family Member 7	Protein Coding	44	GC18M048919	14.4142046	https://www.genecards.org/cgi-bin/carddisp.pl?gene=SMAD7
MYH14	Myosin Heavy Chain 14	Protein Coding	46	GC19P050192	14.4097271	https://www.genecards.org/cgi-bin/carddisp.pl?gene=MYH14
MT-TT	Mitochondrially Encoded TRNA-Thr	RNA Gene	15	GCMTP015890	14.38364792	https://www.genecards.org/cgi-bin/carddisp.pl?gene=MT-TT
KEAP1	Kelch Like ECH Associated Protein 1	Protein Coding	48	GC19M010486	14.35438061	https://www.genecards.org/cgi-bin/carddisp.pl?gene=KEAP1
NOS1	Nitric Oxide Synthase 1	Protein Coding	50	GC12M117208	14.35239029	https://www.genecards.org/cgi-bin/carddisp.pl?gene=NOS1
PPP3CA	Protein Phosphatase 3 Catalytic Subunit Alpha	Protein Coding	54	GC04M101024	14.35124397	https://www.genecards.org/cgi-bin/carddisp.pl?gene=PPP3CA
CD68	CD68 Molecule	Protein Coding	40	GC17P007579	14.33781528	https://www.genecards.org/cgi-bin/carddisp.pl?gene=CD68
GOLM1	Golgi Membrane Protein 1	Protein Coding	39	GC09M086026	14.30690002	https://www.genecards.org/cgi-bin/carddisp.pl?gene=GOLM1
KLF6	Kruppel Like Factor 6	Protein Coding	44	GC10M003779	14.259799	https://www.genecards.org/cgi-bin/carddisp.pl?gene=KLF6
TPM2	Tropomyosin 2	Protein Coding	46	GC09M035672	14.24700546	https://www.genecards.org/cgi-bin/carddisp.pl?gene=TPM2
DBH	Dopamine Beta-Hydroxylase	Protein Coding	51	GC09P133636	14.19199371	https://www.genecards.org/cgi-bin/carddisp.pl?gene=DBH
CES2	Carboxylesterase 2	Protein Coding	44	GC16P066934	14.17992878	https://www.genecards.org/cgi-bin/carddisp.pl?gene=CES2

HCFC1	Host Cell Factor C1	Protein Coding	47	GC0XM153947	14.17186546	https://www.genecards.org/cgi-bin/carddisp.pl?gene=HCFC1
MIR15B	MicroRNA 15b	RNA Gene	19	GC03P160404	14.16932392	https://www.genecards.org/cgi-bin/carddisp.pl?gene=MIR15B
LEPQTL1	Leptin, Serum Levels Of	Genetic Locus	2	GC02U903086	14.10124874	https://www.genecards.org/cgi-bin/carddisp.pl?gene=LEPQTL1
IL6ST	Interleukin 6 Cytokine Family Signal Transducer	Protein Coding	47	GC05M055935	14.09537411	https://www.genecards.org/cgi-bin/carddisp.pl?gene=IL6ST
UBD	Ubiquitin D	Protein Coding	39	GC06M046741	14.06287384	https://www.genecards.org/cgi-bin/carddisp.pl?gene=UBD
PRDX1	Peroxiredoxin 1	Protein Coding	50	GC01M045511	14.05330944	https://www.genecards.org/cgi-bin/carddisp.pl?gene=PRDX1
NEAT1	Nuclear Paraspeckle Assembly Transcript	RNA Gene	23	GC11P066222	14.03329945	https://www.genecards.org/cgi-bin/carddisp.pl?gene=NEAT1
SULT2A1	Sulfotransferase Family 2A Member 1	Protein Coding	45	GC19M047870	13.93974113	https://www.genecards.org/cgi-bin/carddisp.pl?gene=SULT2A1
MIR24-1	MicroRNA 24-1	RNA Gene	19	GC09P095086	13.89949894	https://www.genecards.org/cgi-bin/carddisp.pl?gene=MIR24-1
HDAC1	Histone Deacetylase 1	Protein Coding	50	GC01P032292	13.898386	https://www.genecards.org/cgi-bin/carddisp.pl?gene=HDAC1
FDFT1	Farnesyl-Diphosphate Farnesyltransferase	Protein Coding	46	GC08P011795	13.89482498	https://www.genecards.org/cgi-bin/carddisp.pl?gene=FDFT1
CYP4F2	Cytochrome P450 Family 4 Subfamily F Member 2	Protein Coding	45	GC19M015878	13.87469292	https://www.genecards.org/cgi-bin/carddisp.pl?gene=CYP4F2
MIR154	MicroRNA 154	RNA Gene	18	GC14P106303	13.86999893	https://www.genecards.org/cgi-bin/carddisp.pl?gene=MIR154
RPS6KA3	Ribosomal Protein S6 Kinase A3	Protein Coding	53	GC0XM020149	13.83438778	https://www.genecards.org/cgi-bin/carddisp.pl?gene=RPS6KA3
CD59	CD59 Molecule (CD59 Blood Group)	Protein Coding	47	GC11M033704	13.81402588	https://www.genecards.org/cgi-bin/carddisp.pl?gene=CD59
LNCARSR	LncRNA Regulator Of Akt Signaling Associated With HCC And RCC	RNA Gene	11	GC09M079507	13.79727173	https://www.genecards.org/cgi-bin/carddisp.pl?gene=LNCARSR
CASP7	Caspase 7	Protein Coding	50	GC10P113679	13.77668762	https://www.genecards.org/cgi-bin/carddisp.pl?gene=CASP7
SHC1	SHC Adaptor Protein 1	Protein Coding	46	GC01M154962	13.76587296	https://www.genecards.org/cgi-bin/carddisp.pl?gene=SHC1
HDAC8	Histone Deacetylase 8	Protein Coding	48	GC0XM072329	13.7309866	https://www.genecards.org/cgi-bin/carddisp.pl?gene=HDAC8
STAT5A	Signal Transducer And Activator Of Transcription 5A	Protein Coding	47	GC17P042287	13.69988918	https://www.genecards.org/cgi-bin/carddisp.pl?gene=STAT5A
ANGPTL8	Angiopoietin Like 8	Protein Coding	29	GC19P011238	13.67861843	https://www.genecards.org/cgi-bin/carddisp.pl?gene=ANGPTL8
MIR331	MicroRNA 331	RNA Gene	18	GC12P095308	13.670681	https://www.genecards.org/cgi-bin/carddisp.pl?gene=MIR331
NDUFA11	NADH:Ubiquinone Oxidoreductase Subunit A11	Protein Coding	38	GC19M005891	13.65572929	https://www.genecards.org/cgi-bin/carddisp.pl?gene=NDUFA11
NDUFAB1	NADH:Ubiquinone Oxidoreductase Subunit AB1	Protein Coding	41	GC16M023582	13.6364193	https://www.genecards.org/cgi-bin/carddisp.pl?gene=NDUFAB1
POSTN	Periostin	Protein Coding	44	GC13M037562	13.58043385	https://www.genecards.org/cgi-bin/carddisp.pl?gene=POSTN
SI	Sucrase-Isomaltase	Protein Coding	44	GC03M164978	13.53289604	https://www.genecards.org/cgi-bin/carddisp.pl?gene=SI
GABRG2	Gamma-Aminobutyric Acid Type A Receptor Subunit Gamma2	Protein Coding	48	GC05P162000	13.52079391	https://www.genecards.org/cgi-bin/carddisp.pl?gene=GABRG2
NDUFA9	NADH:Ubiquinone Oxidoreductase Subunit A9	Protein Coding	44	GC12P004649	13.46017838	https://www.genecards.org/cgi-bin/carddisp.pl?gene=NDUFA9
CCL4	C-C Motif Chemokine Ligand 4	Protein Coding	41	GC17P036103	13.45113277	https://www.genecards.org/cgi-bin/carddisp.pl?gene=CCL4
OSM	Oncostatin M	Protein Coding	43	GC22M030262	13.45028496	https://www.genecards.org/cgi-bin/carddisp.pl?gene=OSM
TXNRD2	Thioredoxin Reductase 2	Protein Coding	48	GC22M019863	13.43647003	https://www.genecards.org/cgi-bin/carddisp.pl?gene=TXNRD2
IRF3	Interferon Regulatory Factor 3	Protein Coding	47	GC19M049659	13.42432785	https://www.genecards.org/cgi-bin/carddisp.pl?gene=IRF3
NDUFA2	NADH:Ubiquinone Oxidoreductase Subunit A2	Protein Coding	43	GC05M140653	13.41955948	https://www.genecards.org/cgi-bin/carddisp.pl?gene=NDUFA2
TLR1	Toll Like Receptor 1	Protein Coding	48	GC04M038793	13.35544014	https://www.genecards.org/cgi-bin/carddisp.pl?gene=TLR1
TXN	Thioredoxin	Protein Coding	46	GC09M110243	13.29109383	https://www.genecards.org/cgi-bin/carddisp.pl?gene=TXN
IL33	Interleukin 33	Protein Coding	40	GC09P006206	13.25358009	https://www.genecards.org/cgi-bin/carddisp.pl?gene=IL33
CYC1	Cytochrome C1	Protein Coding	46	GC08P144095	13.20482063	https://www.genecards.org/cgi-bin/carddisp.pl?gene=CYC1
FYN	FYN Proto-Oncogene, Src Family Tyrosine Kinase	Protein Coding	48	GC06M111660	13.20200253	https://www.genecards.org/cgi-bin/carddisp.pl?gene=FYN
GPC4	Glypican 4	Protein Coding	46	GC0XM133300	13.19788933	https://www.genecards.org/cgi-bin/carddisp.pl?gene=GPC4
CANX	Calnexin	Protein Coding	45	GC05P179678	13.18466759	https://www.genecards.org/cgi-bin/carddisp.pl?gene=CANX
IAH1	Isoamyl Acetate Hydrolyzing Esterase 1 (Putative)	Protein Coding	34	GC02P009473	13.18420792	https://www.genecards.org/cgi-bin/carddisp.pl?gene=IAH1
TSPO	Translocator Protein	Protein Coding	44	GC22P043151	13.14738941	https://www.genecards.org/cgi-bin/carddisp.pl?gene=TSPO
WNT3A	Wnt Family Member 3A	Protein Coding	48	GC01P228466	13.12548256	https://www.genecards.org/cgi-bin/carddisp.pl?gene=WNT3A
RB1CC1	RB1 Inducible Coiled-Coil 1	Protein Coding	43	GC08M052622	13.06217957	https://www.genecards.org/cgi-bin/carddisp.pl?gene=RB1CC1
CRH	Corticotropin Releasing Hormone	Protein Coding	44	GC08M066176	13.05428314	https://www.genecards.org/cgi-bin/carddisp.pl?gene=CRH
PIK3CB	Phosphatidylinositol-4,5-Bisphosphate 3-Kinase Catalytic Subunit Beta	Protein Coding	50	GC03M138652	13.05126762	https://www.genecards.org/cgi-bin/carddisp.pl?gene=PIK3CB
IL17RA	Interleukin 17 Receptor A	Protein Coding	45	GC22P017116	13.02085209	https://www.genecards.org/cgi-bin/carddisp.pl?gene=IL17RA
ATF6	Activating Transcription Factor 6	Protein Coding	48	GC01P161766	13.00949764	https://www.genecards.org/cgi-bin/carddisp.pl?gene=ATF6
ERLIN1	ER Lipid Raft Associated 1	Protein Coding	43	GC10M100150	12.97800636	https://www.genecards.org/cgi-bin/carddisp.pl?gene=ERLIN1
MIR125B1	MicroRNA 125b-1	RNA Gene	21	GC11M122100	12.97618294	https://www.genecards.org/cgi-bin/carddisp.pl?gene=MIR125B1
OGG1	8-Oxoguanine DNA Glycosylase	Protein Coding	47	GC03P009751	12.94499207	https://www.genecards.org/cgi-bin/carddisp.pl?gene=OGG1
HNRNPK	Heterogeneous Nuclear Ribonucleoprotein	Protein Coding	47	GC09M086093	12.92714214	https://www.genecards.org/cgi-bin/carddisp.pl?gene=HNRNPK
LBP	Lipopolysaccharide Binding Protein	Protein Coding	44	GC20P038346	12.82848358	https://www.genecards.org/cgi-bin/carddisp.pl?gene=LBP
APPL1	Adaptor Protein, Phosphotyrosine Interacting With PH Domain And Leucine	Protein Coding	46	GC03P057227	12.78715897	https://www.genecards.org/cgi-bin/carddisp.pl?gene=APPL1
PHB	Prohibitin	Protein Coding	48	GC17M049404	12.78440094	https://www.genecards.org/cgi-bin/carddisp.pl?gene=PHB
CLTC	Clathrin Heavy Chain	Protein Coding	47	GC17P059619	12.76284981	https://www.genecards.org/cgi-bin/carddisp.pl?gene=CLTC
CCR2	C-C Motif Chemokine Receptor 2	Protein Coding	45	GC03P046356	12.69025803	https://www.genecards.org/cgi-bin/carddisp.pl?gene=CCR2
MBOAT7	Membrane Bound O-Acyltransferase Domain Containing 7	Protein Coding	41	GC19M054173	12.66814995	https://www.genecards.org/cgi-bin/carddisp.pl?gene=MBOAT7
GCLC	Glutamate-Cysteine Ligase Catalytic	Protein Coding	44	GC06M053497	12.65995121	https://www.genecards.org/cgi-bin/carddisp.pl?gene=GCLC
PKM	Pyruvate Kinase M1/2	Protein Coding	46	GC15M072199	12.65349388	https://www.genecards.org/cgi-bin/carddisp.pl?gene=PKM
CIDEC	Cell Death Inducing DFFA Like Effector C	Protein Coding	42	GC03M009866	12.6244297	https://www.genecards.org/cgi-bin/carddisp.pl?gene=CIDEC
TPM3	Tropomyosin 3	Protein Coding	47	GC01M154127	12.62048721	https://www.genecards.org/cgi-bin/carddisp.pl?gene=TPM3
IARS1	Isoleucyl-TRNA Synthetase 1	Protein Coding	36	GC09M092211	12.5953846	https://www.genecards.org/cgi-bin/carddisp.pl?gene=IARS1
ATP5F1E	ATP Synthase F1 Subunit Epsilon	Protein Coding	32	GC20M059026	12.58127308	https://www.genecards.org/cgi-bin/carddisp.pl?gene=ATP5F1E
PTPN1	Protein Tyrosine Phosphatase Non-Receptor Type 1	Protein Coding	51	GC20P050510	12.57707119	https://www.genecards.org/cgi-bin/carddisp.pl?gene=PTPN1
FTO	FTO Alpha-Ketoglutarate Dependent Dioxygenase	Protein Coding	45	GC16P053737	12.57312965	https://www.genecards.org/cgi-bin/carddisp.pl?gene=FTO
ANXA2	Annexin A2	Protein Coding	49	GC15M060347	12.57032967	https://www.genecards.org/cgi-bin/carddisp.pl?gene=ANXA2
OLR1	Oxidized Low Density Lipoprotein Receptor 1	Protein Coding	44	GC12M015685	12.54725552	https://www.genecards.org/cgi-bin/carddisp.pl?gene=OLR1
GSK3A	Glycogen Synthase Kinase 3 Alpha	Protein Coding	50	GC19M046852	12.53806496	https://www.genecards.org/cgi-bin/carddisp.pl?gene=GSK3A
COX6A2	Cytochrome C Oxidase Subunit 6A2	Protein Coding	40	GC16M031439	12.53235722	https://www.genecards.org/cgi-bin/carddisp.pl?gene=COX6A2
DRD1	Dopamine Receptor D1	Protein Coding	45	GC05M175440	12.52599335	https://www.genecards.org/cgi-bin/carddisp.pl?gene=DRD1
GPBAR1	G Protein-Coupled Bile Acid Receptor 1	Protein Coding	40	GC02P218259	12.50343513	https://www.genecards.org/cgi-bin/carddisp.pl?gene=GPBAR1
HSPA1A	Heat Shock Protein Family A (Hsp70) Member 1A	Protein Coding	44	GC06P055219	12.49759865	https://www.genecards.org/cgi-bin/carddisp.pl?gene=HSPA1A
E2F1	E2F Transcription Factor 1	Protein Coding	44	GC20M033675	12.48872185	https://www.genecards.org/cgi-bin/carddisp.pl?gene=E2F1
MIR95	MicroRNA 95	RNA Gene	16	GC04M008007	12.48134708	https://www.genecards.org/cgi-bin/carddisp.pl?gene=MIR95
ASCL1	Achaete-Scute Family BHLH Transcription Factor 1	Protein Coding	45	GC12P102957	12.46600342	https://www.genecards.org/cgi-bin/carddisp.pl?gene=ASCL1
TFEB	Transcription Factor EB	Protein Coding	43	GC06M047093	12.44377232	https://www.genecards.org/cgi-bin/carddisp.pl?gene=TFEB
ARPC1B	Actin Related Protein 2/3 Complex Subunit 1B	Protein Coding	44	GC07P099374	12.43692398	https://www.genecards.org/cgi-bin/carddisp.pl?gene=ARPC1B
DRD4	Dopamine Receptor D4	Protein Coding	47	GC11P000979	12.43097115	https://www.genecards.org/cgi-bin/carddisp.pl?gene=DRD4
IL1RAPL2	Interleukin 1 Receptor Accessory Protein Like 2	Protein Coding	38	GC0XP104566	12.4201622	https://www.genecards.org/cgi-bin/carddisp.pl?gene=IL1RAPL2
TPM1	Tropomyosin 1	Protein Coding	50	GC15P078910	12.40521622	https://www.genecards.org/cgi-bin/carddisp.pl?gene=TPM1
IL11	Interleukin 11	Protein Coding	41	GC19M055364	12.38793373	https://www.genecards.org/cgi-bin/carddisp.pl?gene=IL11
NDUFB10	NADH:Ubiquinone Oxidoreductase Subunit B10	Protein Coding	44	GC16P005573	12.35549355	https://www.genecards.org/cgi-bin/carddisp.pl?gene=NDUFB10
IGFBP2	Insulin Like Growth Factor Binding	Protein Coding	44	GC02P216632	12.32943249	https://www.genecards.org/cgi-bin/carddisp.pl?gene=IGFBP2
CFLAR	CASP8 And FADD Like Apoptosis Regulator	Protein Coding	47	GC02P201117	12.28454971	https://www.genecards.org/cgi-bin/carddisp.pl?gene=CFLAR

NTS	Neurotensin	Protein Coding	41	GC12P085876	12.27574921	https://www.genecards.org/cgi-bin/carddisp.pl?gene=NTS
DPM1	Dolichyl-Phosphate Mannosyltransferase Subunit 1, Catalytic	Protein Coding	45	GC20M050934	12.26564312	https://www.genecards.org/cgi-bin/carddisp.pl?gene=DPM1
STUB1	STIP1 Homology And U-Box Containing Protein 1	Protein Coding	46	GC16P005516	12.25460815	https://www.genecards.org/cgi-bin/carddisp.pl?gene=STUB1
XRCC4	X-Ray Repair Cross Complementing 4	Protein Coding	44	GC05P083077	12.23878098	https://www.genecards.org/cgi-bin/carddisp.pl?gene=XRCC4
RPL5	Ribosomal Protein L5	Protein Coding	48	GC01P092832	12.23239803	https://www.genecards.org/cgi-bin/carddisp.pl?gene=RPL5
CYP2J2	Cytochrome P450 Family 2 Subfamily J Member 2	Protein Coding	45	GC01M059893	12.15305138	https://www.genecards.org/cgi-bin/carddisp.pl?gene=CYP2J2
SERPINA7	Serpin Family A Member 7	Protein Coding	40	GC0XM106032	12.11055183	https://www.genecards.org/cgi-bin/carddisp.pl?gene=SERPINA7
DRD3	Dopamine Receptor D3	Protein Coding	45	GC03M114128	12.10384369	https://www.genecards.org/cgi-bin/carddisp.pl?gene=DRD3
SF3B1	Splicing Factor 3b Subunit 1	Protein Coding	45	GC02M197393	12.0990696	https://www.genecards.org/cgi-bin/carddisp.pl?gene=SF3B1
ACE2	Angiotensin Converting Enzyme 2	Protein Coding	49	GC0XM015494	12.09478569	https://www.genecards.org/cgi-bin/carddisp.pl?gene=ACE2
MDH2	Malate Dehydrogenase 2	Protein Coding	49	GC07P076048	12.08661652	https://www.genecards.org/cgi-bin/carddisp.pl?gene=MDH2
CDK8	Cyclin Dependent Kinase 8	Protein Coding	48	GC13P026254	12.06512737	https://www.genecards.org/cgi-bin/carddisp.pl?gene=CDK8
SCAP	SREBF Chaperone	Protein Coding	41	GC03M047413	12.04054737	https://www.genecards.org/cgi-bin/carddisp.pl?gene=SCAP
COL12A1	Collagen Type XII Alpha 1 Chain	Protein Coding	42	GC06M075084	11.98452568	https://www.genecards.org/cgi-bin/carddisp.pl?gene=COL12A1
PALLD	Palladin, Cytoskeletal Associated Protein	Protein Coding	44	GC04P168497	11.97765255	https://www.genecards.org/cgi-bin/carddisp.pl?gene=PALLD
MAPK9	Mitogen-Activated Protein Kinase 9	Protein Coding	49	GC05M180234	11.97732925	https://www.genecards.org/cgi-bin/carddisp.pl?gene=MAPK9
CLCN2	Chloride Voltage-Gated Channel 2	Protein Coding	47	GC03M184346	11.97595787	https://www.genecards.org/cgi-bin/carddisp.pl?gene=CLCN2
COX7A2	Cytochrome C Oxidase Subunit 7A2	Protein Coding	39	GC06M075237	11.96281242	https://www.genecards.org/cgi-bin/carddisp.pl?gene=COX7A2
SRD5A2	Steroid 5 Alpha-Reductase 2	Protein Coding	43	GC02M031522	11.95817089	https://www.genecards.org/cgi-bin/carddisp.pl?gene=SRD5A2
HUWE1	HECT, UBA And WWE Domain Containing E3 Ubiquitin Protein Ligase 1	Protein Coding	44	GC0XM053532	11.95288658	https://www.genecards.org/cgi-bin/carddisp.pl?gene=HUWE1
LPIN1	Lipin 1	Protein Coding	48	GC02P011677	11.95048332	https://www.genecards.org/cgi-bin/carddisp.pl?gene=LPIN1
SLC25A1	Solute Carrier Family 25 Member 1	Protein Coding	47	GC22M019177	11.93685532	https://www.genecards.org/cgi-bin/carddisp.pl?gene=SLC25A1
UQCRFS1	Ubiquinol-Cytochrome C Reductase, Rieske Iron-Sulfur Polypeptide 1	Protein Coding	47	GC19M029205	11.9285717	https://www.genecards.org/cgi-bin/carddisp.pl?gene=UQCRFS1
MIR187	MicroRNA 187	RNA Gene	18	GC18M035904	11.92744637	https://www.genecards.org/cgi-bin/carddisp.pl?gene=MIR187
DHCR24	24-Dehydrocholesterol Reductase	Protein Coding	46	GC01M054849	11.92619324	https://www.genecards.org/cgi-bin/carddisp.pl?gene=DHCR24
FGL1	Fibrinogen Like 1	Protein Coding	41	GC08M017864	11.92427444	https://www.genecards.org/cgi-bin/carddisp.pl?gene=FGL1
HNRNPU	Heterogeneous Nuclear Ribonucleoprotein	Protein Coding	43	GC01M244844	11.87169933	https://www.genecards.org/cgi-bin/carddisp.pl?gene=HNRNPU
VTN	Vitronectin	Protein Coding	44	GC17M031079	11.84849072	https://www.genecards.org/cgi-bin/carddisp.pl?gene=VTN
FOXC2	Forkhead Box C2	Protein Coding	45	GC16P086570	11.80293083	https://www.genecards.org/cgi-bin/carddisp.pl?gene=FOXC2
GREM1	Gremlin 1, DAN Family BMP Antagonist	Protein Coding	44	GC15P033128	11.79185867	https://www.genecards.org/cgi-bin/carddisp.pl?gene=GREM1
EGR1	Early Growth Response 1	Protein Coding	44	GC05P138465	11.78876686	https://www.genecards.org/cgi-bin/carddisp.pl?gene=EGR1
RDH5	Retinol Dehydrogenase 5	Protein Coding	47	GC12P055720	11.77545261	https://www.genecards.org/cgi-bin/carddisp.pl?gene=RDH5
LECT2	Leukocyte Cell Derived Chemotaxin 2	Protein Coding	37	GC05M135922	11.76731968	https://www.genecards.org/cgi-bin/carddisp.pl?gene=LECT2
UBQLN4	Ubiquilin 4	Protein Coding	40	GC01M156033	11.59201622	https://www.genecards.org/cgi-bin/carddisp.pl?gene=UBQLN4
CYP4F3	Cytochrome P450 Family 4 Subfamily F Member 3	Protein Coding	44	GC19P015640	11.56530571	https://www.genecards.org/cgi-bin/carddisp.pl?gene=CYP4F3
RUNX3	RUNX Family Transcription Factor 3	Protein Coding	44	GC01M024899	11.55098152	https://www.genecards.org/cgi-bin/carddisp.pl?gene=RUNX3
STING1	Stimulator Of Interferon Response CGAMP Interactor 1	Protein Coding	34	GC05M139476	11.53891373	https://www.genecards.org/cgi-bin/carddisp.pl?gene=STING1
SPTAN1	Spectrin Alpha, Non-Erythrocytic 1	Protein Coding	48	GC09P128552	11.51099682	https://www.genecards.org/cgi-bin/carddisp.pl?gene=SPTAN1
IGF2BP2	Insulin Like Growth Factor 2 MRNA Binding Protein 2	Protein Coding	44	GC03M185643	11.45017338	https://www.genecards.org/cgi-bin/carddisp.pl?gene=IGF2BP2
FOXF1	Forkhead Box F1	Protein Coding	43	GC16P086510	11.43947029	https://www.genecards.org/cgi-bin/carddisp.pl?gene=FOXF1
HYOU1	Hypoxia Up-Regulated 1	Protein Coding	44	GC11M119045	11.43041039	https://www.genecards.org/cgi-bin/carddisp.pl?gene=HYOU1
CNR2	Cannabinoid Receptor 2	Protein Coding	46	GC01M023870	11.39825153	https://www.genecards.org/cgi-bin/carddisp.pl?gene=CNR2
PRPF8	Pre-MRNA Processing Factor 8	Protein Coding	42	GC17M001650	11.3741169	https://www.genecards.org/cgi-bin/carddisp.pl?gene=PRPF8
PTX3	Pentraxin 3	Protein Coding	43	GC03P157436	11.37303257	https://www.genecards.org/cgi-bin/carddisp.pl?gene=PTX3
ACLY	ATP Citrate Lyase	Protein Coding	47	GC17M041866	11.3557663	https://www.genecards.org/cgi-bin/carddisp.pl?gene=ACLY
GAL	Galanin And GMAP Prepropeptide	Protein Coding	45	GC11P068684	11.31365013	https://www.genecards.org/cgi-bin/carddisp.pl?gene=GAL
EPAS1	Endothelial PAS Domain Protein 1	Protein Coding	50	GC02P046293	11.29292488	https://www.genecards.org/cgi-bin/carddisp.pl?gene=EPAS1
BCL2L11	BCL2 Like 11	Protein Coding	45	GC02P111119	11.28868675	https://www.genecards.org/cgi-bin/carddisp.pl?gene=BCL2L11
ADH5	Alcohol Dehydrogenase 5 (Class III), Chi Polypeptide	Protein Coding	47	GC04M099070	11.26165009	https://www.genecards.org/cgi-bin/carddisp.pl?gene=ADH5
P2RX7	Purinergic Receptor P2X 7	Protein Coding	46	GC12P123575	11.25559235	https://www.genecards.org/cgi-bin/carddisp.pl?gene=P2RX7
UQCRQ	Ubiquinol-Cytochrome C Reductase Complex III Subunit VII	Protein Coding	42	GC05P132866	11.23309517	https://www.genecards.org/cgi-bin/carddisp.pl?gene=UQCRQ
SFRP4	Secreted Frizzled Related Protein 4	Protein Coding	44	GC07M037912	11.23053169	https://www.genecards.org/cgi-bin/carddisp.pl?gene=SFRP4
NOX4	NADPH Oxidase 4	Protein Coding	43	GC11M089324	11.20555115	https://www.genecards.org/cgi-bin/carddisp.pl?gene=NOX4
PHGDH	Phosphoglycerate Dehydrogenase	Protein Coding	50	GC01P119660	11.1698761	https://www.genecards.org/cgi-bin/carddisp.pl?gene=PHGDH
TKT	Transketolase	Protein Coding	48	GC03M053224	11.16229248	https://www.genecards.org/cgi-bin/carddisp.pl?gene=TKT
ITGA8	Integrin Subunit Alpha 8	Protein Coding	43	GC10M015513	11.15959072	https://www.genecards.org/cgi-bin/carddisp.pl?gene=ITGA8
LPCAT3	Lysophosphatidylcholine Acyltransferase 3	Protein Coding	36	GC12M006976	11.08821201	https://www.genecards.org/cgi-bin/carddisp.pl?gene=LPCAT3
IFNAR1	Interferon Alpha And Beta Receptor Subunit 1	Protein Coding	45	GC21P033324	11.03121376	https://www.genecards.org/cgi-bin/carddisp.pl?gene=IFNAR1
CPB2	Carboxypeptidase B2	Protein Coding	45	GC13M046053	11.02668762	https://www.genecards.org/cgi-bin/carddisp.pl?gene=CPB2
CLRN1	Clarin 1	Protein Coding	35	GC03M150926	11.02517796	https://www.genecards.org/cgi-bin/carddisp.pl?gene=CLRN1
TLR6	Toll Like Receptor 6	Protein Coding	44	GC04M038828	11.0105896	https://www.genecards.org/cgi-bin/carddisp.pl?gene=TLR6
AREG	Amphiregulin	Protein Coding	43	GC04P074445	10.98157692	https://www.genecards.org/cgi-bin/carddisp.pl?gene=AREG
RARS1	Arginyl-TRNA Synthetase 1	Protein Coding	37	GC05P168487	10.95300484	https://www.genecards.org/cgi-bin/carddisp.pl?gene=RARS1
SOD3	Superoxide Dismutase 3	Protein Coding	41	GC04P024798	10.91558552	https://www.genecards.org/cgi-bin/carddisp.pl?gene=SOD3
P4HB	Prolyl 4-Hydroxylase Subunit Beta	Protein Coding	50	GC17M081843	10.91475868	https://www.genecards.org/cgi-bin/carddisp.pl?gene=P4HB
HNMT	Histamine N-Methyltransferase	Protein Coding	46	GC02P137964	10.89535236	https://www.genecards.org/cgi-bin/carddisp.pl?gene=HNMT
BIRC3	Baculoviral IAP Repeat Containing 3	Protein Coding	47	GC11P102317	10.89016342	https://www.genecards.org/cgi-bin/carddisp.pl?gene=BIRC3
MAP3K7	Mitogen-Activated Protein Kinase Kinase Kinase 7	Protein Coding	51	GC06M090513	10.88270187	https://www.genecards.org/cgi-bin/carddisp.pl?gene=MAP3K7
MIR30C1	MicroRNA 30c-1	RNA Gene	22	GC01P040757	10.82134056	https://www.genecards.org/cgi-bin/carddisp.pl?gene=MIR30C1
ENPP2	Ectonucleotide Pyrophosphatase/Phosphodiesterase 2	Protein Coding	44	GC08M119556	10.81968975	https://www.genecards.org/cgi-bin/carddisp.pl?gene=ENPP2
JCAD	Junctional Cadherin 5 Associated	Protein Coding	26	GC10M030012	10.79609871	https://www.genecards.org/cgi-bin/carddisp.pl?gene=JCAD
RTN4	Reticulon 4	Protein Coding	45	GC02M054934	10.78720856	https://www.genecards.org/cgi-bin/carddisp.pl?gene=RTN4
COX4I2	Cytochrome C Oxidase Subunit 4I2	Protein Coding	43	GC20P031637	10.7298584	https://www.genecards.org/cgi-bin/carddisp.pl?gene=COX4I2
NNMT	Nicotinamide N-Methyltransferase	Protein Coding	46	GC11P114257	10.72583866	https://www.genecards.org/cgi-bin/carddisp.pl?gene=NNMT
IL16	Interleukin 16	Protein Coding	43	GC15P081159	10.7183075	https://www.genecards.org/cgi-bin/carddisp.pl?gene=IL16
TRAPPC9	Trafficking Protein Particle Complex Subunit 9	Protein Coding	39	GC08M139728	10.7010622	https://www.genecards.org/cgi-bin/carddisp.pl?gene=TRAPPC9
SERPINB3	Serpin Family B Member 3	Protein Coding	43	GC18M063655	10.66518497	https://www.genecards.org/cgi-bin/carddisp.pl?gene=SERPINB3
MYL2	Myosin Light Chain 2	Protein Coding	50	GC12M110910	10.65204239	https://www.genecards.org/cgi-bin/carddisp.pl?gene=MYL2
IL18RAP	Interleukin 18 Receptor Accessory Protein	Protein Coding	39	GC02P102418	10.53256416	https://www.genecards.org/cgi-bin/carddisp.pl?gene=IL18RAP
ADH1A	Alcohol Dehydrogenase 1A (Class I), Alpha Polypeptide	Protein Coding	43	GC04M099276	10.49468613	https://www.genecards.org/cgi-bin/carddisp.pl?gene=ADH1A
CUL3	Cullin 3	Protein Coding	47	GC02M224470	10.49339867	https://www.genecards.org/cgi-bin/carddisp.pl?gene=CUL3
TRIB1	Tribbles Pseudokinase 1	Protein Coding	38	GC08P125430	10.48884678	https://www.genecards.org/cgi-bin/carddisp.pl?gene=TRIB1
JAZF1	JAZF Zinc Finger 1	Protein Coding	39	GC07M027830	10.43261147	https://www.genecards.org/cgi-bin/carddisp.pl?gene=JAZF1
OGT	O-Linked N-Acetylglucosamine (GlcNAc) Transferase	Protein Coding	45	GC0XP071534	10.3878212	https://www.genecards.org/cgi-bin/carddisp.pl?gene=OGT
PDK4	Pyruvate Dehydrogenase Kinase 4	Protein Coding	47	GC07M095583	10.38490677	https://www.genecards.org/cgi-bin/carddisp.pl?gene=PDK4
GFPT1	Glutamine--Fructose-6-Phosphate Transaminase 1	Protein Coding	47	GC02M069283	10.33113861	https://www.genecards.org/cgi-bin/carddisp.pl?gene=GFPT1
USF1	Upstream Transcription Factor 1	Protein Coding	44	GC01M161039	10.30202293	https://www.genecards.org/cgi-bin/carddisp.pl?gene=USF1
COLGALT1	Collagen Beta(1-O)Galactosyltransferase 1	Protein Coding	37	GC19P017555	10.28843689	https://www.genecards.org/cgi-bin/carddisp.pl?gene=COLGALT1
CYGB	Cytoglobin	Protein Coding	39	GC17M076527	10.2549715	https://www.genecards.org/cgi-bin/carddisp.pl?gene=CYGB
APEX1	Apurinic/Apyrimidinic Endodeoxyribonuclease 1	Protein Coding	45	GC14P020455	10.24124527	https://www.genecards.org/cgi-bin/carddisp.pl?gene=APEX1

MATN3	Matrilin 3	Protein Coding	43	GC02M019992	10.21483612	https://www.genecards.org/cgi-bin/carddisp.pl?gene=MATN3
MIR423	MicroRNA 423	RNA Gene	18	GC17P030117	10.20427418	https://www.genecards.org/cgi-bin/carddisp.pl?gene=MIR423
PIK3R3	Phosphoinositide-3-Kinase Regulatory Subunit 3	Protein Coding	44	GC01M046041	10.13710117	https://www.genecards.org/cgi-bin/carddisp.pl?gene=PIK3R3
PDGFA	Platelet Derived Growth Factor Subunit A	Protein Coding	44	GC07M000497	10.11775589	https://www.genecards.org/cgi-bin/carddisp.pl?gene=PDGFA
MLX	MAX Dimerization Protein MLX	Protein Coding	44	GC17P042567	10.09683323	https://www.genecards.org/cgi-bin/carddisp.pl?gene=MLX
NDUFC2	NADH:Ubiquinone Oxidoreductase Subunit C2	Protein Coding	41	GC11M078068	10.09604931	https://www.genecards.org/cgi-bin/carddisp.pl?gene=NDUFC2
RORA	RAR Related Orphan Receptor A	Protein Coding	50	GC15M060488	10.09284115	https://www.genecards.org/cgi-bin/carddisp.pl?gene=RORA
SLC39A14	Solute Carrier Family 39 Member 14	Protein Coding	44	GC08P022367	10.06453514	https://www.genecards.org/cgi-bin/carddisp.pl?gene=SLC39A14
MGAM	Maltase-Glucoamylase	Protein Coding	42	GC07P145925	10.06344604	https://www.genecards.org/cgi-bin/carddisp.pl?gene=MGAM
NLRC4	NLR Family CARD Domain Containing 4	Protein Coding	45	GC02M032224	10.02363777	https://www.genecards.org/cgi-bin/carddisp.pl?gene=NLRC4
MIR30B	MicroRNA 30b	RNA Gene	20	GC08M134800	10.00792694	https://www.genecards.org/cgi-bin/carddisp.pl?gene=MIR30B
TRIM21	Tripartite Motif Containing 21	Protein Coding	43	GC11M004384	10.00412178	https://www.genecards.org/cgi-bin/carddisp.pl?gene=TRIM21
NOS1AP	Nitric Oxide Synthase 1 Adaptor Protein	Protein Coding	39	GC01P162069	9.991197586	https://www.genecards.org/cgi-bin/carddisp.pl?gene=NOS1AP
SNRNP200	Small Nuclear Ribonucleoprotein U5 Subunit 200	Protein Coding	42	GC02M096363	9.948816299	https://www.genecards.org/cgi-bin/carddisp.pl?gene=SNRNP200
CACNA2D1	Calcium Voltage-Gated Channel Auxiliary Subunit Alpha2delta 1	Protein Coding	47	GC07M081946	9.947055817	https://www.genecards.org/cgi-bin/carddisp.pl?gene=CACNA2D1
PRPF3	Pre-MRNA Processing Factor 3	Protein Coding	44	GC01P150321	9.927322388	https://www.genecards.org/cgi-bin/carddisp.pl?gene=PRPF3
PRDX5	Peroxiredoxin 5	Protein Coding	47	GC11P064317	9.915092468	https://www.genecards.org/cgi-bin/carddisp.pl?gene=PRDX5
GOT1	Glutamic-Oxaloacetic Transaminase 1	Protein Coding	47	GC10M099396	9.902200699	https://www.genecards.org/cgi-bin/carddisp.pl?gene=GOT1
CYLD	CYLD Lysine 63 Deubiquitinase	Protein Coding	50	GC16P050742	9.901107788	https://www.genecards.org/cgi-bin/carddisp.pl?gene=CYLD
WWTR1	WW Domain Containing Transcription Regulator 1	Protein Coding	43	GC03M149517	9.89236927	https://www.genecards.org/cgi-bin/carddisp.pl?gene=WWTR1
AOC3	Amine Oxidase Copper Containing 3	Protein Coding	44	GC17P042851	9.871923447	https://www.genecards.org/cgi-bin/carddisp.pl?gene=AOC3
AZGP1	Alpha-2-Glycoprotein 1, Zinc-Binding	Protein Coding	44	GC07M099967	9.869994164	https://www.genecards.org/cgi-bin/carddisp.pl?gene=AZGP1
SLC25A12	Solute Carrier Family 25 Member 12	Protein Coding	47	GC02M171783	9.854891777	https://www.genecards.org/cgi-bin/carddisp.pl?gene=SLC25A12
CFL1	Cofilin 1	Protein Coding	47	GC11M065823	9.842188835	https://www.genecards.org/cgi-bin/carddisp.pl?gene=CFL1
TMPO	Thymopoietin	Protein Coding	45	GC12P098515	9.820468903	https://www.genecards.org/cgi-bin/carddisp.pl?gene=TMPO
PARVB	Parvin Beta	Protein Coding	40	GC22P043999	9.806926727	https://www.genecards.org/cgi-bin/carddisp.pl?gene=PARVB
BID	BH3 Interacting Domain Death Agonist	Protein Coding	46	GC22M017734	9.798443794	https://www.genecards.org/cgi-bin/carddisp.pl?gene=BID
APLN	Apelin	Protein Coding	37	GC0XM129645	9.749046326	https://www.genecards.org/cgi-bin/carddisp.pl?gene=APLN
NCAN	Neurocan	Protein Coding	41	GC19P026662	9.744245529	https://www.genecards.org/cgi-bin/carddisp.pl?gene=NCAN
SLC9A9	Solute Carrier Family 9 Member A9	Protein Coding	40	GC03M143265	9.692720413	https://www.genecards.org/cgi-bin/carddisp.pl?gene=SLC9A9
PER2	Period Circadian Regulator 2	Protein Coding	44	GC02M238244	9.684893608	https://www.genecards.org/cgi-bin/carddisp.pl?gene=PER2
COL13A1	Collagen Type XIII Alpha 1 Chain	Protein Coding	41	GC10P069801	9.676892281	https://www.genecards.org/cgi-bin/carddisp.pl?gene=COL13A1
ALDH9A1	Aldehyde Dehydrogenase 9 Family Member A1	Protein Coding	42	GC01M165670	9.657175064	https://www.genecards.org/cgi-bin/carddisp.pl?gene=ALDH9A1
GLP1R	Glucagon Like Peptide 1 Receptor	Protein Coding	47	GC06P039048	9.635458946	https://www.genecards.org/cgi-bin/carddisp.pl?gene=GLP1R
CD82	CD82 Molecule	Protein Coding	43	GC11P044586	9.633619308	https://www.genecards.org/cgi-bin/carddisp.pl?gene=CD82
TYSND1	Trypsin Like Peroxisomal Matrix	Protein Coding	35	GC10M070137	9.625871658	https://www.genecards.org/cgi-bin/carddisp.pl?gene=TYSND1
FOXA1	Forkhead Box A1	Protein Coding	43	GC14M037589	9.614583969	https://www.genecards.org/cgi-bin/carddisp.pl?gene=FOXA1
PLIN5	Perilipin 5	Protein Coding	33	GC19M004522	9.6139431	https://www.genecards.org/cgi-bin/carddisp.pl?gene=PLIN5
DISC1	DISC1 Scaffold Protein	Protein Coding	44	GC01P231626	9.609996796	https://www.genecards.org/cgi-bin/carddisp.pl?gene=DISC1
COMMD1	Copper Metabolism Domain Containing 1	Protein Coding	43	GC02P061888	9.602817535	https://www.genecards.org/cgi-bin/carddisp.pl?gene=COMMD1
EFTUD2	Elongation Factor Tu GTP Binding Domain Containing 2	Protein Coding	43	GC17M044852	9.594213486	https://www.genecards.org/cgi-bin/carddisp.pl?gene=EFTUD2
PDXK	Pyridoxal Kinase	Protein Coding	49	GC21P043719	9.578528404	https://www.genecards.org/cgi-bin/carddisp.pl?gene=PDXK
CDH4	Cadherin 4	Protein Coding	43	GC20P061252	9.577430725	https://www.genecards.org/cgi-bin/carddisp.pl?gene=CDH4
GPI	Glucose-6-Phosphate Isomerase	Protein Coding	47	GC19P034359	9.558523178	https://www.genecards.org/cgi-bin/carddisp.pl?gene=GPI
YWHAE	Tyrosine 3-Monooxygenase/Tryptophan 5-Monooxygenase Activation Protein	Protein Coding	50	GC17M001346	9.536681175	https://www.genecards.org/cgi-bin/carddisp.pl?gene=YWHAE
SLC1A3	Solute Carrier Family 1 Member 3	Protein Coding	51	GC05P036620	9.508004189	https://www.genecards.org/cgi-bin/carddisp.pl?gene=SLC1A3
COX7B	Cytochrome C Oxidase Subunit 7B	Protein Coding	41	GC0XP077899	9.46569252	https://www.genecards.org/cgi-bin/carddisp.pl?gene=COX7B
CYP8B1	Cytochrome P450 Family 8 Subfamily B Member 1	Protein Coding	41	GC03M042856	9.457562447	https://www.genecards.org/cgi-bin/carddisp.pl?gene=CYP8B1
ITPR2	Inositol 1,4,5-Trisphosphate Receptor	Protein Coding	47	GC12M026336	9.429243088	https://www.genecards.org/cgi-bin/carddisp.pl?gene=ITPR2
TRAP1	TNF Receptor Associated Protein 1	Protein Coding	44	GC16M003667	9.409546852	https://www.genecards.org/cgi-bin/carddisp.pl?gene=TRAP1
MIR103A1	MicroRNA 103a-1	RNA Gene	18	GC05M168560	9.403093338	https://www.genecards.org/cgi-bin/carddisp.pl?gene=MIR103A1
YWHAQ	Tyrosine 3-Monooxygenase/Tryptophan 5-Monooxygenase Activation Protein Theta	Protein Coding	48	GC02M009583	9.393497467	https://www.genecards.org/cgi-bin/carddisp.pl?gene=YWHAQ
PRKAG1	Protein Kinase AMP-Activated Non-Catalytic Subunit Gamma 1	Protein Coding	49	GC12M049002	9.357595444	https://www.genecards.org/cgi-bin/carddisp.pl?gene=PRKAG1
MYH10	Myosin Heavy Chain 10	Protein Coding	47	GC17M008474	9.353853226	https://www.genecards.org/cgi-bin/carddisp.pl?gene=MYH10
CXCR5	C-X-C Motif Chemokine Receptor 5	Protein Coding	42	GC11P118908	9.32213974	https://www.genecards.org/cgi-bin/carddisp.pl?gene=CXCR5
YY1	YY1 Transcription Factor	Protein Coding	48	GC14P100238	9.321865082	https://www.genecards.org/cgi-bin/carddisp.pl?gene=YY1
PIN1	Peptidylprolyl Cis/Trans Isomerase, NIMA-Interacting 1	Protein Coding	48	GC19P009835	9.319272995	https://www.genecards.org/cgi-bin/carddisp.pl?gene=PIN1
PRKDC	Protein Kinase, DNA-Activated, Catalytic Subunit	Protein Coding	50	GC08M047773	9.275913239	https://www.genecards.org/cgi-bin/carddisp.pl?gene=PRKDC
XRCC6	X-Ray Repair Cross Complementing 6	Protein Coding	47	GC22P041622	9.273457527	https://www.genecards.org/cgi-bin/carddisp.pl?gene=XRCC6
CASP2	Caspase 2	Protein Coding	50	GC07P145813	9.271942139	https://www.genecards.org/cgi-bin/carddisp.pl?gene=CASP2
EEA1	Early Endosome Antigen 1	Protein Coding	44	GC12M092770	9.25849247	https://www.genecards.org/cgi-bin/carddisp.pl?gene=EEA1
CDH5	Cadherin 5	Protein Coding	48	GC16P066366	9.249822617	https://www.genecards.org/cgi-bin/carddisp.pl?gene=CDH5
UQCRC1	Ubiquinol-Cytochrome C Reductase Core Protein 1	Protein Coding	44	GC03M048598	9.242477417	https://www.genecards.org/cgi-bin/carddisp.pl?gene=UQCRC1
HSD17B13	Hydroxysteroid 17-Beta Dehydrogenase 13	Protein Coding	37	GC04M087303	9.22826767	https://www.genecards.org/cgi-bin/carddisp.pl?gene=HSD17B13
PTPRD	Protein Tyrosine Phosphatase Receptor Type D	Protein Coding	45	GC09M008307	9.208004951	https://www.genecards.org/cgi-bin/carddisp.pl?gene=PTPRD
RPL10	Ribosomal Protein L10	Protein Coding	45	GC0XP154389	9.195249557	https://www.genecards.org/cgi-bin/carddisp.pl?gene=RPL10
HPS1	HPS1 Biogenesis Of Lysosomal Organelles Complex 3 Subunit 1	Protein Coding	43	GC10M098416	9.152247429	https://www.genecards.org/cgi-bin/carddisp.pl?gene=HPS1
RPS15	Ribosomal Protein S15	Protein Coding	41	GC19P001438	9.122495651	https://www.genecards.org/cgi-bin/carddisp.pl?gene=RPS15
AXL	AXL Receptor Tyrosine Kinase	Protein Coding	52	GC19P041219	9.080257416	https://www.genecards.org/cgi-bin/carddisp.pl?gene=AXL
NCOA3	Nuclear Receptor Coactivator 3	Protein Coding	47	GC20P047501	9.04309082	https://www.genecards.org/cgi-bin/carddisp.pl?gene=NCOA3
SLC39A4	Solute Carrier Family 39 Member 4	Protein Coding	44	GC08M144409	9.026463509	https://www.genecards.org/cgi-bin/carddisp.pl?gene=SLC39A4
SLC4A4	Solute Carrier Family 4 Member 4	Protein Coding	47	GC04P071063	9.012678146	https://www.genecards.org/cgi-bin/carddisp.pl?gene=SLC4A4
SIRT6	Sirtuin 6	Protein Coding	47	GC19M004174	9.008852959	https://www.genecards.org/cgi-bin/carddisp.pl?gene=SIRT6
SLC25A22	Solute Carrier Family 25 Member 22	Protein Coding	41	GC11M001138	9.001564026	https://www.genecards.org/cgi-bin/carddisp.pl?gene=SLC25A22
TNFRSF4	TNF Receptor Superfamily Member 4	Protein Coding	43	GC01M001211	8.997526169	https://www.genecards.org/cgi-bin/carddisp.pl?gene=TNFRSF4
EDA	Ectodysplasin A	Protein Coding	41	GC0XP069618	8.956465721	https://www.genecards.org/cgi-bin/carddisp.pl?gene=EDA
ADRB1	Adrenoceptor Beta 1	Protein Coding	49	GC10P114044	8.924825668	https://www.genecards.org/cgi-bin/carddisp.pl?gene=ADRB1
XRCC5	X-Ray Repair Cross Complementing 5	Protein Coding	45	GC02P216107	8.907269478	https://www.genecards.org/cgi-bin/carddisp.pl?gene=XRCC5
SAMM50	SAMM50 Sorting And Assembly Machinery Component	Protein Coding	38	GC22P043955	8.895086288	https://www.genecards.org/cgi-bin/carddisp.pl?gene=SAMM50
IMMT	Inner Membrane Mitochondrial Protein	Protein Coding	40	GC02M086144	8.849901199	https://www.genecards.org/cgi-bin/carddisp.pl?gene=IMMT
SIK1	Salt Inducible Kinase 1	Protein Coding	47	GC21M043414	8.841324806	https://www.genecards.org/cgi-bin/carddisp.pl?gene=SIK1
ALPI	Alkaline Phosphatase, Intestinal	Protein Coding	44	GC02P232456	8.8091259	https://www.genecards.org/cgi-bin/carddisp.pl?gene=ALPI
ERLIN2	ER Lipid Raft Associated 2	Protein Coding	41	GC08P037736	8.787817001	https://www.genecards.org/cgi-bin/carddisp.pl?gene=ERLIN2
CHRM3	Cholinergic Receptor Muscarinic 3	Protein Coding	50	GC01P239386	8.779540062	https://www.genecards.org/cgi-bin/carddisp.pl?gene=CHRM3
LCN1	Lipocalin 1	Protein Coding	40	GC09P135521	8.770139694	https://www.genecards.org/cgi-bin/carddisp.pl?gene=LCN1
SERPINA12	Serpin Family A Member 12	Protein Coding	38	GC14M095825	8.757614136	https://www.genecards.org/cgi-bin/carddisp.pl?gene=SERPINA12
CLDN2	Claudin 2	Protein Coding	42	GC0XP106900	8.728883743	https://www.genecards.org/cgi-bin/carddisp.pl?gene=CLDN2
TXNRD1	Thioredoxin Reductase 1	Protein Coding	48	GC12P104215	8.722616196	https://www.genecards.org/cgi-bin/carddisp.pl?gene=TXNRD1
NAT1	N-Acetyltransferase 1	Protein Coding	45	GC08P018179	8.714513779	https://www.genecards.org/cgi-bin/carddisp.pl?gene=NAT1
SLC34A2	Solute Carrier Family 34 Member 2	Protein Coding	47	GC04P025657	8.712742805	https://www.genecards.org/cgi-bin/carddisp.pl?gene=SLC34A2
MIR363	MicroRNA 363	RNA Gene	15	GC0XM134205	8.698446274	https://www.genecards.org/cgi-bin/carddisp.pl?gene=MIR363

EEF1A1	Eukaryotic Translation Elongation Factor 1 Alpha 1	Protein Coding	44	GC06M073515	8.65370369	https://www.genecards.org/cgi-bin/carddisp.pl?gene=EEF1A1
PRKACG	Protein Kinase CAMP-Activated Catalytic Subunit Gamma	Protein Coding	48	GC09M069013	8.649134636	https://www.genecards.org/cgi-bin/carddisp.pl?gene=PRKACG
CXCL11	C-X-C Motif Chemokine Ligand 11	Protein Coding	41	GC04M076033	8.647530556	https://www.genecards.org/cgi-bin/carddisp.pl?gene=CXCL11
TNC	Tenascin C	Protein Coding	50	GC09M115019	8.615830421	https://www.genecards.org/cgi-bin/carddisp.pl?gene=TNC
AKR1B10	Aldo-Keto Reductase Family 1 Member	Protein Coding	44	GC07P134527	8.546919823	https://www.genecards.org/cgi-bin/carddisp.pl?gene=AKR1B10
OPRM1	Opioid Receptor Mu 1	Protein Coding	49	GC06P154075	8.530946732	https://www.genecards.org/cgi-bin/carddisp.pl?gene=OPRM1
RPS17	Ribosomal Protein S17	Protein Coding	43	GC15M082536	8.52791214	https://www.genecards.org/cgi-bin/carddisp.pl?gene=RPS17
GPNMB	Glycoprotein Nmb	Protein Coding	44	GC07P023238	8.51955986	https://www.genecards.org/cgi-bin/carddisp.pl?gene=GPNMB
ITLN1	Intelectin 1	Protein Coding	39	GC01M160876	8.504840851	https://www.genecards.org/cgi-bin/carddisp.pl?gene=ITLN1
PPIB	Peptidylprolyl Isomerase B	Protein Coding	50	GC15M064155	8.407612801	https://www.genecards.org/cgi-bin/carddisp.pl?gene=PPIB
GPC6	Glypican 6	Protein Coding	44	GC13P093226	8.397035599	https://www.genecards.org/cgi-bin/carddisp.pl?gene=GPC6
PRKAB2	Protein Kinase AMP-Activated Non-Catalytic Subunit Beta 2	Protein Coding	47	GC01M147155	8.382809639	https://www.genecards.org/cgi-bin/carddisp.pl?gene=PRKAB2
APOF	Apolipoprotein F	Protein Coding	39	GC12M056371	8.347465515	https://www.genecards.org/cgi-bin/carddisp.pl?gene=APOF
EIF3F	Eukaryotic Translation Initiation Factor 3 Subunit F	Protein Coding	44	GC11P007966	8.335307121	https://www.genecards.org/cgi-bin/carddisp.pl?gene=EIF3F
CISD2	CDGSH Iron Sulfur Domain 2	Protein Coding	42	GC04P102868	8.319753647	https://www.genecards.org/cgi-bin/carddisp.pl?gene=CISD2
TRAF2	TNF Receptor Associated Factor 2	Protein Coding	45	GC09P136881	8.298054695	https://www.genecards.org/cgi-bin/carddisp.pl?gene=TRAF2
HSP90B1	Heat Shock Protein 90 Beta Family Member 1	Protein Coding	47	GC12P103930	8.281648636	https://www.genecards.org/cgi-bin/carddisp.pl?gene=HSP90B1
MYO6	Myosin VI	Protein Coding	47	GC06P075749	8.277293205	https://www.genecards.org/cgi-bin/carddisp.pl?gene=MYO6
MOGAT2	Monoacylglycerol O-Acyltransferase 2	Protein Coding	38	GC11P075717	8.266434669	https://www.genecards.org/cgi-bin/carddisp.pl?gene=MOGAT2
GRK2	G Protein-Coupled Receptor Kinase 2	Protein Coding	38	GC11P067266	8.264247894	https://www.genecards.org/cgi-bin/carddisp.pl?gene=GRK2
NDUFA4	NDUFA4 Mitochondrial Complex	Protein Coding	44	GC07M010938	8.262947083	https://www.genecards.org/cgi-bin/carddisp.pl?gene=NDUFA4
FUNDC2	FUN14 Domain Containing 2	Protein Coding	35	GC0XP155025	8.254704475	https://www.genecards.org/cgi-bin/carddisp.pl?gene=FUNDC2
SNTA1	Syntrophin Alpha 1	Protein Coding	45	GC20M033407	8.232112885	https://www.genecards.org/cgi-bin/carddisp.pl?gene=SNTA1
AMPH	Amphiphysin	Protein Coding	45	GC07M038782	8.201856613	https://www.genecards.org/cgi-bin/carddisp.pl?gene=AMPH
ZNF365	Zinc Finger Protein 365	Protein Coding	38	GC10P062374	8.189241409	https://www.genecards.org/cgi-bin/carddisp.pl?gene=ZNF365
NDUFC2-KCTD14	NDUFC2-KCTD14 Readthrough	Protein Coding	22	GC11M078015	8.181237221	https://www.genecards.org/cgi-bin/carddisp.pl?gene=NDUFC2-KCTD14
AHNAK	AHNAK Nucleoprotein	Protein Coding	37	GC11M069238	8.172440529	https://www.genecards.org/cgi-bin/carddisp.pl?gene=AHNAK
CCN1	Cellular Communication Network Factor 1	Protein Coding	31	GC01P085581	8.171933174	https://www.genecards.org/cgi-bin/carddisp.pl?gene=CCN1
MIR361	MicroRNA 361	RNA Gene	16	GC0XM085903	8.155086517	https://www.genecards.org/cgi-bin/carddisp.pl?gene=MIR361
NRG3	Neuregulin 3	Protein Coding	43	GC10P085390	8.129633904	https://www.genecards.org/cgi-bin/carddisp.pl?gene=NRG3
ARNT	Aryl Hydrocarbon Receptor Nuclear Translocator	Protein Coding	44	GC01M150809	8.125787735	https://www.genecards.org/cgi-bin/carddisp.pl?gene=ARNT
DMGDH	Dimethylglycine Dehydrogenase	Protein Coding	44	GC05M078997	8.077875137	https://www.genecards.org/cgi-bin/carddisp.pl?gene=DMGDH
PSMC2	Proteasome 26S Subunit, ATPase 2	Protein Coding	41	GC07P103344	8.034032822	https://www.genecards.org/cgi-bin/carddisp.pl?gene=PSMC2
KAT5	Lysine Acetyltransferase 5	Protein Coding	47	GC11P065711	8.023927689	https://www.genecards.org/cgi-bin/carddisp.pl?gene=KAT5
YWHAG	Tyrosine 3-Monooxygenase/Tryptophan 5-Monooxygenase Activation Protein	Protein Coding	50	GC07M076327	8.020187378	https://www.genecards.org/cgi-bin/carddisp.pl?gene=YWHAG
MACROD2	Mono-ADP Ribosylhydrolase 2	Protein Coding	36	GC20P013925	8.018939972	https://www.genecards.org/cgi-bin/carddisp.pl?gene=MACROD2
SLC44A1	Solute Carrier Family 44 Member 1	Protein Coding	45	GC09P105244	8.014473915	https://www.genecards.org/cgi-bin/carddisp.pl?gene=SLC44A1
CCT5	Chaperonin Containing TCP1 Subunit 5	Protein Coding	45	GC05P010236	7.981685638	https://www.genecards.org/cgi-bin/carddisp.pl?gene=CCT5
IL27	Interleukin 27	Protein Coding	38	GC16M028514	7.952753544	https://www.genecards.org/cgi-bin/carddisp.pl?gene=IL27
MIR590	MicroRNA 590	RNA Gene	18	GC07P074191	7.944304466	https://www.genecards.org/cgi-bin/carddisp.pl?gene=MIR590
RPL27	Ribosomal Protein L27	Protein Coding	43	GC17P042998	7.942708492	https://www.genecards.org/cgi-bin/carddisp.pl?gene=RPL27
LOXL2	Lysyl Oxidase Like 2	Protein Coding	44	GC08M023296	7.93822813	https://www.genecards.org/cgi-bin/carddisp.pl?gene=LOXL2
TP53BP1	Tumor Protein P53 Binding Protein 1	Protein Coding	45	GC15M043403	7.923695087	https://www.genecards.org/cgi-bin/carddisp.pl?gene=TP53BP1
HSPA6	Heat Shock Protein Family A (Hsp70) Member 6	Protein Coding	45	GC01P161524	7.898610115	https://www.genecards.org/cgi-bin/carddisp.pl?gene=HSPA6
SLC25A47	Solute Carrier Family 25 Member 47	Protein Coding	35	GC14P100323	7.896558762	https://www.genecards.org/cgi-bin/carddisp.pl?gene=SLC25A47
SIRT4	Sirtuin 4	Protein Coding	40	GC12P120291	7.884446144	https://www.genecards.org/cgi-bin/carddisp.pl?gene=SIRT4
DCLK1	Doublecortin Like Kinase 1	Protein Coding	44	GC13M035768	7.845679283	https://www.genecards.org/cgi-bin/carddisp.pl?gene=DCLK1
IGFBP7	Insulin Like Growth Factor Binding	Protein Coding	47	GC04M057030	7.816283226	https://www.genecards.org/cgi-bin/carddisp.pl?gene=IGFBP7
AQP9	Aquaporin 9	Protein Coding	44	GC15P058138	7.780819893	https://www.genecards.org/cgi-bin/carddisp.pl?gene=AQP9
DROSHA	Drosha Ribonuclease III	Protein Coding	43	GC05M031401	7.770452976	https://www.genecards.org/cgi-bin/carddisp.pl?gene=DROSHA
STEAP4	STEAP4 Metalloreductase	Protein Coding	41	GC07M088315	7.76199007	https://www.genecards.org/cgi-bin/carddisp.pl?gene=STEAP4
TAGLN	Transgelin	Protein Coding	44	GC11P117199	7.757493973	https://www.genecards.org/cgi-bin/carddisp.pl?gene=TAGLN
PZP	PZP Alpha-2-Macroglobulin Like	Protein Coding	40	GC12M009148	7.755242348	https://www.genecards.org/cgi-bin/carddisp.pl?gene=PZP
TLL1	Tolloid Like 1	Protein Coding	44	GC04P165873	7.748857498	https://www.genecards.org/cgi-bin/carddisp.pl?gene=TLL1
IGFBP5	Insulin Like Growth Factor Binding	Protein Coding	43	GC02M216672	7.71900034	https://www.genecards.org/cgi-bin/carddisp.pl?gene=IGFBP5
PRSS2	Serine Protease 2	Protein Coding	41	GC07P145798	7.698745728	https://www.genecards.org/cgi-bin/carddisp.pl?gene=PRSS2
NFIC	Nuclear Factor I C	Protein Coding	41	GC19P003314	7.694828033	https://www.genecards.org/cgi-bin/carddisp.pl?gene=NFIC
TRAF1	TNF Receptor Associated Factor 1	Protein Coding	44	GC09M120902	7.691432953	https://www.genecards.org/cgi-bin/carddisp.pl?gene=TRAF1
ATP5F1B	ATP Synthase F1 Subunit Beta	Protein Coding	34	GC12M056639	7.682571411	https://www.genecards.org/cgi-bin/carddisp.pl?gene=ATP5F1B
RBFOX1	RNA Binding Fox-1 Homolog 1	Protein Coding	38	GC16P005688	7.667833805	https://www.genecards.org/cgi-bin/carddisp.pl?gene=RBFOX1
PPP2CA	Protein Phosphatase 2 Catalytic Subunit Alpha	Protein Coding	50	GC05M134194	7.647490501	https://www.genecards.org/cgi-bin/carddisp.pl?gene=PPP2CA
TSHB	Thyroid Stimulating Hormone Subunit	Protein Coding	43	GC01P115029	7.626227379	https://www.genecards.org/cgi-bin/carddisp.pl?gene=TSHB
TRAF3	TNF Receptor Associated Factor 3	Protein Coding	48	GC14P106036	7.617692471	https://www.genecards.org/cgi-bin/carddisp.pl?gene=TRAF3
SRSF1	Serine And Arginine Rich Splicing Factor	Protein Coding	43	GC17M058000	7.616688728	https://www.genecards.org/cgi-bin/carddisp.pl?gene=SRSF1
ATAD3A	ATPase Family AAA Domain Containing	Protein Coding	40	GC01P002047	7.607479095	https://www.genecards.org/cgi-bin/carddisp.pl?gene=ATAD3A
IGHG1	Immunoglobulin Heavy Constant Gamma 1 (G1m Marker)	Protein Coding	33	GC14M105736	7.600393295	https://www.genecards.org/cgi-bin/carddisp.pl?gene=IGHG1
FST	Follistatin	Protein Coding	47	GC05P053480	7.575922966	https://www.genecards.org/cgi-bin/carddisp.pl?gene=FST
LOC110806262	Solute Carrier Family 6 Member 4 Gene Promoter	Biological Region	1	GC17P030235	7.556408882	https://www.genecards.org/cgi-bin/carddisp.pl?gene=LOC110806262
H4-16	H4 Histone 16	Protein Coding	34	GC12M015752	7.543855667	https://www.genecards.org/cgi-bin/carddisp.pl?gene=H4-16
GPT2	Glutamic--Pyruvic Transaminase 2	Protein Coding	48	GC16P046885	7.525701523	https://www.genecards.org/cgi-bin/carddisp.pl?gene=GPT2
UGDH	UDP-Glucose 6-Dehydrogenase	Protein Coding	47	GC04M039502	7.50583744	https://www.genecards.org/cgi-bin/carddisp.pl?gene=UGDH
RPS15A	Ribosomal Protein S15a	Protein Coding	43	GC16M018781	7.494460106	https://www.genecards.org/cgi-bin/carddisp.pl?gene=RPS15A
PEG10	Paternally Expressed 10	Protein Coding	41	GC07P094656	7.489383698	https://www.genecards.org/cgi-bin/carddisp.pl?gene=PEG10
DGCR8	DGCR8 Microprocessor Complex Subunit	Protein Coding	42	GC22P020080	7.487957954	https://www.genecards.org/cgi-bin/carddisp.pl?gene=DGCR8
OSMR	Oncostatin M Receptor	Protein Coding	45	GC05P038845	7.485833645	https://www.genecards.org/cgi-bin/carddisp.pl?gene=OSMR
RPL7	Ribosomal Protein L7	Protein Coding	43	GC08M073290	7.477601051	https://www.genecards.org/cgi-bin/carddisp.pl?gene=RPL7
AFAP1-AS1	AFAP1 Antisense RNA 1	RNA Gene	16	GC04P007756	7.477543831	https://www.genecards.org/cgi-bin/carddisp.pl?gene=AFAP1-AS1
AUP1	AUP1 Lipid Droplet Regulating VLDL Assembly Factor	Protein Coding	37	GC02M074526	7.477283478	https://www.genecards.org/cgi-bin/carddisp.pl?gene=AUP1
PUF60	Poly(U) Binding Splicing Factor 60	Protein Coding	43	GC08M143816	7.467024803	https://www.genecards.org/cgi-bin/carddisp.pl?gene=PUF60
MYH2	Myosin Heavy Chain 2	Protein Coding	47	GC17M010521	7.437431335	https://www.genecards.org/cgi-bin/carddisp.pl?gene=MYH2
CD5L	CD5 Molecule Like	Protein Coding	39	GC01M157800	7.43599987	https://www.genecards.org/cgi-bin/carddisp.pl?gene=CD5L
MYO1B	Myosin IB	Protein Coding	39	GC02P191246	7.431186676	https://www.genecards.org/cgi-bin/carddisp.pl?gene=MYO1B
ELK1	ETS Transcription Factor ELK1	Protein Coding	44	GC0XM047635	7.425537109	https://www.genecards.org/cgi-bin/carddisp.pl?gene=ELK1
LGALS3BP	Galectin 3 Binding Protein	Protein Coding	41	GC17M078971	7.416460991	https://www.genecards.org/cgi-bin/carddisp.pl?gene=LGALS3BP
AQP3	Aquaporin 3 (Gill Blood Group)	Protein Coding	48	GC09M033431	7.314429283	https://www.genecards.org/cgi-bin/carddisp.pl?gene=AQP3
GPLD1	Glycosylphosphatidylinositol Specific Phospholipase D1	Protein Coding	43	GC06M024438	7.307159424	https://www.genecards.org/cgi-bin/carddisp.pl?gene=GPLD1
GABRA2	Gamma-Aminobutyric Acid Type A Receptor Subunit Alpha2	Protein Coding	47	GC04M046243	7.282387257	https://www.genecards.org/cgi-bin/carddisp.pl?gene=GABRA2
SEMA5A	Semaphorin 5A	Protein Coding	41	GC05M009036	7.270325184	https://www.genecards.org/cgi-bin/carddisp.pl?gene=SEMA5A
UBE2D2	Ubiquitin Conjugating Enzyme E2 D2	Protein Coding	45	GC05P139526	7.266618252	https://www.genecards.org/cgi-bin/carddisp.pl?gene=UBE2D2
CAD	Carbamoyl-Phosphate Synthetase 2, Aspartate Transcarbamylase, And Dihydroorotase	Protein Coding	51	GC02P027217	7.248846531	https://www.genecards.org/cgi-bin/carddisp.pl?gene=CAD
IFNL4	Interferon Lambda 4 (Gene/Pseudogene)	Protein Coding	19	GC19M039246	7.208087921	https://www.genecards.org/cgi-bin/carddisp.pl?gene=IFNL4

PSMD2	Proteasome 26S Subunit Ubiquitin Receptor, Non-ATPase 2	Protein Coding	44	GC03P184298	7.17443943	https://www.genecards.org/cgi-bin/carddisp.pl?gene=PSMD2
MAP3K11	Mitogen-Activated Protein Kinase Kinase Kinase 11	Protein Coding	48	GC11M069402	7.16268301	https://www.genecards.org/cgi-bin/carddisp.pl?gene=MAP3K11
SIPA1L2	Signal Induced Proliferation Associated 1 Like 2	Protein Coding	38	GC01M232397	7.149875641	https://www.genecards.org/cgi-bin/carddisp.pl?gene=SIPA1L2
SDC2	Syndecan 2	Protein Coding	45	GC08P096495	7.136144638	https://www.genecards.org/cgi-bin/carddisp.pl?gene=SDC2
MMP11	Matrix Metallopeptidase 11	Protein Coding	46	GC22P023768	7.109187603	https://www.genecards.org/cgi-bin/carddisp.pl?gene=MMP11
PARD3	Par-3 Family Cell Polarity Regulator	Protein Coding	43	GC10M034110	7.100137711	https://www.genecards.org/cgi-bin/carddisp.pl?gene=PARD3
KHDRBS3	KH RNA Binding Domain Containing, Signal Transduction Associated 3	Protein Coding	39	GC08P135457	7.090822697	https://www.genecards.org/cgi-bin/carddisp.pl?gene=KHDRBS3
CMKLR1	Chemerin Chemokine-Like Receptor 1	Protein Coding	41	GC12M108288	7.008006573	https://www.genecards.org/cgi-bin/carddisp.pl?gene=CMKLR1
MYF5	Myogenic Factor 5	Protein Coding	41	GC12P080716	7.007295609	https://www.genecards.org/cgi-bin/carddisp.pl?gene=MYF5
SORCS3	Sortilin Related VPS10 Domain Containing Receptor 3	Protein Coding	37	GC10P104642	6.999204159	https://www.genecards.org/cgi-bin/carddisp.pl?gene=SORCS3
HNRNPC	Heterogeneous Nuclear Ribonucleoprotein	Protein Coding	42	GC14M021210	6.948101521	https://www.genecards.org/cgi-bin/carddisp.pl?gene=HNRNPC
UFM1	Ubiquitin Fold Modifier 1	Protein Coding	41	GC13P038349	6.938527584	https://www.genecards.org/cgi-bin/carddisp.pl?gene=UFM1
CARS1	Cysteinyl-TRNA Synthetase 1	Protein Coding	34	GC11M003000	6.91210556	https://www.genecards.org/cgi-bin/carddisp.pl?gene=CARS1
ADAMTS9	ADAM Metallopeptidase With Thrombospondin Type 1 Motif 9	Protein Coding	38	GC03M064501	6.903200626	https://www.genecards.org/cgi-bin/carddisp.pl?gene=ADAMTS9
NDRG2	NDRG Family Member 2	Protein Coding	39	GC14M021016	6.884765625	https://www.genecards.org/cgi-bin/carddisp.pl?gene=NDRG2
CFD	Complement Factor D	Protein Coding	45	GC19P000859	6.84444046	https://www.genecards.org/cgi-bin/carddisp.pl?gene=CFD
PTBP1	Polypyrimidine Tract Binding Protein 1	Protein Coding	43	GC19P000797	6.802304268	https://www.genecards.org/cgi-bin/carddisp.pl?gene=PTBP1
SLC38A8	Solute Carrier Family 38 Member 8	Protein Coding	37	GC16M084009	6.798454762	https://www.genecards.org/cgi-bin/carddisp.pl?gene=SLC38A8
ONECUT2	One Cut Homeobox 2	Protein Coding	36	GC18P057436	6.7439394	https://www.genecards.org/cgi-bin/carddisp.pl?gene=ONECUT2
RIPK3	Receptor Interacting Serine/Threonine Kinase 3	Protein Coding	43	GC14M024336	6.739554405	https://www.genecards.org/cgi-bin/carddisp.pl?gene=RIPK3
PYCARD	PYD And CARD Domain Containing	Protein Coding	43	GC16M031201	6.707985878	https://www.genecards.org/cgi-bin/carddisp.pl?gene=PYCARD
CCL19	C-C Motif Chemokine Ligand 19	Protein Coding	42	GC09M034692	6.706582546	https://www.genecards.org/cgi-bin/carddisp.pl?gene=CCL19
RPTOR	Regulatory Associated Protein Of MTOR Complex 1	Protein Coding	44	GC17P080544	6.705212593	https://www.genecards.org/cgi-bin/carddisp.pl?gene=RPTOR
HS3ST1	Heparan Sulfate-Glucosamine 3-Sulfotransferase 1	Protein Coding	43	GC04M011394	6.701167107	https://www.genecards.org/cgi-bin/carddisp.pl?gene=HS3ST1
LCP1	Lymphocyte Cytosolic Protein 1	Protein Coding	43	GC13M046132	6.697141171	https://www.genecards.org/cgi-bin/carddisp.pl?gene=LCP1
TFAP2B	Transcription Factor AP-2 Beta	Protein Coding	45	GC06P055469	6.696252823	https://www.genecards.org/cgi-bin/carddisp.pl?gene=TFAP2B
MAP3K8	Mitogen-Activated Protein Kinase Kinase Kinase 8	Protein Coding	47	GC10P030458	6.692231178	https://www.genecards.org/cgi-bin/carddisp.pl?gene=MAP3K8
SLC25A3	Solute Carrier Family 25 Member 3	Protein Coding	45	GC12P098593	6.669864655	https://www.genecards.org/cgi-bin/carddisp.pl?gene=SLC25A3
MAPK12	Mitogen-Activated Protein Kinase 12	Protein Coding	49	GC22M050246	6.659944057	https://www.genecards.org/cgi-bin/carddisp.pl?gene=MAPK12
PNLIPRP2	Pancreatic Lipase Related Protein 2 (Gene/Pseudogene)	Protein Coding	37	GC10P116620	6.652090073	https://www.genecards.org/cgi-bin/carddisp.pl?gene=PNLIPRP2
YEATS2	YEATS Domain Containing 2	Protein Coding	37	GC03P183698	6.634466648	https://www.genecards.org/cgi-bin/carddisp.pl?gene=YEATS2
DARS1	Aspartyl-TRNA Synthetase 1	Protein Coding	37	GC02M135905	6.609959602	https://www.genecards.org/cgi-bin/carddisp.pl?gene=DARS1
H1-4	H1.4 Linker Histone, Cluster Member	Protein Coding	36	GC06P055576	6.604896069	https://www.genecards.org/cgi-bin/carddisp.pl?gene=H1-4
INSIG1	Insulin Induced Gene 1	Protein Coding	40	GC07P155297	6.590118885	https://www.genecards.org/cgi-bin/carddisp.pl?gene=INSIG1
CXCL16	C-X-C Motif Chemokine Ligand 16	Protein Coding	40	GC17M004733	6.588072777	https://www.genecards.org/cgi-bin/carddisp.pl?gene=CXCL16
LAMP3	Lysosomal Associated Membrane Protein	Protein Coding	40	GC03M183122	6.55397892	https://www.genecards.org/cgi-bin/carddisp.pl?gene=LAMP3
BIRC2	Baculoviral IAP Repeat Containing 2	Protein Coding	46	GC11P102347	6.543526173	https://www.genecards.org/cgi-bin/carddisp.pl?gene=BIRC2
SLC25A10	Solute Carrier Family 25 Member 10	Protein Coding	43	GC17P081712	6.54059124	https://www.genecards.org/cgi-bin/carddisp.pl?gene=SLC25A10
INSIG2	Insulin Induced Gene 2	Protein Coding	41	GC02P118088	6.534506798	https://www.genecards.org/cgi-bin/carddisp.pl?gene=INSIG2
H4C1	H4 Clustered Histone 1	Protein Coding	33	GC06P054981	6.511785507	https://www.genecards.org/cgi-bin/carddisp.pl?gene=H4C1
PTPRG	Protein Tyrosine Phosphatase Receptor Type G	Protein Coding	43	GC03P061561	6.509430885	https://www.genecards.org/cgi-bin/carddisp.pl?gene=PTPRG
NDUFS5	NADH:Ubiquinone Oxidoreductase Subunit S5	Protein Coding	41	GC01P039026	6.506564617	https://www.genecards.org/cgi-bin/carddisp.pl?gene=NDUFS5
BNIP3	BCL2 Interacting Protein 3	Protein Coding	43	GC10M131966	6.503369331	https://www.genecards.org/cgi-bin/carddisp.pl?gene=BNIP3
GLO1	Glyoxalase I	Protein Coding	47	GC06M047047	6.491281509	https://www.genecards.org/cgi-bin/carddisp.pl?gene=GLO1
SOCS2	Suppressor Of Cytokine Signaling 2	Protein Coding	45	GC12P093569	6.465957642	https://www.genecards.org/cgi-bin/carddisp.pl?gene=SOCS2
YBX1	Y-Box Binding Protein 1	Protein Coding	40	GC01P042682	6.453653336	https://www.genecards.org/cgi-bin/carddisp.pl?gene=YBX1
IRF2BP2	Interferon Regulatory Factor 2 Binding Protein 2	Protein Coding	36	GC01M234604	6.43283844	https://www.genecards.org/cgi-bin/carddisp.pl?gene=IRF2BP2
ATXN2L	Ataxin 2 Like	Protein Coding	38	GC16P032257	6.391595364	https://www.genecards.org/cgi-bin/carddisp.pl?gene=ATXN2L
YWHAB	Tyrosine 3-Monooxygenase/Tryptophan 5-Monooxygenase Activation Protein Beta	Protein Coding	49	GC20P044885	6.389775753	https://www.genecards.org/cgi-bin/carddisp.pl?gene=YWHAB
ATF3	Activating Transcription Factor 3	Protein Coding	45	GC01P212565	6.369270325	https://www.genecards.org/cgi-bin/carddisp.pl?gene=ATF3
PRKCE	Protein Kinase C Epsilon	Protein Coding	50	GC02P045651	6.321177483	https://www.genecards.org/cgi-bin/carddisp.pl?gene=PRKCE
NDUFA8	NADH:Ubiquinone Oxidoreductase Subunit A8	Protein Coding	43	GC09M122132	6.320979118	https://www.genecards.org/cgi-bin/carddisp.pl?gene=NDUFA8
UQCR11	Ubiquinol-Cytochrome C Reductase, Complex III Subunit XI	Protein Coding	37	GC19M001597	6.31750679	https://www.genecards.org/cgi-bin/carddisp.pl?gene=UQCR11
CCRL2	C-C Motif Chemokine Receptor Like 2	Protein Coding	40	GC03P046407	6.307687283	https://www.genecards.org/cgi-bin/carddisp.pl?gene=CCRL2
NDUFA5	NADH:Ubiquinone Oxidoreductase Subunit A5	Protein Coding	43	GC07M123536	6.300936222	https://www.genecards.org/cgi-bin/carddisp.pl?gene=NDUFA5
PCLO	Piccolo Presynaptic Cytomatrix Protein	Protein Coding	41	GC07M082754	6.297133446	https://www.genecards.org/cgi-bin/carddisp.pl?gene=PCLO
PCSK5	Proprotein Convertase Subtilisin/Kexin Type 5	Protein Coding	41	GC09P075890	6.295871735	https://www.genecards.org/cgi-bin/carddisp.pl?gene=PCSK5
MIR103A2	MicroRNA 103a-2	RNA Gene	18	GC20P003917	6.281816483	https://www.genecards.org/cgi-bin/carddisp.pl?gene=MIR103A2
CAMKK2	Calcium/Calmodulin Dependent Protein Kinase Kinase 2	Protein Coding	47	GC12M121496	6.273727894	https://www.genecards.org/cgi-bin/carddisp.pl?gene=CAMKK2
SVIL	Supervillin	Protein Coding	42	GC10M031105	6.273127556	https://www.genecards.org/cgi-bin/carddisp.pl?gene=SVIL
SIRT5	Sirtuin 5	Protein Coding	45	GC06P013574	6.262564659	https://www.genecards.org/cgi-bin/carddisp.pl?gene=SIRT5
CTBP2	C-Terminal Binding Protein 2	Protein Coding	45	GC10M124984	6.257704258	https://www.genecards.org/cgi-bin/carddisp.pl?gene=CTBP2
USP7	Ubiquitin Specific Peptidase 7	Protein Coding	49	GC16M008892	6.241252899	https://www.genecards.org/cgi-bin/carddisp.pl?gene=USP7
CYP2A13	Cytochrome P450 Family 2 Subfamily A Member 13	Protein Coding	42	GC19P041088	6.240559101	https://www.genecards.org/cgi-bin/carddisp.pl?gene=CYP2A13
TRIB3	Tribbles Pseudokinase 3	Protein Coding	44	GC20P000361	6.240134239	https://www.genecards.org/cgi-bin/carddisp.pl?gene=TRIB3
NGF-AS1	NGF Antisense RNA 1	RNA Gene	10	GC01P115280	6.172226906	https://www.genecards.org/cgi-bin/carddisp.pl?gene=NGF-AS1
TPM4	Tropomyosin 4	Protein Coding	41	GC19P026610	6.156361103	https://www.genecards.org/cgi-bin/carddisp.pl?gene=TPM4
CALD1	Caldesmon 1	Protein Coding	43	GC07P134744	6.150755405	https://www.genecards.org/cgi-bin/carddisp.pl?gene=CALD1
ESM1	Endothelial Cell Specific Molecule 1	Protein Coding	40	GC05M054977	6.138051987	https://www.genecards.org/cgi-bin/carddisp.pl?gene=ESM1
RPL13	Ribosomal Protein L13	Protein Coding	43	GC16P089802	6.135052204	https://www.genecards.org/cgi-bin/carddisp.pl?gene=RPL13
KLB	Klotho Beta	Protein Coding	40	GC04P039408	6.119310379	https://www.genecards.org/cgi-bin/carddisp.pl?gene=KLB
RPN1	Ribophorin I	Protein Coding	44	GC03M128619	6.111598969	https://www.genecards.org/cgi-bin/carddisp.pl?gene=RPN1
APLNR	Apelin Receptor	Protein Coding	44	GC11M057233	6.1106987	https://www.genecards.org/cgi-bin/carddisp.pl?gene=APLNR
RPLP2	Ribosomal Protein Lateral Stalk Subunit	Protein Coding	42	GC11P000984	6.108006477	https://www.genecards.org/cgi-bin/carddisp.pl?gene=RPLP2
NUDT4	Nudix Hydrolase 4	Protein Coding	40	GC12P093392	6.082500935	https://www.genecards.org/cgi-bin/carddisp.pl?gene=NUDT4
TET1	Tet Methylcytosine Dioxygenase 1	Protein Coding	38	GC10P068560	6.082352638	https://www.genecards.org/cgi-bin/carddisp.pl?gene=TET1
H4C11	H4 Clustered Histone 11	Protein Coding	30	GC06P055048	6.066231251	https://www.genecards.org/cgi-bin/carddisp.pl?gene=H4C11
H4C8	H4 Clustered Histone 8	Protein Coding	30	GC06M047225	6.056311607	https://www.genecards.org/cgi-bin/carddisp.pl?gene=H4C8
SLC6A9	Solute Carrier Family 6 Member 9	Protein Coding	50	GC01M043991	6.052671432	https://www.genecards.org/cgi-bin/carddisp.pl?gene=SLC6A9
SNHG20	Small Nucleolar RNA Host Gene 20	RNA Gene	17	GC17P077087	6.046751976	https://www.genecards.org/cgi-bin/carddisp.pl?gene=SNHG20
H4C4	H4 Clustered Histone 4	Protein Coding	27	GC06M047223	6.037734985	https://www.genecards.org/cgi-bin/carddisp.pl?gene=H4C4
TPT1	Tumor Protein, Translationally-Controlled	Protein Coding	47	GC13M045333	6.0363307	https://www.genecards.org/cgi-bin/carddisp.pl?gene=TPT1
RPL7A	Ribosomal Protein L7a	Protein Coding	43	GC09P133348	6.030329704	https://www.genecards.org/cgi-bin/carddisp.pl?gene=RPL7A
RPL6	Ribosomal Protein L6	Protein Coding	40	GC12M112320	6.025621891	https://www.genecards.org/cgi-bin/carddisp.pl?gene=RPL6
PTPRU	Protein Tyrosine Phosphatase Receptor Type U	Protein Coding	43	GC01P029236	6.015058994	https://www.genecards.org/cgi-bin/carddisp.pl?gene=PTPRU

FARP1	FERM, ARH/RhoGEF And Pleckstrin Domain Protein 1	Protein Coding	38	GC13P098142	6.014532089	https://www.genecards.org/cgi-bin/carddisp.pl?gene=FARP1
CBLB	Cbl Proto-Oncogene B	Protein Coding	45	GC03M105655	6.01316309	https://www.genecards.org/cgi-bin/carddisp.pl?gene=CBLB
SAT1	Spermidine/Spermine N1-	Protein Coding	46	GC0XP023784	6.010082245	https://www.genecards.org/cgi-bin/carddisp.pl?gene=SAT1
YBX3	Y-Box Binding Protein 3	Protein Coding	40	GC12M015696	6.006464005	https://www.genecards.org/cgi-bin/carddisp.pl?gene=YBX3
CCNC	Cyclin C	Protein Coding	41	GC06M099542	6.002334595	https://www.genecards.org/cgi-bin/carddisp.pl?gene=CCNC
NDUFB7	NADH:Ubiquinone Oxidoreductase Subunit B7	Protein Coding	40	GC19M014566	5.997264862	https://www.genecards.org/cgi-bin/carddisp.pl?gene=NDUFB7
IL1R2	Interleukin 1 Receptor Type 2	Protein Coding	45	GC02P101991	5.982992172	https://www.genecards.org/cgi-bin/carddisp.pl?gene=IL1R2
SRSF6	Serine And Arginine Rich Splicing Factor	Protein Coding	40	GC20P043457	5.979786873	https://www.genecards.org/cgi-bin/carddisp.pl?gene=SRSF6
YIPF1	Yip1 Domain Family Member 1	Protein Coding	36	GC01M053851	5.975258827	https://www.genecards.org/cgi-bin/carddisp.pl?gene=YIPF1
MIR33B	MicroRNA 33b	RNA Gene	18	GC17M017813	5.944622993	https://www.genecards.org/cgi-bin/carddisp.pl?gene=MIR33B
PABPC1	Poly(A) Binding Protein Cytoplasmic 1	Protein Coding	43	GC08M100685	5.94400692	https://www.genecards.org/cgi-bin/carddisp.pl?gene=PABPC1
HNRNPH1	Heterogeneous Nuclear Ribonucleoprotein H1	Protein Coding	40	GC05M179614	5.939538002	https://www.genecards.org/cgi-bin/carddisp.pl?gene=HNRNPH1
TSBP1-AS1	TSBP1 And BTNL2 Antisense RNA 1	RNA Gene	9	GC06P058058	5.935389042	https://www.genecards.org/cgi-bin/carddisp.pl?gene=TSBP1-AS1
NFE2L1	Nuclear Factor, Erythroid 2 Like 1	Protein Coding	44	GC17P048049	5.928246498	https://www.genecards.org/cgi-bin/carddisp.pl?gene=NFE2L1
RPS11	Ribosomal Protein S11	Protein Coding	40	GC19P049496	5.921973705	https://www.genecards.org/cgi-bin/carddisp.pl?gene=RPS11
H4C9	H4 Clustered Histone 9	Protein Coding	30	GC06P055592	5.885432243	https://www.genecards.org/cgi-bin/carddisp.pl?gene=H4C9
IL34	Interleukin 34	Protein Coding	40	GC16P070656	5.87556076	https://www.genecards.org/cgi-bin/carddisp.pl?gene=IL34
AP2B1	Adaptor Related Protein Complex 2 Subunit Beta 1	Protein Coding	43	GC17P035578	5.87352562	https://www.genecards.org/cgi-bin/carddisp.pl?gene=AP2B1
RPS18	Ribosomal Protein S18	Protein Coding	41	GC06P055262	5.869342804	https://www.genecards.org/cgi-bin/carddisp.pl?gene=RPS18
AQP7	Aquaporin 7	Protein Coding	45	GC09M033384	5.860530853	https://www.genecards.org/cgi-bin/carddisp.pl?gene=AQP7
SLC3A2	Solute Carrier Family 3 Member 2	Protein Coding	43	GC11P062856	5.83400631	https://www.genecards.org/cgi-bin/carddisp.pl?gene=SLC3A2
H4C3	H4 Clustered Histone 3	Protein Coding	30	GC06P054986	5.831675529	https://www.genecards.org/cgi-bin/carddisp.pl?gene=H4C3
MCM3	Minichromosome Maintenance Complex Component 3	Protein Coding	46	GC06M052264	5.821012497	https://www.genecards.org/cgi-bin/carddisp.pl?gene=MCM3
ST8SIA1	ST8 Alpha-N-Acetyl-Neuraminide Alpha-2,8-Sialyltransferase 1	Protein Coding	41	GC12M022063	5.802177429	https://www.genecards.org/cgi-bin/carddisp.pl?gene=ST8SIA1
H4C2	H4 Clustered Histone 2	Protein Coding	31	GC06M026026	5.790835381	https://www.genecards.org/cgi-bin/carddisp.pl?gene=H4C2
H4C13	H4 Clustered Histone 13	Protein Coding	29	GC06M047221	5.790835381	https://www.genecards.org/cgi-bin/carddisp.pl?gene=H4C13
H4C6	H4 Clustered Histone 6	Protein Coding	29	GC06P055591	5.787128448	https://www.genecards.org/cgi-bin/carddisp.pl?gene=H4C6
ROCK1	Rho Associated Coiled-Coil Containing Protein Kinase 1	Protein Coding	51	GC18M020946	5.780395508	https://www.genecards.org/cgi-bin/carddisp.pl?gene=ROCK1
H4C14	H4 Clustered Histone 14	Protein Coding	29	GC01P149832	5.77880764	https://www.genecards.org/cgi-bin/carddisp.pl?gene=H4C14
H4C15	H4 Clustered Histone 15	Protein Coding	27	GC01M150133	5.77880764	https://www.genecards.org/cgi-bin/carddisp.pl?gene=H4C15
H4C12	H4 Clustered Histone 12	Protein Coding	29	GC06M047220	5.775533676	https://www.genecards.org/cgi-bin/carddisp.pl?gene=H4C12
EHBP1L1	EH Domain Binding Protein 1 Like 1	Protein Coding	32	GC11P066231	5.773977757	https://www.genecards.org/cgi-bin/carddisp.pl?gene=EHBP1L1
PRKAG3	Protein Kinase AMP-Activated Non-Catalytic Subunit Gamma 3	Protein Coding	44	GC02M218823	5.771601677	https://www.genecards.org/cgi-bin/carddisp.pl?gene=PRKAG3
H4C5	H4 Clustered Histone 5	Protein Coding	28	GC06P055590	5.76215744	https://www.genecards.org/cgi-bin/carddisp.pl?gene=H4C5
BCAT1	Branched Chain Amino Acid	Protein Coding	47	GC12M024732	5.727366924	https://www.genecards.org/cgi-bin/carddisp.pl?gene=BCAT1
FAP	Fibroblast Activation Protein Alpha	Protein Coding	43	GC02M162170	5.723641872	https://www.genecards.org/cgi-bin/carddisp.pl?gene=FAP
ESRRG	Estrogen Related Receptor Gamma	Protein Coding	46	GC01M216503	5.6957798	https://www.genecards.org/cgi-bin/carddisp.pl?gene=ESRRG
FRK	Fyn Related Src Family Tyrosine Kinase	Protein Coding	45	GC06M115931	5.694391251	https://www.genecards.org/cgi-bin/carddisp.pl?gene=FRK
UQCR10	Ubiquinol-Cytochrome C Reductase, Complex III Subunit X	Protein Coding	40	GC22P029767	5.678407669	https://www.genecards.org/cgi-bin/carddisp.pl?gene=UQCR10
CYP2F1	Cytochrome P450 Family 2 Subfamily F Member 1	Protein Coding	43	GC19P041114	5.677024841	https://www.genecards.org/cgi-bin/carddisp.pl?gene=CYP2F1
LAPTM4A	Lysosomal Protein Transmembrane 4	Protein Coding	38	GC02M020032	5.6571064	https://www.genecards.org/cgi-bin/carddisp.pl?gene=LAPTM4A
RPL10A	Ribosomal Protein L10a	Protein Coding	43	GC06P035328	5.652759552	https://www.genecards.org/cgi-bin/carddisp.pl?gene=RPL10A
NCL	Nucleolin	Protein Coding	43	GC02M231453	5.64723587	https://www.genecards.org/cgi-bin/carddisp.pl?gene=NCL
TTF2	Transcription Termination Factor 2	Protein Coding	40	GC01P117060	5.64432478	https://www.genecards.org/cgi-bin/carddisp.pl?gene=TTF2
SLC46A3	Solute Carrier Family 46 Member 3	Protein Coding	37	GC13M028700	5.642365932	https://www.genecards.org/cgi-bin/carddisp.pl?gene=SLC46A3
TNR	Tenascin R	Protein Coding	43	GC01M175291	5.59887886	https://www.genecards.org/cgi-bin/carddisp.pl?gene=TNR
NDUFB6	NADH:Ubiquinone Oxidoreductase Subunit B6	Protein Coding	40	GC09M032553	5.591681957	https://www.genecards.org/cgi-bin/carddisp.pl?gene=NDUFB6
FETUB	Fetuin B	Protein Coding	37	GC03P186635	5.586373329	https://www.genecards.org/cgi-bin/carddisp.pl?gene=FETUB
RPL3	Ribosomal Protein L3	Protein Coding	42	GC22M048526	5.510959625	https://www.genecards.org/cgi-bin/carddisp.pl?gene=RPL3
RPL12	Ribosomal Protein L12	Protein Coding	43	GC09M127447	5.504228115	https://www.genecards.org/cgi-bin/carddisp.pl?gene=RPL12
MRTFA	Myocardin Related Transcription Factor A	Protein Coding	35	GC22M049024	5.501933575	https://www.genecards.org/cgi-bin/carddisp.pl?gene=MRTFA
CTSS	Cathepsin S	Protein Coding	46	GC01M150730	5.498209953	https://www.genecards.org/cgi-bin/carddisp.pl?gene=CTSS
AEBP1	AE Binding Protein 1	Protein Coding	41	GC07P044106	5.477787018	https://www.genecards.org/cgi-bin/carddisp.pl?gene=AEBP1
PDGFC	Platelet Derived Growth Factor C	Protein Coding	44	GC04M156760	5.476284027	https://www.genecards.org/cgi-bin/carddisp.pl?gene=PDGFC
PFKFB3	6-Phosphofructo-2-Kinase/Fructose-2,6-Biphosphatase 3	Protein Coding	45	GC10P006144	5.470271111	https://www.genecards.org/cgi-bin/carddisp.pl?gene=PFKFB3
SUGP1	SURP And G-Patch Domain Containing 1	Protein Coding	35	GC19M019276	5.46651125	https://www.genecards.org/cgi-bin/carddisp.pl?gene=SUGP1
FAF2	Fas Associated Factor Family Member 2	Protein Coding	38	GC05P176447	5.463183403	https://www.genecards.org/cgi-bin/carddisp.pl?gene=FAF2
RPS23	Ribosomal Protein S23	Protein Coding	41	GC05M082273	5.451299191	https://www.genecards.org/cgi-bin/carddisp.pl?gene=RPS23
YWHAZ	Tyrosine 3-Monooxygenase/Tryptophan 5-Monooxygenase Activation Protein Zeta	Protein Coding	48	GC08M100917	5.445477486	https://www.genecards.org/cgi-bin/carddisp.pl?gene=YWHAZ
CWF19L1	CWF19 Like Cell Cycle Control Factor 1	Protein Coding	39	GC10M100232	5.412953854	https://www.genecards.org/cgi-bin/carddisp.pl?gene=CWF19L1
HSP90AB1	Heat Shock Protein 90 Alpha Family Class B Member 1	Protein Coding	46	GC06P044246	5.409389496	https://www.genecards.org/cgi-bin/carddisp.pl?gene=HSP90AB1
HNRNPF	Heterogeneous Nuclear Ribonucleoprotein	Protein Coding	41	GC10M043385	5.407585621	https://www.genecards.org/cgi-bin/carddisp.pl?gene=HNRNPF
GCN1	GCN1 Activator Of EIF2AK4	Protein Coding	29	GC12M120128	5.405367851	https://www.genecards.org/cgi-bin/carddisp.pl?gene=GCN1
PCBP2	Poly(RC) Binding Protein 2	Protein Coding	42	GC12P053452	5.392615318	https://www.genecards.org/cgi-bin/carddisp.pl?gene=PCBP2
ZP4	Zona Pellucida Glycoprotein 4	Protein Coding	33	GC01M237877	5.388515472	https://www.genecards.org/cgi-bin/carddisp.pl?gene=ZP4
RPL30	Ribosomal Protein L30	Protein Coding	41	GC08M098024	5.386371613	https://www.genecards.org/cgi-bin/carddisp.pl?gene=RPL30
NMT2	N-Myristoyltransferase 2	Protein Coding	41	GC10M015115	5.380367279	https://www.genecards.org/cgi-bin/carddisp.pl?gene=NMT2
COX5B	Cytochrome C Oxidase Subunit 5B	Protein Coding	42	GC02P097628	5.375883102	https://www.genecards.org/cgi-bin/carddisp.pl?gene=COX5B
INHBA	Inhibin Subunit Beta A	Protein Coding	45	GC07M041668	5.367442131	https://www.genecards.org/cgi-bin/carddisp.pl?gene=INHBA
PCBP1	Poly(RC) Binding Protein 1	Protein Coding	43	GC02P070087	5.364738941	https://www.genecards.org/cgi-bin/carddisp.pl?gene=PCBP1
COX7A2L	Cytochrome C Oxidase Subunit 7A2 Like	Protein Coding	42	GC02M042312	5.352900505	https://www.genecards.org/cgi-bin/carddisp.pl?gene=COX7A2L
HLX	H2.0 Like Homeobox	Protein Coding	41	GC01P220879	5.319946766	https://www.genecards.org/cgi-bin/carddisp.pl?gene=HLX
MIR372	MicroRNA 372	RNA Gene	16	GC19P056407	5.312681198	https://www.genecards.org/cgi-bin/carddisp.pl?gene=MIR372
CLRN1-AS1	CLRN1 Antisense RNA 1	RNA Gene	17	GC03P150852	5.305912971	https://www.genecards.org/cgi-bin/carddisp.pl?gene=CLRN1-AS1
FAM107B	Family With Sequence Similarity 107 Member B	Protein Coding	34	GC10M014519	5.301181793	https://www.genecards.org/cgi-bin/carddisp.pl?gene=FAM107B
DNAJA1	DnaJ Heat Shock Protein Family (Hsp40) Member A1	Protein Coding	42	GC09P033025	5.294562817	https://www.genecards.org/cgi-bin/carddisp.pl?gene=DNAJA1
DISC2	Disrupted In Schizophrenia 2	RNA Gene	19	GC01M231814	5.283132076	https://www.genecards.org/cgi-bin/carddisp.pl?gene=DISC2
PPP1CA	Protein Phosphatase 1 Catalytic Subunit Alpha	Protein Coding	49	GC11M069512	5.275168419	https://www.genecards.org/cgi-bin/carddisp.pl?gene=PPP1CA
ANXA9	Annexin A9	Protein Coding	39	GC01P150982	5.275134087	https://www.genecards.org/cgi-bin/carddisp.pl?gene=ANXA9
SORCS2	Sortilin Related VPS10 Domain Containing Receptor 2	Protein Coding	36	GC04P007196	5.270096779	https://www.genecards.org/cgi-bin/carddisp.pl?gene=SORCS2
POMP	Proteasome Maturation Protein	Protein Coding	43	GC13P028659	5.263608932	https://www.genecards.org/cgi-bin/carddisp.pl?gene=POMP
HNRNPM	Heterogeneous Nuclear Ribonucleoprotein	Protein Coding	40	GC19P008444	5.252953529	https://www.genecards.org/cgi-bin/carddisp.pl?gene=HNRNPM
GGT2	Gamma-Glutamyltransferase 2	Protein Coding	31	GC22M021207	5.252270699	https://www.genecards.org/cgi-bin/carddisp.pl?gene=GGT2
TMBIM1	Transmembrane BAX Inhibitor Motif Containing 1	Protein Coding	36	GC02M218274	5.24852562	https://www.genecards.org/cgi-bin/carddisp.pl?gene=TMBIM1
RPS2	Ribosomal Protein S2	Protein Coding	44	GC16M003048	5.240426064	https://www.genecards.org/cgi-bin/carddisp.pl?gene=RPS2
SLC25A11	Solute Carrier Family 25 Member 11	Protein Coding	44	GC17M004937	5.220676422	https://www.genecards.org/cgi-bin/carddisp.pl?gene=SLC25A11
GPN1	GPN-Loop GTPase 1	Protein Coding	38	GC02P027628	5.217983723	https://www.genecards.org/cgi-bin/carddisp.pl?gene=GPN1
CREB5	CAMP Responsive Element Binding Protein 5	Protein Coding	41	GC07P028305	5.2126894	https://www.genecards.org/cgi-bin/carddisp.pl?gene=CREB5

SERPINA4	Serpin Family A Member 4	Protein Coding	41	GC14P094561	5.211485863	https://www.genecards.org/cgi-bin/carddisp.pl?gene=SERPINA4
NDUFA3	NADH:Ubiquinone Oxidoreductase Subunit A3	Protein Coding	39	GC19P054102	5.203584194	https://www.genecards.org/cgi-bin/carddisp.pl?gene=NDUFA3
RPS9	Ribosomal Protein S9	Protein Coding	43	GC19P056806	5.202595234	https://www.genecards.org/cgi-bin/carddisp.pl?gene=RPS9
BICD1	BICD Cargo Adaptor 1	Protein Coding	40	GC12P032107	5.194260597	https://www.genecards.org/cgi-bin/carddisp.pl?gene=BICD1
MYO18A	Myosin XVIIIA	Protein Coding	38	GC17M031089	5.183431625	https://www.genecards.org/cgi-bin/carddisp.pl?gene=MYO18A
NRG4	Neuregulin 4	Protein Coding	40	GC15M075935	5.170539856	https://www.genecards.org/cgi-bin/carddisp.pl?gene=NRG4
HTR1B	5-Hydroxytryptamine Receptor 1B	Protein Coding	45	GC06M077478	5.16864109	https://www.genecards.org/cgi-bin/carddisp.pl?gene=HTR1B
HSPH1	Heat Shock Protein Family H (Hsp110) Member 1	Protein Coding	44	GC13M031134	5.168605804	https://www.genecards.org/cgi-bin/carddisp.pl?gene=HSPH1
MIR449A	MicroRNA 449a	RNA Gene	20	GC05M055171	5.165687561	https://www.genecards.org/cgi-bin/carddisp.pl?gene=MIR449A
IGF2BP1	Insulin Like Growth Factor 2 MRNA Binding Protein 1	Protein Coding	41	GC17P048997	5.164940834	https://www.genecards.org/cgi-bin/carddisp.pl?gene=IGF2BP1
TRIM33	Tripartite Motif Containing 33	Protein Coding	44	GC01M114392	5.159249306	https://www.genecards.org/cgi-bin/carddisp.pl?gene=TRIM33
DDX1	DEAD-Box Helicase 1	Protein Coding	44	GC02P015591	5.139266014	https://www.genecards.org/cgi-bin/carddisp.pl?gene=DDX1
CTPS1	CTP Synthase 1	Protein Coding	45	GC01P040979	5.113494873	https://www.genecards.org/cgi-bin/carddisp.pl?gene=CTPS1
VIL1	Villin 1	Protein Coding	40	GC02P218419	5.110589027	https://www.genecards.org/cgi-bin/carddisp.pl?gene=VIL1
AGAP1	ArfGAP With GTPase Domain, Ankyrin Repeat And PH Domain 1	Protein Coding	41	GC02P235494	5.070719242	https://www.genecards.org/cgi-bin/carddisp.pl?gene=AGAP1
HNRNPA3	Heterogeneous Nuclear Ribonucleoprotein A3	Protein Coding	38	GC02P177212	5.062342644	https://www.genecards.org/cgi-bin/carddisp.pl?gene=HNRNPA3
TUBA8	Tubulin Alpha 8	Protein Coding	45	GC22P018110	5.061789989	https://www.genecards.org/cgi-bin/carddisp.pl?gene=TUBA8
CCAR2	Cell Cycle And Apoptosis Regulator 2	Protein Coding	37	GC08P022604	5.053204536	https://www.genecards.org/cgi-bin/carddisp.pl?gene=CCAR2
AHSA1	Activator Of HSP90 ATPase Activity 1	Protein Coding	40	GC14P077457	5.047645569	https://www.genecards.org/cgi-bin/carddisp.pl?gene=AHSA1
RANGAP1	Ran GTPase Activating Protein 1	Protein Coding	43	GC22M041244	5.045892715	https://www.genecards.org/cgi-bin/carddisp.pl?gene=RANGAP1
DHX9	DExH-Box Helicase 9	Protein Coding	41	GC01P182839	5.045092583	https://www.genecards.org/cgi-bin/carddisp.pl?gene=DHX9
HNRNPUL1	Heterogeneous Nuclear Ribonucleoprotein U Like 1	Protein Coding	38	GC19P041262	5.025044441	https://www.genecards.org/cgi-bin/carddisp.pl?gene=HNRNPUL1
TMSB4X	Thymosin Beta 4 X-Linked	Protein Coding	40	GC0XP012975	5.0191679	https://www.genecards.org/cgi-bin/carddisp.pl?gene=TMSB4X
CRTC1	CREB Regulated Transcription	Protein Coding	43	GC19P026660	5.003367901	https://www.genecards.org/cgi-bin/carddisp.pl?gene=CRTC1
TNFRSF12A	TNF Receptor Superfamily Member 12A	Protein Coding	44	GC16P003018	5.002279282	https://www.genecards.org/cgi-bin/carddisp.pl?gene=TNFRSF12A
TARBP1	TAR (HIV-1) RNA Binding Protein 1	Protein Coding	39	GC01M234391	4.99900341	https://www.genecards.org/cgi-bin/carddisp.pl?gene=TARBP1
LEPROT	Leptin Receptor Overlapping Transcript	Protein Coding	33	GC01P065420	4.961866379	https://www.genecards.org/cgi-bin/carddisp.pl?gene=LEPROT
NFE2	Nuclear Factor, Erythroid 2	Protein Coding	41	GC12M054292	4.958201885	https://www.genecards.org/cgi-bin/carddisp.pl?gene=NFE2
BBC3	BCL2 Binding Component 3	Protein Coding	41	GC19M047220	4.95677948	https://www.genecards.org/cgi-bin/carddisp.pl?gene=BBC3
ITSN1	Intersectin 1	Protein Coding	42	GC21P033642	4.940186977	https://www.genecards.org/cgi-bin/carddisp.pl?gene=ITSN1
COX6C	Cytochrome C Oxidase Subunit 6C	Protein Coding	40	GC08M099899	4.936024189	https://www.genecards.org/cgi-bin/carddisp.pl?gene=COX6C
NDUFB4	NADH:Ubiquinone Oxidoreductase Subunit B4	Protein Coding	41	GC03P120596	4.918389797	https://www.genecards.org/cgi-bin/carddisp.pl?gene=NDUFB4
DDX5	DEAD-Box Helicase 5	Protein Coding	47	GC17M064498	4.91580677	https://www.genecards.org/cgi-bin/carddisp.pl?gene=DDX5
POLR3H	RNA Polymerase III Subunit H	Protein Coding	38	GC22M041525	4.915407658	https://www.genecards.org/cgi-bin/carddisp.pl?gene=POLR3H
APPL2	Adaptor Protein, Phosphotyrosine Interacting With PH Domain And Leucine	Protein Coding	36	GC12M105173	4.913758278	https://www.genecards.org/cgi-bin/carddisp.pl?gene=APPL2
RPS4X	Ribosomal Protein S4 X-Linked	Protein Coding	40	GC0XM072255	4.906749725	https://www.genecards.org/cgi-bin/carddisp.pl?gene=RPS4X
PNPLA7	Patatin Like Phospholipase Domain Containing 7	Protein Coding	34	GC09M137459	4.905105591	https://www.genecards.org/cgi-bin/carddisp.pl?gene=PNPLA7
DDX60L	DExD/H-Box 60 Like	Protein Coding	33	GC04M168356	4.903104782	https://www.genecards.org/cgi-bin/carddisp.pl?gene=DDX60L
PSG1	Pregnancy Specific Beta-1-Glycoprotein 1	Protein Coding	39	GC19M042866	4.901046753	https://www.genecards.org/cgi-bin/carddisp.pl?gene=PSG1
FOXK1	Forkhead Box K1	Protein Coding	35	GC07P004682	4.887200832	https://www.genecards.org/cgi-bin/carddisp.pl?gene=FOXK1
MCM5	Minichromosome Maintenance Complex Component 5	Protein Coding	46	GC22P035400	4.884897232	https://www.genecards.org/cgi-bin/carddisp.pl?gene=MCM5
RPS3A	Ribosomal Protein S3A	Protein Coding	42	GC04P151099	4.882931709	https://www.genecards.org/cgi-bin/carddisp.pl?gene=RPS3A
RPL4	Ribosomal Protein L4	Protein Coding	43	GC15M066498	4.865651131	https://www.genecards.org/cgi-bin/carddisp.pl?gene=RPL4
RPL8	Ribosomal Protein L8	Protein Coding	42	GC08M144821	4.859796047	https://www.genecards.org/cgi-bin/carddisp.pl?gene=RPL8
HNRNPL	Heterogeneous Nuclear Ribonucleoprotein	Protein Coding	40	GC19M038836	4.852230072	https://www.genecards.org/cgi-bin/carddisp.pl?gene=HNRNPL
RPL9	Ribosomal Protein L9	Protein Coding	43	GC04M039452	4.850320816	https://www.genecards.org/cgi-bin/carddisp.pl?gene=RPL9
MCM7	Minichromosome Maintenance Complex Component 7	Protein Coding	45	GC07M100092	4.847979546	https://www.genecards.org/cgi-bin/carddisp.pl?gene=MCM7
C1QTNF1	C1q And TNF Related 1	Protein Coding	40	GC17P079022	4.838021278	https://www.genecards.org/cgi-bin/carddisp.pl?gene=C1QTNF1
SLC23A1	Solute Carrier Family 23 Member 1	Protein Coding	43	GC05M139377	4.825046539	https://www.genecards.org/cgi-bin/carddisp.pl?gene=SLC23A1
CCN4	Cellular Communication Network Factor 4	Protein Coding	31	GC08P133192	4.817196369	https://www.genecards.org/cgi-bin/carddisp.pl?gene=CCN4
POLR2D	RNA Polymerase II Subunit D	Protein Coding	43	GC02M128042	4.811548233	https://www.genecards.org/cgi-bin/carddisp.pl?gene=POLR2D
SEL1L3	SEL1L Family Member 3	Protein Coding	33	GC04M025715	4.793689728	https://www.genecards.org/cgi-bin/carddisp.pl?gene=SEL1L3
STK24	Serine/Threonine Kinase 24	Protein Coding	45	GC13M098445	4.78702879	https://www.genecards.org/cgi-bin/carddisp.pl?gene=STK24
PGAM5	PGAM Family Member 5, Mitochondrial Serine/Threonine Protein Phosphatase	Protein Coding	34	GC12P132710	4.785676479	https://www.genecards.org/cgi-bin/carddisp.pl?gene=PGAM5
STX17	Syntaxin 17	Protein Coding	36	GC09P099906	4.774443626	https://www.genecards.org/cgi-bin/carddisp.pl?gene=STX17
AP2A2	Adaptor Related Protein Complex 2 Subunit Alpha 2	Protein Coding	38	GC11P000924	4.773753166	https://www.genecards.org/cgi-bin/carddisp.pl?gene=AP2A2
CCT4	Chaperonin Containing TCP1 Subunit 4	Protein Coding	40	GC02M061868	4.761928558	https://www.genecards.org/cgi-bin/carddisp.pl?gene=CCT4
MCF2L2	MCF.2 Cell Line Derived Transforming Sequence-Like 2	Protein Coding	34	GC03M183183	4.754241943	https://www.genecards.org/cgi-bin/carddisp.pl?gene=MCF2L2
NDUFB5	NADH:Ubiquinone Oxidoreductase Subunit B5	Protein Coding	37	GC03P179604	4.746250153	https://www.genecards.org/cgi-bin/carddisp.pl?gene=NDUFB5
TXNL1	Thioredoxin Like 1	Protein Coding	40	GC18M056597	4.729324341	https://www.genecards.org/cgi-bin/carddisp.pl?gene=TXNL1
STMP1	Short Transmembrane Mitochondrial Protein 1	Protein Coding	22	GC07P135671	4.714742661	https://www.genecards.org/cgi-bin/carddisp.pl?gene=STMP1
MYO1D	Myosin ID	Protein Coding	40	GC17M032492	4.709003448	https://www.genecards.org/cgi-bin/carddisp.pl?gene=MYO1D
QARS1	Glutaminyl-TRNA Synthetase 1	Protein Coding	26	GC03M049445	4.708961487	https://www.genecards.org/cgi-bin/carddisp.pl?gene=QARS1
UQCRH	Ubiquinol-Cytochrome C Reductase Hinge Protein	Protein Coding	38	GC01P046303	4.708562851	https://www.genecards.org/cgi-bin/carddisp.pl?gene=UQCRH
NDUFB2	NADH:Ubiquinone Oxidoreductase Subunit B2	Protein Coding	40	GC07P140690	4.6989398	https://www.genecards.org/cgi-bin/carddisp.pl?gene=NDUFB2
SNRPD2	Small Nuclear Ribonucleoprotein D2 Polypeptide	Protein Coding	41	GC19M046999	4.662291527	https://www.genecards.org/cgi-bin/carddisp.pl?gene=SNRPD2
LOC110366354	CYP3A4 5' Regulatory Region	Biological Region	1	GC07P099784	4.662278175	https://www.genecards.org/cgi-bin/carddisp.pl?gene=LOC110366354
CAND1	Cullin Associated And Neddylation Dissociated 1	Protein Coding	39	GC12P067270	4.631176949	https://www.genecards.org/cgi-bin/carddisp.pl?gene=CAND1
SLC23A2	Solute Carrier Family 23 Member 2	Protein Coding	43	GC20M004852	4.628893852	https://www.genecards.org/cgi-bin/carddisp.pl?gene=SLC23A2
ORM1	Orosomucoid 1	Protein Coding	40	GC09P114323	4.628304482	https://www.genecards.org/cgi-bin/carddisp.pl?gene=ORM1
RPL24	Ribosomal Protein L24	Protein Coding	40	GC03M101681	4.617097855	https://www.genecards.org/cgi-bin/carddisp.pl?gene=RPL24
ILF3	Interleukin Enhancer Binding Factor 3	Protein Coding	39	GC19P010625	4.615589619	https://www.genecards.org/cgi-bin/carddisp.pl?gene=ILF3
CRACR2A	Calcium Release Activated Channel Regulator 2A	Protein Coding	31	GC12M004349	4.615138054	https://www.genecards.org/cgi-bin/carddisp.pl?gene=CRACR2A
ARRDC3	Arrestin Domain Containing 3	Protein Coding	38	GC05M091368	4.601058006	https://www.genecards.org/cgi-bin/carddisp.pl?gene=ARRDC3
SLC30A6	Solute Carrier Family 30 Member 6	Protein Coding	39	GC02P032166	4.592472553	https://www.genecards.org/cgi-bin/carddisp.pl?gene=SLC30A6
ACTR2	Actin Related Protein 2	Protein Coding	44	GC02P065227	4.58913517	https://www.genecards.org/cgi-bin/carddisp.pl?gene=ACTR2
RUVBL1	RuvB Like AAA ATPase 1	Protein Coding	45	GC03M128064	4.578635216	https://www.genecards.org/cgi-bin/carddisp.pl?gene=RUVBL1
EDC4	Enhancer Of MRNA Decapping 4	Protein Coding	39	GC16P067873	4.578551292	https://www.genecards.org/cgi-bin/carddisp.pl?gene=EDC4
G0S2	G0/G1 Switch 2	Protein Coding	33	GC01P209675	4.57546711	https://www.genecards.org/cgi-bin/carddisp.pl?gene=G0S2
RNF114	Ring Finger Protein 114	Protein Coding	40	GC20P049936	4.564092636	https://www.genecards.org/cgi-bin/carddisp.pl?gene=RNF114
HKDC1	Hexokinase Domain Containing 1	Protein Coding	41	GC10P069220	4.553481102	https://www.genecards.org/cgi-bin/carddisp.pl?gene=HKDC1
NDUFV3	NADH:Ubiquinone Oxidoreductase Subunit V3	Protein Coding	41	GC21P042879	4.547256947	https://www.genecards.org/cgi-bin/carddisp.pl?gene=NDUFV3
EIF4A3	Eukaryotic Translation Initiation Factor	Protein Coding	44	GC17M080135	4.542392731	https://www.genecards.org/cgi-bin/carddisp.pl?gene=EIF4A3
TP53BP2	Tumor Protein P53 Binding Protein 2	Protein Coding	43	GC01M223779	4.541913986	https://www.genecards.org/cgi-bin/carddisp.pl?gene=TP53BP2

TLCD4-RWDD3	TLCD4-RWDD3 Readthrough	Protein Coding	16	GC01P095119	4.539422035	https://www.genecards.org/cgi-bin/carddisp.pl?gene=TLCD4-RWDD3
PLPPR4	Phospholipid Phosphatase Related 4	Protein Coding	31	GC01P099262	4.529919624	https://www.genecards.org/cgi-bin/carddisp.pl?gene=PLPPR4
COX7C	Cytochrome C Oxidase Subunit 7C	Protein Coding	38	GC05P086617	4.528826714	https://www.genecards.org/cgi-bin/carddisp.pl?gene=COX7C
PDIA4	Protein Disulfide Isomerase Family A Member 4	Protein Coding	43	GC07M149003	4.506326199	https://www.genecards.org/cgi-bin/carddisp.pl?gene=PDIA4
NDUFC1	NADH:Ubiquinone Oxidoreductase Subunit C1	Protein Coding	36	GC04M139267	4.505256176	https://www.genecards.org/cgi-bin/carddisp.pl?gene=NDUFC1
SERPINB4	Serpin Family B Member 4	Protein Coding	39	GC18M063637	4.496322632	https://www.genecards.org/cgi-bin/carddisp.pl?gene=SERPINB4
NUCB2	Nucleobindin 2	Protein Coding	39	GC11P017237	4.483721733	https://www.genecards.org/cgi-bin/carddisp.pl?gene=NUCB2
MIR873	MicroRNA 873	RNA Gene	16	GC09M028880	4.47608757	https://www.genecards.org/cgi-bin/carddisp.pl?gene=MIR873
SFRP5	Secreted Frizzled Related Protein 5	Protein Coding	40	GC10M097766	4.475298405	https://www.genecards.org/cgi-bin/carddisp.pl?gene=SFRP5
SLC30A9	Solute Carrier Family 30 Member 9	Protein Coding	42	GC04P041992	4.474620819	https://www.genecards.org/cgi-bin/carddisp.pl?gene=SLC30A9
COX7A1	Cytochrome C Oxidase Subunit 7A1	Protein Coding	37	GC19M047084	4.4688344	https://www.genecards.org/cgi-bin/carddisp.pl?gene=COX7A1
SRSF7	Serine And Arginine Rich Splicing Factor	Protein Coding	40	GC02M038743	4.464326859	https://www.genecards.org/cgi-bin/carddisp.pl?gene=SRSF7
PKNOX1	PBX/Knotted 1 Homeobox 1	Protein Coding	41	GC21P042974	4.461466312	https://www.genecards.org/cgi-bin/carddisp.pl?gene=PKNOX1
SP4	Sp4 Transcription Factor	Protein Coding	42	GC07P021434	4.449212074	https://www.genecards.org/cgi-bin/carddisp.pl?gene=SP4
TUBA1C	Tubulin Alpha 1c	Protein Coding	41	GC12P049188	4.431495667	https://www.genecards.org/cgi-bin/carddisp.pl?gene=TUBA1C
SEC16B	SEC16 Homolog B, Endoplasmic Reticulum Export Factor	Protein Coding	38	GC01M177923	4.420809269	https://www.genecards.org/cgi-bin/carddisp.pl?gene=SEC16B
CILP2	Cartilage Intermediate Layer Protein 2	Protein Coding	37	GC19P019538	4.416835785	https://www.genecards.org/cgi-bin/carddisp.pl?gene=CILP2
EIF4A1	Eukaryotic Translation Initiation Factor	Protein Coding	45	GC17P007572	4.408568859	https://www.genecards.org/cgi-bin/carddisp.pl?gene=EIF4A1
GATAD2A	GATA Zinc Finger Domain Containing 2A	Protein Coding	39	GC19P026663	4.395526886	https://www.genecards.org/cgi-bin/carddisp.pl?gene=GATAD2A
AP2A1	Adaptor Related Protein Complex 2 Subunit Alpha 1	Protein Coding	40	GC19P049766	4.384301186	https://www.genecards.org/cgi-bin/carddisp.pl?gene=AP2A1
ZNF512	Zinc Finger Protein 512	Protein Coding	32	GC02P027582	4.382470131	https://www.genecards.org/cgi-bin/carddisp.pl?gene=ZNF512
PABIR1	PP2A Aalpha (PPP2R1A) And B55A (PPP2R2A) Interacting Phosphatase Regulator 1	Protein Coding	25	GC09P068781	4.370363235	https://www.genecards.org/cgi-bin/carddisp.pl?gene=PABIR1
MTARC1	Mitochondrial Amidoxime Reducing Component 1	Protein Coding	28	GC01P220788	4.330311775	https://www.genecards.org/cgi-bin/carddisp.pl?gene=MTARC1
NDUFA7	NADH:Ubiquinone Oxidoreductase Subunit A7	Protein Coding	38	GC19M008308	4.325894356	https://www.genecards.org/cgi-bin/carddisp.pl?gene=NDUFA7
SENP3	SUMO Specific Peptidase 3	Protein Coding	40	GC17P009106	4.298048973	https://www.genecards.org/cgi-bin/carddisp.pl?gene=SENP3
LOC101928118	Uncharacterized LOC101928118	RNA Gene	11	GC01M095163	4.28229332	https://www.genecards.org/cgi-bin/carddisp.pl?gene=LOC101928118
ACTR3	Actin Related Protein 3	Protein Coding	43	GC02P113889	4.277811527	https://www.genecards.org/cgi-bin/carddisp.pl?gene=ACTR3
GRIK3	Glutamate Ionotropic Receptor Kainate Type Subunit 3	Protein Coding	43	GC01M036795	4.268043041	https://www.genecards.org/cgi-bin/carddisp.pl?gene=GRIK3
SIRT7	Sirtuin 7	Protein Coding	42	GC17M081911	4.255383492	https://www.genecards.org/cgi-bin/carddisp.pl?gene=SIRT7
MIR1296	MicroRNA 1296	RNA Gene	16	GC10M063372	4.224890232	https://www.genecards.org/cgi-bin/carddisp.pl?gene=MIR1296
C2orf16	Chromosome 2 Open Reading Frame 16	Protein Coding	27	GC02P027611	4.216482639	https://www.genecards.org/cgi-bin/carddisp.pl?gene=C2orf16
CCT8	Chaperonin Containing TCP1 Subunit 8	Protein Coding	40	GC21M029055	4.197618484	https://www.genecards.org/cgi-bin/carddisp.pl?gene=CCT8
RWDD3	RWD Domain Containing 3	Protein Coding	36	GC01P095171	4.196980476	https://www.genecards.org/cgi-bin/carddisp.pl?gene=RWDD3
COX8C	Cytochrome C Oxidase Subunit 8C	Protein Coding	32	GC14P093348	4.193313122	https://www.genecards.org/cgi-bin/carddisp.pl?gene=COX8C
SDK1	Sidekick Cell Adhesion Molecule 1	Protein Coding	36	GC07P003307	4.189126968	https://www.genecards.org/cgi-bin/carddisp.pl?gene=SDK1
BMP8B	Bone Morphogenetic Protein 8b	Protein Coding	39	GC01M039757	4.175535202	https://www.genecards.org/cgi-bin/carddisp.pl?gene=BMP8B
NDUFB1	NADH:Ubiquinone Oxidoreductase Subunit B1	Protein Coding	37	GC14M092116	4.166594505	https://www.genecards.org/cgi-bin/carddisp.pl?gene=NDUFB1
TEX36	Testis Expressed 36	Protein Coding	29	GC10M125576	4.156787872	https://www.genecards.org/cgi-bin/carddisp.pl?gene=TEX36
EIF3B	Eukaryotic Translation Initiation Factor 3 Subunit B	Protein Coding	40	GC07P002354	4.150002003	https://www.genecards.org/cgi-bin/carddisp.pl?gene=EIF3B
DDX50	DExD-Box Helicase 50	Protein Coding	40	GC10P068901	4.144034386	https://www.genecards.org/cgi-bin/carddisp.pl?gene=DDX50
PSME2	Proteasome Activator Subunit 2	Protein Coding	43	GC14M024143	4.14070797	https://www.genecards.org/cgi-bin/carddisp.pl?gene=PSME2
NUDT21	Nudix Hydrolase 21	Protein Coding	38	GC16M056429	4.132987022	https://www.genecards.org/cgi-bin/carddisp.pl?gene=NUDT21
OPRD1	Opioid Receptor Delta 1	Protein Coding	45	GC01P028812	4.123717785	https://www.genecards.org/cgi-bin/carddisp.pl?gene=OPRD1
FAM171A1	Family With Sequence Similarity 171 Member A1	Protein Coding	33	GC10M015254	4.119468212	https://www.genecards.org/cgi-bin/carddisp.pl?gene=FAM171A1
MYO1C	Myosin IC	Protein Coding	44	GC17M001464	4.114840984	https://www.genecards.org/cgi-bin/carddisp.pl?gene=MYO1C
DNAJC15	DnaJ Heat Shock Protein Family (Hsp40) Member C15	Protein Coding	37	GC13P043023	4.10560894	https://www.genecards.org/cgi-bin/carddisp.pl?gene=DNAJC15
FAT1	FAT Atypical Cadherin 1	Protein Coding	40	GC04M186587	4.101447105	https://www.genecards.org/cgi-bin/carddisp.pl?gene=FAT1
PHF2	PHD Finger Protein 2	Protein Coding	39	GC09P093576	4.100211143	https://www.genecards.org/cgi-bin/carddisp.pl?gene=PHF2
MACROH2A2	MacroH2A.2 Histone	Protein Coding	30	GC10P070053	4.09620285	https://www.genecards.org/cgi-bin/carddisp.pl?gene=MACROH2A2
CHST2	Carbohydrate Sulfotransferase 2	Protein Coding	38	GC03P143119	4.075469017	https://www.genecards.org/cgi-bin/carddisp.pl?gene=CHST2
RPS8	Ribosomal Protein S8	Protein Coding	40	GC01P044775	4.07418251	https://www.genecards.org/cgi-bin/carddisp.pl?gene=RPS8
RPL23A	Ribosomal Protein L23a	Protein Coding	42	GC17P028719	4.071253777	https://www.genecards.org/cgi-bin/carddisp.pl?gene=RPL23A
ZFP90	ZFP90 Zinc Finger Protein	Protein Coding	36	GC16P068530	4.040327072	https://www.genecards.org/cgi-bin/carddisp.pl?gene=ZFP90
SEC16A	SEC16 Homolog A, Endoplasmic Reticulum Export Factor	Protein Coding	37	GC09M136440	4.038976669	https://www.genecards.org/cgi-bin/carddisp.pl?gene=SEC16A
MAPK13	Mitogen-Activated Protein Kinase 13	Protein Coding	48	GC06P055340	4.032077312	https://www.genecards.org/cgi-bin/carddisp.pl?gene=MAPK13
IL36A	Interleukin 36 Alpha	Protein Coding	37	GC02P113005	4.031521797	https://www.genecards.org/cgi-bin/carddisp.pl?gene=IL36A
TCP1	T-Complex 1	Protein Coding	41	GC06M159778	4.024827957	https://www.genecards.org/cgi-bin/carddisp.pl?gene=TCP1
RGS4	Regulator Of G Protein Signaling 4	Protein Coding	44	GC01P163038	4.008200169	https://www.genecards.org/cgi-bin/carddisp.pl?gene=RGS4
RTRAF	RNA Transcription, Translation And Transport Factor	Protein Coding	29	GC14P051992	4.000492573	https://www.genecards.org/cgi-bin/carddisp.pl?gene=RTRAF
RRBP1	Ribosome Binding Protein 1	Protein Coding	40	GC20M017613	3.978198528	https://www.genecards.org/cgi-bin/carddisp.pl?gene=RRBP1
SRRM1	Serine And Arginine Repetitive Matrix 1	Protein Coding	38	GC01P024631	3.965520382	https://www.genecards.org/cgi-bin/carddisp.pl?gene=SRRM1
CCT2	Chaperonin Containing TCP1 Subunit 2	Protein Coding	41	GC12P069585	3.961687088	https://www.genecards.org/cgi-bin/carddisp.pl?gene=CCT2
AATF	Apoptosis Antagonizing Transcription	Protein Coding	40	GC17P036948	3.959928751	https://www.genecards.org/cgi-bin/carddisp.pl?gene=AATF
PIP5K1B	Phosphatidylinositol-4-Phosphate 5-Kinase Type 1 Beta	Protein Coding	44	GC09P068705	3.938464642	https://www.genecards.org/cgi-bin/carddisp.pl?gene=PIP5K1B
USP48	Ubiquitin Specific Peptidase 48	Protein Coding	40	GC01M021678	3.935755014	https://www.genecards.org/cgi-bin/carddisp.pl?gene=USP48
RUVBL2	RuvB Like AAA ATPase 2	Protein Coding	45	GC19P048993	3.933964252	https://www.genecards.org/cgi-bin/carddisp.pl?gene=RUVBL2
PCSK7	Proprotein Convertase Subtilisin/Kexin Type 7	Protein Coding	44	GC11M117199	3.930381536	https://www.genecards.org/cgi-bin/carddisp.pl?gene=PCSK7
LOC643339	Uncharacterized LOC643339	RNA Gene	9	GC12M093005	3.923657656	https://www.genecards.org/cgi-bin/carddisp.pl?gene=LOC643339
OSBPL5	Oxysterol Binding Protein Like 5	Protein Coding	40	GC11M003088	3.89450717	https://www.genecards.org/cgi-bin/carddisp.pl?gene=OSBPL5
CCT3	Chaperonin Containing TCP1 Subunit 3	Protein Coding	42	GC01M156308	3.880765438	https://www.genecards.org/cgi-bin/carddisp.pl?gene=CCT3
C1QTNF9	C1q And TNF Related 9	Protein Coding	34	GC13P024307	3.872867584	https://www.genecards.org/cgi-bin/carddisp.pl?gene=C1QTNF9
TLCD4	TLC Domain Containing 4	Protein Coding	27	GC01P095092	3.872813702	https://www.genecards.org/cgi-bin/carddisp.pl?gene=TLCD4
SRSF4	Serine And Arginine Rich Splicing Factor	Protein Coding	39	GC01M029147	3.872788668	https://www.genecards.org/cgi-bin/carddisp.pl?gene=SRSF4
GSTK1	Glutathione S-Transferase Kappa 1	Protein Coding	42	GC07P145809	3.865218639	https://www.genecards.org/cgi-bin/carddisp.pl?gene=GSTK1
ENHO	Energy Homeostasis Associated	Protein Coding	30	GC09M034516	3.846152782	https://www.genecards.org/cgi-bin/carddisp.pl?gene=ENHO
TMC4	Transmembrane Channel Like 4	Protein Coding	35	GC19M054160	3.824649334	https://www.genecards.org/cgi-bin/carddisp.pl?gene=TMC4
TMBIM6	Transmembrane BAX Inhibitor Motif Containing 6	Protein Coding	37	GC12P049707	3.821199417	https://www.genecards.org/cgi-bin/carddisp.pl?gene=TMBIM6
RBM14	RNA Binding Motif Protein 14	Protein Coding	37	GC11P066785	3.819814205	https://www.genecards.org/cgi-bin/carddisp.pl?gene=RBM14
KIF16B	Kinesin Family Member 16B	Protein Coding	38	GC20M016272	3.819159985	https://www.genecards.org/cgi-bin/carddisp.pl?gene=KIF16B
SVIL-AS1	SVIL Antisense RNA 1	RNA Gene	13	GC10P029410	3.811452866	https://www.genecards.org/cgi-bin/carddisp.pl?gene=SVIL-AS1
RAB37	RAB37, Member RAS Oncogene Family	Protein Coding	39	GC17P074671	3.804848433	https://www.genecards.org/cgi-bin/carddisp.pl?gene=RAB37
NDUFA4L2	NDUFA4 Mitochondrial Complex Associated Like 2	Protein Coding	37	GC12M057234	3.802900076	https://www.genecards.org/cgi-bin/carddisp.pl?gene=NDUFA4L2
DDX17	DEAD-Box Helicase 17	Protein Coding	41	GC22M038483	3.797605515	https://www.genecards.org/cgi-bin/carddisp.pl?gene=DDX17
RND3	Rho Family GTPase 3	Protein Coding	43	GC02M150468	3.790923595	https://www.genecards.org/cgi-bin/carddisp.pl?gene=RND3
TMOD3	Tropomodulin 3	Protein Coding	40	GC15P051829	3.783874989	https://www.genecards.org/cgi-bin/carddisp.pl?gene=TMOD3

CARD6	Caspase Recruitment Domain Family Member 6	Protein Coding	38	GC05P040841	3.781528711	https://www.genecards.org/cgi-bin/carddisp.pl?gene=CARD6
GSDMD	Gasdermin D	Protein Coding	40	GC08P143553	3.768371582	https://www.genecards.org/cgi-bin/carddisp.pl?gene=GSDMD
XPO4	Exportin 4	Protein Coding	38	GC13M020777	3.76751852	https://www.genecards.org/cgi-bin/carddisp.pl?gene=XPO4
UBE2D3	Ubiquitin Conjugating Enzyme E2 D3	Protein Coding	47	GC04M102794	3.764195442	https://www.genecards.org/cgi-bin/carddisp.pl?gene=UBE2D3
SF3B3	Splicing Factor 3b Subunit 3	Protein Coding	40	GC16P070523	3.760380745	https://www.genecards.org/cgi-bin/carddisp.pl?gene=SF3B3
PDE7B	Phosphodiesterase 7B	Protein Coding	39	GC06P135795	3.755542755	https://www.genecards.org/cgi-bin/carddisp.pl?gene=PDE7B
RNU6-53P	RNA, U6 Small Nuclear 53, Pseudogene	Pseudogene	8	GC13M028703	3.752223015	https://www.genecards.org/cgi-bin/carddisp.pl?gene=RNU6-53P
FOXN3	Forkhead Box N3	Protein Coding	39	GC14M095787	3.749269485	https://www.genecards.org/cgi-bin/carddisp.pl?gene=FOXN3
CIDEA	Cell Death Inducing DFFA Like Effector A	Protein Coding	41	GC18P012254	3.748991013	https://www.genecards.org/cgi-bin/carddisp.pl?gene=CIDEA
IBTK	Inhibitor Of Bruton Tyrosine Kinase	Protein Coding	40	GC06M082169	3.722085476	https://www.genecards.org/cgi-bin/carddisp.pl?gene=IBTK
EIF3L	Eukaryotic Translation Initiation Factor 3 Subunit L	Protein Coding	39	GC22P037848	3.714426041	https://www.genecards.org/cgi-bin/carddisp.pl?gene=EIF3L
TRMT44	TRNA Methyltransferase 44 Homolog	Protein Coding	32	GC04P008439	3.696261883	https://www.genecards.org/cgi-bin/carddisp.pl?gene=TRMT44
SLC9A4	Solute Carrier Family 9 Member A4	Protein Coding	36	GC02P102456	3.694443941	https://www.genecards.org/cgi-bin/carddisp.pl?gene=SLC9A4
RLN2	Relaxin 2	Protein Coding	37	GC09M005306	3.670796394	https://www.genecards.org/cgi-bin/carddisp.pl?gene=RLN2
NLRP2	NLR Family Pyrin Domain Containing 2	Protein Coding	42	GC19P054953	3.669286251	https://www.genecards.org/cgi-bin/carddisp.pl?gene=NLRP2
PLPP7	Phospholipid Phosphatase 7 (Inactive)	Protein Coding	28	GC09P131290	3.65639019	https://www.genecards.org/cgi-bin/carddisp.pl?gene=PLPP7
WSPAR	WNT Signaling Pathway Activating Non-Coding RNA	RNA Gene	11	GC05P133914	3.637709856	https://www.genecards.org/cgi-bin/carddisp.pl?gene=WSPAR
USP4	Ubiquitin Specific Peptidase 4	Protein Coding	43	GC03M049277	3.622792959	https://www.genecards.org/cgi-bin/carddisp.pl?gene=USP4
C1QTNF3	C1q And TNF Related 3	Protein Coding	38	GC05M034017	3.622492552	https://www.genecards.org/cgi-bin/carddisp.pl?gene=C1QTNF3
UBXN1	UBX Domain Protein 1	Protein Coding	36	GC11M069259	3.616335869	https://www.genecards.org/cgi-bin/carddisp.pl?gene=UBXN1
LINC00917	Long Intergenic Non-Protein Coding RNA 917	RNA Gene	15	GC16M086366	3.609225273	https://www.genecards.org/cgi-bin/carddisp.pl?gene=LINC00917
ADAMTS9-AS2	ADAMTS9 Antisense RNA 2	RNA Gene	16	GC03P064671	3.607175827	https://www.genecards.org/cgi-bin/carddisp.pl?gene=ADAMTS9-AS2
CIDEB	Cell Death Inducing DFFA Like Effector B	Protein Coding	37	GC14M024305	3.60635519	https://www.genecards.org/cgi-bin/carddisp.pl?gene=CIDEB
EIF3E	Eukaryotic Translation Initiation Factor 3 Subunit E	Protein Coding	39	GC08M108163	3.590932846	https://www.genecards.org/cgi-bin/carddisp.pl?gene=EIF3E
ARPC2	Actin Related Protein 2/3 Complex	Protein Coding	43	GC02P218217	3.587478876	https://www.genecards.org/cgi-bin/carddisp.pl?gene=ARPC2
KRTAP5-5	Keratin Associated Protein 5-5	Protein Coding	26	GC11P001629	3.583631992	https://www.genecards.org/cgi-bin/carddisp.pl?gene=KRTAP5-5
KLHL6	Kelch Like Family Member 6	Protein Coding	36	GC03M183487	3.572049141	https://www.genecards.org/cgi-bin/carddisp.pl?gene=KLHL6
HSD17B11	Hydroxysteroid 17-Beta Dehydrogenase 11	Protein Coding	41	GC04M087336	3.558064461	https://www.genecards.org/cgi-bin/carddisp.pl?gene=HSD17B11
SLC26A6	Solute Carrier Family 26 Member 6	Protein Coding	39	GC03M048625	3.55651474	https://www.genecards.org/cgi-bin/carddisp.pl?gene=SLC26A6
COX7B2	Cytochrome C Oxidase Subunit 7B2	Protein Coding	32	GC04M046734	3.554057598	https://www.genecards.org/cgi-bin/carddisp.pl?gene=COX7B2
VSIG4	V-Set And Immunoglobulin Domain Containing 4	Protein Coding	37	GC0XM066021	3.552012444	https://www.genecards.org/cgi-bin/carddisp.pl?gene=VSIG4
SNHG18	Small Nucleolar RNA Host Gene 18	RNA Gene	15	GC05P009562	3.546533346	https://www.genecards.org/cgi-bin/carddisp.pl?gene=SNHG18
ILF2	Interleukin Enhancer Binding Factor 2	Protein Coding	40	GC01M153661	3.53593874	https://www.genecards.org/cgi-bin/carddisp.pl?gene=ILF2
HIGD1A	HIG1 Hypoxia Inducible Domain Family Member 1A	Protein Coding	38	GC03M042782	3.535363674	https://www.genecards.org/cgi-bin/carddisp.pl?gene=HIGD1A
MYBBP1A	MYB Binding Protein 1a	Protein Coding	40	GC17M004538	3.525005579	https://www.genecards.org/cgi-bin/carddisp.pl?gene=MYBBP1A
PWWP3A	PWWP Domain Containing 3A, DNA Repair Factor	Protein Coding	21	GC19P001625	3.518794775	https://www.genecards.org/cgi-bin/carddisp.pl?gene=PWWP3A
GGT3P	Gamma-Glutamyltransferase 3 Pseudogene	Pseudogene	18	GC22M018773	3.518324614	https://www.genecards.org/cgi-bin/carddisp.pl?gene=GGT3P
ANKK1	Ankyrin Repeat And Kinase Domain Containing 1	Protein Coding	38	GC11P113387	3.5160923	https://www.genecards.org/cgi-bin/carddisp.pl?gene=ANKK1
MAU2	MAU2 Sister Chromatid Cohesion Factor	Protein Coding	33	GC19P019320	3.510564804	https://www.genecards.org/cgi-bin/carddisp.pl?gene=MAU2
LOC101929710	Uncharacterized LOC101929710	RNA Gene	11	GC05P095962	3.50092864	https://www.genecards.org/cgi-bin/carddisp.pl?gene=LOC101929710
ZBTB7A	Zinc Finger And BTB Domain Containing	Protein Coding	37	GC19M004045	3.495354891	https://www.genecards.org/cgi-bin/carddisp.pl?gene=ZBTB7A
MIR1183	MicroRNA 1183	RNA Gene	15	GC07P021510	3.488068104	https://www.genecards.org/cgi-bin/carddisp.pl?gene=MIR1183
CDH26	Cadherin 26	Protein Coding	36	GC20P059958	3.485193253	https://www.genecards.org/cgi-bin/carddisp.pl?gene=CDH26
AGR3	Anterior Gradient 3, Protein Disulphide Isomerase Family Member	Protein Coding	38	GC07M016854	3.473514557	https://www.genecards.org/cgi-bin/carddisp.pl?gene=AGR3
COL21A1	Collagen Type XXI Alpha 1 Chain	Protein Coding	37	GC06M056057	3.468084812	https://www.genecards.org/cgi-bin/carddisp.pl?gene=COL21A1
SMOC2	SPARC Related Modular Calcium Binding	Protein Coding	42	GC06P168441	3.46173811	https://www.genecards.org/cgi-bin/carddisp.pl?gene=SMOC2
PABPC4	Poly(A) Binding Protein Cytoplasmic 4	Protein Coding	42	GC01M039560	3.453729153	https://www.genecards.org/cgi-bin/carddisp.pl?gene=PABPC4
LRRFIP2	LRR Binding FLII Interacting Protein 2	Protein Coding	38	GC03M037052	3.445348263	https://www.genecards.org/cgi-bin/carddisp.pl?gene=LRRFIP2
CNOT4	CCR4-NOT Transcription Complex Subunit 4	Protein Coding	41	GC07M135361	3.438128233	https://www.genecards.org/cgi-bin/carddisp.pl?gene=CNOT4
CKAP4	Cytoskeleton Associated Protein 4	Protein Coding	38	GC12M106237	3.431976795	https://www.genecards.org/cgi-bin/carddisp.pl?gene=CKAP4
SUB1	SUB1 Regulator Of Transcription	Protein Coding	39	GC05P032540	3.412381172	https://www.genecards.org/cgi-bin/carddisp.pl?gene=SUB1
BECN2	Beclin 2	Protein Coding	23	GC01P241957	3.368844032	https://www.genecards.org/cgi-bin/carddisp.pl?gene=BECN2
COX6B2	Cytochrome C Oxidase Subunit 6B2	Protein Coding	32	GC19M055350	3.349303246	https://www.genecards.org/cgi-bin/carddisp.pl?gene=COX6B2
USP10	Ubiquitin Specific Peptidase 10	Protein Coding	44	GC16P084734	3.347266674	https://www.genecards.org/cgi-bin/carddisp.pl?gene=USP10
LATS2	Large Tumor Suppressor Kinase 2	Protein Coding	46	GC13M020973	3.345319748	https://www.genecards.org/cgi-bin/carddisp.pl?gene=LATS2
HSALNG0013825		RNA Gene	1	GC02P027507	3.336766005	https://www.genecards.org/cgi-bin/carddisp.pl?gene=HSALNG0013825
ACTR5	Actin Related Protein 5	Protein Coding	35	GC20P038748	3.329617023	https://www.genecards.org/cgi-bin/carddisp.pl?gene=ACTR5
CCDC63	Coiled-Coil Domain Containing 63	Protein Coding	34	GC12P110846	3.328995228	https://www.genecards.org/cgi-bin/carddisp.pl?gene=CCDC63
FAM3B	FAM3 Metabolism Regulating Signaling Molecule B	Protein Coding	39	GC21P041304	3.328571796	https://www.genecards.org/cgi-bin/carddisp.pl?gene=FAM3B
DDX60	DExD/H-Box Helicase 60	Protein Coding	35	GC04M168216	3.312973022	https://www.genecards.org/cgi-bin/carddisp.pl?gene=DDX60
TSNAX-DISC1	TSNAX-DISC1 Readthrough (NMD Candidate)	RNA Gene	19	GC01P231528	3.306806564	https://www.genecards.org/cgi-bin/carddisp.pl?gene=TSNAX-DISC1
SRRM2	Serine/Arginine Repetitive Matrix 2	Protein Coding	34	GC16P005607	3.298483372	https://www.genecards.org/cgi-bin/carddisp.pl?gene=SRRM2
GGT5	Gamma-Glutamyltransferase 5	Protein Coding	40	GC22M024219	3.290547848	https://www.genecards.org/cgi-bin/carddisp.pl?gene=GGT5
LINC01405	Long Intergenic Non-Protein Coding RNA 1405	RNA Gene	15	GC12P110936	3.284021854	https://www.genecards.org/cgi-bin/carddisp.pl?gene=LINC01405
LINC01016	Long Intergenic Non-Protein Coding RNA 1016	RNA Gene	13	GC06M047659	3.284021854	https://www.genecards.org/cgi-bin/carddisp.pl?gene=LINC01016
SH3BP5	SH3 Domain Binding Protein 5	Protein Coding	41	GC03M016936	3.279747486	https://www.genecards.org/cgi-bin/carddisp.pl?gene=SH3BP5
MYL6B	Myosin Light Chain 6B	Protein Coding	40	GC12P056388	3.252141237	https://www.genecards.org/cgi-bin/carddisp.pl?gene=MYL6B
ZNF101	Zinc Finger Protein 101	Protein Coding	34	GC19P026665	3.250607252	https://www.genecards.org/cgi-bin/carddisp.pl?gene=ZNF101
RESF1	Retroelement Silencing Factor 1	Protein Coding	25	GC12P032003	3.248668194	https://www.genecards.org/cgi-bin/carddisp.pl?gene=RESF1
STRA8	Stimulated By Retinoic Acid 8	Protein Coding	36	GC07P135231	3.248404503	https://www.genecards.org/cgi-bin/carddisp.pl?gene=STRA8
FAM3A	FAM3 Metabolism Regulating Signaling Molecule A	Protein Coding	37	GC0XM154506	3.223508358	https://www.genecards.org/cgi-bin/carddisp.pl?gene=FAM3A
MLXIP	MLX Interacting Protein	Protein Coding	33	GC12P122078	3.219124794	https://www.genecards.org/cgi-bin/carddisp.pl?gene=MLXIP
DEFA1	Defensin Alpha 1	Protein Coding	41	GC08M006977	3.217813015	https://www.genecards.org/cgi-bin/carddisp.pl?gene=DEFA1
DDX46	DEAD-Box Helicase 46	Protein Coding	40	GC05P134758	3.211338997	https://www.genecards.org/cgi-bin/carddisp.pl?gene=DDX46
KDM4B	Lysine Demethylase 4B	Protein Coding	44	GC19P004969	3.19203496	https://www.genecards.org/cgi-bin/carddisp.pl?gene=KDM4B
UQCRHL	Ubiquinol-Cytochrome C Reductase Hinge Protein Like	Protein Coding	23	GC01M015808	3.182341337	https://www.genecards.org/cgi-bin/carddisp.pl?gene=UQCRHL
MIR646HG	MIR646 Host Gene	RNA Gene	15	GC20P060088	3.172647953	https://www.genecards.org/cgi-bin/carddisp.pl?gene=MIR646HG
EIF4A2	Eukaryotic Translation Initiation Factor	Protein Coding	45	GC03P186783	3.160809994	https://www.genecards.org/cgi-bin/carddisp.pl?gene=EIF4A2
ARL2BPP6	ADP Ribosylation Factor Like GTPase 2 Binding Protein Pseudogene 6	Pseudogene	6	GC05P175290	3.157893658	https://www.genecards.org/cgi-bin/carddisp.pl?gene=ARL2BPP6
ZNF267	Zinc Finger Protein 267	Protein Coding	36	GC16P032629	3.153172493	https://www.genecards.org/cgi-bin/carddisp.pl?gene=ZNF267
ENSG00000231487		RNA Gene	6	GC11M001683	3.115470886	https://www.genecards.org/cgi-bin/carddisp.pl?gene=ENSG00000231487
C16orf95	Chromosome 16 Open Reading Frame 95	Protein Coding	26	GC16M087119	3.112183571	https://www.genecards.org/cgi-bin/carddisp.pl?gene=C16orf95
lnc-TM6SF2-1		RNA Gene	4	GC19M019270	3.110280991	https://www.genecards.org/cgi-bin/carddisp.pl?gene=lnc-TM6SF2-1
ENSG00000267629		RNA Gene	3	GC19M019254	3.110280991	https://www.genecards.org/cgi-bin/carddisp.pl?gene=ENSG00000267629

MIR6090	MicroRNA 6090	RNA Gene	10	GC11P128522	3.100980997	https://www.genecards.org/cgi-bin/carddisp.pl?gene=MIR6090
IL36B	Interleukin 36 Beta	Protein Coding	35	GC02M113022	3.09431529	https://www.genecards.org/cgi-bin/carddisp.pl?gene=IL36B
ETS1-AS1	ETS1 Antisense RNA 1	RNA Gene	11	GC11P128527	3.086783648	https://www.genecards.org/cgi-bin/carddisp.pl?gene=ETS1-AS1
ARHGAP8	Rho GTPase Activating Protein 8	Protein Coding	34	GC22P044752	3.085615635	https://www.genecards.org/cgi-bin/carddisp.pl?gene=ARHGAP8
RSPO3	R-Spondin 3	Protein Coding	38	GC06P127118	3.079731464	https://www.genecards.org/cgi-bin/carddisp.pl?gene=RSPO3
A2MP1	Alpha-2-Macroglobulin Pseudogene 1	Pseudogene	13	GC12M009228	3.064694881	https://www.genecards.org/cgi-bin/carddisp.pl?gene=A2MP1
GLT8D2	Glycosyltransferase 8 Domain Containing	Protein Coding	36	GC12M103988	3.059843779	https://www.genecards.org/cgi-bin/carddisp.pl?gene=GLT8D2
RAB32	RAB32, Member RAS Oncogene Family	Protein Coding	39	GC06P146543	3.033777237	https://www.genecards.org/cgi-bin/carddisp.pl?gene=RAB32
HIF3A	Hypoxia Inducible Factor 3 Subunit Alpha	Protein Coding	40	GC19P046297	3.029106379	https://www.genecards.org/cgi-bin/carddisp.pl?gene=HIF3A
TMEM97	Transmembrane Protein 97	Protein Coding	36	GC17P028319	3.014130592	https://www.genecards.org/cgi-bin/carddisp.pl?gene=TMEM97
TRPC4AP	Transient Receptor Potential Cation Channel Subfamily C Member 4	Protein Coding	37	GC20M035002	3.013672829	https://www.genecards.org/cgi-bin/carddisp.pl?gene=TRPC4AP
LINC02694	Long Intergenic Non-Protein Coding RNA 2694	RNA Gene	23	GC15P038622	3.004884243	https://www.genecards.org/cgi-bin/carddisp.pl?gene=LINC02694
SMIM20	Small Integral Membrane Protein 20	Protein Coding	29	GC04P025865	3.002661467	https://www.genecards.org/cgi-bin/carddisp.pl?gene=SMIM20
RNU6-961P	RNA, U6 Small Nuclear 961, Pseudogene	Pseudogene	8	GC02P020175	2.9941535	https://www.genecards.org/cgi-bin/carddisp.pl?gene=RNU6-961P
DHX15	DEAH-Box Helicase 15	Protein Coding	38	GC04M024519	2.965582848	https://www.genecards.org/cgi-bin/carddisp.pl?gene=DHX15
LINC01354	Long Intergenic Non-Protein Coding RNA 1354	RNA Gene	12	GC01M234528	2.957972765	https://www.genecards.org/cgi-bin/carddisp.pl?gene=LINC01354
RUBCNL	Rubicon Like Autophagy Enhancer	Protein Coding	26	GC13M046343	2.950154305	https://www.genecards.org/cgi-bin/carddisp.pl?gene=RUBCNL
GBX2	Gastrulation Brain Homeobox 2	Protein Coding	40	GC02M236165	2.949873447	https://www.genecards.org/cgi-bin/carddisp.pl?gene=GBX2
ENSG00000 253111		RNA Gene	7	GC08P125466	2.923579693	https://www.genecards.org/cgi-bin/carddisp.pl?gene=ENSG00000253111
SDF2L1	Stromal Cell Derived Factor 2 Like 1	Protein Coding	35	GC22P026487	2.91104269	https://www.genecards.org/cgi-bin/carddisp.pl?gene=SDF2L1
EBNA1BP2	EBNA1 Binding Protein 2	Protein Coding	38	GC01M043165	2.898739815	https://www.genecards.org/cgi-bin/carddisp.pl?gene=EBNA1BP2
HSALNG00 13412		RNA Gene	4	GC02P020202	2.88000536	https://www.genecards.org/cgi-bin/carddisp.pl?gene=HSALNG0013412
lnc-COA6-		RNA Gene	4	GC01P234522	2.88000536	https://www.genecards.org/cgi-bin/carddisp.pl?gene=lnc-COA6-13
RF00017-		RNA Gene	3	GC01M234512	2.88000536	https://www.genecards.org/cgi-bin/carddisp.pl?gene=RF00017-457
LINC01679	Long Intergenic Non-Protein Coding RNA 1679	RNA Gene	9	GC21M043360	2.876531601	https://www.genecards.org/cgi-bin/carddisp.pl?gene=LINC01679
RTCB	RNA 2',3'-Cyclic Phosphate And 5'-OH Ligase	Protein Coding	36	GC22M032387	2.847796679	https://www.genecards.org/cgi-bin/carddisp.pl?gene=RTCB
DUSP26	Dual Specificity Phosphatase 26	Protein Coding	37	GC08M033591	2.838163137	https://www.genecards.org/cgi-bin/carddisp.pl?gene=DUSP26
CAPSL	Calcyphosine Like	Protein Coding	34	GC05M035906	2.813187122	https://www.genecards.org/cgi-bin/carddisp.pl?gene=CAPSL
ENSG00000 231992		RNA Gene	8	GC01P095120	2.807580948	https://www.genecards.org/cgi-bin/carddisp.pl?gene=ENSG00000231992
SLC2A8	Solute Carrier Family 2 Member 8	Protein Coding	40	GC09P127398	2.771994591	https://www.genecards.org/cgi-bin/carddisp.pl?gene=SLC2A8
FECHP1	Ferrochelatase Pseudogene 1	Pseudogene	6	GC03P034873	2.76852107	https://www.genecards.org/cgi-bin/carddisp.pl?gene=FECHP1
LINC00322	Long Intergenic Non-Protein Coding RNA 322	RNA Gene	17	GC21M043322	2.767420769	https://www.genecards.org/cgi-bin/carddisp.pl?gene=LINC00322
CENPM	Centromere Protein M	Protein Coding	36	GC22M048546	2.765411615	https://www.genecards.org/cgi-bin/carddisp.pl?gene=CENPM
LOC644135	Uncharacterized LOC644135	RNA Gene	11	GC06M135856	2.751729727	https://www.genecards.org/cgi-bin/carddisp.pl?gene=LOC644135
LOC101928 135	Uncharacterized LOC101928135	RNA Gene	6	GC03M034875	2.745675802	https://www.genecards.org/cgi-bin/carddisp.pl?gene=LOC101928135
ENSG00000 287092		RNA Gene	5	GC06M155810	2.735893488	https://www.genecards.org/cgi-bin/carddisp.pl?gene=ENSG00000287092
COP1	COP1 E3 Ubiquitin Ligase	Protein Coding	34	GC01M175944	2.710287809	https://www.genecards.org/cgi-bin/carddisp.pl?gene=COP1
LOC389199	Uncharacterized LOC389199	Protein Coding	17	GC04P007939	2.694767237	https://www.genecards.org/cgi-bin/carddisp.pl?gene=LOC389199
INPP4B	Inositol Polyphosphate-4-Phosphatase Type II B	Protein Coding	39	GC04M142023	2.680804968	https://www.genecards.org/cgi-bin/carddisp.pl?gene=INPP4B
ENSG00000 228852		RNA Gene	7	GC01M095243	2.677582741	https://www.genecards.org/cgi-bin/carddisp.pl?gene=ENSG00000228852
ENSG00000 287292		RNA Gene	6	GC04P148445	2.65933919	https://www.genecards.org/cgi-bin/carddisp.pl?gene=ENSG00000287292
ENSG00000 254599		RNA Gene	5	GC11P098676	2.65933919	https://www.genecards.org/cgi-bin/carddisp.pl?gene=ENSG00000254599
HSALNG01 33406		RNA Gene	3	GC21P043345	2.65933919	https://www.genecards.org/cgi-bin/carddisp.pl?gene=HSALNG0133406
PRR5-ARHGAP8	PRR5-ARHGAP8 Readthrough	Protein Coding	27	GC22P044702	2.655734301	https://www.genecards.org/cgi-bin/carddisp.pl?gene=PRR5-ARHGAP8
LINC01684	Long Intergenic Non-Protein Coding RNA 1684	RNA Gene	9	GC21P024306	2.652424335	https://www.genecards.org/cgi-bin/carddisp.pl?gene=LINC01684
FAIM	Fas Apoptotic Inhibitory Molecule	Protein Coding	40	GC03P138608	2.64071846	https://www.genecards.org/cgi-bin/carddisp.pl?gene=FAIM
ATP6AP1L	ATPase H+ Transporting Accessory Protein 1 Like	Protein Coding	26	GC05P082279	2.607598066	https://www.genecards.org/cgi-bin/carddisp.pl?gene=ATP6AP1L
LYPLAL1	Lysophospholipase Like 1	Protein Coding	37	GC01P219173	2.601939678	https://www.genecards.org/cgi-bin/carddisp.pl?gene=LYPLAL1
piR-37045		RNA Gene	2	GC22P044002	2.59913063	https://www.genecards.org/cgi-bin/carddisp.pl?gene=piR-37045
HSALNG01 35823		RNA Gene	2	GC22P043995	2.59913063	https://www.genecards.org/cgi-bin/carddisp.pl?gene=HSALNG0135823
RAMP3	Receptor Activity Modifying Protein 3	Protein Coding	41	GC07P045163	2.592380047	https://www.genecards.org/cgi-bin/carddisp.pl?gene=RAMP3
REPIN1	Replication Initiator 1	Protein Coding	34	GC07P150368	2.590229988	https://www.genecards.org/cgi-bin/carddisp.pl?gene=REPIN1
H2BC11	H2B Clustered Histone 11	Protein Coding	29	GC06M046548	2.588163853	https://www.genecards.org/cgi-bin/carddisp.pl?gene=H2BC11
TPI1P1	Triosephosphate Isomerase 1 Pseudogene	Pseudogene	7	GC01P076699	2.586718082	https://www.genecards.org/cgi-bin/carddisp.pl?gene=TPI1P1
TEX36-AS1	TEX36 Antisense RNA 1	RNA Gene	11	GC10P125574	2.571750164	https://www.genecards.org/cgi-bin/carddisp.pl?gene=TEX36-AS1
ENSG00000 255046		RNA Gene	8	GC08M011991	2.571750164	https://www.genecards.org/cgi-bin/carddisp.pl?gene=ENSG00000255046
ENSG00000 222042		RNA Gene	6	GC21M025169	2.571750164	https://www.genecards.org/cgi-bin/carddisp.pl?gene=ENSG00000222042
OTX2P1	OTX2 Pseudogene 1	Pseudogene	5	GC09M075724	2.571750164	https://www.genecards.org/cgi-bin/carddisp.pl?gene=OTX2P1
lnc-JCAD-2		RNA Gene	5	GC10M031184	2.571750164	https://www.genecards.org/cgi-bin/carddisp.pl?gene=lnc-JCAD-2
ENSG00000 237936		Pseudogene	4	GC10M029891	2.571750164	https://www.genecards.org/cgi-bin/carddisp.pl?gene=ENSG00000237936
THNSL2	Threonine Synthase Like 2	Protein Coding	33	GC02P088170	2.56402421	https://www.genecards.org/cgi-bin/carddisp.pl?gene=THNSL2
HCAR2	Hydroxycarboxylic Acid Receptor 2	Protein Coding	38	GC12M122701	2.548469543	https://www.genecards.org/cgi-bin/carddisp.pl?gene=HCAR2
STARP1	Steroidogenic Acute Regulatory Protein Pseudogene 1	Pseudogene	7	GC13M065280	2.520123243	https://www.genecards.org/cgi-bin/carddisp.pl?gene=STARP1
ENSG00000 234389		RNA Gene	8	GC02P102438	2.508545876	https://www.genecards.org/cgi-bin/carddisp.pl?gene=ENSG00000234389
ENSG00000 249631		RNA Gene	7	GC04P014604	2.508545876	https://www.genecards.org/cgi-bin/carddisp.pl?gene=ENSG00000249631
HSALNG00 88100		RNA Gene	4	GC11M128462	2.508545876	https://www.genecards.org/cgi-bin/carddisp.pl?gene=HSALNG0088100
HSALNG00 88101		RNA Gene	4	GC11P128480	2.508545876	https://www.genecards.org/cgi-bin/carddisp.pl?gene=HSALNG0088101
HSALNG00 88102		RNA Gene	4	GC11M128489	2.508545876	https://www.genecards.org/cgi-bin/carddisp.pl?gene=HSALNG0088102
FNDC3B	Fibronectin Type III Domain Containing	Protein Coding	37	GC03P172039	2.500176907	https://www.genecards.org/cgi-bin/carddisp.pl?gene=FNDC3B
LINC00355	Long Intergenic Non-Protein Coding RNA 355	RNA Gene	15	GC13M063838	2.494673729	https://www.genecards.org/cgi-bin/carddisp.pl?gene=LINC00355
LOC105373 170	Uncharacterized LOC105373170	RNA Gene	6	GC01M231768	2.494673729	https://www.genecards.org/cgi-bin/carddisp.pl?gene=LOC105373170
KRT8P18	Keratin 8 Pseudogene 18	Pseudogene	5	GC03M035233	2.470010996	https://www.genecards.org/cgi-bin/carddisp.pl?gene=KRT8P18
CTDNEP1	CTD Nuclear Envelope Phosphatase 1	Protein Coding	36	GC17M007243	2.459027767	https://www.genecards.org/cgi-bin/carddisp.pl?gene=CTDNEP1
SLC9A9-	SLC9A9 Antisense RNA 1	RNA Gene	13	GC03P143342	2.457602024	https://www.genecards.org/cgi-bin/carddisp.pl?gene=SLC9A9-AS1
LOC100130 331	POTE Ankyrin Domain Family, Member F Pseudogene	Pseudogene	10	GC01P237862	2.457602024	https://www.genecards.org/cgi-bin/carddisp.pl?gene=LOC100130331

ENSG00000257322		RNA Gene	8	GC12M093003	2.457602024	https://www.genecards.org/cgi-bin/carddisp.pl?gene=ENSG00000257322
ENSG00000236501		RNA Gene	8	GC02M176959	2.457602024	https://www.genecards.org/cgi-bin/carddisp.pl?gene=ENSG00000236501
RNU6-908P	RNA, U6 Small Nuclear 908, Pseudogene	Pseudogene	7	GC10P029711	2.457602024	https://www.genecards.org/cgi-bin/carddisp.pl?gene=RNU6-908P
ENSG00000250541		RNA Gene	6	GC04P025771	2.457602024	https://www.genecards.org/cgi-bin/carddisp.pl?gene=ENSG00000250541
ENSG00000265994		RNA Gene	6	GC18P028638	2.457602024	https://www.genecards.org/cgi-bin/carddisp.pl?gene=ENSG00000265994
ENSG00000207171		RNA Gene	6	GC04P168471	2.457602024	https://www.genecards.org/cgi-bin/carddisp.pl?gene=ENSG00000207171
EEF1A1P20	Eukaryotic Translation Elongation Factor 1 Alpha 1 Pseudogene 20	Pseudogene	5	GC05P099996	2.457602024	https://www.genecards.org/cgi-bin/carddisp.pl?gene=EEF1A1P20
RNU1-35P	RNA, U1 Small Nuclear 35, Pseudogene	Pseudogene	5	GC08M135742	2.457602024	https://www.genecards.org/cgi-bin/carddisp.pl?gene=RNU1-35P
ENSG00000235464		RNA Gene	5	GC07M035496	2.457602024	https://www.genecards.org/cgi-bin/carddisp.pl?gene=ENSG00000235464
ENSG00000236389		RNA Gene	5	GC06M134706	2.457602024	https://www.genecards.org/cgi-bin/carddisp.pl?gene=ENSG00000236389
RNU6-161P	RNA, U6 Small Nuclear 161, Pseudogene	Pseudogene	5	GC01P076753	2.457602024	https://www.genecards.org/cgi-bin/carddisp.pl?gene=RNU6-161P
MTND1P25	MT-ND1 Pseudogene 25	Pseudogene	4	GC01M237942	2.457602024	https://www.genecards.org/cgi-bin/carddisp.pl?gene=MTND1P25
ENSG00000284676		RNA Gene	4	GC01P029755	2.457602024	https://www.genecards.org/cgi-bin/carddisp.pl?gene=ENSG00000284676
ENSG00000287032		RNA Gene	4	GC07M035614	2.457602024	https://www.genecards.org/cgi-bin/carddisp.pl?gene=ENSG00000287032
ENSG00000276627		RNA Gene	4	GC20P060716	2.457602024	https://www.genecards.org/cgi-bin/carddisp.pl?gene=ENSG00000276627
HSALNG0076986		RNA Gene	4	GC10M031161	2.457602024	https://www.genecards.org/cgi-bin/carddisp.pl?gene=HSALNG0076986
lnc-CRYAA-1		RNA Gene	4	GC21P043350	2.457602024	https://www.genecards.org/cgi-bin/carddisp.pl?gene=lnc-CRYAA-1
piR-55809-001		RNA Gene	4	GC01M095168	2.457602024	https://www.genecards.org/cgi-bin/carddisp.pl?gene=piR-55809-001
lnc-DDX60L-3		RNA Gene	4	GC04M168468	2.457602024	https://www.genecards.org/cgi-bin/carddisp.pl?gene=lnc-DDX60L-3
LOC102723733	Uncharacterized LOC102723733	RNA Gene	3	GC04P025862	2.457602024	https://www.genecards.org/cgi-bin/carddisp.pl?gene=LOC102723733
ENSG00000270927		Pseudogene	3	GC01M029904	2.457602024	https://www.genecards.org/cgi-bin/carddisp.pl?gene=ENSG00000270927
ENSG00000226973		Pseudogene	3	GC01P115471	2.457602024	https://www.genecards.org/cgi-bin/carddisp.pl?gene=ENSG00000226973
LOC107986198	Uncharacterized LOC107986198	RNA Gene	3	GC04M168488	2.457602024	https://www.genecards.org/cgi-bin/carddisp.pl?gene=LOC107986198
lnc-EDRF1-4		RNA Gene	3	GC10P125590	2.457602024	https://www.genecards.org/cgi-bin/carddisp.pl?gene=lnc-EDRF1-4
HSALNG0038430		RNA Gene	3	GC04P168487	2.457602024	https://www.genecards.org/cgi-bin/carddisp.pl?gene=HSALNG0038430
HSALNG0029504		RNA Gene	3	GC03P143161	2.457602024	https://www.genecards.org/cgi-bin/carddisp.pl?gene=HSALNG0029504
HSALNG0076987-001		RNA Gene	3	GC10P029811	2.457602024	https://www.genecards.org/cgi-bin/carddisp.pl?gene=HSALNG0076987-001
HSALNG0076987-002		RNA Gene	3	GC10P029812	2.457602024	https://www.genecards.org/cgi-bin/carddisp.pl?gene=HSALNG0076987-002
lnc-AIFM2-3		RNA Gene	3	GC10M069839	2.457602024	https://www.genecards.org/cgi-bin/carddisp.pl?gene=lnc-AIFM2-3
HSALNG0093093		RNA Gene	3	GC12M093112	2.457602024	https://www.genecards.org/cgi-bin/carddisp.pl?gene=HSALNG0093093
piR-38051-198		RNA Gene	3	GC04M025877	2.457602024	https://www.genecards.org/cgi-bin/carddisp.pl?gene=piR-38051-198
piR-41195-080		RNA Gene	3	GC08M012002	2.457602024	https://www.genecards.org/cgi-bin/carddisp.pl?gene=piR-41195-080
piR-59297-626		RNA Gene	3	GC08P011819	2.457602024	https://www.genecards.org/cgi-bin/carddisp.pl?gene=piR-59297-626
AB372775		RNA Gene	2	GC10P069827	2.457602024	https://www.genecards.org/cgi-bin/carddisp.pl?gene=AB372775
HSALNG0029512		RNA Gene	2	GC03P143373	2.457602024	https://www.genecards.org/cgi-bin/carddisp.pl?gene=HSALNG0029512
HSALNG0093097		RNA Gene	2	GC12M093154	2.457602024	https://www.genecards.org/cgi-bin/carddisp.pl?gene=HSALNG0093097
piR-31109-001		RNA Gene	2	GC10P125633	2.457602024	https://www.genecards.org/cgi-bin/carddisp.pl?gene=piR-31109-001
piR-57133-376		RNA Gene	2	GC20P060683	2.457602024	https://www.genecards.org/cgi-bin/carddisp.pl?gene=piR-57133-376
HSALNG0131408		RNA Gene	2	GC20M060680	2.457602024	https://www.genecards.org/cgi-bin/carddisp.pl?gene=HSALNG0131408
ENSG00000254939		Pseudogene	1	GC11M098568	2.457602024	https://www.genecards.org/cgi-bin/carddisp.pl?gene=ENSG00000254939
HSALNG0068856		RNA Gene	1	GC08M135698	2.457602024	https://www.genecards.org/cgi-bin/carddisp.pl?gene=HSALNG0068856
POT1-AS1	POT1 Antisense RNA 1	RNA Gene	15	GC07P124929	2.442903757	https://www.genecards.org/cgi-bin/carddisp.pl?gene=POT1-AS1
PBX2P1	PBX Homeobox 2 Pseudogene 1	Pseudogene	11	GC03P143176	2.440346241	https://www.genecards.org/cgi-bin/carddisp.pl?gene=PBX2P1
MAS1	MAS1 Proto-Oncogene, G Protein-Coupled Receptor	Protein Coding	40	GC06P159906	2.435238361	https://www.genecards.org/cgi-bin/carddisp.pl?gene=MAS1
LINC02629	Long Intergenic Non-Protein Coding RNA 2629	RNA Gene	6	GC10P033910	2.434705019	https://www.genecards.org/cgi-bin/carddisp.pl?gene=LINC02629
RNU6-976P	RNA, U6 Small Nuclear 976, Pseudogene	Pseudogene	5	GC14P084739	2.434705019	https://www.genecards.org/cgi-bin/carddisp.pl?gene=RNU6-976P
lnc-TRIB1-7		RNA Gene	4	GC08P125495	2.434705019	https://www.genecards.org/cgi-bin/carddisp.pl?gene=lnc-TRIB1-7
ENSG00000268473		Pseudogene	2	GC16M086434	2.434705019	https://www.genecards.org/cgi-bin/carddisp.pl?gene=ENSG00000268473
HSALNG0055801		RNA Gene	2	GC07P004174	2.434705019	https://www.genecards.org/cgi-bin/carddisp.pl?gene=HSALNG0055801
ZNF300	Zinc Finger Protein 300	Protein Coding	37	GC05M150894	2.425186634	https://www.genecards.org/cgi-bin/carddisp.pl?gene=ZNF300
ST13P12	ST13, Hsp70 Interacting Protein Pseudogene 12	Pseudogene	6	GC05P082968	2.425083399	https://www.genecards.org/cgi-bin/carddisp.pl?gene=ST13P12
MIR190B	MicroRNA 190b	RNA Gene	15	GC01M154193	2.424841881	https://www.genecards.org/cgi-bin/carddisp.pl?gene=MIR190B
CLGN	Calmegin	Protein Coding	37	GC04M140388	2.420041561	https://www.genecards.org/cgi-bin/carddisp.pl?gene=CLGN
LINC02789	Long Intergenic Non-Protein Coding RNA 2789	RNA Gene	8	GC01P199149	2.383283615	https://www.genecards.org/cgi-bin/carddisp.pl?gene=LINC02789
IGHG4	Immunoglobulin Heavy Constant Gamma 4 (G4m Marker)	Protein Coding	27	GC14M110309	2.380206108	https://www.genecards.org/cgi-bin/carddisp.pl?gene=IGHG4
ARPC4	Actin Related Protein 2/3 Complex	Protein Coding	38	GC03P009792	2.371128082	https://www.genecards.org/cgi-bin/carddisp.pl?gene=ARPC4
ENSG00000286231		Protein Coding	5	GC01P220787	2.362083912	https://www.genecards.org/cgi-bin/carddisp.pl?gene=ENSG00000286231
PSAPL1	Prosaposin Like 1	Protein Coding	33	GC04M007434	2.347115993	https://www.genecards.org/cgi-bin/carddisp.pl?gene=PSAPL1
ENSG00000248537		RNA Gene	7	GC05P009363	2.347115993	https://www.genecards.org/cgi-bin/carddisp.pl?gene=ENSG00000248537
ENSG00000250001		RNA Gene	7	GC05M009519	2.347115993	https://www.genecards.org/cgi-bin/carddisp.pl?gene=ENSG00000250001
HNRNPA1P58	Heterogeneous Nuclear Ribonucleoprotein A1 Pseudogene 58	Pseudogene	4	GC06P024003	2.347115993	https://www.genecards.org/cgi-bin/carddisp.pl?gene=HNRNPA1P58
LOC101060035	DnaJ Homolog Subfamily C Member 24 Pseudogene	Pseudogene	4	GC05M133720	2.347115993	https://www.genecards.org/cgi-bin/carddisp.pl?gene=LOC101060035

HSALNG0040068		RNA Gene	4	GC05M009421	2.347115993	https://www.genecards.org/cgi-bin/carddisp.pl?gene=HSALNG0040068
lnc-PPP1CC-3		RNA Gene	4	GC12M110993	2.347115993	https://www.genecards.org/cgi-bin/carddisp.pl?gene=lnc-PPP1CC-3
LINC00888	Long Intergenic Non-Protein Coding RNA 888	RNA Gene	13	GC03P183447	2.341049671	https://www.genecards.org/cgi-bin/carddisp.pl?gene=LINC00888
LINC01090	Long Intergenic Non-Protein Coding RNA 1090	RNA Gene	13	GC02M187712	2.334821939	https://www.genecards.org/cgi-bin/carddisp.pl?gene=LINC01090
ENSG00000274121		RNA Gene	5	GC01P231814	2.334821939	https://www.genecards.org/cgi-bin/carddisp.pl?gene=ENSG00000274121
MIR378C	MicroRNA 378c	RNA Gene	13	GC10M130962	2.284116507	https://www.genecards.org/cgi-bin/carddisp.pl?gene=MIR378C
SLC23A4P	Solute Carrier Family 23 Member 4, Pseudogene	Pseudogene	7	GC07M135247	2.283819199	https://www.genecards.org/cgi-bin/carddisp.pl?gene=SLC23A4P
H2BC18	H2B Clustered Histone 18	Protein Coding	27	GC01M150113	2.279964924	https://www.genecards.org/cgi-bin/carddisp.pl?gene=H2BC18
MIR4772	MicroRNA 4772	RNA Gene	13	GC02P102432	2.269135475	https://www.genecards.org/cgi-bin/carddisp.pl?gene=MIR4772
RAB24	RAB24, Member RAS Oncogene Family	Protein Coding	37	GC05M177301	2.254528522	https://www.genecards.org/cgi-bin/carddisp.pl?gene=RAB24
HMCES	5-Hydroxymethylcytosine Binding, ES Cell Specific	Protein Coding	31	GC03P129278	2.237731934	https://www.genecards.org/cgi-bin/carddisp.pl?gene=HMCES
CPB2-AS1	CPB2 Antisense RNA 1	RNA Gene	14	GC13P046052	2.232967854	https://www.genecards.org/cgi-bin/carddisp.pl?gene=CPB2-AS1
LINC01029	Long Intergenic Non-Protein Coding RNA 1029	RNA Gene	12	GC18M077972	2.232967854	https://www.genecards.org/cgi-bin/carddisp.pl?gene=LINC01029
HDHD5-AS1	HDHD5 Antisense RNA 1	RNA Gene	12	GC22P017160	2.232967854	https://www.genecards.org/cgi-bin/carddisp.pl?gene=HDHD5-AS1
LINC02534	Long Intergenic Non-Protein Coding RNA 2534	RNA Gene	9	GC06M115635	2.232967854	https://www.genecards.org/cgi-bin/carddisp.pl?gene=LINC02534
LINC02392	Long Intergenic Non-Protein Coding RNA 2392	RNA Gene	9	GC12P090282	2.232967854	https://www.genecards.org/cgi-bin/carddisp.pl?gene=LINC02392
SNORA63E	Small Nucleolar RNA, H/ACA Box 63E	RNA Gene	9	GC03P183457	2.232967854	https://www.genecards.org/cgi-bin/carddisp.pl?gene=SNORA63E
LINC01818	Long Intergenic Non-Protein Coding RNA 1818	RNA Gene	8	GC02P150170	2.232967854	https://www.genecards.org/cgi-bin/carddisp.pl?gene=LINC01818
SNORA63D	Small Nucleolar RNA, H/ACA Box 63D	RNA Gene	8	GC03P183470	2.232967854	https://www.genecards.org/cgi-bin/carddisp.pl?gene=SNORA63D
ENSG00000243273		RNA Gene	8	GC03P150764	2.232967854	https://www.genecards.org/cgi-bin/carddisp.pl?gene=ENSG00000243273
ENSG00000251152		RNA Gene	7	GC04P014575	2.232967854	https://www.genecards.org/cgi-bin/carddisp.pl?gene=ENSG00000251152
ENSG00000249664		RNA Gene	7	GC05M083012	2.232967854	https://www.genecards.org/cgi-bin/carddisp.pl?gene=ENSG00000249664
ENSG00000251186		RNA Gene	7	GC04M008455	2.232967854	https://www.genecards.org/cgi-bin/carddisp.pl?gene=ENSG00000251186
ENSG00000260234		Protein Coding	7	GC03M150882	2.232967854	https://www.genecards.org/cgi-bin/carddisp.pl?gene=ENSG00000260234
LINC02822	Long Intergenic Non-Protein Coding RNA 2822	RNA Gene	6	GC12P090411	2.232967854	https://www.genecards.org/cgi-bin/carddisp.pl?gene=LINC02822
ENSG00000252040		RNA Gene	6	GC16M068630	2.232967854	https://www.genecards.org/cgi-bin/carddisp.pl?gene=ENSG00000252040
ENSG00000253092		RNA Gene	6	GC03P183452	2.232967854	https://www.genecards.org/cgi-bin/carddisp.pl?gene=ENSG00000253092
ENSG00000253354		RNA Gene	6	GC08P040370	2.232967854	https://www.genecards.org/cgi-bin/carddisp.pl?gene=ENSG00000253354
ENSG00000272479		RNA Gene	6	GC08M040519	2.232967854	https://www.genecards.org/cgi-bin/carddisp.pl?gene=ENSG00000272479
ENSG00000234481		RNA Gene	6	GC01P036773	2.232967854	https://www.genecards.org/cgi-bin/carddisp.pl?gene=ENSG00000234481
ENSG00000241679		RNA Gene	6	GC03P143123	2.232967854	https://www.genecards.org/cgi-bin/carddisp.pl?gene=ENSG00000241679
RPL35AP33	Ribosomal Protein L35a Pseudogene 33	Pseudogene	6	GC16M068489	2.232967854	https://www.genecards.org/cgi-bin/carddisp.pl?gene=RPL35AP33
RNU6-655P	RNA, U6 Small Nuclear 655, Pseudogene	Pseudogene	6	GC18P078036	2.232967854	https://www.genecards.org/cgi-bin/carddisp.pl?gene=RNU6-655P
LGMNP1	Legumain Pseudogene 1	Pseudogene	6	GC13M064957	2.232967854	https://www.genecards.org/cgi-bin/carddisp.pl?gene=LGMNP1
ENSG00000248112		RNA Gene	6	GC05M082907	2.232967854	https://www.genecards.org/cgi-bin/carddisp.pl?gene=ENSG00000248112
HNRNPA3P5	Heterogeneous Nuclear Ribonucleoprotein A3 Pseudogene 5	Pseudogene	5	GC13M065787	2.232967854	https://www.genecards.org/cgi-bin/carddisp.pl?gene=HNRNPA3P5
ENSG00000272823		RNA Gene	5	GC01M220828	2.232967854	https://www.genecards.org/cgi-bin/carddisp.pl?gene=ENSG00000272823
ENSG00000259080		RNA Gene	5	GC02P027583	2.232967854	https://www.genecards.org/cgi-bin/carddisp.pl?gene=ENSG00000259080
ENSG00000254171		RNA Gene	5	GC05P166128	2.232967854	https://www.genecards.org/cgi-bin/carddisp.pl?gene=ENSG00000254171
ENSG00000251730		RNA Gene	5	GC03P183454	2.232967854	https://www.genecards.org/cgi-bin/carddisp.pl?gene=ENSG00000251730
ENSG00000197462		RNA Gene	5	GC07P125917	2.232967854	https://www.genecards.org/cgi-bin/carddisp.pl?gene=ENSG00000197462
ENSG00000234034		RNA Gene	5	GC21M022500	2.232967854	https://www.genecards.org/cgi-bin/carddisp.pl?gene=ENSG00000234034
ENSG00000235503		RNA Gene	5	GC07P083355	2.232967854	https://www.genecards.org/cgi-bin/carddisp.pl?gene=ENSG00000235503
LOC107987158	Uncharacterized LOC107987158	Protein Coding	5	GC11M003142	2.232967854	https://www.genecards.org/cgi-bin/carddisp.pl?gene=LOC107987158
MRPL45P1	Mitochondrial Ribosomal Protein L45 Pseudogene 1	Pseudogene	5	GC02M088428	2.232967854	https://www.genecards.org/cgi-bin/carddisp.pl?gene=MRPL45P1
RPL23AP20	Ribosomal Protein L23a Pseudogene 20	Pseudogene	5	GC01P241916	2.232967854	https://www.genecards.org/cgi-bin/carddisp.pl?gene=RPL23AP20
RNU6-	RNA, U6 Small Nuclear 1172, Pseudogene	Pseudogene	5	GC08M096431	2.232967854	https://www.genecards.org/cgi-bin/carddisp.pl?gene=RNU6-1172P
lnc-PNPLA5-3		RNA Gene	5	GC22M048943	2.232967854	https://www.genecards.org/cgi-bin/carddisp.pl?gene=lnc-PNPLA5-3
ENSG00000271682		Pseudogene	4	GC03M183462	2.232967854	https://www.genecards.org/cgi-bin/carddisp.pl?gene=ENSG00000271682
ENSG00000233983		Pseudogene	4	GC01P099464	2.232967854	https://www.genecards.org/cgi-bin/carddisp.pl?gene=ENSG00000233983
lnc-TSNAX-		RNA Gene	4	GC01P231693	2.232967854	https://www.genecards.org/cgi-bin/carddisp.pl?gene=lnc-TSNAX-2
SDR42E1P5	Short Chain Dehydrogenase/Reductase Family 42E, Member 1 Pseudogene 5	Pseudogene	4	GC02P102412	2.232967854	https://www.genecards.org/cgi-bin/carddisp.pl?gene=SDR42E1P5
ENSG00000288092		RNA Gene	4	GC12P103013	2.232967854	https://www.genecards.org/cgi-bin/carddisp.pl?gene=ENSG00000288092
lnc-FAM122A-1		RNA Gene	4	GC09P068711	2.232967854	https://www.genecards.org/cgi-bin/carddisp.pl?gene=lnc-FAM122A-1
lnc-MRGPRG-1		RNA Gene	4	GC11M003146	2.232967854	https://www.genecards.org/cgi-bin/carddisp.pl?gene=lnc-MRGPRG-1
lnc-RHOB-		RNA Gene	4	GC02P020182	2.232967854	https://www.genecards.org/cgi-bin/carddisp.pl?gene=lnc-RHOB-11
lnc-SLC46A3-1		RNA Gene	4	GC13M028701	2.232967854	https://www.genecards.org/cgi-bin/carddisp.pl?gene=lnc-SLC46A3-1
lnc-WDR91-		RNA Gene	4	GC07M135225	2.232967854	https://www.genecards.org/cgi-bin/carddisp.pl?gene=lnc-WDR91-4
HSALNG0071703		RNA Gene	4	GC09M068727	2.232967854	https://www.genecards.org/cgi-bin/carddisp.pl?gene=HSALNG0071703
lnc-MYL2-4		RNA Gene	4	GC12M110918	2.232967854	https://www.genecards.org/cgi-bin/carddisp.pl?gene=lnc-MYL2-4
MN309223		RNA Gene	4	GC11P003129	2.232967854	https://www.genecards.org/cgi-bin/carddisp.pl?gene=MN309223
ENSG00000275383		RNA Gene	3	GC16P068591	2.232967854	https://www.genecards.org/cgi-bin/carddisp.pl?gene=ENSG00000275383
ENSG00000225906		Pseudogene	3	GC21P022882	2.232967854	https://www.genecards.org/cgi-bin/carddisp.pl?gene=ENSG00000225906

ENSG00000 231984		Pseudogene	3	GC01P199753	2.232967854	https://www.genecards.org/cgi-bin/carddisp.pl?gene=ENSG00000231984
lnc-TRIB1-8		RNA Gene	3	GC08P125497	2.232967854	https://www.genecards.org/cgi-bin/carddisp.pl?gene=lnc-TRIB1-8
HSALNG00 17419		RNA Gene	3	GC02P102419	2.232967854	https://www.genecards.org/cgi-bin/carddisp.pl?gene=HSALNG0017419
ENSG00000 287720		RNA Gene	3	GC03P003918	2.232967854	https://www.genecards.org/cgi-bin/carddisp.pl?gene=ENSG00000287720
lnc-BICD1-6		RNA Gene	3	GC12P031995	2.232967854	https://www.genecards.org/cgi-bin/carddisp.pl?gene=lnc-BICD1-6
HSALNG00 23242		RNA Gene	3	GC02M235672	2.232967854	https://www.genecards.org/cgi-bin/carddisp.pl?gene=HSALNG0023242
HSALNG01 13292		RNA Gene	3	GC16P086387	2.232967854	https://www.genecards.org/cgi-bin/carddisp.pl?gene=HSALNG0113292
HSALNG01 13293		RNA Gene	3	GC16M086387	2.232967854	https://www.genecards.org/cgi-bin/carddisp.pl?gene=HSALNG0113293
NONHSAG0 13110.2		RNA Gene	3	GC13P028704	2.232967854	https://www.genecards.org/cgi-bin/carddisp.pl?gene=NONHSAG013110.2
lnc-SLC22A18-7		RNA Gene	3	GC11P003146	2.232967854	https://www.genecards.org/cgi-bin/carddisp.pl?gene=lnc-SLC22A18-7
HSALNG00 71704		RNA Gene	3	GC09M068734	2.232967854	https://www.genecards.org/cgi-bin/carddisp.pl?gene=HSALNG0071704
hsa-miR-5096-076		RNA Gene	3	GC07M004224	2.232967854	https://www.genecards.org/cgi-bin/carddisp.pl?gene=hsa-miR-5096-076
lnc-KLHL24-6		RNA Gene	3	GC03P183484	2.232967854	https://www.genecards.org/cgi-bin/carddisp.pl?gene=lnc-KLHL24-6
piR-50443-100		RNA Gene	3	GC13P046157	2.232967854	https://www.genecards.org/cgi-bin/carddisp.pl?gene=piR-50443-100
piR-61101-044		RNA Gene	3	GC10M015274	2.232967854	https://www.genecards.org/cgi-bin/carddisp.pl?gene=piR-61101-044
ENSG00000 280306		Uncategorized	2	GC16P020487	2.232967854	https://www.genecards.org/cgi-bin/carddisp.pl?gene=ENSG00000280306
ENSG00000 285529		RNA Gene	2	GC10P130525	2.232967854	https://www.genecards.org/cgi-bin/carddisp.pl?gene=ENSG00000285529
ENSG00000 258506		Pseudogene	2	GC14P084951	2.232967854	https://www.genecards.org/cgi-bin/carddisp.pl?gene=ENSG00000258506
ENSG00000 271078		Pseudogene	2	GC15M032251	2.232967854	https://www.genecards.org/cgi-bin/carddisp.pl?gene=ENSG00000271078
HSALNG00 11461		RNA Gene	2	GC01M231809	2.232967854	https://www.genecards.org/cgi-bin/carddisp.pl?gene=HSALNG0011461
LOC105376 433	Uncharacterized LOC105376433	RNA Gene	2	GC10P015236	2.232967854	https://www.genecards.org/cgi-bin/carddisp.pl?gene=LOC105376433
LOC107986 000	Uncharacterized LOC107986000	RNA Gene	2	GC02P235730	2.232967854	https://www.genecards.org/cgi-bin/carddisp.pl?gene=LOC107986000
lnc-RASGRP1-1		RNA Gene	2	GC15M038754	2.232967854	https://www.genecards.org/cgi-bin/carddisp.pl?gene=lnc-RASGRP1-1
HSALNG00 32698		RNA Gene	2	GC04P007752	2.232967854	https://www.genecards.org/cgi-bin/carddisp.pl?gene=HSALNG0032698
HSALNG00 43115		RNA Gene	2	GC05M082908	2.232967854	https://www.genecards.org/cgi-bin/carddisp.pl?gene=HSALNG0043115
HSALNG00 43127		RNA Gene	2	GC05P083011	2.232967854	https://www.genecards.org/cgi-bin/carddisp.pl?gene=HSALNG0043127
HSALNG00 14027		RNA Gene	2	GC02M031425	2.232967854	https://www.genecards.org/cgi-bin/carddisp.pl?gene=HSALNG0014027
HSALNG00 10758		RNA Gene	2	GC01P220796	2.232967854	https://www.genecards.org/cgi-bin/carddisp.pl?gene=HSALNG0010758
HSALNG00 31159		RNA Gene	2	GC03P183480	2.232967854	https://www.genecards.org/cgi-bin/carddisp.pl?gene=HSALNG0031159
HSALNG00 32696		RNA Gene	2	GC04M007742	2.232967854	https://www.genecards.org/cgi-bin/carddisp.pl?gene=HSALNG0032696
HSALNG00 56551		RNA Gene	2	GC07M021497	2.232967854	https://www.genecards.org/cgi-bin/carddisp.pl?gene=HSALNG0056551
HSALNG00 61543		RNA Gene	2	GC07P135291	2.232967854	https://www.genecards.org/cgi-bin/carddisp.pl?gene=HSALNG0061543
NONHSAG0 18421.2		RNA Gene	2	GC16P005964	2.232967854	https://www.genecards.org/cgi-bin/carddisp.pl?gene=NONHSAG018421.2
piR-43105-110		RNA Gene	2	GC12P031998	2.232967854	https://www.genecards.org/cgi-bin/carddisp.pl?gene=piR-43105-110
piR-48799-012		RNA Gene	2	GC10P015269	2.232967854	https://www.genecards.org/cgi-bin/carddisp.pl?gene=piR-48799-012
RF00017-1289		RNA Gene	2	GC13P046130	2.232967854	https://www.genecards.org/cgi-bin/carddisp.pl?gene=RF00017-1289
piR-55036-040		RNA Gene	2	GC02P031489	2.232967854	https://www.genecards.org/cgi-bin/carddisp.pl?gene=piR-55036-040
lnc-MARC2-		RNA Gene	2	GC01P220804	2.232967854	https://www.genecards.org/cgi-bin/carddisp.pl?gene=lnc-MARC2-2
piR-31534-137		RNA Gene	2	GC13M028707	2.232967854	https://www.genecards.org/cgi-bin/carddisp.pl?gene=piR-31534-137
piR-39858-368		RNA Gene	2	GC20M016549	2.232967854	https://www.genecards.org/cgi-bin/carddisp.pl?gene=piR-39858-368
piR-43106-189		RNA Gene	2	GC07P135282	2.232967854	https://www.genecards.org/cgi-bin/carddisp.pl?gene=piR-43106-189
LOC107984 123	Uncharacterized LOC107984123	RNA Gene	1	GC07M135264	2.232967854	https://www.genecards.org/cgi-bin/carddisp.pl?gene=LOC107984123
LOC105377 705	Uncharacterized LOC105377705	RNA Gene	1	GC05M166230	2.232967854	https://www.genecards.org/cgi-bin/carddisp.pl?gene=LOC105377705
ENSG00000 219736		Pseudogene	1	GC06M074850	2.232967854	https://www.genecards.org/cgi-bin/carddisp.pl?gene=ENSG00000219736
ENSG00000 229119		Pseudogene	1	GC05P166383	2.232967854	https://www.genecards.org/cgi-bin/carddisp.pl?gene=ENSG00000229119
ENSG00000 220748		Pseudogene	1	GC06P023972	2.232967854	https://www.genecards.org/cgi-bin/carddisp.pl?gene=ENSG00000220748
LOC102724 253	Uncharacterized LOC102724253	RNA Gene	1	GC15M038755	2.232967854	https://www.genecards.org/cgi-bin/carddisp.pl?gene=LOC102724253
RF00026-		RNA Gene	1	GC13M028705	2.232967854	https://www.genecards.org/cgi-bin/carddisp.pl?gene=RF00026-272
HSALNG01 05127-003		RNA Gene	1	GC15P038755	2.232967854	https://www.genecards.org/cgi-bin/carddisp.pl?gene=HSALNG0105127-003
HSP90AA2P	Heat Shock Protein 90 Alpha Family Class A Member 2, Pseudogene	Pseudogene	19	GC11M027888	2.202373981	https://www.genecards.org/cgi-bin/carddisp.pl?gene=HSP90AA2P
DUSP14	Dual Specificity Phosphatase 14	Protein Coding	38	GC17P037489	2.19440794	https://www.genecards.org/cgi-bin/carddisp.pl?gene=DUSP14
ZC3H12A	Zinc Finger CCCH-Type Containing 12A	Protein Coding	37	GC01P037474	2.164163351	https://www.genecards.org/cgi-bin/carddisp.pl?gene=ZC3H12A
TSKU	Tsukushi, Small Leucine Rich	Protein Coding	34	GC11P076782	2.163715601	https://www.genecards.org/cgi-bin/carddisp.pl?gene=TSKU
TRIM47	Tripartite Motif Containing 47	Protein Coding	34	GC17M075874	2.146330357	https://www.genecards.org/cgi-bin/carddisp.pl?gene=TRIM47
SLC25A48	Solute Carrier Family 25 Member 48	Protein Coding	32	GC05P135579	2.117699146	https://www.genecards.org/cgi-bin/carddisp.pl?gene=SLC25A48
DNAJC11	DnaJ Heat Shock Protein Family (Hsp40) Member C11	Protein Coding	35	GC01M006634	2.115141392	https://www.genecards.org/cgi-bin/carddisp.pl?gene=DNAJC11
SNRNP40	Small Nuclear Ribonucleoprotein U5 Subunit 40	Protein Coding	37	GC01M031259	2.111979246	https://www.genecards.org/cgi-bin/carddisp.pl?gene=SNRNP40
YIPF6	Yip1 Domain Family Member 6	Protein Coding	34	GC0XP068498	2.097386837	https://www.genecards.org/cgi-bin/carddisp.pl?gene=YIPF6
RPL17-C18orf32	RPL17-C18orf32 Readthrough	Protein Coding	21	GC18M049481	2.043186188	https://www.genecards.org/cgi-bin/carddisp.pl?gene=RPL17-C18orf32

LINC00333	Long Intergenic Non-Protein Coding RNA 333	RNA Gene	13	GC13P084140	2.035597801	https://www.genecards.org/cgi-bin/carddisp.pl?gene=LINC00333
PDIA2	Protein Disulfide Isomerase Family A Member 2	Protein Coding	39	GC16P005498	2.022377729	https://www.genecards.org/cgi-bin/carddisp.pl?gene=PDIA2
TXNDC5	Thioredoxin Domain Containing 5	Protein Coding	39	GC06M007893	2.007545948	https://www.genecards.org/cgi-bin/carddisp.pl?gene=TXNDC5
PHLPP2	PH Domain And Leucine Rich Repeat Protein Phosphatase 2	Protein Coding	41	GC16M071637	2.00033474	https://www.genecards.org/cgi-bin/carddisp.pl?gene=PHLPP2
LOC109279247	FGF21/FUT1 Promoter Region	Biological Region	1	GC19P048783	1.936319113	https://www.genecards.org/cgi-bin/carddisp.pl?gene=LOC109279247
PRMT3	Protein Arginine Methyltransferase 3	Protein Coding	43	GC11P020409	1.901653171	https://www.genecards.org/cgi-bin/carddisp.pl?gene=PRMT3
FAM166B	Family With Sequence Similarity 166 Member B	Protein Coding	30	GC09M035554	1.870354414	https://www.genecards.org/cgi-bin/carddisp.pl?gene=FAM166B
CR1L	Complement C3b/C4b Receptor 1 Like	Protein Coding	34	GC01P207646	1.857897997	https://www.genecards.org/cgi-bin/carddisp.pl?gene=CR1L
SAP18	Sin3A Associated Protein 18	Protein Coding	40	GC13P021140	1.847493052	https://www.genecards.org/cgi-bin/carddisp.pl?gene=SAP18
IGKV2D-23	Immunoglobulin Kappa Variable 2D-23 (Pseudogene)	Pseudogene	5	GC02P090720	1.844097137	https://www.genecards.org/cgi-bin/carddisp.pl?gene=IGKV2D-23
NMBR	Neuromedin B Receptor	Protein Coding	43	GC06M142059	1.779913902	https://www.genecards.org/cgi-bin/carddisp.pl?gene=NMBR
SUCNR1	Succinate Receptor 1	Protein Coding	41	GC03P151873	1.748568058	https://www.genecards.org/cgi-bin/carddisp.pl?gene=SUCNR1
SGIP1	SH3GL Interacting Endocytic Adaptor 1	Protein Coding	35	GC01P066533	1.687782764	https://www.genecards.org/cgi-bin/carddisp.pl?gene=SGIP1
MIR103B2	MicroRNA 103b-2	RNA Gene	9	GC20M003899	1.669028759	https://www.genecards.org/cgi-bin/carddisp.pl?gene=MIR103B2
MIR103B1	MicroRNA 103b-1	RNA Gene	8	GC05P168560	1.669028759	https://www.genecards.org/cgi-bin/carddisp.pl?gene=MIR103B1
DNAJC10	DnaJ Heat Shock Protein Family (Hsp40) Member C10	Protein Coding	38	GC02P182716	1.648108721	https://www.genecards.org/cgi-bin/carddisp.pl?gene=DNAJC10
HERC4	HECT And RLD Domain Containing E3 Ubiquitin Protein Ligase 4	Protein Coding	37	GC10M067921	1.544003963	https://www.genecards.org/cgi-bin/carddisp.pl?gene=HERC4
FAM174A	Family With Sequence Similarity 174 Member A	Protein Coding	32	GC05P100535	1.195732713	https://www.genecards.org/cgi-bin/carddisp.pl?gene=FAM174A
MIR3666	MicroRNA 3666	RNA Gene	11	GC07P114653	0.87816745	https://www.genecards.org/cgi-bin/carddisp.pl?gene=MIR3666
MIR4668	MicroRNA 4668	RNA Gene	12	GC09P111932	0.84098649	https://www.genecards.org/cgi-bin/carddisp.pl?gene=MIR4668
TM6SF1	Transmembrane 6 Superfamily Member 1	Protein Coding	33	GC15P083357	0.515707552	https://www.genecards.org/cgi-bin/carddisp.pl?gene=TM6SF1
