# Supplementary material for: Explore the Mechanism of Astragalus mongholicus Bunge against Nonalcoholic Fatty Liver Disease Based on Network Pharmacology and Experimental Verification
Source: Gastroenterol Res Pract. 2022 Apr 5;2022:4745042. doi: 10.1155/2022/4745042 (PMC9005278; doi:10.1155/2022/4745042)
Supplement: Supplementary Materials — Table S1: the bioactive components and corresponding targets of ABM. Table S2: information of NAFLD-related targets collected from the GeneCards database. Table S3: the common targets of ABM and NAFLD. Table S4: the 383 biological process terms. Table S5: the 40 cellular component terms. Table S6: the 63 molecular function terms. [file 4745042.f1.zip › 4745042.f1/Table S3.pdf]

AM-related targets	NAFLD-related targets	common targets of AM and NAFLD
PGR	PNPLA3	XDH
NOS2	IL6	VCAM1
PTGS1	APOE	TP53
AR	PRMT7	TNF
SCN5A	MIR122	THBD
PTGS2	TNF	TGFB1
ESR2	TP53	STAT1
DPP4	PKD2	SPP1
HSP90AA1	INS	SOD1
CDK2	HFE	SLC6A4
CHEK1	ALB	SLC6A3
PRSS1	IL10	SLC2A4
NCOA2	PPARG	SIRT1
CALM	HADHA	SERPINE1
CHRM3	TGFB1	SELE
CHRM1	LMNA	RXRA
GABRA2	MARS1	RELA
GABRA3	FABP1	PTPN1
CHRM2	SMPD1	PTGS2
ADRA1B	SLC17A5	PTEN
GABRA1	SERPINA1	PRSS1
GRIA2	FASN	PRKCA
GABRA6	MIR21	PPP3CA
GABRA5	ATP7B	PPARG
IGHG1	GPT	PPARD
LYZ	FAS	PPARA
COBT	ABCB11	PON1
RXRA	GANAB	PLAU
PDE3A	APOA1	OPRM1
SLC6A2	CTNNB1	OPRD1
NOR51B	PPARA	NR1I3
ESR1	IL1B	NR1I2
PPARG	MIR34A	NQO1
PTPN1	APOB	NOS2
MAPK14	NAFLD1	NFE2L2
GSK3B	ADIPOQ	MYC
PIK3CG	NOD2	MT-ND6
PRKACA	MIR10B	MPO
PIM1	LIPA	MMP9
CCNA2	PIK3CA	MMP2
PYGM	CPT2	MMP1
PPARD	TLR4	MGAM
AKR1B1	SQSTM1	MAPK8
NCOA1	CRP	MAPK14
F7	NR1H4	LDLR
F2	MFN2	JUN
NOS3	CPT1A	IRF1
ACHE	ABCA1	INSR
MAOB	APP	IL6
RELA	MIR17	IL1B
XDH	G6PC1	IL1A
NCF1	KRT18	IKBKB
LDLR	MIR155	IGHG1
ADRB1	CXCL8	IGFBP3
HTR3A	AKT1	IFNG
ADRA2C	LPL	ICAM1

ADRB2
ADRA1D
OPRM1
DRD1
KCNH2
F10
CHRM5
CHRM4
OPRD1
HTR2A
ADRA1A
SLC6A3
SLC6A4
KCNMA1
TOP2A
CHRNA7
KDR
MET
PKIA
LACTB
JUN
IL4R
SIRT1
ATP5B
MT-ND6
HSD3B2
HSD3B1
IKBKB
AKT1
BCL2
BAX
TNF
AHSA1
CASP3
MAPK8
MMP1
STAT1
CDK1
HMOX1
CYP3A4
UBE2O
CYP1A1
ICAM1
SELE
VCAM1
NR1I2
CYP1B1
ALOX5
HAS2
AHR
PSMD3
SLC2A4
NR1I3
INSR
DIO1
PPP3CA
PRXC1A
GSTM1
PTEN
CD36
CTLA4
GGT1
HNF4A
CYP2E1
LEP
VWF
VCP
FABP4
SREBF1
F2
MTTP
INSR
IFNG
NR1H3
CFTR
ACE
TERT
FABP2
MIR146A
ACOX1
ABCB4
SERPINE1
EGFR
CASP8
IGF1
CYBB
STAT3
RBP4
HMOX1
HLA-DQB1
HBB
TTR
IRS1
EGF
SLC25A13
IL1A
AFP
TLR2
KRT8
ABHD5
MIR27A
LEPR
CASP3
CCL2
SOD1
MTHFR
BSCL2
PPARGC1A
MIR29A
VEGFA
MIR200B
MIR203A
RETN
PON1
JAG1
MMP9
HTR2A
HSPB1
HSPA5
HSP90AA1
HMOX1
GSTM1
GSK3B
GABRA2
FOS
F3
F2
ESR1
ELK1
EGFR
DRD1
DPP4
CYP3A4
CYP1B1
CYP1A1
CXCL8
CXCL11
CXCL10
CTSD
CRP
COL3A1
CHUK
CHRM3
CDKN1A
CCND1
CCL2
CAV1
CASP9
CASP8
CASP3
BIRC5
BCL2
BAX
AKT1
AHSA1
AHR
ADRB2
ADRB1
ACACA

GSTM2
AKR1C3
SLPI
MMP3
EGFR
CCND1
BCL2L1
FOS
CDKN1A
Eif6
CASP9
PLAU
MMP2
MMP9
MAPK1
IL10RA
RB1
IL6
TP53
ELK1
CHUK
ODC1
CASP8
TOP1
RAF1
SOD1
PRKCA
HIF1AN
RUNX1T1
HSPA5
ERBB2
ACACA
CAV1
MYC
F3
GJA1
IL1B
CCL2
PTGER3
CXCL8
Prkcb
BIRC5
DUOXA2
HSPB1
TGFB1
MGAM
IL2
CCNB1
PLAT
THBD
SERPINE1
IFNG
pteN
IL1A
MPO
ABCA2
NFE2L2
NQO1
GARS1
YARS1
SMAD4
CYP3A4
FADS2
LDLR
FABP3
HSPB1
UCP2
MMP1
MIR22
NOTCH1
IL18
SOD2
MPO
SCD
CYBA
CFH
MLXIPL
CAV1
MAPK8
PNPLA2
TNFRSF1A
CP
SERPINA3
SPP1
MIR20A
BRCA2
UGT1A1
MTOR
RELA
SIRT1
APOC3
DNAJB11
ACACA
ELOVL6
PRKAG2
ABCC2
LIPC
ICAM1
CCND1
MIR126
HAMP
HMGCR
TF
HGF
NFKB1
ANO5
LCAT
CAT
MIR221
PTGS2
TIMP1
IRS2
ESR1
FASLG
IL1RN
XBP1

TNKS
COL3A1
GYRB1
CXCL11
CXCL2
DCAF1
CHEK2
CLDN4
PPARA
HSF1
CRP
CXCL10
SPP1
RUNX2
RASSF1
2F1
E2F2
ACPP
CTSD
IGFBP3
IGF2
CD40LG
IRF1
ERBB3
PON1
PCOLCE
NPEPPS
HK2
NKX3-1
RASA1

PTPN11
MIR132
MIR222
ADIPOR1
CDKN2A
DDIT3
ADIPOR2
LITAF
CYCS
SDHB
STAT1
MT-CYB
BDNF
HP
MIR140
AGT
TM6SF2
MMP2
ENPP1
TGFBR2
FOS
NOS2
MIR181A1
JUN
MYC
FABP12
CYP1A1
SLC27A2
TARDBP
IL17A
SDHD
SLC2A1
NR1H2
GCKR
GFER
MT-CO1
TNFRSF11B
PEMT
COL1A1
MIR107
SLC40A1
CEBPA
CETP
NDUFS1
PLAU
CYP1A2
DGAT1
MIR29C
GSTP1
SLC27A4
IFNA1
AKT2
GSTM1
GCG
GRN
AKR1D1
PCSK9
VDR

HNRNPA1
ALMS1
SMAD3
MIR195
CDKN3
GCK
AHSG
CTSD
SLC27A1
MAP3K5
NLRP3
TFR2
AGTR1
DSP
SLC2A4
DPP4
MT-CO3
SERPINC1
CASR
CES1
APOA5
MIR31
C3
MIR30A
PIK3R1
MIR200A
MIR23B
SOCS3
MIR181B1
BAX
CCN2
THBD
ADH1C
LIPE
F3
MIR199A1
NAMPT
ALDH2
PKLR
CYP7A1
ATP2A2
ASS1
ACTB
NDUFS4
SDHA
FN1
GHRL
SMARCA4
IKBKB
NDUFV1
SREBF2
ACACB
HIF1A
HJV
FGF21
ALPL
GSK3B
CYP2A6

MT-ND3
AGPAT2
MIR224
MSH6
MT-ND6
IL6R
BMP6
HSPD1
FFAR4
TNFRSF1B
PRKAB1
PRKAA1
BCL2
COX5A
H19
ELANE
RARRES2
UGT1A7
VIM
ITCH
AIFM1
CDKN1A
FGF23
PLIN2
MIR200C
FGA
A2M
MIR125A
MT-CO2
BTNL2
LPA
SDHC
SELE
IGF1R
NR1I2
FTL
PDGFRB
UGT1A6
MSR1
NPPB
CXCL10
IFIH1
XDH
ATF4
MYH9
TGFBR1
IDH1
KRT7
KRT19
ADA2
POMC
NFE2L2
ALPP
TLR9
MIR130A
MAPK14
PRKCQ
VCAM1

UGT1A8
NDUFS2
FGF19
ERN1
SPINK1
EIF2AK4
BGLAP
CCL5
TBP
MIR16-1
MIR29B1
IFNA2
NDUFS7
SHH
MIRLET7D
EIF2S1
LCN2
UGT1A9
HBA1
RXRA
SELP
CNR1
CXCR4
MIRLET7C
LGALS3
TET2
EP300
TCF7L2
ADRB2
NDUFV2
MIR33A
MST1
COX6A1
LIVAR
CD14
FLT1
LRP6
MIR26A1
EIF2AK3
TNFAIP3
IGFBP3
MIR141
MIR183
NDUFB9
ERCC2
TNFSF11
MIR144
MDM2
MIR451A
CPS1
DGAT2
SHBG
CTSB
NR5A2
DNMT1
CD44
APOA2
CHIT1

COL3A1
IL21
CLU
ABCB7
UGT1A4
NDUFS3
GHR
MYD88
MIR182
SLC10A1
COPA
UQCRB
SCARB1
PC
PPARD
LOX
NGF
PLA2G4A
CDH2
MIRLET7E
IL1R1
IGFBP1
UGT1A10
CYP4A11
MIR151A
TIMP2
PPIG
GH1
ACSL3
SLC6A3
CDK4
CRYAA
ERBB4
TNFSF10
EPHX1
UGT1A3
IL18BP
HSPA5
SLC6A4
SIRT3
NPY
SPARC
MIR378A
ADH1B
KHK
CEL
MIR192
GNMT
MIR429
UCP3
MEG3
CRAT
NDUFA13
CYP1B1
NEU1
IL15
NAT2
FNDC5

NDUFA1
GLUD1
CASP9
GC
PRKAA2
GSTT1
EIF4G1
CYP2B6
EPRS1
CHUK
SERPINF2
SERPINA6
PARP1
MIR30D
AGER
MIR483
DKK1
S100A9
SDCCAG8
EZH2
LRPPRC
MIR205
CCL3
SP1
CEBPB
SOST
CSF1
NDUFS8
FGFR4
DRD2
TMPRSS6
RAC1
HSD11B1
RPS27A
UCP1
PIK3CD
CCR1
NPC1L1
MIR27B
NR0B2
IFNL3
LOC110806263
MIR214
NR1I3
AHR
KL
PRL
GSR
SUMF1
SERPINH1
NDUFA6
ABCC3
LTBP3
HTR2A
GPX1
AKT3
BLVRA
DNMT3A

NDUFA10
MIR9-1
EPHX2
CD163
COX4I1
HSP90AA1
IRGM
IRF1
HMGB1
DYSF
DNASE1
NDUFB8
FGB
MMP13
CXCR3
FLI1
LMBRD1
SERPINF1
NQO1
PRKCA
UGT1A5
ANGPTL3
NDUFB11
NDUFS6
BIRC5
AKR1A1
MERTK
MAPK10
PRSS1
CCK
HSPG2
GDF15
CDC42
MAOA
NDUFA12
GSS
COX6B1
NDUFB3
HDAC9
ATP1A1
ABCG1
GOT2
CHI3L1
MATR3
CD55
CCL20
COX8A
UQCRC2
THBS1
ETS1
PEPD
ACSM2B
EIF2AK2
MIR149
PLCB1
FOXO1
CA2
DDX3X

SNAI2
PLIN1
ALOX12
FARSA
RPS6KB1
DICER1
BMPR1A
CNTNAP2
CPOX
DNAH11
PIK3R2
IRF8
CYP17A1
PLAUR
SDC1
PLTP
POR
ADRB3
ALAD
TGM2
STAR
SMAD7
MYH14
MT-TT
KEAP1
NOS1
PPP3CA
CD68
GOLM1
KLF6
TPM2
DBH
CES2
HCFC1
MIR15B
LEPQTL1
IL6ST
UBD
PRDX1
NEAT1
SULT2A1
MIR24-1
HDAC1
FDFT1
CYP4F2
MIR154
RPS6KA3
CD59
LNCARSR
CASP7
SHC1
HDAC8
STAT5A
ANGPTL8
MIR331
NDUFA11
NDUFAB1
POSTN

SI
GABRG2
NDUFA9
CCL4
OSM
TXNRD2
IRF3
NDUFA2
TLR1
TXN
IL33
CYC1
FYN
GPC4
CANX
IAH1
TSPO
WNT3A
RB1CC1
CRH
PIK3CB
IL17RA
ATF6
ERLIN1
MIR125B1
OGG1
HNRNPK
LBP
APPL1
PHB
CLTC
CCR2
MBOAT7
GCLC
PKM
CIDEC
TPM3
IARS1
ATP5F1E
PTPN1
FTO
ANXA2
OLR1
GSK3A
COX6A2
DRD1
GPBAR1
HSPA1A
E2F1
MIR95
ASCL1
TFEB
ARPC1B
DRD4
IL1RAPL2
TPM1
IL11
NDUFB10

IGFBP2
CFLAR
NTS
DPM1
STUB1
XRCC4
RPL5
CYP2J2
SERPINA7
DRD3
SF3B1
ACE2
MDH2
CDK8
SCAP
COL12A1
PALLD
MAPK9
CLCN2
COX7A2
SRD5A2
HUWE1
LPIN1
SLC25A1
UQCRFS1
MIR187
DHCR24
FGL1
HNRNPU
VTN
FOXC2
GREM1
EGR1
RDH5
LECT2
UBQLN4
CYP4F3
RUNX3
STING1
SPTAN1
IGF2BP2
FOXF1
HYOU1
CNR2
PRPF8
PTX3
ACLY
GAL
EPAS1
BCL2L11
ADH5
P2RX7
UQCRQ
SFRP4
NOX4
PHGDH
TKT
ITGA8

LPCAT3
IFNAR1
CPB2
CLRN1
TLR6
AREG
RARS1
SOD3
P4HB
HNMT
BIRC3
MAP3K7
MIR30C1
ENPP2
JCAD
RTN4
COX4I2
NNMT
IL16
TRAPPC9
SERPINB3
MYL2
IL18RAP
ADH1A
CUL3
TRIB1
JAZF1
OGT
PDK4
GFPT1
USF1
COLGALT1
CYGB
APEX1
MATN3
MIR423
PIK3R3
PDGFA
MLX
NDUFC2
RORA
SLC39A14
MGAM
NLRC4
MIR30B
TRIM21
NOS1AP
SNRNP200
CACNA2D1
PRPF3
PRDX5
GOT1
CYLD
WWTR1
AOC3
AZGP1
SLC25A12
CFL1

TMPO
PARVB
BID
APLN
NCAN
SLC9A9
PER2
COL13A1
ALDH9A1
GLP1R
CD82
TYSND1
FOXA1
PLIN5
DISC1
COMMD1
EFTUD2
PDXK
CDH4
GPI
YWHAE
SLC1A3
COX7B
CYP8B1
ITPR2
TRAP1
MIR103A1
YWHAQ
PRKAG1
MYH10
CXCR5
YY1
PIN1
PRKDC
XRCC6
CASP2
EEA1
CDH5
UQCRC1
HSD17B13
PTPRD
RPL10
HPS1
RPS15
AXL
NCOA3
SLC39A4
SLC4A4
SIRT6
SLC25A22
TNFRSF4
EDA
ADRB1
XRCC5
SAMM50
IMMT
SIK1
ALPI

ERLIN2
CHRM3
LCN1
SERPINA12
CLDN2
TXNRD1
NAT1
SLC34A2
MIR363
EEF1A1
PRKACG
CXCL11
TNC
AKR1B10
OPRM1
RPS17
GPNMB
ITLN1
PPIB
GPC6
PRKAB2
APOF
EIF3F
CISD2
TRAF2
HSP90B1
MYO6
MOGAT2
GRK2
NDUFA4
FUNDC2
SNTA1
AMPH
ZNF365
NDUFC2-KCTD14
AHNAK
CCN1
MIR361
NRG3
ARNT
DMGDH
PSMC2
KAT5
YWHAG
MACROD2
SLC44A1
CCT5
IL27
MIR590
RPL27
LOXL2
TP53BP1
HSPA6
SLC25A47
SIRT4
DCLK1
IGFBP7
AQP9

DROSHA
STEAP4
TAGLN
PZP
TLL1
IGFBP5
PRSS2
NFIC
TRAF1
ATP5F1B
RBFOX1
PPP2CA
TSHB
TRAF3
SRSF1
ATAD3A
IGHG1
FST
LOC110806262
H4-16
GPT2
UGDH
RPS15A
PEG10
DGCR8
OSMR
RPL7
AFAP1-AS1
AUP1
PUF60
MYH2
CD5L
MYO1B
ELK1
LGALS3BP
AQP3
GPLD1
GABRA2
SEMA5A
UBE2D2
CAD
IFNL4
PSMD2
MAP3K11
SIPA1L2
SDC2
MMP11
PARD3
KHDRBS3
CMKLR1
MYF5
SORCS3
HNRNPC
UFM1
CARS1
ADAMTS9
NDRG2
CFD

PTBP1
SLC38A8
ONECUT2
RIPK3
PYCARD
CCL19
RPTOR
HS3ST1
LCP1
TFAP2B
MAP3K8
SLC25A3
MAPK12
PNLIPRP2
YEATS2
DARS1
H1-4
INSIG1
CXCL16
LAMP3
BIRC2
SLC25A10
INSIG2
H4C1
PTPRG
NDUFS5
BNIP3
GLO1
SOCS2
YBX1
IRF2BP2
ATXN2L
YWHAB
ATF3
PRKCE
NDUFA8
UQCR11
CCRL2
NDUFA5
PCLO
PCSK5
MIR103A2
CAMKK2
SVIL
SIRT5
CTBP2
USP7
CYP2A13
TRIB3
NGF-AS1
TPM4
CALD1
ESM1
RPL13
KLB
RPN1
APLNR
RPLP2

NUDT4
TET1
H4C11
H4C8
SLC6A9
SNHG20
H4C4
TPT1
RPL7A
RPL6
PTPRU
FARP1
CBLB
SAT1
YBX3
CCNC
NDUFB7
IL1R2
SRSF6
YIPF1
MIR33B
PABPC1
HNRNPH1
TSBP1-AS1
NFE2L1
RPS11
H4C9
IL34
AP2B1
RPS18
AQP7
SLC3A2
H4C3
MCM3
ST8SIA1
H4C2
H4C13
H4C6
ROCK1
H4C14
H4C15
H4C12
EHBP1L1
PRKAG3
H4C5
BCAT1
FAP
ESRRG
FRK
UQCR10
CYP2F1
LAPTM4A
RPL10A
NCL
TTF2
SLC46A3
TNR
NDUFB6

FETUB
RPL3
RPL12
MRTFA
CTSS
AEBP1
PDGFC
PFKFB3
SUGP1
FAF2
RPS23
YWHAZ
CWF19L1
HSP90AB1
HNRNPF
GCN1
PCBP2
ZP4
RPL30
NMT2
COX5B
INHBA
PCBP1
COX7A2L
HLX
MIR372
CLRN1-AS1
FAM107B
DNAJA1
DISC2
PPP1CA
ANXA9
SORCS2
POMP
HNRNPM
GGT2
TMBIM1
RPS2
SLC25A11
GPN1
CREB5
SERPINA4
NDUFA3
RPS9
BICD1
MYO18A
NRG4
HTR1B
HSPH1
MIR449A
IGF2BP1
TRIM33
DDX1
CTPS1
VIL1
AGAP1
HNRNPA3
TUBA8

CCAR2
AHSA1
RANGAP1
DHX9
HNRNPUL1
TMSB4X
CRTC1
TNFRSF12A
TARBP1
LEPROT
NFE2
BBC3
ITSN1
COX6C
NDUFB4
DDX5
POLR3H
APPL2
RPS4X
PNPLA7
DDX60L
PSG1
FOXK1
MCM5
RPS3A
RPL4
RPL8
HNRNPL
RPL9
MCM7
C1QTNF1
SLC23A1
CCN4
POLR2D
SEL1L3
STK24
PGAM5
STX17
AP2A2
CCT4
MCF2L2
NDUFB5
TXNL1
STMP1
MYO1D
QARS1
UQCRH
NDUFB2
SNRPD2
LOC110366354
CAND1
SLC23A2
ORM1
RPL24
ILF3
CRACR2A
ARRDC3
SLC30A6

ACTR2
RUVBL1
EDC4
G0S2
RNF114
HKDC1
NDUFV3
EIF4A3
TP53BP2
TLCD4-RWDD3
PLPPR4
COX7C
PDIA4
NDUFC1
SERPINB4
NUCB2
MIR873
SFRP5
SLC30A9
COX7A1
SRSF7
PKNOX1
SP4
TUBA1C
SEC16B
CILP2
EIF4A1
GATAD2A
AP2A1
ZNF512
PABIR1
MTARC1
NDUFA7
SENP3
LOC101928118
ACTR3
GRIK3
SIRT7
MIR1296
C2orf16
CCT8
RWDD3
COX8C
SDK1
BMP8B
NDUFB1
TEX36
EIF3B
DDX50
PSME2
NUDT21
OPRD1
FAM171A1
MYO1C
DNAJC15
FAT1
PHF2
MACROH2A2

CHST2
RPS8
RPL23A
ZFP90
SEC16A
MAPK13
IL36A
TCP1
RGS4
RTRAF
RRBP1
SRRM1
CCT2
AATF
PIP5K1B
USP48
RUVBL2
PCSK7
LOC643339
OSBPL5
CCT3
C1QTNF9
TLCD4
SRSF4
GSTK1
ENHO
TMC4
TMBIM6
RBM14
KIF16B
SVIL-AS1
RAB37
NDUFA4L2
DDX17
RND3
TMOD3
CARD6
GSDMD
XPO4
UBE2D3
SF3B3
PDE7B
RNU6-53P
FOXN3
CIDEA
IBTK
EIF3L
TRMT44
SLC9A4
RLN2
NLRP2
PLPP7
WSPAR
USP4
C1QTNF3
UBXN1
LINC00917
ADAMTS9-AS2

CIDEB
EIF3E
ARPC2
KRTAP5-5
KLHL6
HSD17B11
SLC26A6
COX7B2
VSIG4
SNHG18
ILF2
HIGD1A
MYBBP1A
PWWP3A
GGT3P
ANKK1
MAU2
LOC101929710
ZBTB7A
MIR1183
CDH26
AGR3
COL21A1
SMOC2
PABPC4
LRRFIP2
CNOT4
CKAP4
SUB1
BECN2
COX6B2
USP10
LATS2
HSALNG0013825
ACTR5
CCDC63
FAM3B
DDX60
TSNAX-DISC1
SRRM2
GGT5
LINC01405
LINC01016
SH3BP5
MYL6B
ZNF101
RESF1
STRA8
FAM3A
MLXIP
DEFA1
DDX46
KDM4B
UQCRHL
MIR646HG
EIF4A2
ARL2BPP6
ZNF267

ENSG00000231487
C16orf95
lnc-TM6SF2-1
ENSG00000267629
MIR6090
IL36B
ETS1-AS1
ARHGAP8
RSPO3
A2MP1
GLT8D2
RAB32
HIF3A
TMEM97
TRPC4AP
LINC02694
SMIM20
RNU6-961P
DHX15
LINC01354
RUBCNL
GBX2
ENSG00000253111
SDF2L1
EBNA1BP2
HSALNG0013412
lnc-COA6-13
RF00017-457
LINC01679
RTCB
DUSP26
CAPSL
ENSG00000231992
SLC2A8
FECHP1
LINC00322
CENPM
LOC644135
LOC101928135
ENSG00000287092
COP1
LOC389199
INPP4B
ENSG00000228852
ENSG00000287292
ENSG00000254599
HSALNG0133406
PRR5-ARHGAP8
LINC01684
FAIM
ATP6AP1L
LYPLAL1
piR-37045
HSALNG0135823
RAMP3
REPIN1
H2BC11
TPI1P1

TEX36-AS1
ENSG00000255046
ENSG00000222042
OTX2P1
lnc-JCAD-2
ENSG00000237936
THNSL2
HCAR2
STARP1
ENSG00000234389
ENSG00000249631
HSALNG0088100
HSALNG0088101
HSALNG0088102
FNDC3B
LINC00355
LOC105373170
KRT8P18
CTDNEP1
SLC9A9-AS1
LOC100130331
ENSG00000257322
ENSG00000236501
RNU6-908P
ENSG00000250541
ENSG00000265994
ENSG00000207171
EEF1A1P20
RNU1-35P
ENSG00000235464
ENSG00000236389
RNU6-161P
MTND1P25
ENSG00000284676
ENSG00000287032
ENSG00000276627
HSALNG0076986
lnc-CRYAA-1
piR-55809-001
lnc-DDX60L-3
LOC102723733
ENSG00000270927
ENSG00000226973
LOC107986198
lnc-EDRF1-4
HSALNG0038430
HSALNG0029504
HSALNG0076987-001
HSALNG0076987-002
lnc-AIFM2-3
HSALNG0093093
piR-38051-198
piR-41195-080
piR-59297-626
AB372775
HSALNG0029512
HSALNG0093097
piR-31109-001

piR-57133-376
HSALNG0131408
ENSG00000254939
HSALNG0068856
POT1-AS1
PBX2P1
MAS1
LINC02629
RNU6-976P
lnc-TRIB1-7
ENSG00000268473
HSALNG0055801
ZNF300
ST13P12
MIR190B
CLGN
LINC02789
IGHG4
ARPC4
ENSG00000286231
PSAPL1
ENSG00000248537
ENSG00000250001
HNRNPA1P58
LOC101060035
HSALNG0040068
lnc-PPP1CC-3
LINC00888
LINC01090
ENSG00000274121
MIR378C
SLC23A4P
H2BC18
MIR4772
RAB24
HMCES
CPB2-AS1
LINC01029
HDHD5-AS1
LINC02534
LINC02392
SNORA63E
LINC01818
SNORA63D
ENSG00000243273
ENSG00000251152
ENSG00000249664
ENSG00000251186
ENSG00000260234
LINC02822
ENSG00000252040
ENSG00000253092
ENSG00000253354
ENSG00000272479
ENSG00000234481
ENSG00000241679
RPL35AP33
RNU6-655P

LGMNP1
ENSG00000248112
HNRNPA3P5
ENSG00000272823
ENSG00000259080
ENSG00000254171
ENSG00000251730
ENSG00000197462
ENSG00000234034
ENSG00000235503
LOC107987158
MRPL45P1
RPL23AP20
RNU6-1172P
lnc-PNPLA5-3
ENSG00000271682
ENSG00000233983
lnc-TSNAX-2
SDR42E1P5
ENSG00000288092
lnc-FAM122A-1
lnc-MRGPRG-1
lnc-RHOB-11
lnc-SLC46A3-1
lnc-WDR91-4
HSALNG0071703
lnc-MYL2-4
MN309223
ENSG00000275383
ENSG00000225906
ENSG00000231984
lnc-TRIB1-8
HSALNG0017419
ENSG00000287720
lnc-BICD1-6
HSALNG0023242
HSALNG0113292
HSALNG0113293
NONHSAG013110.2
lnc-SLC22A18-7
HSALNG0071704
hsa-miR-5096-076
lnc-KLHL24-6
piR-50443-100
piR-61101-044
ENSG00000280306
ENSG00000285529
ENSG00000258506
ENSG00000271078
HSALNG0011461
LOC105376433
LOC107986000
lnc-RASGRP1-1
HSALNG0032698
HSALNG0043115
HSALNG0043127
HSALNG0014027
HSALNG0010758

HSALNG0031159
HSALNG0032696
HSALNG0056551
HSALNG0061543
NONHSAG018421.2
piR-43105-110
piR-48799-012
RF00017-1289
piR-55036-040
lnc-MARC2-2
piR-31534-137
piR-39858-368
piR-43106-189
LOC107984123
LOC105377705
ENSG00000219736
ENSG00000229119
ENSG00000220748
LOC102724253
RF00026-272
HSALNG0105127-003
HSP90AA2P
DUSP14
ZC3H12A
TSKU
TRIM47
SLC25A48
DNAJC11
SNRNP40
YIPF6
RPL17-C18orf32
LINC00333
PDIA2
TXNDC5
PHLPP2
LOC109279247
PRMT3
FAM166B
CR1L
SAP18
IGKV2D-23
NMBR
SUCNR1
SGIP1
MIR103B2
MIR103B1
DNAJC10
HERC4
FAM174A
MIR3666
MIR4668
TM6SF1
