# Supplementary material for: Explore the Mechanism of Astragalus mongholicus Bunge against Nonalcoholic Fatty Liver Disease Based on Network Pharmacology and Experimental Verification
Source: Gastroenterol Res Pract. 2022 Apr 5;2022:4745042. doi: 10.1155/2022/4745042 (PMC9005278; doi:10.1155/2022/4745042)
Supplement: Supplementary Materials — Table S1: the bioactive components and corresponding targets of ABM. Table S2: information of NAFLD-related targets collected from the GeneCards database. Table S3: the common targets of ABM and NAFLD. Table S4: the 383 biological process terms. Table S5: the 40 cellular component terms. Table S6: the 63 molecular function terms. [file 4745042.f1.zip › 4745042.f1/Table S4.pdf]

Category	Term	Count	%	PValue	Genes	List Total	Pop Hits	Pop Total	Fold Enrichment	Bonferroni	Benjamini	FDR
GOTERM_BP_DIRECT	response to drug	24	24.4897959	1.18E-19	CDKN1A, JUN, HSP90AA1, TGFB1, CHUK, STAT1, PTEN, FOS, HTR2A, PTGS2, SLC6A3, RELA, SLC6A4, ICAM1, SOD1, IL6, IFNG, CCND1, MYC, CASP3, CYP1A1, BCL2, PPARG, DRD1	98	304	16792	13.52739	2.23E-16	2.23E-16	1.96E-16
GOTERM_BP_DIRECT	aging	18	18.3673469	5.92E-17	NQO1, JUN, TGFB1, VCAM1, PTEN, FOS, HTR2A, MPO, SLC6A3, RELA, SOD1, CASP9, COL3A1, IL6, CYP1A1, CCL2, AKT1, NFE2L2	98	165	16792	18.692393	2.10E-13	5.61E-14	4.93E-14
GOTERM_BP_DIRECT	positive regulation of transcription from RNA polymerase II promoter	33	33.6734694	2.08E-16	GSK3B, NR1I3, NR1I2, SERPINE1, AHR, ADRB2, ELK1, TNF, RELA, EGFR, IKBKB, PPP3CA, RXRA, MYC, AKT1, JUN, TGFB1, CHUK, STAT1, FOS, MAPK14, ESR1, SIRT1, IL1A, CXCL10, IL6, IFNG, IRF1, IL1B, PPARG, PPARA, TP53, NFE2L2	98	981	16792	5.7639643	4.21E-13	1.32E-13	1.16E-13
GOTERM_BP_DIRECT	positive regulation of nitric oxide biosynthetic process	12	12.244898	6.49E-16	IL6, HSP90AA1, IFNG, IL1B, INSR, AKT1, OPRM1, PTGS2, ESR1, TNF, EGFR, ICAM1	98	43	16792	47.81775	1.26E-12	3.08E-13	2.70E-13
GOTERM_BP_DIRECT	response to lipopolysaccharide	15	15.3061224	5.69E-13	JUN, VCAM1, CHUK, FOS, OPRM1, PTGS2, MPO, SELE, CASP9, CXCL10, THBD, CXCL11, CASP8, CASP3,	98	164	16792	15.671976	1.08E-09	2.16E-10	1.90E-10
GOTERM_BP_DIRECT	negative regulation of apoptotic process	21	21.4285714	1.20E-12	NQO1, GSK3B, CDKN1A, HSPA5, PTEN, HSPB1, MPO, SIRT1, MMP9, EGFR, RELA, IKBKB, IL6, MAPK8, MYC, CASP3, BCL2, BIRC5, AKT1, TP53, PPARD	98	455	16792	7.9083203	2.27E-09	3.78E-10	3.32E-10
GOTERM_BP_DIRECT	positive regulation of gene expression	17	17.3469388	2.00E-12	CRP, TGFB1, CAV1, NR1I2, MAPK14, TNF, SLC6A4, IL1A, IL6, MAPK8, IFNG, MYC, IL1B, LDLR, TP53, NFE2L2, PPARD	98	262	16792	11.117931	3.80E-09	4.97E-10	4.37E-10
GOTERM_BP_DIRECT	cellular response to lipopolysaccharide	13	13.2653061	2.10E-12	CXCL8, NOS2, SERPINE1, MAPK14, TNF, RELA, ICAM1, CXCL10, IL6, MAPK8, IFNG, CCL2, PPARD	98	113	16792	19.71248	3.98E-09	4.97E-10	4.37E-10
GOTERM_BP_DIRECT	inflammatory response	19	19.3877551	5.11E-12	CRP, TGFB1, CXCL8, CHUK, FOS, PTGS2, SELE, TNF, RELA, IKBKB, IL1A, CXCL10, CXCL11, IL6, IL1B, SPP1, CCL2, AKT1, NFE2L2	98	379	16792	8.5899521	9.68E-09	1.08E-09	9.45E-10
GOTERM_BP_DIRECT	cellular response to hypoxia	12	12.244898	7.86E-12	OPRD1, PTEN, BCL2, HMOX1, AKT1, SLC2A4, PTGS2, SIRT1, TP53, ICAM1, PPARD, NFE2L2	98	96	16792	21.418367	1.49E-08	1.49E-09	1.31E-09
GOTERM_BP_DIRECT	response to antibiotic	9	9.18367347	8.67E-12	CASP9, IL6, HSP90AA1, CASP8, CASP3, CYP1A1, CCL2, TP53, SOD1	98	32	16792	48.191327	1.64E-08	1.49E-09	1.31E-09
GOTERM_BP_DIRECT	cellular response to organic cyclic compound	10	10.2040816	4.86E-11	TGFB1, CASP8, STAT1, IL1B, CASP3, CYP1A1, CYP1B1, CCL2, AKT1, TNF	98	59	16792	29.041854	9.20E-08	7.67E-09	6.74E-09
GOTERM_BP_DIRECT	positive regulation of transcription, DNA-templated	20	20.4081633	9.67E-11	JUN, TGFB1, STAT1, INSR, NR1I2, AHR, FOS, ELK1, ESR1, TNF, RELA, IKBKB, IL6, MYC, IL1B, IRF1, PPARG, PPARA, TP53, PPARD	98	515	16792	6.65425	1.83E-07	1.41E-08	1.24E-08
GOTERM_BP_DIRECT	response to estradiol	11	11.2244898	1.13E-10	CASP9, NQO1, TGFB1, CASP8, CCND1, MYC, CASP3, PTEN, PTGS2, ESR1, SLC6A4	98	91	16792	20.712267	2.13E-07	1.52E-08	1.34E-08
GOTERM_BP_DIRECT	response to hypoxia	13	13.2653061	3.04E-10	TGFB1, VCAM1, NOS2, CAV1, MMP2, SLC6A4, DPP4, PLAU, CASP3, CYP1A1, CCL2, HMOX1, PPARA	98	172	16792	12.950641	5.76E-07	3.84E-08	3.38E-08
GOTERM_BP_DIRECT	response to ethanol	11	11.2244898	4.73E-10	NQO1, VCAM1, CASP8, CCND1, MYC, PTEN, CCL2, SLC2A4, SLC6A3, ICAM1, SOD1	98	105	16792	17.950632	8.96E-07	5.60E-08	4.92E-08
GOTERM_BP_DIRECT	cellular response to tumor necrosis factor	11	11.2244898	7.50E-10	IKBKB, IL6, VCAM1, CXCL8, CHUK, CCL2, SLC2A4, SIRT1, RELA, ICAM1, NFE2L2	98	110	16792	17.134694	1.42E-06	8.37E-08	7.35E-08
GOTERM_BP_DIRECT	positive regulation of angiogenesis	11	11.2244898	1.16E-09	IL1A, CXCL8, IL1B, SERPINE1, HSPB1, CYP1B1, HMOX1, PRKCA, SIRT1, F3, NFE2L2	98	115	16792	16.389707	2.21E-06	1.23E-07	1.08E-07
GOTERM_BP_DIRECT	response to cold	8	8.16326531	1.25E-09	CXCL10, IL6, HSP90AA1, CASP8, ADRB1, PPARG, FOS, ADRB2	98	36	16792	38.077098	2.38E-06	1.25E-07	1.10E-07
GOTERM_BP_DIRECT	response to toxic substance	10	10.2040816	1.41E-09	NQO1, CDKN1A, CHUK, PON1, BCL2, BAX, CYP1B1, FOS, AHR, SLC6A4	98	85	16792	20.158463	2.67E-06	1.33E-07	1.17E-07

GOTERM_BP_DIRECT	positive regulation of smooth muscle cell proliferation	9	9.18367347	1.85E-09	IL6, JUN, STAT1, MYC, HMOX1, AKT1, PTGS2, TNF, EGFR	98	60	16792	25.702041	3.50E-06	1.67E-07	1.47E-07
GOTERM_BP_DIRECT	embryo implantation	8	8.16326531	3.94E-09	RXRA, IL1B, MMP2, SPP1, PTGS2, MMP9, SOD1,	98	42	16792	32.637512	7.47E-06	3.39E-07	2.98E-07
GOTERM_BP_DIRECT	angiogenesis	13	13.2653061	5.91E-09	JUN, CXCL8, CAV1, MMP2, SERPINE1, PTEN, PRKCA, PTGS2, MAPK14, SIRT1, CYP1B1, CCL2, HMOX1	98	223	16792	9.988835	1.12E-05	4.87E-07	4.28E-07
GOTERM_BP_DIRECT	negative regulation of gene expression	11	11.2244898	6.46E-09	OPRD1, CDKN1A, TGFB1, IFNG, NOS2, AKT1, ESR1, SIRT1, TNF, XDH, LDLR	98	137	16792	13.757783	1.22E-05	5.10E-07	4.48E-07
GOTERM_BP_DIRECT	extrinsic apoptotic signaling pathway in absence of ligand	7	7.14285714	3.75E-08	CASP9, IL1A, GSK3B, IL1B, CASP3, BCL2, BAX	98	34	16792	35.277311	7.11E-05	2.84E-06	2.50E-06
GOTERM_BP_DIRECT	positive regulation of protein	10	10.2040816	4.96E-08	TGFB1, CCND1, IL1B, INSR, AKT1, F2, SIRT1, TNF, MMP9, EGFR	98	127	16792	13.491885	9.41E-05	3.62E-06	3.18E-06
GOTERM_BP_DIRECT	positive regulation of blood vessel endothelial cell	6	6.12244898	6.32E-08	TGFB1, HSPB1, AKT1, PRKCA, MAPK14, NFE2L2	98	19	16792	54.10956	1.20E-04	4.44E-06	3.90E-06
GOTERM_BP_DIRECT	positive regulation of ERK1 and ERK2 cascade	11	11.2244898	6.72E-08	IL6, JUN, TGFB1, PTEN, CCL2, PRKCA, HTR2A, OPRM1, TNF, EGFR, ICAM1	98	175	16792	10.770379	1.27E-04	4.55E-06	4.00E-06
GOTERM_BP_DIRECT	cellular response to drug	8	8.16326531	1.39E-07	PPP3CA, NOS2, MYC, IL1B, CCL2, TP53, EGFR, NFE2L2	98	69	16792	19.866312	2.63E-04	9.07E-06	7.98E-06
GOTERM_BP_DIRECT	apoptotic process	17	17.3469388	1.46E-07	STAT1, IGFBP3, PTEN, AHR, MAPK14, CASP9, IL1A, CASP8, IFNG, IL1B, CASP3, IRF1, BCL2, BAX, BIRC5, TP53, PPARD	98	567	16792	5.1373862	2.76E-04	9.21E-06	8.10E-06
GOTERM_BP_DIRECT	positive regulation of peptidyl-serine phosphorylation	8	8.16326531	1.54E-07	OPRD1, GSK3B, IL6, TGFB1, CAV1, BCL2, AKT1, TNF	98	70	16792	19.582507	2.91E-04	9.25E-06	8.13E-06
GOTERM_BP_DIRECT	positive regulation of apoptotic process	13	13.2653061	1.56E-07	TGFB1, IGFBP3, PTGS2, SIRT1, TNF, SOD1, IL6, MAPK8, BAX, CYP1B1, AKT1, HMOX1, TP53	98	300	16792	7.425034	2.96E-04	9.25E-06	8.13E-06
GOTERM_BP_DIRECT	cellular response to mechanical stimulus	8	8.16326531	1.70E-07	MAPK8, TGFB1, CASP8, IL1B, IRF1, AKT1, PTGS2, EGFR	98	71	16792	19.306697	3.21E-04	9.73E-06	8.56E-06
GOTERM_BP_DIRECT	negative regulation of cell proliferation	14	14.2857143	4.45E-07	CDKN1A, JUN, TGFB1, CXCL8, IGFBP3, PTEN, OPRM1, PTGS2, IL1A, IL6, IL1B, IRF1, CYP1B1, TP53	98	396	16792	6.0577201	8.43E-04	2.44E-05	2.15E-05
GOTERM_BP_DIRECT	positive regulation of cell proliferation	15	15.3061224	4.51E-07	TGFB1, INSR, PTEN, HTR2A, F2, SIRT1, EGFR, RELA, DPP4, CXCL10, IL6, IFNG, MYC, BCL2, BIRC5	98	466	16792	5.5154594	8.54E-04	2.44E-05	2.15E-05
GOTERM_BP_DIRECT	response to hydrogen peroxide	7	7.14285714	4.64E-07	JUN, STAT1, CASP3, BCL2, HMOX1, SIRT1, SOD1	98	51	16792	23.518207	8.79E-04	2.44E-05	2.15E-05
GOTERM_BP_DIRECT	positive regulation of reactive oxygen species metabolic	6	6.12244898	7.37E-07	CDKN1A, F2, MAPK14, TP53, XDH, NFE2L2	98	30	16792	34.269388	0.0013951	3.77E-05	3.32E-05
GOTERM_BP_DIRECT	response to amino acid	6	6.12244898	8.74E-07	IL6, CHUK, CASP3, CCL2, RELA, ICAM1	98	31	16792	33.163924	0.0016556	4.25E-05	3.74E-05
GOTERM_BP_DIRECT	response to gamma radiation	6	6.12244898	8.74E-07	CXCL10, MYC, BCL2, BAX, CCL2, TP53	98	31	16792	33.163924	0.0016556	4.25E-05	3.74E-05
GOTERM_BP_DIRECT	steroid hormone mediated signaling pathway	7	7.14285714	9.09E-07	RXRA, NR1I3, NR1I2, PPARG, PPARA, ESR1, PPARD	98	57	16792	21.042607	0.0017215	4.31E-05	3.79E-05
GOTERM_BP_DIRECT	positive regulation of vasoconstriction	6	6.12244898	1.03E-06	CAV1, AKT1, HTR2A, PTGS2, EGFR, ICAM1	98	32	16792	32.127551	0.001953	4.65E-05	4.09E-05
GOTERM_BP_DIRECT	lipopolysaccharide-mediated signaling pathway	6	6.12244898	1.03E-06	TGFB1, IL1B, CCL2, AKT1, MAPK14, TNF	98	32	16792	32.127551	0.001953	4.65E-05	4.09E-05
GOTERM_BP_DIRECT	protein kinase B signaling	6	6.12244898	1.21E-06	TGFB1, IL1B, PTEN, CCL2, AKT1, TNF	98	33	16792	31.153989	0.0022909	5.33E-05	4.69E-05

GOTERM_BP_DIRECT	positive regulation of sequence-specific DNA binding transcription factor	8	8.16326531	2.47E-06	PPP3CA, IL6, IL1B, PTEN, AKT1, PPARG, ESR1, TNF	98	105	16792	13.055005	0.0046762	1.07E-04	9.37E-05
GOTERM_BP_DIRECT	positive regulation of neuron apoptotic process	6	6.12244898	4.69E-06	CASP9, NQO1, JUN, CASP3, BAX, TP53	98	43	16792	23.908875	0.0088475	1.97E-04	1.74E-04
GOTERM_BP_DIRECT	cellular response to insulin stimulus	7	7.14285714	5.41E-06	STAT1, MYC, INSR, CCL2, AKT1, PPARG, SLC2A4	98	77	16792	15.576994	0.0102063	2.23E-04	1.96E-04
GOTERM_BP_DIRECT	response to cAMP	6	6.12244898	6.59E-06	THBD, JUN, STAT1, FOS, RELA, SLC6A3	98	46	16792	22.349601	0.0124055	2.66E-04	2.34E-04
GOTERM_BP_DIRECT	wound healing	7	7.14285714	6.77E-06	COL3A1, IL6, CASP3, OPRM1, PPARA, EGFR, PPARD	98	80	16792	14.992857	0.0127406	2.67E-04	2.35E-04
GOTERM_BP_DIRECT	response to X-ray	5	5.10204082	7.07E-06	THBD, CDKN1A, CCND1, CASP3, TP53	98	22	16792	38.942486	0.0133009	2.73E-04	2.40E-04
GOTERM_BP_DIRECT	intrinsic apoptotic signaling pathway in response to DNA damage	6	6.12244898	7.34E-06	CASP9, BCL2, BAX, HMOX1, SIRT1, TNF	98	47	16792	21.874077	0.0138093	2.78E-04	2.45E-04
GOTERM_BP_DIRECT	activation of cysteine-type endopeptidase activity involved in apoptotic process	7	7.14285714	8.38E-06	CASP8, CASP3, BAX, PPARG, TNF, F3, XDH	98	83	16792	14.450947	0.0157577	3.11E-04	2.74E-04
GOTERM_BP_DIRECT	positive regulation of NF-kappaB transcription factor activity	8	8.16326531	1.19E-05	IKBKB, IL6, TGFB1, CHUK, IL1B, TNF, RELA, ICAM1	98	133	16792	10.306583	0.0222155	4.23E-04	3.72E-04
GOTERM_BP_DIRECT	regulation of sequence-specific DNA binding transcription factor	5	5.10204082	1.21E-05	JUN, MAPK8, HMOX1, FOS, MAPK14	98	25	16792	34.269388	0.0225911	4.23E-04	3.72E-04
GOTERM_BP_DIRECT	cell aging	5	5.10204082	1.21E-05	BCL2, SIRT1, TP53, ICAM1, SOD1	98	25	16792	34.269388	0.0225911	4.23E-04	3.72E-04
GOTERM_BP_DIRECT	regulation of nitric-oxide synthase activity	5	5.10204082	1.42E-05	HSP90AA1, IL1B, CAV1, AKT1, EGFR	98	26	16792	32.951334	0.0265273	4.89E-04	4.30E-04
GOTERM_BP_DIRECT	positive regulation of fibroblast proliferation	6	6.12244898	1.47E-05	CDKN1A, JUN, TGFB1, MYC, ESR1, EGFR	98	54	16792	19.038549	0.0273864	4.96E-04	4.36E-04
GOTERM_BP_DIRECT	positive regulation of superoxide anion generation	4	4.08163265	1.53E-05	CRP, TGFB1, EGFR, SOD1	98	9	16792	76.154195	0.0285882	5.09E-04	4.47E-04
GOTERM_BP_DIRECT	positive regulation of release of sequestered calcium ion into	5	5.10204082	1.66E-05	CXCL10, CXCL11, BAX, DRD1, F2	98	27	16792	31.730915	0.0309333	5.33E-04	4.68E-04
GOTERM_BP_DIRECT	positive regulation of vascular endothelial growth factor production	5	5.10204082	1.66E-05	IL1A, TGFB1, IL1B, CYP1B1, PTGS2	98	27	16792	31.730915	0.0309333	5.33E-04	4.68E-04
GOTERM_BP_DIRECT	protein import into nucleus, translocation	5	5.10204082	1.93E-05	OPRD1, TGFB1, IFNG, AKT1, TNF	98	28	16792	30.597668	0.0358389	6.08E-04	5.35E-04
GOTERM_BP_DIRECT	positive regulation of chemokine biosynthetic process	4	4.08163265	2.18E-05	IFNG, IL1B, HMOX1, TNF	98	10	16792	68.538776	0.0404221	6.59E-04	5.79E-04
GOTERM_BP_DIRECT	cellular response to dexamethasone	5	5.10204082	2.22E-05	CASP9, IL6, TGFB1, CCL2, EGFR	98	29	16792	29.542576	0.0412735	6.59E-04	5.79E-04
GOTERM_BP_DIRECT	liver regeneration	5	5.10204082	2.22E-05	TGFB1, CCND1, MYC, HMOX1, EGFR	98	29	16792	29.542576	0.0412735	6.59E-04	5.79E-04
GOTERM_BP_DIRECT	negative regulation of smooth muscle cell proliferation	5	5.10204082	2.22E-05	IFNG, IGFBP3, HMOX1, PPARG, PPARD	98	29	16792	29.542576	0.0412735	6.59E-04	5.79E-04
GOTERM_BP_DIRECT	response to mechanical stimulus	6	6.12244898	2.27E-05	COL3A1, JUN, STAT1, CCL2, PPARG, MPO	98	59	16792	17.425112	0.0420582	6.61E-04	5.81E-04

GOTERM_BP_DIRECT	cellular response to ionizing radiation	5	5.10204082	2.92E-05	CDKN1A, TGFB1, MAPK14, SIRT1, TP53	98	31	16792	27.636603	0.0538433	8.39E-04	7.37E-04
GOTERM_BP_DIRECT	negative regulation of transcription, DNA-templated	13	13.2653061	2.99E-05	JUN, TGFB1, NR1I3, NR1I2, AHR, SIRT1, TNF, RELA, IRF1, BIRC5, PPARG, TP53, PPARD	98	499	16792	4.4639483	0.055097	8.46E-04	7.44E-04
GOTERM_BP_DIRECT	regulation of apoptotic process	9	9.18367347	3.23E-05	CASP9, TGFB1, CASP8, STAT1, IGFBP3, BAX, BIRC5, ESR1, TP53	98	213	16792	7.2400115	0.0593307	8.99E-04	7.91E-04
GOTERM_BP_DIRECT	response to estrogen	6	6.12244898	3.64E-05	HSP90AA1, CCND1, CAV1, HMOX1, PPARG, ESR1	98	65	16792	15.816641	0.0666556	1.00E-03	8.79E-04
GOTERM_BP_DIRECT	positive regulation of cAMP-mediated signaling	4	4.08163265	3.96E-05	CXCL10, CXCL11, ADRB1, OPRM1	98	12	16792	57.115646	0.07227	0.00105652	9.29E-04
GOTERM_BP_DIRECT	PERK-mediated unfolded protein response	4	4.08163265	3.96E-05	CXCL8, HSPA5, CCL2, NFE2L2	98	12	16792	57.115646	0.07227	0.00105652	9.29E-04
GOTERM_BP_DIRECT	positive regulation of endothelial cell proliferation	6	6.12244898	4.86E-05	JUN, CCL2, AKT1, PRKCA, SIRT1, F3	98	69	16792	14.899734	0.0880434	0.00128001	0.00112533
GOTERM_BP_DIRECT	nitric oxide biosynthetic process	4	4.08163265	5.12E-05	NQO1, NOS2, CYP1B1, AKT1	98	13	16792	52.722135	0.0925445	0.00133025	0.0011695
GOTERM_BP_DIRECT	apoptotic signaling pathway	6	6.12244898	5.58E-05	CASP8, CASP3, CAV1, BAX, PRKCA, PPARD	98	71	16792	14.480023	0.1003992	0.00141068	0.00124021
GOTERM_BP_DIRECT	cellular response to interleukin-1	6	6.12244898	5.58E-05	IL6, CXCL8, MYC, CCL2, RELA, ICAM1	98	71	16792	14.480023	0.1003992	0.00141068	0.00124021
GOTERM_BP_DIRECT	negative regulation of extrinsic apoptotic signaling pathway in absence of ligand	5	5.10204082	5.97E-05	IL1A, IL1B, BCL2, AKT1, TNF	98	37	16792	23.154992	0.1069677	0.00146921	0.00129166
GOTERM_BP_DIRECT	response to nicotine	5	5.10204082	5.97E-05	VCAM1, CASP3, BCL2, HMOX1, SLC6A3	98	37	16792	23.154992	0.1069677	0.00146921	0.00129166
GOTERM_BP_DIRECT	negative regulation of transcription from RNA polymerase II	15	15.3061224	6.46E-05	TGFB1, STAT1, CAV1, ESR1, SIRT1, TNF, RELA, RXRA, IFNG, CCND1, MYC, PPARG, PPARA, TP53, PPARD	98	720	16792	3.5697279	0.115166	0.00155791	0.00136964
GOTERM_BP_DIRECT	negative regulation of oxidative stress-induced intrinsic apoptotic signaling	4	4.08163265	6.49E-05	HSPB1, AKT1, SIRT1, NFE2L2	98	14	16792	48.956268	0.1158058	0.00155791	0.00136964
GOTERM_BP_DIRECT	response to nutrient	6	6.12244898	6.81E-05	NQO1, VCAM1, STAT1, PTEN, PPARG, SLC6A4	98	74	16792	13.892995	0.121141	0.00161408	0.00141903
GOTERM_BP_DIRECT	negative regulation of cell growth	7	7.14285714	7.16E-05	CDKN1A, TGFB1, BCL2, PPARG, SIRT1, TP53, PPARD	98	121	16792	9.9126328	0.1268215	0.0016742	0.00147189
GOTERM_BP_DIRECT	circadian rhythm	6	6.12244898	7.27E-05	GSK3B, JUN, NOS2, SERPINE1, EGFR, SLC6A4	98	75	16792	13.707755	0.1286631	0.00167953	0.00147657
GOTERM_BP_DIRECT	response to progesterone	5	5.10204082	7.37E-05	TGFB1, CAV1, CCL2, FOS, RELA	98	39	16792	21.967556	0.1303405	0.00168251	0.00147919
GOTERM_BP_DIRECT	leukocyte migration	7	7.14285714	7.49E-05	THBD, MMP1, CAV1, F2, SELE, MMP9, ICAM1	98	122	16792	9.8313817	0.1323538	0.00169007	0.00148584
GOTERM_BP_DIRECT	positive regulation of cell migration	8	8.16326531	9.47E-05	TGFB1, HSPA5, PLAU, INSR, PRKCA, DRD1, F3, EGFR	98	184	16792	7.4498669	0.1643397	0.00208626	0.00183414
GOTERM_BP_DIRECT	response to UV-A	3	3.06122449	9.87E-05	CCND1, AKT1, EGFR	98	3	16792	171.34694	0.1706038	0.00208626	0.00183414
GOTERM_BP_DIRECT	positive regulation of calcidiol 1-monooxygenase	3	3.06122449	9.87E-05	IFNG, IL1B, TNF	98	3	16792	171.34694	0.1706038	0.00208626	0.00183414
GOTERM_BP_DIRECT	ovarian follicle development	5	5.10204082	9.90E-05	MYC, BCL2, BAX, ICAM1, SOD1	98	42	16792	20.398445	0.171008	0.00208626	0.00183414
GOTERM_BP_DIRECT	extrinsic apoptotic signaling pathway	5	5.10204082	9.90E-05	TGFB1, CASP8, IFNG, BAX, TNF	98	42	16792	20.398445	0.171008	0.00208626	0.00183414
GOTERM_BP_DIRECT	response to muscle stretch	4	4.08163265	9.91E-05	JUN, FOS, MAPK14, RELA	98	16	16792	42.836735	0.1711966	0.00208626	0.00183414

GOTERM_BP_DIRECT	negative regulation of neuron apoptotic process	7	7.14285714	1.16E-04	JUN, BCL2, BAX, HMOX1, CCL2, BIRC5, SOD1	98	132	16792	9.0865801	0.1971304	0.00241264	0.00212109
GOTERM_BP_DIRECT	cellular response to	5	5.10204082	1.19E-04	CASP9, MYC, BAX, PTGS2, TP53	98	44	16792	19.471243	0.2018508	0.0024505	0.00215437
GOTERM_BP_DIRECT	positive regulation of protein kinase B signaling	6	6.12244898	1.25E-04	IL6, TGFB1, INSR, TNF, F3, EGFR	98	84	16792	12.239067	0.2105967	0.00254262	0.00223535
GOTERM_BP_DIRECT	endoplasmic reticulum unfolded protein response	5	5.10204082	1.30E-04	PTPN1, IFNG, CCND1, HSPA5, NFE2L2	98	45	16792	19.038549	0.2183644	0.00262075	0.00230405
GOTERM_BP_DIRECT	response to vitamin D	4	4.08163265	1.43E-04	CXCL10, TGFB1, SPP1, PTGS2	98	18	16792	38.077098	0.237636	0.00284843	0.00250421
GOTERM_BP_DIRECT	proteolysis	12	12.244898	1.44E-04	CASP9, DPP4, PRSS1, IGHG1, CASP8, PLAU, MMP1, CASP3, MMP2, F2, CTSD, MMP9	98	500	16792	4.1123265	0.2392642	0.00284843	0.00250421
GOTERM_BP_DIRECT	immune response	11	11.2244898	1.59E-04	OPRD1, IL1A, CXCL10, IL6, CXCL11, CXCL8, IFNG, CHUK, IL1B, CCL2, TNF	98	421	16792	4.4769984	0.2598415	0.00310172	0.0027269
GOTERM_BP_DIRECT	cell cycle arrest	7	7.14285714	1.66E-04	CDKN1A, TGFB1, CXCL8, IFNG, MYC, IRF1, TP53	98	141	16792	8.5065856	0.2703632	0.00320695	0.00281941
GOTERM_BP_DIRECT	response to heat	5	5.10204082	1.68E-04	IL6, HSP90AA1, CCL2, AKT1, SOD1	98	48	16792	17.848639	0.272044	0.00320695	0.00281941
GOTERM_BP_DIRECT	re-entry into mitotic cell cycle	3	3.06122449	1.97E-04	GSK3B, CCND1, MYC	98	4	16792	128.5102	0.3111434	0.00368503	0.00323972
GOTERM_BP_DIRECT	stress-induced premature senescence	3	3.06122449	1.97E-04	CDKN1A, MAPK14, SIRT1	98	4	16792	128.5102	0.3111434	0.00368503	0.00323972
GOTERM_BP_DIRECT	positive regulation of pri-miRNA transcription from RNA polymerase II	4	4.08163265	1.98E-04	JUN, TGFB1, FOS, RELA	98	20	16792	34.269388	0.3133364	0.00368503	0.00323972
GOTERM_BP_DIRECT	cellular response to DNA damage stimulus	8	8.16326531	2.02E-04	CASP9, CDKN1A, CCND1, MYC, BCL2, AKT1, SIRT1, TP53	98	208	16792	6.5902669	0.3186649	0.00372488	0.00327474
GOTERM_BP_DIRECT	response to cytokine	5	5.10204082	2.29E-04	COL3A1, JUN, STAT1, BCL2, FOS	98	52	16792	16.475667	0.352121	0.00411967	0.00362183
GOTERM_BP_DIRECT	response to food	4	4.08163265	2.30E-04	CYP1A1, AKT1, OPRM1, MPO	98	21	16792	32.637512	0.3538576	0.00411967	0.00362183
GOTERM_BP_DIRECT	response to immobilization stress	4	4.08163265	2.30E-04	TGFB1, CYP1A1, PPARG, FOS	98	21	16792	32.637512	0.3538576	0.00411967	0.00362183
GOTERM_BP_DIRECT	transcription initiation from RNA polymerase II promoter	7	7.14285714	2.50E-04	RXRA, NR1I3, NR1I2, PPARG, PPARA, ESR1, PPARD	98	152	16792	7.8909774	0.3774447	0.00442863	0.00389346
GOTERM_BP_DIRECT	cell proliferation	10	10.2040816	2.66E-04	IL1A, CHRM3, MYC, PTEN, BCL2, CYP1A1, AKT1, TP53, EGFR, PPARD	98	366	16792	4.6816103	0.3959717	0.00466729	0.00410327
GOTERM_BP_DIRECT	protein phosphorylation	11	11.2244898	3.01E-04	IKBKB, GSK3B, MAPK8, TGFB1, CCND1, CHUK, IGFBP3, CCL2, BIRC5, AKT1, PRKCA	98	456	16792	4.1333691	0.4352057	0.00519485	0.00456708
GOTERM_BP_DIRECT	negative regulation of myoblast	4	4.08163265	3.04E-04	CXCL10, TGFB1, TNF, PPARD	98	23	16792	29.799468	0.4382601	0.00519485	0.00456708
GOTERM_BP_DIRECT	cellular response to vascular endothelial growth factor stimulus	4	4.08163265	3.04E-04	VCAM1, HSPB1, AKT1, MAPK14	98	23	16792	29.799468	0.4382601	0.00519485	0.00456708
GOTERM_BP_DIRECT	positive regulation of fever generation	3	3.06122449	3.27E-04	IL1B, PTGS2, TNF	98	5	16792	102.80816	0.4614649	0.00538638	0.00473546
GOTERM_BP_DIRECT	humoral immune response	5	5.10204082	3.27E-04	IL6, IFNG, BCL2, CCL2, TNF	98	57	16792	15.030433	0.4618051	0.00538638	0.00473546
GOTERM_BP_DIRECT	negative regulation of protein binding	5	5.10204082	3.27E-04	GSK3B, MAPK8, CAV1, BAX, PPARA	98	57	16792	15.030433	0.4618051	0.00538638	0.00473546
GOTERM_BP_DIRECT	cellular response to hydrogen peroxide	5	5.10204082	3.27E-04	IL6, CYP1B1, SIRT1, RELA, NFE2L2	98	57	16792	15.030433	0.4618051	0.00538638	0.00473546
GOTERM_BP_DIRECT	positive regulation of I-kappaB kinase/NF-kappaB signaling	7	7.14285714	3.41E-04	IKBKB, IL1A, CASP8, CHUK, HMOX1, TNF, RELA	98	161	16792	7.4498669	0.4756931	0.00556524	0.00489271
GOTERM_BP_DIRECT	response to calcium	5	5.10204082	3.50E-04	PPP3CA, IL6, CCND1, CAV1, EGFR	98	58	16792	14.771288	0.4844426	0.00566146	0.00497731

GOTERM_BP_DIRECT	defense response to virus	7	7.14285714	3.88E-04	CXCL10, IL6, IFNG, STAT1, IRF1, BCL2, RELA	98	165	16792	7.2692641	0.5210371	0.0062372	0.00548347
GOTERM_BP_DIRECT	response to tumor necrosis factor	4	4.08163265	3.92E-04	CASP8, CASP3, PTGS2, SELE	98	25	16792	27.41551	0.524205	0.00624053	0.00548639
GOTERM_BP_DIRECT	response to virus	6	6.12244898	4.40E-04	IKBKB, IFNG, CHUK, CYP1A1, HSPB1, TNF	98	110	16792	9.3461967	0.5653619	0.00688478	0.00605279
GOTERM_BP_DIRECT	response to oxidative stress	6	6.12244898	4.40E-04	HMOX1, AKT1, PTGS2, MPO, SIRT1, EGFR	98	110	16792	9.3461967	0.5653619	0.00688478	0.00605279
GOTERM_BP_DIRECT	memory	5	5.10204082	4.52E-04	PTEN, DRD1, HTR2A, PTGS2, SLC6A4	98	62	16792	13.818302	0.5751042	0.00701408	0.00616647
GOTERM_BP_DIRECT	response to wounding	5	5.10204082	4.80E-04	TGFB1, MYC, CYP1A1, CCL2, F2	98	63	16792	13.598963	0.5974497	0.00728115	0.00640126
GOTERM_BP_DIRECT	positive regulation of interleukin-12 biosynthetic process	3	3.06122449	4.88E-04	IFNG, IRF1, RELA	98	6	16792	85.673469	0.6034414	0.00728115	0.00640126
GOTERM_BP_DIRECT	response to cobalt ion	3	3.06122449	4.88E-04	CASP9, CASP8, CASP3	98	6	16792	85.673469	0.6034414	0.00728115	0.00640126
GOTERM_BP_DIRECT	macrophage derived foam cell	3	3.06122449	4.88E-04	TGFB1, STAT1, PPARG	98	6	16792	85.673469	0.6034414	0.00728115	0.00640126
GOTERM_BP_DIRECT	negative regulation of production of miRNAs involved in gene silencing by miRNA	3	3.06122449	4.88E-04	PPP3CA, TGFB1, ESR1	98	6	16792	85.673469	0.6034414	0.00728115	0.00640126
GOTERM_BP_DIRECT	collagen catabolic process	5	5.10204082	5.10E-04	COL3A1, MMP1, MMP2, CTSD, MMP9	98	64	16792	13.38648	0.6195295	0.00754766	0.00663557
GOTERM_BP_DIRECT	response to glucocorticoid	5	5.10204082	5.41E-04	IL6, CASP3, BCL2, PTGS2, TNF	98	65	16792	13.180534	0.6412771	0.00794518	0.00698505
GOTERM_BP_DIRECT	response to radiation	4	4.08163265	5.51E-04	COL3A1, JUN, BCL2, OPRM1	98	28	16792	24.478134	0.6482533	0.00797371	0.00701013
GOTERM_BP_DIRECT	regulation of mitochondrial membrane potential	4	4.08163265	5.51E-04	OPRD1, BCL2, BAX, SOD1	98	28	16792	24.478134	0.6482533	0.00797371	0.00701013
GOTERM_BP_DIRECT	Fc-epsilon receptor signaling pathway	7	7.14285714	5.80E-04	IKBKB, PPP3CA, JUN, MAPK8, CHUK, FOS, RELA	98	178	16792	6.7383628	0.6672205	0.008333	0.007326
GOTERM_BP_DIRECT	glucose metabolic process	5	5.10204082	6.07E-04	MYC, AKT1, MAPK14, TNF, PPARD	98	67	16792	12.787085	0.6835224	0.00846875	0.00744535
GOTERM_BP_DIRECT	regulation of insulin secretion	5	5.10204082	6.07E-04	IFNG, NOS2, IL1B, PRKCA, TNF	98	67	16792	12.787085	0.6835224	0.00846875	0.00744535
GOTERM_BP_DIRECT	positive regulation of vasodilation	4	4.08163265	6.12E-04	NOS2, HMOX1, EGFR, PPARD	98	29	16792	23.634061	0.6866936	0.00846875	0.00744535
GOTERM_BP_DIRECT	cellular response to glucose starvation	4	4.08163265	6.12E-04	HSPA5, BCL2, TP53, NFE2L2	98	29	16792	23.634061	0.6866936	0.00846875	0.00744535
GOTERM_BP_DIRECT	endothelial cell migration	4	4.08163265	6.12E-04	DPP4, STAT1, PTEN, CYP1B1	98	29	16792	23.634061	0.6866936	0.00846875	0.00744535
GOTERM_BP_DIRECT	positive regulation of glucose import	4	4.08163265	6.77E-04	INSR, AKT1, MAPK14, NFE2L2	98	30	16792	22.846259	0.7231243	0.0091469	0.00804155
GOTERM_BP_DIRECT	positive regulation of protein insertion into mitochondrial membrane involved in apoptotic signaling pathway	4	4.08163265	6.77E-04	MAPK8, CASP8, BCL2, TP53	98	30	16792	22.846259	0.7231243	0.0091469	0.00804155
GOTERM_BP_DIRECT	regulation of tumor necrosis factor-mediated signaling pathway	4	4.08163265	6.77E-04	IKBKB, CASP8, CHUK, TNF	98	30	16792	22.846259	0.7231243	0.0091469	0.00804155
GOTERM_BP_DIRECT	regulation of establishment of endothelial barrier	3	3.06122449	6.81E-04	IKBKB, IL1B, TNF	98	7	16792	73.434402	0.7247711	0.0091469	0.00804155
GOTERM_BP_DIRECT	chemotaxis	6	6.12244898	7.05E-04	CXCL10, CXCL11, CXCL8, PLAU, CCL2, MAPK14	98	122	16792	8.4268986	0.7372855	0.00940997	0.00827283

GOTERM_BP_DIRECT	regulation of cell proliferation	7	7.14285714	7.11E-04	CXCL10, CXCL11, JUN, NOS2, PLAU, SIRT1, TNF	98	185	16792	6.4833977	0.7400316	0.0094176	0.00827954
GOTERM_BP_DIRECT	negative regulation of fibroblast proliferation	4	4.08163265	7.47E-04	IFNG, MYC, BAX, TP53	98	31	16792	22.109282	0.7572918	0.00976115	0.00858157
GOTERM_BP_DIRECT	response to amphetamine	4	4.08163265	7.47E-04	PPP3CA, DRD1, ICAM1, SOD1	98	31	16792	22.109282	0.7572918	0.00976115	0.00858157
GOTERM_BP_DIRECT	interferon-gamma-mediated signaling pathway	5	5.10204082	7.56E-04	VCAM1, IFNG, STAT1, IRF1, ICAM1	98	71	16792	12.066686	0.7614328	0.00981207	0.00862634
GOTERM_BP_DIRECT	peptidyl-serine phosphorylation	6	6.12244898	7.87E-04	GSK3B, MAPK8, BCL2, AKT1, PRKCA, MAPK14	98	125	16792	8.2246531	0.775081	0.01014577	0.00891971
GOTERM_BP_DIRECT	brown fat cell differentiation	4	4.08163265	8.21E-04	ADRB1, SLC2A4, ADRB2, PTGS2	98	32	16792	21.418367	0.7890022	0.01050857	0.00923867
GOTERM_BP_DIRECT	positive regulation of intrinsic apoptotic signaling pathway	4	4.08163265	8.99E-04	CAV1, BCL2, BAX, TP53	98	33	16792	20.769326	0.8181236	0.01112432	0.00978001
GOTERM_BP_DIRECT	negative regulation of extrinsic apoptotic signaling pathway via death domain	4	4.08163265	8.99E-04	CASP8, SERPINE1, HMOX1, ICAM1	98	33	16792	20.769326	0.8181236	0.01112432	0.00978001
GOTERM_BP_DIRECT	ectopic germ cell programmed cell death	3	3.06122449	9.04E-04	IL1A, IL1B, BAX	98	8	16792	64.255102	0.8198417	0.01112432	0.00978001
GOTERM_BP_DIRECT	activation of signaling protein activity involved in unfolded protein response	3	3.06122449	9.04E-04	PTPN1, HSPA5, BAX	98	8	16792	64.255102	0.8198417	0.01112432	0.00978001
GOTERM_BP_DIRECT	negative regulation of lipid storage	3	3.06122449	9.04E-04	CRP, IL6, TNF	98	8	16792	64.255102	0.8198417	0.01112432	0.00978001
GOTERM_BP_DIRECT	negative regulation of collagen biosynthetic process	3	3.06122449	9.04E-04	IL6, PPARG, PPARD	98	8	16792	64.255102	0.8198417	0.01112432	0.00978001
GOTERM_BP_DIRECT	cytokine-mediated signaling pathway	6	6.12244898	9.72E-04	IL1A, IL6, IL1B, CCL2, F3, RELA	98	131	16792	7.8479514	0.8416095	0.01186192	0.01042847
GOTERM_BP_DIRECT	extracellular matrix disassembly	5	5.10204082	9.76E-04	PRSS1, MMP1, MMP2, SPP1, MMP9	98	76	16792	11.272825	0.8429771	0.01186192	0.01042847
GOTERM_BP_DIRECT	cell surface receptor signaling pathway	8	8.16326531	0.00104981	CXCL10, CASP8, IFNG, CCL2, ADRB2, F2, MAPK14, EGFR	98	274	16792	5.0028303	0.863365	0.01267132	0.01114006
GOTERM_BP_DIRECT	xenobiotic metabolic process	5	5.10204082	0.00107618	NQO1, NR1I2, CYP1B1, AHR, CYP3A4	98	78	16792	10.983778	0.8700301	0.01290731	0.01134753
GOTERM_BP_DIRECT	activation of cysteine-type endopeptidase activity involved in apoptotic process by cytochrome c	3	3.06122449	0.00115796	CASP9, CASP3, BAX	98	9	16792	57.115646	0.8887081	0.01371455	0.01205722
GOTERM_BP_DIRECT	response to salt stress	3	3.06122449	0.00115796	HSP90AA1, BAX, TNF	98	9	16792	57.115646	0.8887081	0.01371455	0.01205722
GOTERM_BP_DIRECT	cellular response to	4	4.08163265	0.00125912	IL1A, CXCL10, CDKN1A, HMOX1	98	37	16792	18.523993	0.9081436	0.01482003	0.01302911
GOTERM_BP_DIRECT	positive regulation of peptidyl-tyrosine phosphorylation	5	5.10204082	0.0012966	IL6, TGFB1, HTR2A, TP53, ICAM1	98	82	16792	10.447984	0.9144505	0.01516708	0.01333422
GOTERM_BP_DIRECT	peptidyl-threonine phosphorylation	4	4.08163265	0.00136135	GSK3B, MAPK8, BCL2, AKT1	98	38	16792	18.03652	0.9243403	0.01573019	0.01382929
GOTERM_BP_DIRECT	intracellular receptor signaling pathway	4	4.08163265	0.00136135	NR1I2, AHR, PPARA, PPARD	98	38	16792	18.03652	0.9243403	0.01573019	0.01382929
GOTERM_BP_DIRECT	striated muscle cell differentiation	3	3.06122449	0.001442	CHUK, AKT1, MAPK14	98	10	16792	51.404082	0.9350778	0.01636284	0.01438548

GOTERM_BP_DIRECT	negative regulation of fibrinolysis	3	3.06122449	0.001442	THBD, SERPINE1, F2	98	10	16792	51.404082	0.9350778	0.01636284	0.01438548
GOTERM_BP_DIRECT	ER overload response	3	3.06122449	0.001442	GSK3B, HSPA5, TP53	98	10	16792	51.404082	0.9350778	0.01636284	0.01438548
GOTERM_BP_DIRECT	movement of cell or subcellular component	5	5.10204082	0.00154682	CXCL8, IFNG, HSPB1, MAPK14, PTGS2	98	86	16792	9.9620313	0.9467891	0.01741991	0.01531481
GOTERM_BP_DIRECT	activation of adenylate cyclase activity	4	4.08163265	0.00158112	ADRB1, PRKCA, DRD1, ADRB2	98	40	16792	17.134694	0.950143	0.01741991	0.01531481
GOTERM_BP_DIRECT	regulation of mitotic cell cycle	4	4.08163265	0.00158112	CDKN1A, MYC, BIRC5, SIRT1	98	40	16792	17.134694	0.950143	0.01741991	0.01531481
GOTERM_BP_DIRECT	negative regulation of neuron death	4	4.08163265	0.00158112	IL6, AKT1, PPARA, SIRT1	98	40	16792	17.134694	0.950143	0.01741991	0.01531481
GOTERM_BP_DIRECT	negative regulation of I-kappaB kinase/NF-kappaB signaling	4	4.08163265	0.00158112	CASP8, STAT1, ESR1, SIRT1	98	40	16792	17.134694	0.950143	0.01741991	0.01531481
GOTERM_BP_DIRECT	negative regulation of epithelial cell differentiation	3	3.06122449	0.00175582	IFNG, CCND1, CAV1	98	11	16792	46.730983	0.9642143	0.01923285	0.01690867
GOTERM_BP_DIRECT	lactation	4	4.08163265	0.00182191	CCND1, CAV1, XDH, SLC6A3	98	42	16792	16.318756	0.968434	0.01961663	0.01724607
GOTERM_BP_DIRECT	positive regulation of DNA replication	4	4.08163265	0.00182191	IL6, JUN, INSR, EGFR	98	42	16792	16.318756	0.968434	0.01961663	0.01724607
GOTERM_BP_DIRECT	negative regulation of fat cell differentiation	4	4.08163265	0.00182191	IL6, TGFB1, SIRT1, TNF	98	42	16792	16.318756	0.968434	0.01961663	0.01724607
GOTERM_BP_DIRECT	steroid metabolic process	4	4.08163265	0.00195041	NR1I2, CYP1A1, CYP1B1, CYP3A4	98	43	16792	15.93925	0.9752675	0.02088151	0.0183581
GOTERM_BP_DIRECT	transforming growth factor beta receptor signaling pathway	5	5.10204082	0.00198242	COL3A1, JUN, TGFB1, CCL2, FOS	98	92	16792	9.3123336	0.9767258	0.02110495	0.01855454
GOTERM_BP_DIRECT	removal of superoxide radicals	3	3.06122449	0.00209907	NQO1, MPO, SOD1	98	12	16792	42.836735	0.9813501	0.02222199	0.01953659
GOTERM_BP_DIRECT	positive regulation of interleukin-6	4	4.08163265	0.00222402	IL1A, IL6, IL1B, TNF	98	45	16792	15.230839	0.9852897	0.02341398	0.02058453
GOTERM_BP_DIRECT	G-protein coupled receptor signaling pathway, coupled to cyclic nucleotide second messenger	4	4.08163265	0.00236929	OPRD1, CCL2, DRD1, OPRM1	98	46	16792	14.899734	0.9888369	0.02480559	0.02180797
GOTERM_BP_DIRECT	cellular response to ATP	3	3.06122449	0.0024714	CCL2, PTGS2, SOD1	98	13	16792	39.541601	0.9908051	0.02517906	0.02213631
GOTERM_BP_DIRECT	response to arsenic-containing substance	3	3.06122449	0.0024714	CDKN1A, PTEN, CYP1A1	98	13	16792	39.541601	0.9908051	0.02517906	0.02213631
GOTERM_BP_DIRECT	negative regulation of macrophage derived foam cell	3	3.06122449	0.0024714	CRP, PPARG, PPARA	98	13	16792	39.541601	0.9908051	0.02517906	0.02213631
GOTERM_BP_DIRECT	leukocyte tethering or rolling	3	3.06122449	0.0024714	VCAM1, SELE, TNF	98	13	16792	39.541601	0.9908051	0.02517906	0.02213631
GOTERM_BP_DIRECT	prepulse inhibition	3	3.06122449	0.0024714	PTEN, DRD1, SLC6A3	98	13	16792	39.541601	0.9908051	0.02517906	0.02213631
GOTERM_BP_DIRECT	positive regulation of fat cell differentiation	4	4.08163265	0.00252032	AKT1, PPARG, HTR2A, PPARD	98	47	16792	14.582718	0.9916211	0.02540428	0.02233432
GOTERM_BP_DIRECT	cellular response to amino acid stimulus	4	4.08163265	0.00252032	COL3A1, MMP2, TNF, EGFR	98	47	16792	14.582718	0.9916211	0.02540428	0.02233432
GOTERM_BP_DIRECT	signal transduction	16	16.3265306	0.00262593	CHRM3, HSP90AA1, CXCL8, NR1I3, NR1I2, MAPK14, ESR1, EGFR, CXCL10, THBD, CXCL11, PLAU, IL1B, CCL2, AKT1, PPARG	98	1161	16792	2.3613704	0.9931443	0.02632875	0.02314707
GOTERM_BP_DIRECT	glucose homeostasis	5	5.10204082	0.00278477	IL6, INSR, AKT1, PPARG, SLC2A4	98	101	16792	8.4825217	0.9949304	0.02777216	0.02441605
GOTERM_BP_DIRECT	transcription from RNA polymerase II	10	10.2040816	0.00285513	JUN, MYC, IRF1, FOS, AHR, ELK1, ESR1, TP53, RELA, NFE2L2	98	513	16792	3.3400963	0.9955649	0.02777216	0.02441605

GOTERM_BP_DIRECT	positive regulation of glycolytic process	3	3.06122449	0.00287248	MYC, INSR, HTR2A	98	14	16792	36.717201	0.9957087	0.02777216	0.02441605
GOTERM_BP_DIRECT	cellular response to hepatocyte growth factor stimulus	3	3.06122449	0.00287248	GSK3B, IL6, RELA	98	14	16792	36.717201	0.9957087	0.02777216	0.02441605
GOTERM_BP_DIRECT	positive regulation of protein	3	3.06122449	0.00287248	MMP1, BAX, TP53	98	14	16792	36.717201	0.9957087	0.02777216	0.02441605
GOTERM_BP_DIRECT	positive regulation of blood coagulation	3	3.06122449	0.00287248	SERPINE1, F2, NFE2L2	98	14	16792	36.717201	0.9957087	0.02777216	0.02441605
GOTERM_BP_DIRECT	negative regulation of smooth muscle cell migration	3	3.06122449	0.00287248	IGFBP3, SERPINE1, PPARD	98	14	16792	36.717201	0.9957087	0.02777216	0.02441605
GOTERM_BP_DIRECT	regulation of I-kappaB kinase/NF-kappaB signaling	3	3.06122449	0.00330196	IL1B, HSPB1, TNF	98	15	16792	34.269388	0.9981032	0.03144328	0.02764354
GOTERM_BP_DIRECT	response to copper ion	3	3.06122449	0.00330196	IL1A, ICAM1, SOD1	98	15	16792	34.269388	0.9981032	0.03144328	0.02764354
GOTERM_BP_DIRECT	positive regulation of membrane protein ectodomain proteolysis	3	3.06122449	0.00330196	IFNG, IL1B, TNF	98	15	16792	34.269388	0.9981032	0.03144328	0.02764354
GOTERM_BP_DIRECT	activation of MAPK activity	5	5.10204082	0.00342859	IL1B, INSR, MAPK14, TNF, SOD1	98	107	16792	8.0068663	0.998509	0.03248584	0.02856011
GOTERM_BP_DIRECT	negative regulation of growth of symbiont in host	3	3.06122449	0.00375951	IFNG, MPO, TNF	98	16	16792	32.127551	0.9992054	0.03509496	0.03085393
GOTERM_BP_DIRECT	execution phase of apoptosis	3	3.06122449	0.00375951	CASP8, CASP3, AKT1	98	16	16792	32.127551	0.9992054	0.03509496	0.03085393
GOTERM_BP_DIRECT	positive regulation of monocyte chemotaxis	3	3.06122449	0.00375951	CXCL10, SERPINE1, CCL2	98	16	16792	32.127551	0.9992054	0.03509496	0.03085393
GOTERM_BP_DIRECT	MAPK cascade	7	7.14285714	0.00412262	TGFB1, MYC, IL1B, CAV1, CCL2, TNF, EGFR	98	262	16792	4.5779716	0.9996018	0.03829594	0.03366809
GOTERM_BP_DIRECT	heart development	6	6.12244898	0.00418034	COL3A1, TGFB1, PTEN, PPARG, PPARA, PPARD	98	183	16792	5.6179324	0.9996432	0.03864265	0.03397291
GOTERM_BP_DIRECT	cellular response to prostaglandin E stimulus	3	3.06122449	0.00424481	AKT1, PPARG, ACACA	98	17	16792	30.237695	0.9996844	0.03904809	0.03432935
GOTERM_BP_DIRECT	platelet activation	5	5.10204082	0.0044352	COL3A1, IL6, AKT1, PRKCA, F2	98	115	16792	7.4498669	0.9997803	0.04060247	0.03569589
GOTERM_BP_DIRECT	response to vitamin A	3	3.06122449	0.00475751	CYP1A1, PPARG, PPARD	98	18	16792	28.557823	0.9998811	0.04252586	0.03738685
GOTERM_BP_DIRECT	positive regulation of leukocyte chemotaxis	3	3.06122449	0.00475751	CXCL10, IL6, CXCL11	98	18	16792	28.557823	0.9998811	0.04252586	0.03738685
GOTERM_BP_DIRECT	macrophage differentiation	3	3.06122449	0.00475751	CASP8, SIRT1, MMP9	98	18	16792	28.557823	0.9998811	0.04252586	0.03738685
GOTERM_BP_DIRECT	response to iron ion	3	3.06122449	0.00475751	CCND1, BCL2, SLC6A3	98	18	16792	28.557823	0.9998811	0.04252586	0.03738685
GOTERM_BP_DIRECT	response to corticosterone	3	3.06122449	0.00475751	CDKN1A, CCND1, FOS	98	18	16792	28.557823	0.9998811	0.04252586	0.03738685
GOTERM_BP_DIRECT	positive regulation of MAP kinase activity	4	4.08163265	0.00480701	TGFB1, HTR2A, TNF, EGFR	98	59	16792	11.616742	0.9998918	0.04276655	0.03759846
GOTERM_BP_DIRECT	positive regulation of epithelial cell proliferation	4	4.08163265	0.00503905	IL6, TGFB1, MYC, EGFR	98	60	16792	11.423129	0.9999304	0.04441392	0.03904675
GOTERM_BP_DIRECT	positive regulation of T cell proliferation	4	4.08163265	0.00503905	IL6, VCAM1, IFNG, IL1B	98	60	16792	11.423129	0.9999304	0.04441392	0.03904675
GOTERM_BP_DIRECT	negative regulation of protein	4	4.08163265	0.00527774	TGFB1, IGFBP3, PTEN, XDH	98	61	16792	11.235865	0.9999558	0.04542254	0.03993348
GOTERM_BP_DIRECT	response to stress	4	4.08163265	0.00527774	HSP90AA1, MAPK8, AHSA1, EGFR	98	61	16792	11.235865	0.9999558	0.04542254	0.03993348
GOTERM_BP_DIRECT	positive regulation of protein export from nucleus	3	3.06122449	0.0052973	GSK3B, IL1B, TP53	98	19	16792	27.05478	0.9999575	0.04542254	0.03993348

GOTERM_BP_DIRECT	temperature homeostasis	3	3.06122449	0.0052973	ADRB1, DRD1, HTR2A	98	19	16792	27.05478	0.9999575	0.04542254	0.03993348
GOTERM_BP_DIRECT	positive regulation of synaptic transmission, glutamatergic	3	3.06122449	0.0052973	DRD1, PTGS2, EGFR	98	19	16792	27.05478	0.9999575	0.04542254	0.03993348
GOTERM_BP_DIRECT	positive regulation of osteoclast differentiation	3	3.06122449	0.0052973	IFNG, FOS, TNF	98	19	16792	27.05478	0.9999575	0.04542254	0.03993348
GOTERM_BP_DIRECT	extracellular matrix organization	6	6.12244898	0.00557755	COL3A1, VCAM1, SERPINE1, SPP1, TNF, ICAM1	98	196	16792	5.2453145	0.9999751	0.04761017	0.04185675
GOTERM_BP_DIRECT	regulation of inflammatory response	4	4.08163265	0.00577529	PTGS2, SELE, ESR1, RELA	98	63	16792	10.879171	0.9999829	0.04907704	0.04314636
GOTERM_BP_DIRECT	decidualization	3	3.06122449	0.00586385	SPP1, PTGS2, PPARD	98	20	16792	25.702041	0.9999855	0.04960711	0.04361237
GOTERM_BP_DIRECT	negative regulation of transforming growth factor beta receptor signaling pathway	4	4.08163265	0.00603425	TGFB1, HSPA5, CAV1, SIRT1	98	64	16792	10.709184	0.9999896	0.0508218	0.04468028
GOTERM_BP_DIRECT	regulation of blood pressure	4	4.08163265	0.00630006	HMOX1, PPARG, PTGS2, SOD1	98	65	16792	10.544427	0.9999937	0.05282573	0.04644204
GOTERM_BP_DIRECT	positive regulation of NF-kappaB import into nucleus	3	3.06122449	0.00645684	IL1B, PTGS2, TNF	98	21	16792	24.478134	0.9999953	0.05366539	0.04718024
GOTERM_BP_DIRECT	fibrinolysis	3	3.06122449	0.00645684	PLAU, SERPINE1, F2	98	21	16792	24.478134	0.9999953	0.05366539	0.04718024
GOTERM_BP_DIRECT	neutrophil chemotaxis	4	4.08163265	0.00657277	CXCL8, IFNG, IL1B, CCL2	98	66	16792	10.384663	0.9999963	0.05415388	0.04760969
GOTERM_BP_DIRECT	phospholipase C-activating G-protein coupled receptor signaling pathway	4	4.08163265	0.00657277	OPRD1, HTR2A, OPRM1, ESR1	98	66	16792	10.384663	0.9999963	0.05415388	0.04760969
GOTERM_BP_DIRECT	response to insulin	4	4.08163265	0.00685242	IL6, PPARA, SIRT1, RELA	98	67	16792	10.229668	0.9999978	0.05621353	0.04942045
GOTERM_BP_DIRECT	positive regulation of protein complex assembly	3	3.06122449	0.00707595	GSK3B, TGFB1, TNF	98	22	16792	23.365492	0.9999986	0.05779711	0.05081265
GOTERM_BP_DIRECT	response to glucose	4	4.08163265	0.00713905	TGFB1, CASP3, PTEN, PPARD	98	68	16792	10.079232	0.9999987	0.05781408	0.05082758
GOTERM_BP_DIRECT	cholesterol metabolic process	4	4.08163265	0.00713905	RXRA, PON1, LDLR, PPARD	98	68	16792	10.079232	0.9999987	0.05781408	0.05082758
GOTERM_BP_DIRECT	oxidation-reduction process	10	10.2040816	0.00721083	NQO1, VCAM1, NOS2, CYP1A1, CYP1B1, CYP3A4, PTGS2, MPO, XDH, SOD1	98	592	16792	2.894374	0.9999989	0.05814694	0.05112021
GOTERM_BP_DIRECT	positive regulation of collagen biosynthetic process	3	3.06122449	0.00772087	TGFB1, CCL2, F2	98	23	16792	22.349601	0.9999996	0.06106188	0.0536829
GOTERM_BP_DIRECT	release of cytochrome c from mitochondria	3	3.06122449	0.00772087	JUN, BCL2, BAX	98	23	16792	22.349601	0.9999996	0.06106188	0.0536829
GOTERM_BP_DIRECT	response to bacterium	3	3.06122449	0.00772087	NOS2, CAV1, CCL2	98	23	16792	22.349601	0.9999996	0.06106188	0.0536829
GOTERM_BP_DIRECT	Ras protein signal transduction	4	4.08163265	0.00773343	CDKN1A, JUN, MAPK14, TP53	98	70	16792	9.7912536	0.9999996	0.06106188	0.0536829
GOTERM_BP_DIRECT	cellular response to retinoic acid	4	4.08163265	0.00773343	MYC, CCL2, PPARG, SLC6A4	98	70	16792	9.7912536	0.9999996	0.06106188	0.0536829
GOTERM_BP_DIRECT	chemokine-mediated signaling pathway	4	4.08163265	0.00804126	CXCL10, CXCL11, CXCL8, CCL2	98	71	16792	9.6533487	0.9999998	0.06322899	0.05558813
GOTERM_BP_DIRECT	vascular endothelial growth factor receptor signaling pathway	4	4.08163265	0.00835623	HSP90AA1, HSPB1, CCL2, MAPK14	98	72	16792	9.5192744	0.9999999	0.06543412	0.05752678
GOTERM_BP_DIRECT	positive regulation of inflammatory response	4	4.08163265	0.00867838	SERPINE1, CCL2, LDLR, EGFR	98	73	16792	9.3888734	0.9999999	0.06767708	0.05949869
GOTERM_BP_DIRECT	leukocyte cell-cell adhesion	3	3.06122449	0.00908689	VCAM1, SELE, ICAM1	98	25	16792	20.561633	1	0.06999858	0.06153965

GOTERM_BP_DIRECT	negative regulation of G1/S transition of mitotic cell cycle	3	3.06122449	0.00908689	CDKN1A, PTEN, BCL2	98	25	16792	20.561633	1	0.06999858	0.06153965
GOTERM_BP_DIRECT	nucleotide-binding oligomerization domain containing signaling pathway	3	3.06122449	0.00908689	IKBKB, CASP8, CHUK	98	25	16792	20.561633	1	0.06999858	0.06153965
GOTERM_BP_DIRECT	activation of phospholipase C activity	3	3.06122449	0.00980737	HTR2A, SELE, EGFR	98	26	16792	19.770801	1	0.07463838	0.06561876
GOTERM_BP_DIRECT	positive regulation of interleukin-8	3	3.06122449	0.00980737	IL1B, SERPINE1, TNF	98	26	16792	19.770801	1	0.07463838	0.06561876
GOTERM_BP_DIRECT	positive regulation of mitotic nuclear	3	3.06122449	0.00980737	IL1A, IL1B, INSR	98	26	16792	19.770801	1	0.07463838	0.06561876
GOTERM_BP_DIRECT	T cell receptor signaling pathway	5	5.10204082	0.01066292	IKBKB, IFNG, CHUK, PTEN, RELA	98	148	16792	5.7887479	1	0.08082494	0.0710577
GOTERM_BP_DIRECT	viral entry into host	4	4.08163265	0.01113714	DPP4, HTR2A, LDLR, ICAM1	98	80	16792	8.5673469	1	0.08332282	0.07325373
GOTERM_BP_DIRECT	ossification	4	4.08163265	0.01113714	BCL2, SPP1, MMP9, EGFR	98	80	16792	8.5673469	1	0.08332282	0.07325373
GOTERM_BP_DIRECT	TRIF-dependent toll-like receptor signaling pathway	3	3.06122449	0.01132174	IKBKB, CASP8, CHUK	98	28	16792	18.358601	1	0.08332282	0.07325373
GOTERM_BP_DIRECT	negative regulation of endothelial cell apoptotic process	3	3.06122449	0.01132174	SERPINE1, ICAM1, NFE2L2	98	28	16792	18.358601	1	0.08332282	0.07325373
GOTERM_BP_DIRECT	positive regulation of release of cytochrome c from mitochondria	3	3.06122449	0.01132174	BAX, TP53, MMP9	98	28	16792	18.358601	1	0.08332282	0.07325373
GOTERM_BP_DIRECT	positive regulation of catalytic activity	4	4.08163265	0.01151784	CAV1, IGFBP3, BCL2, SOD1	98	81	16792	8.4615772	1	0.08332282	0.07325373
GOTERM_BP_DIRECT	positive regulation of MAPK cascade	4	4.08163265	0.01151784	IL6, INSR, IGFBP3, ADRB2	98	81	16792	8.4615772	1	0.08332282	0.07325373
GOTERM_BP_DIRECT	positive regulation of translational initiation by iron	2	2.04081633	0.01152009	RXRA, TNF	98	2	16792	171.34694	1	0.08332282	0.07325373
GOTERM_BP_DIRECT	negative regulation of synaptic transmission, dopaminergic	2	2.04081633	0.01152009	PTGS2, SLC6A4	98	2	16792	171.34694	1	0.08332282	0.07325373
GOTERM_BP_DIRECT	positive regulation of vitamin D biosynthetic process	2	2.04081633	0.01152009	IFNG, TNF	98	2	16792	171.34694	1	0.08332282	0.07325373
GOTERM_BP_DIRECT	regulation of nitrogen utilization	2	2.04081633	0.01152009	BCL2, BAX	98	2	16792	171.34694	1	0.08332282	0.07325373
GOTERM_BP_DIRECT	positive regulation of leukocyte mediated cytotoxicity	2	2.04081633	0.01152009	NOS2, CCL2	98	2	16792	171.34694	1	0.08332282	0.07325373
GOTERM_BP_DIRECT	behavioral fear response	3	3.06122449	0.01211504	DPP4, BCL2, DRD1	98	29	16792	17.725545	1	0.08663393	0.07616471
GOTERM_BP_DIRECT	negative regulation of insulin receptor signaling pathway	3	3.06122449	0.01211504	PTPN1, IL1B, RELA	98	29	16792	17.725545	1	0.08663393	0.07616471
GOTERM_BP_DIRECT	negative regulation of endothelial cell proliferation	3	3.06122449	0.01211504	STAT1, CAV1, XDH	98	29	16792	17.725545	1	0.08663393	0.07616471
GOTERM_BP_DIRECT	canonical Wnt signaling pathway	4	4.08163265	0.01230161	GSK3B, CCND1, MYC, PTEN	98	83	16792	8.2576838	1	0.08763738	0.0770469
GOTERM_BP_DIRECT	locomotory behavior	4	4.08163265	0.01270471	PTEN, OPRM1, SLC6A3, SOD1	98	84	16792	8.159378	1	0.08983366	0.07897777

GOTERM_BP_DIRECT	positive regulation of cell growth	4	4.08163265	0.01270471	BCL2, AKT1, F2, EGFR	98	84	16792	8.159378	1	0.08983366	0.07897777
GOTERM_BP_DIRECT	intrinsic apoptotic signaling pathway	3	3.06122449	0.01293201	CDKN1A, BAX, TP53	98	30	16792	17.134694	1	0.09076353	0.07979527
GOTERM_BP_DIRECT	cellular response to fibroblast growth factor stimulus	3	3.06122449	0.01293201	CXCL8, MYC, CCL2	98	30	16792	17.134694	1	0.09076353	0.07979527
GOTERM_BP_DIRECT	lipid metabolic process	5	5.10204082	0.01301624	PPARG, CYP3A4, PPARA, LDLR, PPARD	98	157	16792	5.4569089	1	0.09101759	0.08001863
GOTERM_BP_DIRECT	intrinsic apoptotic signaling pathway in response to DNA damage by p53 class mediator	3	3.06122449	0.01377236	CDKN1A, SIRT1, TP53	98	31	16792	16.581962	1	0.09559934	0.0840467
GOTERM_BP_DIRECT	cellular response to estradiol stimulus	3	3.06122449	0.01377236	IL6, ESR1, EGFR	98	31	16792	16.581962	1	0.09559934	0.0840467
GOTERM_BP_DIRECT	negative regulation of protein catabolic process	3	3.06122449	0.0146358	NOS2, RELA, EGFR	98	32	16792	16.063776	1	0.10085398	0.08866635
GOTERM_BP_DIRECT	response to cocaine	3	3.06122449	0.0146358	HSP90AA1, OPRM1, SLC6A3	98	32	16792	16.063776	1	0.10085398	0.08866635
GOTERM_BP_DIRECT	female pregnancy	4	4.08163265	0.01483322	THBD, TGFB1, BCL2, FOS	98	89	16792	7.700986	1	0.10184404	0.08953677
GOTERM_BP_DIRECT	intrinsic apoptotic signaling pathway in response to endoplasmic reticulum stress	3	3.06122449	0.01552204	GSK3B, BCL2, BAX	98	33	16792	15.576994	1	0.10505098	0.09235617
GOTERM_BP_DIRECT	response to interleukin-1	3	3.06122449	0.01552204	PRKCA, SELE, RELA	98	33	16792	15.576994	1	0.10505098	0.09235617
GOTERM_BP_DIRECT	glucose transport	3	3.06122449	0.01552204	AKT1, SLC2A4, PPARD	98	33	16792	15.576994	1	0.10505098	0.09235617
GOTERM_BP_DIRECT	cellular response to epidermal growth factor stimulus	3	3.06122449	0.01552204	MYC, AKT1, EGFR	98	33	16792	15.576994	1	0.10505098	0.09235617
GOTERM_BP_DIRECT	positive regulation of cell cycle	3	3.06122449	0.0164308	CCND1, MYC, SLC6A4	98	34	16792	15.118848	1	0.11080559	0.09741536
GOTERM_BP_DIRECT	cellular calcium ion homeostasis	4	4.08163265	0.01667264	TGFB1, CAV1, CCL2, HTR2A	98	93	16792	7.3697608	1	0.11106239	0.09764113
GOTERM_BP_DIRECT	response to cobalamin	2	2.04081633	0.01723079	RELA, EGFR	98	3	16792	114.23129	1	0.11106239	0.09764113
GOTERM_BP_DIRECT	vasodilation by norepinephrine-epinephrine involved in regulation of systemic arterial blood	2	2.04081633	0.01723079	ADRB1, ADRB2	98	3	16792	114.23129	1	0.11106239	0.09764113
GOTERM_BP_DIRECT	negative regulation of helicase activity	2	2.04081633	0.01723079	SIRT1, TP53	98	3	16792	114.23129	1	0.11106239	0.09764113
GOTERM_BP_DIRECT	negative regulation of receptor biosynthetic process	2	2.04081633	0.01723079	PPARG, PPARA	98	3	16792	114.23129	1	0.11106239	0.09764113
GOTERM_BP_DIRECT	heat generation	2	2.04081633	0.01723079	ADRB1, ADRB2	98	3	16792	114.23129	1	0.11106239	0.09764113
GOTERM_BP_DIRECT	positive regulation of killing of cells of other organism	2	2.04081633	0.01723079	IFNG, NOS2	98	3	16792	114.23129	1	0.11106239	0.09764113
GOTERM_BP_DIRECT	positive regulation of cyclase activity	2	2.04081633	0.01723079	MAPK8, MAPK14	98	3	16792	114.23129	1	0.11106239	0.09764113
GOTERM_BP_DIRECT	neutrophil apoptotic process	2	2.04081633	0.01723079	IL6, IFNG	98	3	16792	114.23129	1	0.11106239	0.09764113
GOTERM_BP_DIRECT	cellular response to prolactin	2	2.04081633	0.01723079	IL6, MYC	98	3	16792	114.23129	1	0.11106239	0.09764113

GOTERM_BP_DIRECT	fever generation	2	2.04081633	0.01723079	IL1A, IL1B	98	3	16792	114.23129	1	0.11106239	0.09764113
GOTERM_BP_DIRECT	transformation of host cell by virus	2	2.04081633	0.01723079	MYC, INSR	98	3	16792	114.23129	1	0.11106239	0.09764113
GOTERM_BP_DIRECT	positive regulation of cellular extravasation	2	2.04081633	0.01723079	CCL2, ICAM1	98	3	16792	114.23129	1	0.11106239	0.09764113
GOTERM_BP_DIRECT	digestive tract development	3	3.06122449	0.01736179	COL3A1, TGFB1, CYP1A1	98	35	16792	14.68688	1	0.11115065	0.09771873
GOTERM_BP_DIRECT	placenta development	3	3.06122449	0.01736179	PPARG, MAPK14, SOD1	98	35	16792	14.68688	1	0.11115065	0.09771873
GOTERM_BP_DIRECT	negative regulation of cell migration	4	4.08163265	0.01763819	SERPINE1, PTEN, BCL2, CYP1B1	98	95	16792	7.2146079	1	0.11253995	0.09894013
GOTERM_BP_DIRECT	learning or memory	3	3.06122449	0.01831473	CASP3, PTEN, EGFR	98	36	16792	14.278912	1	0.11646446	0.10239039
GOTERM_BP_DIRECT	negative regulation of protein kinase B signaling	3	3.06122449	0.01928933	PTEN, SIRT1, XDH	98	37	16792	13.892995	1	0.1222518	0.10747836
GOTERM_BP_DIRECT	regulation of gene expression	4	4.08163265	0.02018633	MYC, BCL2, AHR, F2	98	100	16792	6.8538776	1	0.12728711	0.11190518
GOTERM_BP_DIRECT	ERBB2 signaling pathway	3	3.06122449	0.02028533	HSP90AA1, AKT1, EGFR	98	38	16792	13.52739	1	0.12728711	0.11190518
GOTERM_BP_DIRECT	extrinsic apoptotic signaling pathway via death domain	3	3.06122449	0.02028533	BCL2, BAX, TNF	98	38	16792	13.52739	1	0.12728711	0.11190518
GOTERM_BP_DIRECT	acute-phase response	3	3.06122449	0.02130245	CRP, IL6, F2	98	39	16792	13.180534	1	0.1332282	0.11712833
GOTERM_BP_DIRECT	blood coagulation	5	5.10204082	0.0219729	THBD, PLAU, IRF1, F2, F3	98	184	16792	4.6561668	1	0.1369692	0.12041725
GOTERM_BP_DIRECT	positive regulation of cysteine-type endopeptidase activity involved in apoptotic process	3	3.06122449	0.02234042	MYC, SIRT1, TNF	98	40	16792	12.85102	1	0.1378166	0.12116225
GOTERM_BP_DIRECT	sequestering of triglyceride	2	2.04081633	0.02290883	IL1B, TNF	98	4	16792	85.673469	1	0.1378166	0.12116225
GOTERM_BP_DIRECT	regulation of vascular endothelial growth factor production	2	2.04081633	0.02290883	IL6, CCL2	98	4	16792	85.673469	1	0.1378166	0.12116225
GOTERM_BP_DIRECT	connective tissue replacement involved in inflammatory response wound	2	2.04081633	0.02290883	IL1A, TGFB1	98	4	16792	85.673469	1	0.1378166	0.12116225
GOTERM_BP_DIRECT	negative regulation of smooth muscle contraction	2	2.04081633	0.02290883	ADRB2, PTGS2	98	4	16792	85.673469	1	0.1378166	0.12116225
GOTERM_BP_DIRECT	positive regulation of mononuclear cell migration	2	2.04081633	0.02290883	TGFB1, TNF	98	4	16792	85.673469	1	0.1378166	0.12116225
GOTERM_BP_DIRECT	positive regulation of cell adhesion molecule production	2	2.04081633	0.02290883	IL1B, CAV1	98	4	16792	85.673469	1	0.1378166	0.12116225
GOTERM_BP_DIRECT	negative regulation of extracellular matrix disassembly	2	2.04081633	0.02290883	DPP4, TGFB1	98	4	16792	85.673469	1	0.1378166	0.12116225
GOTERM_BP_DIRECT	regulation of cytokine production involved in inflammatory response	2	2.04081633	0.02290883	NOS2, MAPK14	98	4	16792	85.673469	1	0.1378166	0.12116225
GOTERM_BP_DIRECT	negative regulation of bicellular tight junction assembly	2	2.04081633	0.02290883	IKBKB, TNF	98	4	16792	85.673469	1	0.1378166	0.12116225

GOTERM_BP_DIRECT	regulation of protein import into nucleus, translocation	2	2.04081633	0.02290883	CDKN1A, SIRT1	98	4	16792	85.673469	1	0.1378166	0.12116225
GOTERM_BP_DIRECT	cellular iron ion homeostasis	3	3.06122449	0.02669539	MYC, HMOX1, SOD1	98	44	16792	11.682746	1	0.16008785	0.14074214
GOTERM_BP_DIRECT	regulation of entry of bacterium into host	2	2.04081633	0.0285544	CXCL8, CAV1	98	5	16792	68.538776	1	0.1680453	0.14773798
GOTERM_BP_DIRECT	glial cell apoptotic process	2	2.04081633	0.0285544	CASP9, CASP3	98	5	16792	68.538776	1	0.1680453	0.14773798
GOTERM_BP_DIRECT	membrane to membrane docking	2	2.04081633	0.0285544	VCAM1, ICAM1	98	5	16792	68.538776	1	0.1680453	0.14773798
GOTERM_BP_DIRECT	negative regulation of myosin-light-chain-phosphatase activity	2	2.04081633	0.0285544	IKBKB, TNF	98	5	16792	68.538776	1	0.1680453	0.14773798
GOTERM_BP_DIRECT	chemical synaptic transmission, postsynaptic	2	2.04081633	0.0285544	GSK3B, AKT1	98	5	16792	68.538776	1	0.1680453	0.14773798
GOTERM_BP_DIRECT	negative regulation of sequestering of triglyceride	2	2.04081633	0.0285544	PPARG, PPARA	98	5	16792	68.538776	1	0.1680453	0.14773798
GOTERM_BP_DIRECT	intracellular signal transduction	7	7.14285714	0.02929791	GSK3B, CXCL8, HSPB1, HMOX1, AKT1, PRKCA, MAPK14	98	403	16792	2.9762496	1	0.17188711	0.15111553
GOTERM_BP_DIRECT	T cell activation	3	3.06122449	0.03016754	DPP4, PPP3CA, CASP8	98	47	16792	10.937039	1	0.17536037	0.15416907
GOTERM_BP_DIRECT	positive regulation of cell division	3	3.06122449	0.03016754	IL1A, TGFB1, IL1B	98	47	16792	10.937039	1	0.17536037	0.15416907
GOTERM_BP_DIRECT	organ regeneration	3	3.06122449	0.03016754	CDKN1A, CCL2, PPARG	98	47	16792	10.937039	1	0.17536037	0.15416907
GOTERM_BP_DIRECT	cellular protein metabolic process	4	4.08163265	0.03095271	MMP1, MMP2, IGFBP3, F2	98	118	16792	5.8083708	1	0.17937429	0.15769792
GOTERM_BP_DIRECT	regulation of circadian rhythm	3	3.06122449	0.03257651	MAPK8, PPARG, PPARA	98	49	16792	10.490629	1	0.18820879	0.16546482
GOTERM_BP_DIRECT	heterophilic cell-cell adhesion via plasma membrane cell adhesion molecules	3	3.06122449	0.0338085	VCAM1, SELE, ICAM1	98	50	16792	10.280816	1	0.19156142	0.16841231
GOTERM_BP_DIRECT	negative regulation of cholesterol storage	2	2.04081633	0.03416768	PPARG, PPARA	98	6	16792	57.115646	1	0.19156142	0.16841231
GOTERM_BP_DIRECT	positive regulation of fatty acid oxidation	2	2.04081633	0.03416768	PPARG, PPARA	98	6	16792	57.115646	1	0.19156142	0.16841231
GOTERM_BP_DIRECT	maternal process involved in parturition	2	2.04081633	0.03416768	CYP1A1, CCL2	98	6	16792	57.115646	1	0.19156142	0.16841231
GOTERM_BP_DIRECT	positive regulation of cAMP metabolic process	2	2.04081633	0.03416768	CXCL10, CXCL11	98	6	16792	57.115646	1	0.19156142	0.16841231
GOTERM_BP_DIRECT	positive regulation of exit from mitosis	2	2.04081633	0.03416768	TGFB1, BIRC5	98	6	16792	57.115646	1	0.19156142	0.16841231
GOTERM_BP_DIRECT	positive regulation of brown fat cell differentiation	2	2.04081633	0.03416768	MAPK14, PTGS2	98	6	16792	57.115646	1	0.19156142	0.16841231
GOTERM_BP_DIRECT	conditioned taste aversion	2	2.04081633	0.03416768	FOS, DRD1	98	6	16792	57.115646	1	0.19156142	0.16841231
GOTERM_BP_DIRECT	lipid hydroxylation	2	2.04081633	0.03416768	CYP1A1, CYP3A4	98	6	16792	57.115646	1	0.19156142	0.16841231
GOTERM_BP_DIRECT	epithelial cell proliferation involved in salivary gland morphogenesis	2	2.04081633	0.03416768	IL6, TNF	98	6	16792	57.115646	1	0.19156142	0.16841231

GOTERM_BP_DIRECT	sensory perception of pain	3	3.06122449	0.03632627	OPRD1, OPRM1, PTGS2	98	52	16792	9.8854003	1	0.20246555	0.17799874
GOTERM_BP_DIRECT	regulation of protein localization	3	3.06122449	0.03632627	MAPK8, BCL2, AKT1	98	52	16792	9.8854003	1	0.20246555	0.17799874
GOTERM_BP_DIRECT	protein dephosphorylation	4	4.08163265	0.03653373	PTPN1, PPP3CA, PTEN, BCL2	98	126	16792	5.4395854	1	0.20302469	0.1784903
GOTERM_BP_DIRECT	dopamine transport	2	2.04081633	0.03974887	DRD1, SLC6A3	98	7	16792	48.956268	1	0.21398893	0.18812958
GOTERM_BP_DIRECT	peroxisome proliferator activated receptor signaling	2	2.04081633	0.03974887	RXRA, PPARG	98	7	16792	48.956268	1	0.21398893	0.18812958
GOTERM_BP_DIRECT	peripheral nervous system myelin maintenance	2	2.04081633	0.03974887	AKT1, SOD1	98	7	16792	48.956268	1	0.21398893	0.18812958
GOTERM_BP_DIRECT	salivary gland morphogenesis	2	2.04081633	0.03974887	TGFB1, EGFR	98	7	16792	48.956268	1	0.21398893	0.18812958
GOTERM_BP_DIRECT	regulation of branching involved in prostate gland	2	2.04081633	0.03974887	RXRA, ESR1	98	7	16792	48.956268	1	0.21398893	0.18812958
GOTERM_BP_DIRECT	cellular response to hyperoxia	2	2.04081633	0.03974887	CAV1, PPARG	98	7	16792	48.956268	1	0.21398893	0.18812958
GOTERM_BP_DIRECT	response to leptin	2	2.04081633	0.03974887	CCND1, SIRT1	98	7	16792	48.956268	1	0.21398893	0.18812958
GOTERM_BP_DIRECT	negative regulation of myelination	2	2.04081633	0.03974887	IFNG, PTEN	98	7	16792	48.956268	1	0.21398893	0.18812958
GOTERM_BP_DIRECT	negative regulation of glial cell apoptotic process	2	2.04081633	0.03974887	CCL2, PRKCA	98	7	16792	48.956268	1	0.21398893	0.18812958
GOTERM_BP_DIRECT	positive regulation of monocyte chemotactic protein-1 production	2	2.04081633	0.03974887	IL1A, IL1B	98	7	16792	48.956268	1	0.21398893	0.18812958
GOTERM_BP_DIRECT	regulation of protein homodimerization activity	2	2.04081633	0.03974887	BCL2, BAX	98	7	16792	48.956268	1	0.21398893	0.18812958
GOTERM_BP_DIRECT	defense response to Gram-negative bacterium	3	3.06122449	0.04023375	IL6, NOS2, SERPINE1	98	55	16792	9.3461967	1	0.21598574	0.18988509
GOTERM_BP_DIRECT	negative regulation of epithelial cell proliferation	3	3.06122449	0.04157018	TGFB1, PTEN, PPARD	98	56	16792	9.1793003	1	0.22190279	0.1950871
GOTERM_BP_DIRECT	cell growth	3	3.06122449	0.04157018	IL6, TGFB1, BCL2	98	56	16792	9.1793003	1	0.22190279	0.1950871
GOTERM_BP_DIRECT	cellular response to interferon-gamma	3	3.06122449	0.04292318	NOS2, MYC, CCL2	98	57	16792	9.0182599	1	0.22720511	0.19974866
GOTERM_BP_DIRECT	learning	3	3.06122449	0.04292318	JUN, DRD1, PTGS2	98	57	16792	9.0182599	1	0.22720511	0.19974866
GOTERM_BP_DIRECT	circadian regulation of gene expression	3	3.06122449	0.04292318	AHR, PPARA, SIRT1	98	57	16792	9.0182599	1	0.22720511	0.19974866
GOTERM_BP_DIRECT	negative regulation of platelet activation	2	2.04081633	0.04529813	THBD, F2	98	8	16792	42.836735	1	0.22829775	0.20070926
GOTERM_BP_DIRECT	low-density lipoprotein particle clearance	2	2.04081633	0.04529813	HMOX1, LDLR	98	8	16792	42.836735	1	0.22829775	0.20070926
GOTERM_BP_DIRECT	positive regulation of IRE1-mediated unfolded protein response	2	2.04081633	0.04529813	PTPN1, BAX	98	8	16792	42.836735	1	0.22829775	0.20070926
GOTERM_BP_DIRECT	flavonoid metabolic process	2	2.04081633	0.04529813	MYC, CYP1A1	98	8	16792	42.836735	1	0.22829775	0.20070926
GOTERM_BP_DIRECT	amine metabolic process	2	2.04081633	0.04529813	VCAM1, CYP1A1	98	8	16792	42.836735	1	0.22829775	0.20070926

GOTERM_BP_DIRECT	positive regulation of ER-associated ubiquitin-dependent protein catabolic process	2	2.04081633	0.04529813	CAV1, NFE2L2	98	8	16792	42.836735	1	0.22829775	0.20070926
GOTERM_BP_DIRECT	positive regulation of MHC class II biosynthetic process	2	2.04081633	0.04529813	IFNG, SIRT1	98	8	16792	42.836735	1	0.22829775	0.20070926
GOTERM_BP_DIRECT	positive regulation of interleukin-6 biosynthetic process	2	2.04081633	0.04529813	IFNG, IL1B	98	8	16792	42.836735	1	0.22829775	0.20070926
GOTERM_BP_DIRECT	cellular response to nicotine	2	2.04081633	0.04529813	TNF, RELA	98	8	16792	42.836735	1	0.22829775	0.20070926
GOTERM_BP_DIRECT	T cell chemotaxis	2	2.04081633	0.04529813	CXCL10, CXCL11	98	8	16792	42.836735	1	0.22829775	0.20070926
GOTERM_BP_DIRECT	regulation of protein heterodimerization activity	2	2.04081633	0.04529813	BCL2, BAX	98	8	16792	42.836735	1	0.22829775	0.20070926
GOTERM_BP_DIRECT	myeloid cell homeostasis	2	2.04081633	0.04529813	BAX, SOD1	98	8	16792	42.836735	1	0.22829775	0.20070926
GOTERM_BP_DIRECT	positive regulation of cyclin-dependent protein serine/threonine kinase activity involved in G1/S transition of	2	2.04081633	0.04529813	AKT1, EGFR	98	8	16792	42.836735	1	0.22829775	0.20070926
GOTERM_BP_DIRECT	negative regulation of cell adhesion mediated by integrin	2	2.04081633	0.04529813	SERPINE1, CYP1B1	98	8	16792	42.836735	1	0.22829775	0.20070926
GOTERM_BP_DIRECT	acute inflammatory response to antigenic stimulus	2	2.04081633	0.04529813	OPRM1, ICAM1	98	8	16792	42.836735	1	0.22829775	0.20070926
GOTERM_BP_DIRECT	monoamine transport	2	2.04081633	0.04529813	SLC6A3, SLC6A4	98	8	16792	42.836735	1	0.22829775	0.20070926
GOTERM_BP_DIRECT	positive regulation of programmed cell death	2	2.04081633	0.04529813	CDKN1A, TNF	98	8	16792	42.836735	1	0.22829775	0.20070926
GOTERM_BP_DIRECT	death-inducing signaling complex	2	2.04081633	0.04529813	CASP8, TNF	98	8	16792	42.836735	1	0.22829775	0.20070926
GOTERM_BP_DIRECT	I-kappaB kinase/NF-kappaB signaling	3	3.06122449	0.04707932	IKBKB, CHUK, TNF	98	60	16792	8.5673469	1	0.23601936	0.20749776
GOTERM_BP_DIRECT	negative regulation of sequence-specific DNA binding transcription factor	3	3.06122449	0.04707932	HMOX1, ESR1, SIRT1	98	60	16792	8.5673469	1	0.23601936	0.20749776
GOTERM_BP_DIRECT	regulation of cell	4	4.08163265	0.04745415	IL6, CCL2, F2, ICAM1	98	140	16792	4.8956268	1	0.23727075	0.20859793
GOTERM_BP_DIRECT	cell adhesion	7	7.14285714	0.04989249	VCAM1, SPP1, CYP1B1, CCL2, PRKCA, SELE, ICAM1	98	459	16792	2.6131341	1	0.24502712	0.21541698
GOTERM_BP_DIRECT	DNA damage response, signal transduction by p53 class mediator resulting in cell cycle	3	3.06122449	0.04992877	CDKN1A, BAX, TP53	98	62	16792	8.2909809	1	0.24502712	0.21541698
GOTERM_BP_DIRECT	SMAD protein signal transduction	3	3.06122449	0.04992877	JUN, TGFB1, FOS	98	62	16792	8.2909809	1	0.24502712	0.21541698
GOTERM_BP_DIRECT	negative regulation of angiogenesis	3	3.06122449	0.04992877	CXCL10, STAT1, CCL2	98	62	16792	8.2909809	1	0.24502712	0.21541698
