# Supplementary material for: Explore the Mechanism of Astragalus mongholicus Bunge against Nonalcoholic Fatty Liver Disease Based on Network Pharmacology and Experimental Verification
Source: Gastroenterol Res Pract. 2022 Apr 5;2022:4745042. doi: 10.1155/2022/4745042 (PMC9005278; doi:10.1155/2022/4745042)
Supplement: Supplementary Materials — Table S1: the bioactive components and corresponding targets of ABM. Table S2: information of NAFLD-related targets collected from the GeneCards database. Table S3: the common targets of ABM and NAFLD. Table S4: the 383 biological process terms. Table S5: the 40 cellular component terms. Table S6: the 63 molecular function terms. [file 4745042.f1.zip › 4745042.f1/Table S5.pdf]

Category	Term	Count	%	PValue	Genes	List Total	Pop Hits	Pop Total	Fold Enrichment	Bonferroni	Benjamini	FDR
GOTERM_CC_DIRECT	extracellular space	33	33.6734694	1.76E-13	CRP, CXCL8, PON1, SERPINE1, HSPB1, MPO, TNF, EGFR, ICAM1, THBD, IGHG1, PLAU, SPP1, CCL2, HMOX1, XDH, CTSD, TGFB1, VCAM1, MMP2, IGFBP3, F2, SELE, MMP9, F3, SOD1, IL1A, CXCL10, COL3A1	98	1347	18224	4.55579292	3.92E-11	3.92E-11	3.48E-11
GOTERM_CC_DIRECT	membrane raft	15	15.3061224	4.38E-12	OPRD1, CAV1, SLC2A4, OPRM1, SELE, TNF, SLC6A3, EGFR, SLC6A4, ICAM1, DPP4, IKBKB, CASP8, CASP3, CTSD	98	206	18224	13.5407173	9.72E-10	4.86E-10	4.31E-10
GOTERM_CC_DIRECT	cytosol	43	43.877551	8.81E-09	GSK3B, CDKN1A, NR1I3, PTEN, HSPB1, HTR2A, SLC2A4, RELA, ACACA, SLC6A4, CASP9, IKBKB, PPP3CA, MAPK8, CASP8, CCND1, MYC, CASP3, AKT1, HMOX1, XDH, NQO1, PTPN1, JUN, HSP90AA1, GSTM1, NOS2, CHUK, STAT1, AHSA1, PRKCA, FOS, MAPK14, SOD1, IL1A, IRF1, IL1B, BCL2, BAX, BIRC5, PPARG, TP53, NFE2L2	98	3315	18224	2.41214024	1.96E-06	6.52E-07	5.79E-07
GOTERM_CC_DIRECT	extracellular region	27	27.5510204	1.90E-07	CRP, PRSS1, CXCL8, PON1, SERPINE1, PTEN, TNF, IGHG1, PLAU, SPP1, CCL2, CTSD, HSP90AA1, TGFB1, MMP1, MMP2, IGFBP3, F2, MMP9, SOD1, IL1A, CXCL10, COL3A1, CXCL11, IL6, IFNG, IL1B	98	1610	18224	3.11857016	4.21E-05	1.05E-05	9.35E-06
GOTERM_CC_DIRECT	extracellular matrix	11	11.2244898	3.97E-06	COL3A1, HSP90AA1, TGFB1, HSPA5, MMP1, MMP2, SERPINE1, HSPB1, CTSD, F3,	98	296	18224	6.91064534	8.81E-04	1.50E-04	1.33E-04
GOTERM_CC_DIRECT	neuron projection	10	10.2040816	4.84E-06	OPRD1, HSP90AA1, CASP8, IFNG, PTEN, FOS, OPRM1, PTGS2, SLC6A3, SLC6A4	98	237	18224	7.84637906	0.001073	1.50E-04	1.33E-04
GOTERM_CC_DIRECT	postsynapse	5	5.10204082	5.13E-06	GABRA2, GSK3B, PPP3CA, AKT1, OPRM1	98	22	18224	42.2634508	0.00113719	1.50E-04	1.33E-04

GOTERM_CC_DIRECT	mitochondrion	22	22.4489796	5.40E-06	GSK3B, HSPA5, MMP2, PTEN, PRKCA, MAPK14, MPO, ELK1, SIRT1, ACACA, SOD1, CASP9, PPP3CA, MAPK8, CASP8, MYC, CYP1A1, BCL2, BAX, CYP1B1, AKT1, TP53	98	1331	18224	3.07370552	0.00119792	1.50E-04	1.33E-04
GOTERM_CC_DIRECT	external side of plasma membrane	9	9.18367347	1.81E-05	CXCL10, IL6, IGHG1, VCAM1, IFNG, SLC2A4, TNF, LDLR, ICAM1	98	213	18224	7.8574303	0.00400902	4.46E-04	3.96E-04
GOTERM_CC_DIRECT	caveola	6	6.12244898	2.47E-05	CAV1, INSR, HMOX1, HTR2A, PTGS2, SELE	98	65	18224	17.1654631	0.00546894	5.01E-04	4.44E-04
GOTERM_CC_DIRECT	perinuclear region of cytoplasm	14	14.2857143	2.48E-05	GSK3B, CDKN1A, HSP90AA1, NOS2, STAT1, CAV1, PRKCA, SLC2A4, SELE, EGFR, SPP1, CCL2, HMOX1, PPARG	98	621	18224	4.19231654	0.00549486	5.01E-04	4.44E-04
GOTERM_CC_DIRECT	cell surface	13	13.2653061	3.03E-05	HSP90AA1, TGFB1, VCAM1, HSPA5, F3, TNF, SLC6A3, EGFR, ICAM1, DPP4, THBD, PLAU,	98	542	18224	4.46027562	0.00671261	5.27E-04	4.67E-04
GOTERM_CC_DIRECT	plasma membrane	41	41.8367347	3.08E-05	OPRD1, CHRM3, GSK3B, SERPINE1, PTEN, HSPB1, ADRB1, ADRB2, HTR2A, SLC2A4, TNF, SLC6A3, EGFR, SLC6A4, ICAM1, DPP4, THBD, PLAU, CASP3, AKT1, HMOX1, DRD1, LDLR, GABRA2, PTPN1, MGAM, HSP90AA1, TGFB1, VCAM1, HSPA5, CAV1, MMP2, INSR, PRKCA, OPRM1, F3, SELE	98	4121	18224	1.85011563	0.00682148	5.27E-04	4.67E-04
GOTERM_CC_DIRECT	nucleoplasm	31	31.6326531	7.60E-05	CDKN1A, NR1I3, NR1I2, PTEN, AHR, ELK1, RELA, PPP3CA, MAPK8, CASP8, RXRA, CCND1, MYC, CASP3, AKT1, JUN, HSP90AA1, CHUK, STAT1, PRKCA, FOS, MAPK14, ESR1, SIRT1, SOD1, IRF1, BIRC5, PPARG, PPARA, TP53, PPARD	98	2784	18224	2.0706662	0.01673375	0.00114018	0.00101178

GOTERM_CC_DIRECT	nuclear chromatin	8	8.16326531	7.70E-05	RXRA, STAT1, IRF1, ESR1, SIRT1, TP53, RELA, PPARD	98	193	18224	7.70815269	0.01695793	0.00114018	0.00101178
GOTERM_CC_DIRECT	extracellular exosome	30	30.6122449	2.28E-04	CRP, PRSS1, PON1, SERPINE1, HSPB1, SLC2A4, MPO, ACACA, ICAM1, DPP4, IGHG1, PLAU, SPP1, CTSD, NQO1, MGAM, HSP90AA1, VCAM1, HSPA5, IGFBP3, INSR, AHSA1, PRKCA, MAPK14, F2, MMP9, F3, SOD1, IL1B, BAX	98	2811	18224	1.98462309	0.04938233	0.00316485	0.00280844
GOTERM_CC_DIRECT	neuronal cell body	9	9.18367347	2.78E-04	GABRA2, NQO1, GSK3B, HSP90AA1, TGFB1, HTR2A, ELK1, SLC6A3, SOD1	98	315	18224	5.31311953	0.05980455	0.003627	0.00321855
GOTERM_CC_DIRECT	nucleus	46	46.9387755	3.75E-04	GSK3B, CDKN1A, NR1I3, NR1I2, PTEN, HSPB1, AHR, ADRB2, PTGS2, MPO, ELK1, RELA, EGFR, CASP9, IKBKB, PPP3CA, MAPK8, RXRA, CCND1, MYC, CASP3, AKT1, HMOX1, DRD1, JUN, HSP90AA1, TGFB1, NOS2, HSPA5, STAT1, MMP2, IGFBP3, FOS, MAPK14, ESR1, SIRT1, SOD1, IRF1, BCL2, BAX, BIRC5, PPARG, PPARA, TP53, PPARD	98	5415	18224	1.57970867	0.07982918	0.00462113	0.00410073
GOTERM_CC_DIRECT	endoplasmic reticulum	13	13.2653061	0.001466	PTPN1, VCAM1, HSPA5, CAV1, AHSA1, PRKCA, FOS, OPRM1, PTGS2, BCL2, BAX, HMOX1,	98	828	18224	2.91964902	0.27790031	0.01712386	0.0151955
GOTERM_CC_DIRECT	axon terminus	4	4.08163265	0.002125	OPRD1, CHRM3, CCL2, ELK1	98	48	18224	15.4965986	0.37646937	0.02359276	0.02093592
GOTERM_CC_DIRECT	apical plasma membrane	7	7.14285714	0.004623	DPP4, HSP90AA1, MGAM, CAV1, PTEN, ADRB2, EGFR	98	291	18224	4.47324497	0.64251471	0.04707054	0.04176981
GOTERM_CC_DIRECT	receptor complex	5	5.10204082	0.004731	RXRA, INSR, ADRB2, LDLR, EGFR	98	127	18224	7.3212277	0.65103429	0.04707054	0.04176981
GOTERM_CC_DIRECT	focal adhesion	8	8.16326531	0.004877	DPP4, HSPA5, PLAU, CAV1, HSPB1, OPRM1, EGFR, ICAM1	98	391	18224	3.80479148	0.66218974	0.04707054	0.04176981

GOTERM_CC_DIRECT	protein complex	8	8.16326531	0.006448	CDKN1A, HSP90AA1, MYC, CAV1, AKT1, PTGS2, TP53, SOD1	98	412	18224	3.61085794	0.76212497	0.05964064	0.05292435
GOTERM_CC_DIRECT	lysosome	6	6.12244898	0.007185	IL1B, ADRB2, MPO, CTSD, LDLR, SOD1	98	226	18224	4.93696948	0.79825112	0.06379897	0.0566144
GOTERM_CC_DIRECT	integral component of plasma membrane	16	16.3265306	0.007676	OPRD1, CHRM3, CAV1, INSR, ADRB1, SLC2A4, ADRB2, HTR2A, OPRM1, TNF, SLC6A3, SLC6A4, ICAM1, THBD, DRD1, [illegible]	98	1415	18224	2.10271868	0.81925627	0.06554235	0.05816145
GOTERM_CC_DIRECT	blood microparticle	5	5.10204082	0.00885	PRSS1, IGHG1, TGFB1, PON1, F2	98	152	18224	6.11707841	0.86102821	0.0727681	0.06457349
GOTERM_CC_DIRECT	organelle membrane	4	4.08163265	0.011222	CYP1A1, CYP1B1, CYP3A4, PTGS2	98	87	18224	8.54984753	0.91835821	0.08897605	0.07895623
GOTERM_CC_DIRECT	basolateral plasma membrane	5	5.10204082	0.015658	CHRM3, HSP90AA1, CAV1, LDLR, EGFR	98	180	18224	5.16553288	0.96991344	0.11704825	0.10386714
GOTERM_CC_DIRECT	cytoplasm	39	39.7959184	0.016319	[illegible] OPRD1, GSK3B, NR1I3, PTEN, HSPB1, AHR, CYP3A4, PTGS2, SLC6A3, RELA, ACACA, EGFR, IKBKB, PPP3CA, CASP8, CCND1, CASP3, AKT1, NQO1, HSP90AA1, TGFB1, GSTM1, NOS2, CHUK, STAT1, AHSA1, PRKCA, MAPK14, ESR1, SIRT1, SOD1, IL6, IFNG, IRF1, BCL2, BAX, BIRC5, TP53, NFE2L2 [illegible]	98	5222	18224	1.38881811	0.97407759	0.11704825	0.10386714
GOTERM_CC_DIRECT	endoplasmic reticulum	11	11.2244898	0.016345	HSPA5, CAV1, BCL2, CYP1A1, BAX, CYP1B1, HMOX1, DRD1, CYP3A4, PTGS2, EGFR	98	862	18224	2.37302903	0.97422824	0.11704825	0.10386714
GOTERM_CC_DIRECT	cytoplasmic side of plasma membrane	3	3.06122449	0.021027	IKBKB, CHUK, PTEN	98	42	18224	13.2827988	0.99106433	0.14587281	0.1294457
GOTERM_CC_DIRECT	axon	5	5.10204082	0.030855	GABRA2, TGFB1, STAT1, HTR2A, SLC6A3	98	222	18224	4.1882699	0.99904874	0.20757042	0.18419537

GOTERM_CC_DIRECT	dendrite	6	6.12244898	0.033335	GABRA2, CHRM3, STAT1, CCL2, HTR2A, ELK1	98	335	18224	3.33061224	0.99946139	0.20813939	0.18470027
GOTERM_CC_DIRECT	endocytic vesicle	3	3.06122449	0.033543	DPP4, CCL2, EGFR	98	54	18224	10.3310658	0.99948647	0.20813939	0.18470027
GOTERM_CC_DIRECT	early endosome	5	5.10204082	0.034008	PTPN1, VCAM1, ADRB1, ADRB2, LDLR	98	229	18224	4.06024419	0.99953857	0.20813939	0.18470027
GOTERM_CC_DIRECT	clathrin-coated pit	3	3.06122449	0.03469	SLC2A4, SELE, LDLR	98	55	18224	10.1432282	0.99960551	0.20813939	0.18470027
GOTERM_CC_DIRECT	death-inducing signaling complex	2	2.04081633	0.036675	CASP8, CASP3	98	7	18224	53.1311953	0.9997502	0.21425809	0.19012993
GOTERM_CC_DIRECT	pore complex	2	2.04081633	0.046907	BCL2, BAX	98	9	18224	41.324263	0.99997666	0.26384982	0.23413701
GOTERM_CC_DIRECT	myelin sheath	4	4.08163265	0.047541	HSP90AA1, HSPA5, BCL2, SOD1	98	152	18224	4.89366273	0.99997987	0.26384982	0.23413701
