# Supplementary material for: Explore the Mechanism of Astragalus mongholicus Bunge against Nonalcoholic Fatty Liver Disease Based on Network Pharmacology and Experimental Verification
Source: Gastroenterol Res Pract. 2022 Apr 5;2022:4745042. doi: 10.1155/2022/4745042 (PMC9005278; doi:10.1155/2022/4745042)
Supplement: Supplementary Materials — Table S1: the bioactive components and corresponding targets of ABM. Table S2: information of NAFLD-related targets collected from the GeneCards database. Table S3: the common targets of ABM and NAFLD. Table S4: the 383 biological process terms. Table S5: the 40 cellular component terms. Table S6: the 63 molecular function terms. [file 4745042.f1.zip › 4745042.f1/Table S6.pdf]

Category	Term	Count	%	PValue	Genes	List Total	Pop Hits	Pop Total	Fold Enrichment	Bonferroni	Benjamini	FDR
GOTERM_MF_DIRECT	enzyme binding	25	25.5102041	4.70E-20	PTEN, CYP3A4, PTGS2, EGFR, PPP3CA, MAPK8, RXRA, CCND1, AKT1, HMOX1, PTPN1, JUN, TGFB1, GSTM1, HSPA5, STAT1, CAV1, PRKCA, MAPK14, ESR1, SIRT1, CYP1A1	98	333	16881	12.9320647	1.86E-17	1.86E-17	1.70E-17
GOTERM_MF_DIRECT	protein binding	82	83.6734694	7.42E-11	CHRM3, GSK3B, CDKN1A, CXCL8, SERPINE1, PTEN, HSPB1, AHR, SLC2A4, ELK1, TNF, SLC6A3, SLC6A4, ICAM1, CASP9, IKBKB, THBD, PPP3CA, IGHG1, CASP8, CCND1, PLAU, MYC, CASP3, AKT1, CTSD, HSP90AA1, CHUK, IGFBP3, MMP2, AHSA1, PRKCA, FOS, OPRM1, F2, MMP9, F3, SIRT1, IL1A, IRF1, BIRC5, PPARG, PPARA, TP53, PPARD, CRP, OPRD1, NR1I2, ADRB1, ADRB2, PTGS2, ACACA, EGFR, RELA, DPP4, MAPK8, RXRA, SPP1, HMOX1, DRD1, LDLR, PTPN1, NQO1, JUN, TGFB1, NOS2, HSPA5, STAT1, INSR, CAV1, MAPK14, SELE, ESR1, SOD1, CXCL10	98	8785	16881	1.607845	2.93E-08	1.47E-08	1.34E-08
GOTERM_MF_DIRECT	identical protein binding	23	23.4693878	1.88E-10	NQO1, JUN, HSP90AA1, STAT1, CAV1, PTEN, HSPB1, ESR1, SIRT1, TNF, MMP9, EGFR, RELA, SOD1, DPP4, CASP8, BCL2, BAX, BIRC5, AKT1	98	749	16881	5.28954252	7.42E-08	2.47E-08	2.27E-08
GOTERM_MF_DIRECT	RNA polymerase II transcription factor activity, ligand-activated sequence-specific DNA	8	8.16326531	1.21E-09	RXRA, NR1I3, NR1I2, PPARG, AHR, PPARA, ESR1, PPARD	98	36	16881	38.2789116	4.78E-07	1.19E-07	1.09E-07

GOTERM_MF_DIRECT	protein heterodimerization activity	17	17.3469388	8.68E-09	JUN, TGFB1, CHUK, CAV1, ADRB1, AHR, FOS, EGFR, RELA, IKBKB, PPP3CA, RXRA, BCL2, BAX, BIRC5, TP53, PPARD	98	465	16881	6.29749835	3.43E-06	6.86E-07	6.29E-07
GOTERM_MF_DIRECT	protein homodimerization activity	20	20.4081633	2.80E-08	JUN, HSP90AA1, TGFB1, GSTM1, NOS2, CHUK, STAT1, PON1, ADRB2, PTGS2, RELA, SLC6A4, SOD1, DPP4, IKBKB, BCL2, BAX, BIRC5, HMOX1	98	730	16881	4.71931786	1.11E-05	1.84E-06	1.69E-06
GOTERM_MF_DIRECT	transcription factor binding	13	13.2653061	8.14E-08	JUN, AHR, FOS, ESR1, SIRT1, RELA, CCND1, MYC, BCL2, PPARG, PPARA, TP53, PPARD	98	284	16881	7.88491664	3.22E-05	4.60E-06	4.21E-06
GOTERM_MF_DIRECT	protease binding	9	9.18367347	1.16E-07	DPP4, CASP3, SERPINE1, BCL2, TNF, TP53, F3, LDLR, SLC6A3	98	101	16881	15.3494645	4.56E-05	5.71E-06	5.23E-06
GOTERM_MF_DIRECT	drug binding	8	8.16326531	2.63E-07	PPP3CA, CHRM3, NR1I2, PPARG, HTR2A, PPARA, SLC6A3, PPARD	98	76	16881	18.132116	1.04E-04	1.15E-05	1.06E-05
GOTERM_MF_DIRECT	protein phosphatase 2A binding	6	6.12244898	4.12E-07	PTPN1, STAT1, BCL2, AKT1, TP53, SLC6A3	98	27	16881	38.2789116	1.63E-04	1.63E-05	1.49E-05
GOTERM_MF_DIRECT	steroid hormone receptor activity	7	7.14285714	7.93E-07	RXRA, NR1I3, NR1I2, PPARG, PPARA, ESR1, PPARD	98	56	16881	21.5318878	3.13E-04	2.85E-05	2.61E-05
GOTERM_MF_DIRECT	sequence-specific DNA binding	15	15.3061224	1.49E-06	JUN, NR1I3, NR1I2, FOS, ELK1, ESR1, RXRA, MYC, IRF1, BCL2, PPARG, PPARA, TP53, NFE2L2, PPARD	98	518	16881	4.98808211	5.88E-04	4.90E-05	4.49E-05
GOTERM_MF_DIRECT	nitric-oxide synthase regulator activity	4	4.08163265	1.01E-05	HSP90AA1, AKT1, ESR1, EGFR	98	8	16881	86.127551	0.00397637	3.06E-04	2.81E-04
GOTERM_MF_DIRECT	serine-type endopeptidase activity	10	10.2040816	1.60E-05	DPP4, PRSS1, IGHG1, PLAU, MMP1, MMP2, F2, CTSD, F3, MMP9	98	255	16881	6.75510204	0.00629011	4.51E-04	4.13E-04
GOTERM_MF_DIRECT	protein complex binding	9	9.18367347	2.45E-05	CDKN1A, CASP8, CCND1, MYC, CASP3, HTR2A, PPARA, RELA, SLC6A3	98	206	16881	7.52570834	0.00962492	6.45E-04	5.91E-04

GOTERM_MF_DIRECT	transcription regulatory region DNA [illegible]	9	9.18367347	3.11E-05	JUN, RXRA, PPARG, FOS, AHR, TNF, TP53, RELA, NFE2L2	98	213	16881	7.27838459	0.01220857	7.68E-04	7.04E-04
GOTERM_MF_DIRECT	protein kinase binding	11	11.2244898	6.00E-05	CASP9, IKBKB, GSK3B, PTPN1, CCND1, CAV1, PTEN, HSPB1, TP53, RELA, EGFR	98	376	16881	5.03937799	0.02340349	0.001393	0.00127662
GOTERM_MF_DIRECT	chaperone binding	6	6.12244898	1.02E-04	HSPA5, BAX, AHSA1, BIRC5, TP53, SOD1	98	81	16881	12.7596372	0.03965482	0.0022478	0.00206001
GOTERM_MF_DIRECT	transcription factor activity, sequence-specific DNA [illegible]	17	17.3469388	1.10E-04	JUN, STAT1, NR1I3, NR1I2, AHR, FOS, ELK1, ESR1, RELA, RXRA, MYC, IRF1, PPARG, PPARA, TP53, NFE2L2, PPARD	98	961	16881	3.04717662	0.04244962	0.00228287	0.00209215
GOTERM_MF_DIRECT	heme binding	7	7.14285714	1.38E-04	NOS2, CYP1A1, CYP1B1, HMOX1, CYP3A4, PTGS2, MPO	98	137	16881	8.80135558	0.05309565	0.00272767	0.00249979
GOTERM_MF_DIRECT	transcriptional activator activity, RNA polymerase II core promoter proximal region sequence-specific	8	8.16326531	4.22E-04	JUN, MYC, IRF1, FOS, PPARA, ELK1, ESR1, RELA	98	236	16881	5.839156	0.15351836	0.00793484	0.00727193
GOTERM_MF_DIRECT	protein phosphatase	5	5.10204082	4.71E-04	HSP90AA1, PPARG, MAPK14, TP53, EGFR	98	63	16881	13.6710398	0.16968946	0.00845053	0.00774454
GOTERM_MF_DIRECT	glycoprotein binding	5	5.10204082	5.30E-04	HSP90AA1, TGFB1, HSPA5, LDLR, EGFR	98	65	16881	13.2503925	0.189028	0.00875619	0.00802466

GOTERM_MF_DIRECT	cytokine activity	7	7.14285714	5.32E-04	IL1A, IL6, TGFB1, IFNG, IL1B, SPP1, TNF	98	176	16881	6.85105519	0.18958151	0.00875619	0.00802466
GOTERM_MF_DIRECT	RNA polymerase II core promoter proximal region sequence-specific DNA	9	9.18367347	0.00100486	JUN, STAT1, MYC, IRF1, FOS, PPARA, ELK1, ESR1, RELA	98	355	16881	4.36703076	0.32774731	0.01587686	0.01455044
GOTERM_MF_DIRECT	protein kinase activity	9	9.18367347	0.00107971	IKBKB, GSK3B, CCND1, CHUK, CCL2, AKT1, PRKCA, MAPK14, EGFR	98	359	16881	4.31837303	0.3473507	0.01640327	0.01503287
GOTERM_MF_DIRECT	double-stranded DNA binding	5	5.10204082	0.00121503	STAT1, MYC, ELK1, TP53, EGFR	98	81	16881	10.633031	0.38135907	0.0177755	0.01629046
GOTERM_MF_DIRECT	ubiquitin protein ligase binding	8	8.16326531	0.001331	GSK3B, CDKN1A, CASP8, HSPA5, BCL2, TP53, RELA, EGFR	98	287	16881	4.80153595	0.40909108	0.01877654	0.01720787
GOTERM_MF_DIRECT	chromatin binding	9	9.18367347	0.00185301	JUN, PPARG, FOS, MPO, ELK1, ESR1, TP53, RELA, EGFR	98	391	16881	3.9649512	0.51935028	0.02523922	0.02313063
GOTERM_MF_DIRECT	core promoter sequence-specific DNA [illegible]	4	4.08163265	0.00192141	PPARG, ESR1, SIRT1, TP53	98	43	16881	16.0237304	0.53218795	0.02529862	0.02318506
GOTERM_MF_DIRECT	cysteine-type endopeptidase activity involved in apoptotic	3	3.06122449	0.00244594	CASP9, CASP8, CASP3	98	13	16881	39.7511774	0.61990405	0.03116607	0.02856232

GOTERM_MF_DIRECT	chemokine activity	4	4.08163265	0.00279807	CXCL10, CXCL11, CXCL8, CCL2	98	49	16881	14.061641	0.66938026	0.03453866	0.03165315
GOTERM_MF_DIRECT	oxidoreductase activity, acting on paired donors, with incorporation or reduction of molecular oxygen, reduced flavin or flavoprotein as one donor, and incorpora	3	3.06122449	0.00326808	CYP1A1, CYP1B1, CYP3A4	98	15	16881	34.4510204	0.7255549	0.03911788	0.0358498
GOTERM_MF_DIRECT	receptor binding	8	8.16326531	0.00423487	DPP4, CXCL10, NOS2, CAV1, SERPINE1, CCL2, F2, SLC6A3	98	353	16881	3.90379835	0.81293972	0.04919919	0.04508888
GOTERM_MF_DIRECT	cysteine-type endopeptidase activity	4	4.08163265	0.00520131	CASP9, CASP8, CASP3, CTSD	98	61	16881	11.2954165	0.87253063	0.0587005	0.05379641
GOTERM_MF_DIRECT	zinc ion binding	15	15.3061224	0.00681086	PTPN1, MMP1, NR1I3, MMP2, NR1I2, PRKCA, ESR1, MMP9, SOD1, RXRA, BIRC5, PPARG, PPARA, TP53, PPARD	98	1169	16881	2.21028788	0.93276053	0.07473032	0.06848703
GOTERM_MF_DIRECT	virus receptor activity	4	4.08163265	0.00762292	DPP4, HTR2A, LDLR, ICAM1	98	70	16881	9.84314869	0.95132486	0.08137978	0.07458096
GOTERM_MF_DIRECT	aromatase activity	3	3.06122449	0.0104466	CYP1A1, CYP1B1, CYP3A4	98	27	16881	19.1394558	0.98420577	0.10858962	0.09951758

GOTERM_MF_DIRECT	lipid binding	5	5.10204082	0.01121283	PTEN, BAX, PPARA, PTGS2, PPARD	98	151	16881	5.70381133	0.98836904	0.11316287	0.10370875
GOTERM_MF_DIRECT	vitamin D 24-hydroxylase activity	2	2.04081633	0.01145953	CYP1A1, CYP3A4	98	2	16881	172.255102	0.98946077	0.11316287	0.10370875
GOTERM_MF_DIRECT	tumor necrosis factor receptor binding	3	3.06122449	0.01199401	CASP8, STAT1, TNF	98	29	16881	17.8194933	0.99148798	0.11555208	0.10589836
GOTERM_MF_DIRECT	kinase activity	6	6.12244898	0.01267021	GSK3B, CDKN1A, MAPK8, PLAU, AKT1, PRKCA	98	241	16881	4.28850876	0.99350491	0.11760962	0.10778401
GOTERM_MF_DIRECT	NF-kappaB binding	3	3.06122449	0.01280307	GSK3B, RELA, PPARD	98	30	16881	17.2255102	0.99384116	0.11760962	0.10778401
GOTERM_MF_DIRECT	IkappaB kinase activity	2	2.04081633	0.01714046	IKBKB, CHUK	98	3	16881	114.836735	0.99891825	0.1538746	0.14101925
GOTERM_MF_DIRECT	protein N-terminus	4	4.08163265	0.01837868	TGFB1, TP53, RELA, SLC6A3	98	97	16881	7.10330318	0.99934254	0.16132399	0.14784629
GOTERM_MF_DIRECT	histone deacetylase	4	4.08163265	0.02096991	HSP90AA1, MAPK8, CCND1, RELA	98	102	16881	6.75510204	0.99976856	0.18006775	0.16502411
GOTERM_MF_DIRECT	integrin binding	4	4.08163265	0.02261607	COL3A1, VCAM1, EGFR, ICAM1	98	105	16881	6.56209913	0.99988094	0.18370789	0.16836014
GOTERM_MF_DIRECT	BH3 domain binding	2	2.04081633	0.02278908	BCL2, BAX	98	4	16881	86.127551	0.99988898	0.18370789	0.16836014
GOTERM_MF_DIRECT	norepinephrine binding	2	2.04081633	0.02278908	ADRB1, ADRB2	98	4	16881	86.127551	0.99988898	0.18370789	0.16836014
GOTERM_MF_DIRECT	CXCR3 chemokine receptor binding	2	2.04081633	0.02840557	CXCL10, CXCL11	98	5	16881	68.9020408	0.99998861	0.21577306	0.19774645

GOTERM_MF_DIRECT	superoxide dismutase activity	2	2.04081633	0.02840557	NQO1, SOD1	98	5	16881	68.9020408	0.99998861	0.21577306	0.19774645
GOTERM_MF_DIRECT	NFAT protein binding	2	2.04081633	0.02840557	MAPK14, PPARA	98	5	16881	68.9020408	0.99998861	0.21577306	0.19774645
GOTERM_MF_DIRECT	oxygen binding	3	3.06122449	0.02987658	CYP1A1, CYP1B1, CYP3A4	98	47	16881	10.9950065	0.99999374	0.21854164	0.20028373
GOTERM_MF_DIRECT	RNA polymerase II transcription factor [illegible]	3	3.06122449	0.02987658	GSK3B, ELK1, TP53	98	47	16881	10.9950065	0.99999374	0.21854164	0.20028373
GOTERM_MF_DIRECT	scaffold protein binding	3	3.06122449	0.03106088	IKBKB, CASP8, CHUK	98	48	16881	10.7659439	0.99999614	0.22140206	0.20290518
GOTERM_MF_DIRECT	protein domain specific binding	5	5.10204082	0.03194916	HSPA5, IL1B, OPRM1, PPARA, NFE2L2	98	208	16881	4.14074765	0.99999731	0.22140206	0.20290518
GOTERM_MF_DIRECT	RNA polymerase II regulatory region sequence-specific DNA	5	5.10204082	0.03194916	RXRA, NR1I3, NR1I2, TP53, RELA	98	208	16881	4.14074765	0.99999731	0.22140206	0.20290518
GOTERM_MF_DIRECT	epinephrine binding	2	2.04081633	0.0339901	ADRB1, ADRB2	98	6	16881	57.4183673	0.99999883	0.23148433	0.21214514
GOTERM_MF_DIRECT	monoamine transmembrane transporter [illegible]	2	2.04081633	0.03954287	SLC6A3, SLC6A4	98	7	16881	49.2157434	0.99999988	0.26473618	0.24261898

GOTERM_MF_DIRECT	copper ion binding	3	3.06122449	0.04117632	IL1A, TP53, SOD1	98	56	16881	9.2279519	0.99999994	0.27107745	0.24843048
GOTERM_MF_DIRECT	monooxygenase activity	3	3.06122449	0.04387454	CYP1A1, CYP1B1, CYP3A4	98	58	16881	8.90974666	0.99999998	0.28254444	0.25893946
GOTERM_MF_DIRECT	insulin-like growth factor II binding	2	2.04081633	0.04506405	INSR, IGFBP3	98	8	16881	43.0637755	0.99999999	0.28254444	0.25893946
GOTERM_MF_DIRECT	thyroid hormone receptor activity	2	2.04081633	0.04506405	NR1I3, NR1I2	98	8	16881	43.0637755	0.99999999	0.28254444	0.25893946
